# Global patterns and trends of suicide mortality and years of life lost among adolescents and young adults from 1990 to 2021: a systematic analysis for the Global Burden of Disease Study 2021

**DOI:** 10.1017/S2045796024000532

**Published:** 2024-10-21

**Authors:** Na Yan, Yunjiao Luo, Louisa Esi Mackay, Yuhao Wang, Yingxue Wang, Yihan Wang, Blen Dereje Shiferaw, Jingjing Wang, Jie Tang, Wenjun Yan, Qingzhi Wang, Xiuyin Gao, Wei Wang

**Affiliations:** 1 School of Public Health, Xuzhou Medical University, Xuzhou, China; 2 Research Center for Mental Crisis Prevention and Intervention of College Students in Jiangsu Province, Xuzhou Medical University, Xuzhou, China; 3 Jiangsu Engineering Research Center of Biological Data Mining and Healthcare Transformation, Xuzhou Medical University, Xuzhou, China

**Keywords:** age-period-cohort, joinpoint analysis, mortality, suicide, years of life lost

## Abstract

**Aims:**

We aimed to report an overview of trends in suicide mortality and years of life lost (YLLs) among adolescents and young adults aged 10–24 years by sex, age group, Socio-demographic Index (SDI), region and country from 1990 to 2021 as well as the suicide mortality with age, period and birth cohort effects.

**Methods:**

Estimates and 95% uncertainty intervals for suicide mortality and YLLs were extracted from the Global Burden of Diseases Study 2021. Joinpoint analysis was used to calculate the annual percentage change (APC) and average annual percentage change (AAPC) to describe the mortality and rate of YLLs trends. Age, period and cohort model was utilized to disentangle age, period and birth cohort effects on suicide mortality trends.

**Results:**

Globally, suicide mortality and the rate of YLLs among adolescents and young adults both declined from 1990 to 2021 (AAPC: −1.6 [−2.1 to −1.2]). In 2021, the global number of suicide death cases was 112.9 thousand [103.9–122.2 thousand] and led to 7.9 million [7.2–8.6 million] YLLs. A significant reduction in suicide mortality was observed in all sexes and age groups. By SDI quintiles, the high SDI region (AAPC: −0.3 [−0.6 to 0.0]) had the slowest decline trend, and low-middle SDI region remained the highest suicide mortality till 2021 (7.8 per 100,000 population [6.9–8.6]). Most SDI regions showed generally lower period and cohort effects during the study period, whereas high SDI region showed more unfavourable risks, especially period and cohort effects in females. Regionally, Central Latin America (AAPC: 1.7 [1.1–2.3]), Tropical Latin America (AAPC: 1.5 [0.9–2.0]), High-income Asia Pacific (AAPC: 1.2 [0.7–1.7]) and Southern sub-Saharan Africa (AAPC: 0.8 [0.4–1.2]) had the significance increase in suicide mortality. In 2021, Southern sub-Saharan Africa had the highest mortality (10.5 per 100,000 population [8.6–12.5]). Nationally, a total of 29 countries had a significant upward trend in suicide mortality and rate of YLLs over the past three decades, and certain countries in low-middle and middle regions exhibited an extremely higher burden of suicide.

**Conclusions:**

Global suicide mortality and the rate of YLLs among adolescents and young adults both declined from 1990 to 2021, but obvious variability was observed across regions and countries. Earlier mental health education and targeted management are urgently required for adolescents and young adults in certain areas.

## Introduction

Globally, suicide is a significant public health issue. Approximately 703,000 people take their own lives each year (World Health Organization, 2023). In 2016, suicide was one of the top 10 leading causes of death in Eastern, Central and Western Europe, Central Asia, Oceania, southern Latin America and high-income North America (Naghavi, 2019). Despite a significant decrease in suicide rate from 1990 to 2019 (from 13.8% to 9.8% per 100,000 population), the overall number of suicide deaths increased by 19,897 (Yip *et al.*, 2022). Suicide in adolescents and young adults is the end product of a complex interplay between genetic, biological, psychiatric, psychological, social and cultural factors (Hawton *et al.*, 2012). Psychological problems such as avoidance, impulsivity, depression and anxiety in the youth can lead to suicide, with some of the most extreme groups of the population seeing death as a last resort to solve their problems (Hawton *et al.*, 2012; Townsend, 2014). Therefore, the prevalence of suicide increases significantly throughout adolescence, despite it is a relatively rare occurrence among young people (Kõlves and de Leo, 2017).

Previous studies have also reported the prevalence of suicide among young people. According to the World Health Organization (WHO), suicide is the second leading cause of death among people aged 15–19 years worldwide, and also the third leading cause of disability-adjusted life years for the 10–24 years population (GBD 2019 Diseases and Injuries Collaborators, 2020). Although the overall suicide mortality for 5–19 years adolescents decreased from 4.74 per 100,000 population in 1990 to 2.70 per 100,000 population in 2019 (Kim *et al.*, 2024), the global suicide rate is still high, accounting for 4.2% of overall death cases among this population (Liu *et al.*, 2022). Regionally, the worrying phenomenon of suicide occurred in certain areas. Fifteen percent of adolescents in low- and middle-income countries had suicidal thoughts (United Nations, 2019). In America, suicide mortality for people aged 10–24 increased continuously from 2007 through 2021 (from 6.8 to 11.0 per 100,000 population) (Centers for Disease Control and Prevention, 2023).

It is well known the epidemic of suicide varies significantly by sex, age and geographic region, and is also influenced by local economic, political and cultural changes (Turecki and Brent, 2016). Currently, the trend of suicide mortality and the authentic extent of the burden from suicide among adolescents and young adults in specific regions and countries is still unknown. Although some studies have reported on global suicide, some national data are omitted and have not been systematically analysed for the 10-24-year-old population who are at high risk for suicide. Furthermore, since the COVID-19 pandemic, the government has implemented strict quarantine measures, thus posing significant challenges to young people’s psychological well-being. Trends in suicide mortality in the post-epidemic era have not been addressed. Therefore, we aim to use data from the Global Burden of Disease 2021 to explore the global burden and changing trends in suicide mortality and years of life lost (YLLs) among adolescents and young adults from 1990 to 2021. The purposes of this study are: (1) to describe the mortality, YLLs of suicide in males and females aged 10–14, 15–19 and 20–24 years, from 1990 to 2021 among Socio-demographic Index (SDI) quintiles, 21 regions and 204 countries; (2) to determine the association of SDI level with suicide mortality and rate of YLLs by each region and country; (3) to report the association of suicide mortality with age, period and cohort effects across SDI quintiles, 21 regions and countries.

## Methods

### Data sources

The Global Burden of Disease Study (GBD) 2021 includes estimates of mortality and YLLs by location, age group and sex from 371 causes (including suicide), in 204 countries and territories from 1990 to 2021 (GBD 2021 Diseases and Injuries Collaborators, 2024). The GBD data uses the International Classification of Diseases (ICD) to define suicide mortality as death due to intentional self-inflicted poisoning or injury (ICD-10 codes: X60-X64.9, X66-X84.9, Y87.0; ICD-9 codes: E950-E959) and provides comprehensive suicide mortality and YLLs for assessment. In GBD 2021, data on suicide could extended back to 1980, but considering the sparser of the data before 1990 in some developing countries, we restricted our analysis from 1990 to 2021.

The major data sources for suicide mortality estimation for GBD 2021 include hospital records, emergency department records, insurance claims and population-representative surveys (GBD 2021 Diseases and Injuries Collaborators, 2024). The quality and comparability of cause of death data for suicide was assessed and improved through multiple methods. These included data standardization and redistribution of inappropriately coded death or ‘junk codes’. Undercounting or misclassification of suicide deaths is a known problem in the estimation of suicide deaths, with the extent and type of misclassification varying by location, age, sex and time. The misassignment is partially corrected by reallocating ICD codes that may include suicide deaths (GBD 2016 Causes of Death Collaborators, 2017; India State-Level Disease Burden Initiative Suicide Collaborators, 2018). Mortality was modelled using the Death Ensemble model (CODEm). This modelling approach can estimate a more accurate measure of uncertainty than other modelling techniques, thereby improving the accuracy of the final mortality estimate and reducing uncertainty. To YLLs, each death caused by suicide was multiplied by the standard life expectancy at that age. The specific modelling process for suicide data can be seen in Supplementary Method S1.

The mortality, number of deaths and YLLs of suicide were extracted directly from GBD 2021 at the disease surveillance system on the Global Health Data Exchange website (http://ghdx.healthdata.org). The 95% uncertainty interval is defined by the 2.5th and 97.5th values of the ordered 1000 estimates based on the GBD algorithm (GBD 2021 Diseases and Injuries Collaborators, 2024). Based on the data provided by GBD, we also used each country’s SDI, a measure that combines per capita income, average years of schooling and fertility rates for women under 25 years old (GBD 2021 Diseases and Injuries Collaborators, 2024). SDI ranges from 0 to 1, with higher values indicating higher socio-economic levels. Based on the 2021 SDI values, all countries were included in one of the five SDI quintile regions. We collected suicide data from sexes, three age groups (10–14 years, 15–19 years and 20–24 years), as well as SDI regions, 21 regional groups and 204 countries at life stages from childhood to adulthood. We compared the GBD 2021 data on suicide death with those published by the WHO for 2021. The result showed they were highly comparable (Supplementary Table 1).

### Data analysis

#### Joinpoint analysis

Joinpoint regression program version 5.0.2 (National Cancer Institute, 2024) was used to report trends of suicide mortality and rate of YLLs across different sexes, age groups, SDI qualities, regions and countries from 1990 to 2021. The model is calculated by using the least squares method to estimate the pattern of change in mortality and rate of YLLs, avoiding the non-objectivity of typical trend analyses based on linear trends. Calculating the sum of the squares of the residuals between the estimated and actual values gives the joinpoints of the moving trend. Based on the officially recommended joinpoint number, we set the maximum number of joinpoints to 6 (National Cancer Institute, 2024). For each statistically significant segment of the time trend, the model gives the average percentage change (APC) reflecting the rates of change between the two connecting points. The model also gives values for the average annual percentage change (AAPC), which describes the overall rate of change in suicide. The *Z* test is used to determine whether AAPC or APC is different from 0. If APC/AAPC > 0, the trend is upward. If APC/AAPC < 0, the trend is downward.

#### Correlation with SDI

Gaussian curves was used in this study to determine the relationship of each region or country’s socio-economic development status with mortality and rate of YLLs for suicide. Based on SDI and observed mortality and rate of YLLs in all regions and countries, the expected mortality and rate of YLLs were analysed, and assessed by the Spearman rank order correlation tests (Jin *et al.*, 2021).

#### Age, period and cohort analysis

We used the web-based age, period and cohort model (https://analysistools.cancer.gov/apc/) to analyse the effect of age, period and cohort on suicide mortality trends across SDI qualities, regions and countries. GBD 2021 estimates for suicide death cases and population data of each country/region were used as data inputs for the age, period and cohort model. Before formal analysis, a total of 15 countries were excluded because the number of suicide deaths among 10–24-year-olds was less than 1 for several consecutive years (the details can be seen in Supplementary Method S2). In a typical age, period and cohort model, the age and period interval must be equal, so ages are divided into 5-year age groups (10–14 years, 15–19 years and 20–24 years) (Bell, 2020; Yang *et al.*, 2018), and periods are divided into 5-year groups (1992–1996, 1997–2001, …, 2017–2021). Finally, eight partially coincidental birth cohorts are generated by age groups and period groups (1967–1976, 1972–1981, …, 2022–2011). The age effect is represented longitudinally by a fit of a given number of birth cohorts adjusted for period bias to a specific age rate. The period/cohorteffect is expressed by the period/cohort relative mortality, which is calculated as the rate of age-specific mortality in each period/cohort relative to the reference period/cohort. In our study, we chose 10 years old as the reference age and 1990 as the reference period. The choice of reference did not affect the interpretation of the results (Rosenberg *et al.*, 2014). The statistical significance of these parameters was tested by the Wald *χ*^2^, with a significance level of 0.05. These processes were completed in R 4.2.1.

## Results

### Joinpoint regression analysis of suicide mortality and YLLs

#### Global trends

**Figure 1.**
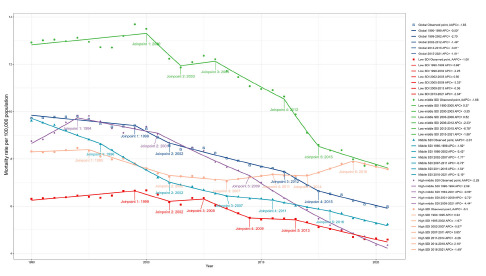
Joinpoint regression analysis of suicide mortality aged 10-24 years from 1990 to 2021 in global and 5 SDI regions.

Globally, the mortality and rate of YLLs of suicide among adolescents and young adults were both on a downward trend. The suicide mortality in 2021 (6.0 per 100,000 population [5.5–6.5] was lower than in 1990 (9.7 per 100,000 population [8.0–10.5]). The global rate of YLLs also declined from 1990 (684.3 per 100,000 population [561.9–735.4]) to 2021 (419.4 per 100,000 population [385.7–454.1]). Joinpoint regression analysis identified similar trends of suicide mortality and rate of YLLs, with four substantial changes for the trends in 1999, 2002, 2012 and 2015, and both with an average decrease (AAPC: −1.6 [−2.1 to −1.2], Figure 1) per year. The mortality experienced a fast downward trend in 2012–2015 (APC: −3.8 [−6.4 to −1.2]).

#### Global trends by sex and age group

The mortality declined for both sexes in the past three decades, and females fell faster than males (AAPC for males: −1.2 [−1.5 to −1.0]; AAPC for females: −2.1 [−2.5 to −1.7], Table 1, Supplementary Figure S1). Meanwhile, males kept a consistently higher mortality and rate of YLLs than females from 1990 to 2021. Among the 112,936 global suicide death cases in the 10–24 years population in 2021, 69,247 (61.3%) occurred in males, thus accounting for 4,837,216 (61.1%) YLLs. The mortality in three age groups all showed a decreasing trend from 1990 to 2021, and the adolescents in 20–24 years experienced the slowest decrease (AAPC: −1.4 [−1.9 to −0.9], Table 1, Supplementary Figure S3). As the age increased, the burden of suicide mortality increased and peaked at the age of 20–24. A total of 63,237 (56%) death cases occurred in 20–24 years young adults, which accounted for 4,275,913 (54%) YLLs in 2021.

**Table 1.** The mortality and rate of YLLs of suicide and their AAPC in both sexes from 1990 to 2021 at the global and region levels

	Mortality						YLLs					
Group	Cases (*n*), 1990	Mortality (per 100,000 population), 1990	Cases (*n*), 2021	Mortality (per 100,000 population), 2021	AAPC: 1990–2021	*P* value	Cases (*n*), 1990	YLLs (per 100,000 population), 1990	Cases (*n*), 2021	YLLs (per 100,000 population), 2021	AAPC: 1990–2021	*P* value
**Global**	150,497 (123,474–161,707)	9.7 (8.0–10.5)	112,936 (103,870–122,216)	6.0 (5.5–6.5)	−1.6 (−2.1 to −1.2)	<0.001	10,587,607 (8,693,231–11,378,273)	684.3 (561.9–735.4)	7,917,201 (7,281,085–8,572,099)	419.4 (385.7–454.1)	−1.6 (−2.1 to −1.2)	<0.001
**Sex**												
Male	81,993 (70,320–88,344)	10.4 (8.9–11.2)	69,247 (64,304–74,751)	7.2 (6.6–7.7)	−1.2 (−1.5 to −1.0)	<0.001	5,760,246 (4,939,755–6,204,874)	732.8 (628.4–789.3)	4,837,216 (4,494,376–5,216,642)	500.0 (464.6–539.2)	−1.3 (−1.5 to −1.0)	<0.001
Female	68,504 (48,974–76,706)	9.0 (6.4–10.1)	43,689 (38,897–49,576)	4.7 (4.2–5.4)	−2.1 (−2.5 to −1.7)	<0.001	4,827,360 (3,453,306–5,404,246)	634.3 (453.7–710.1)	3,079,985 (2,743,466–3,488,323)	334.7 (298.1–379)	−2.1 (−2.5 to −1.7)	<0.001
**Age group, years**												
10–14	11,951 (9,265–13,299)	2.2 (1.7–2.5)	7,777 (6,795–9,379)	1.2 (1.0–1.4)	−2.1 (−2.6 to −1.6)	<0.001	926,667 (718,418–1,031,196)	173.0 (134.1–192.5)	602,607 (526,465–726,663)	90.4 (79.0–109.0)	−2.1 (−2.6 to −1.6)	<0.001
15–19	59,018 (49,297–63,365)	11.4 (9.5–12.2)	41,922 (38,570–46,398)	6.7 (6.2–7.4)	−1.7 (−2.2 to −1.2)	<0.001	4,279,769 (3,574,714–4,594,996)	823.9 (688.2–884.6)	3,038,681 (2,795,691–3,363,401)	487.0 (448.0–539.0)	−1.7 (−2.2 to −1.2)	<0.001
20–24	79,527 (65,288–85,631)	16.2 (13.3–17.4)	63,237 (58,168–68,218)	10.6 (9.7–11.4)	−1.4 (−1.9 to −0.9)	<0.001	5,381,171 (4,417,570–5,794,279)	1,093.5 (897.7–1,177.5)	4,275,913 (3,932,944–4,612,818)	716.0 (658.6–772.5)	−1.4 (−1.9 to −1.0)	<0.001
**Socio-demographic index**												
Low	9,822 (8,095–11,810)	6.3 (5.2–7.6)	9,836 (9,152–10,689)	4.6 (4.0–5.6)	−1.1 (−1.7 to −0.5)	0.001	697,814 (573,781–838,623)	448.3 (368.6–538.8)	1,199,277 (1,037,337–1,451,320)	324.6 (280.8–392.9)	−1.1 (−1.8 to −0.5)	0.001
Low-middle	46,730 (35,258–52,796)	12.9 (9.7–14.6)	43,131 (37,967–47,490)	7.8 (6.9–8.6)	−1.7 (−2.5 to −0.8)	<0.001	3,294,248 (2,486,161–3,719,922)	910.7 (687.3–1,028.4)	3,024,640 (2,664,476–3,332,180)	547.2 (482.0–602.8)	−1.7 (−2.6 to −0.8)	<0.001
Middle	52,733 (39,123–57,813)	9.6 (7.1–10.5)	16,955 (14,665–20,532)	5.2 (4.8–5.7)	−2.0 (−2.2 to −1.8)	<0.001	3,709,295 (2,749,761–4,064,995)	675.9 (501.0–740.7)	2,024,017 (1,863,518–2,214,538)	366.2 (337.1–400.6)	−2.0 (−2.2 to −1.8)	<0.001
High-middle	24,835 (20,997–26,932)	8.8 (7.4–9.5)	28,914 (26,624–31,574)	4.4 (4.1–4.7)	−2.3 (−2.7 to −1.8)	<0.001	1,743,989 (1,474,683–1,890,831)	614.5 (519.6–666.3)	686,934 (639,445–746,229)	304.1 (283.1–330.4)	−2.3 (−2.7 to −1.9)	<0.001
High	16,282 (15,946–17,330)	8.3 (8.1–8.8)	14,040 (13,710–14,386)	7.6 (7.4–7.8)	−0.3 (−0.6 to 0.0)	0.061	1,135,599 (1,112,092–1,209,778)	579.8 (567.8–617.7)	978,218 (955,005–1,002,377)	527.1 (514.6–540.1)	−0.3 (−0.6 to 0.0)	0.064
**Region**												
Andean Latin America	559 (501–639)	4.5 (4.1–5.2)	924 (767–1,085)	5.4 (4.4–6.3)	0.4 (−0.1 to 1.0)	0.116	39,601 (35,471–45,215)	321.7 (288.1–367.3)	65,256 (54,202–76,651)	378.0 (314.0–444.0)	0.4 (−0.1 to 1.0)	0.133
Australasia	606 (582–628)	12.6 (12.1–13.1)	556 (523–586)	9.7 (9.1–10.2)	−0.9 (−1.7 to 0.0)	0.038	42,193 (40,514–43,795)	877.0 (842.1–910.3)	38,780 (36,472–40,879)	675.9 (635.7–712.5)	−0.9 (−1.7 to 0.0)	0.043
Caribbean	859 (792–919)	8.0 (7.4–8.6)	501 (407–605)	4.4 (3.6–5.3)	−2.0 (−2.5 to −1.5)	<0.001	60,055 (55,371–64,282)	562.4 (518.5–602.0)	35,029 (28,447–42,314)	309.2 (251.1–373.6)	−2.0 (−2.5 to −1.5)	<0.001
Central Asia	1,513 (1,442–1,585)	7.6 (7.3–8.0)	1,690 (1,517–1,865)	7.6 (6.9–8.4)	0.1 (−0.6 to 0.8)	0.819	107,184 (102,098–112,261)	540.5 (514.8–566.1)	119,210 (107,032–131,496)	538.7 (483.7–594.3)	0.1 (−0.6 to 0.7)	0.858
Central Europe	2,242 (2,173–2,315)	7.7 (7.4–7.9)	911 (841–960)	5.0 (4.6–5.3)	−1.4 (−1.8 to −1.0)	<0.001	157,349 (152,439–162,487)	538.9 (522.1–556.5)	63,419 (58,509–66,860)	349.7 (322.6–368.7)	−1.5 (−1.9 to −1.1)	<0.001
Central Latin America	2,229 (2,148–2,302)	4.1 (4.0–4.2)	4,502 (4,083–4,947)	6.9 (6.3–7.6)	1.7 (1.1–2.3)	<0.001	156,995 (151,322–162,139)	289.4 (278.9–298.8)	316,470 (286,904–348,152)	486.6 (441.2–535.3)	1.7 (1.1–2.2)	<0.001
Central sub-Saharan Africa	800 (622–1,045)	4.6 (3.6–6.0)	1,833 (1,326–2,619)	4.1 (2.9–5.8)	−0.5 (−0.7 to −0.2)	<0.001	57,113 (44,532–74,379)	330.0 (257.3–429.7)	129,851 (93,899–186,040)	288.9 (208.9–413.9)	−0.5 (−0.7 to −0.3)	<0.001
East Asia	42,844 (28,359–49,220)	11.5 (7.6–13.2)	7,035 (5,858–8,806)	2.9 (2.4–3.6)	−4.3 (−4.7 to −4.0)	<0.001	3,015,915 (1,999,466–3,456,810)	810.3 (537.2–928.8)	492,718 (410,913–615,659)	202.8 (169.1–253.3)	−4.4 (−4.7 to −4.0)	<0.001
Eastern Europe	5,952 (5,849–6,044)	12.6 (12.4–12.8)	3,188 (2,900–3,472)	9.7 (8.8–10.5)	−1.0 (−1.6 to −0.3)	0.003	417,450 (410,213–423,878)	883.9 (868.6–897.5)	222,348 (202,378–241,790)	673.9 (613.4–732.8)	−1.2 (−2.0 to −0.3)	0.006
Eastern sub-Saharan Africa	3,030 (2,454–3,519)	4.9 (4.0–5.7)	5,471 (4,614–6,808)	3.8 (3.2–4.7)	−0.8 (−0.9 to −0.8)	<0.001	216,835 (175,485–251,686)	349.5 (282.9–405.7)	387,768 (326,992–483,778)	266.6 (224.8–332.6)	−0.9 (−0.9 to −0.8)	<0.001
High-income Asia Pacific	3,011 (2,820–3,949)	7.1 (6.7–9.4)	2,462 (2,286–2,539)	9.4 (8.8–9.7)	1.2 (0.7–1.7)	<0.001	210,223 (196,837–276,157)	499.0 (467.2–655.5)	170,872 (158,573–176,209)	654.6 (607.4–675.0)	1.2 (0.7–1.7)	<0.001
High-income North America	6,095 (5,989–6,209)	10.0 (9.8–10.1)	7,358 (7,112–7,597)	10.3 (10.0–10.7)	0.1 (−0.1 to 0.4)	0.368	426,489 (419,218–434,484)	697.2 (685.3–710.3)	514,605 (497,267–531,181)	722.0 (697.6–745.2)	0.1 (−0.1 to 0.4)	0.372
North Africa and Middle East	4,890 (3,824–5,511)	4.5 (3.5–5.1)	4,871 (3,984–5,741)	3.0 (2.5–3.5)	−1.3 (−1.4 to −1.2)	<0.001	347,078 (271,091–391,349)	318.7 (248.9–359.3)	342,196 (279,667–403,241)	210.8 (172.3–248.5)	−1.3 (−1.4 to −1.2)	<0.001
Oceania	148 (122–177)	7.1 (5.8–8.5)	205 (167–284)	5.1 (4.1–7.1)	−1.0 (−1.4 to −0.7)	<0.001	10,417 (8,623–12,487)	498.0 (412.2–596.9)	14,390 (11,755–20,067)	356.9 (291.5–497.6)	−1.1 (−1.4 to −0.7)	<0.001
South Asia	56,583 (43,713–63,956)	16.9 (13.1–19.1)	52,159 (46,504–57,787)	9.9 (8.8–11.0)	−1.7 (−2.5 to −0.9)	<0.001	3,984,695 (3,075,988–4,506,382)	1,191.3 (919.7–1,347.3)	3,654,785 (3,261,969–4,057,141)	695.0 (620.3–771.5)	−1.8 (−2.7 to −0.8)	<0.001
Southeast Asia	7,574 (6,473–8,519)	5.1 (4.4–5.7)	5,283 (4,554–6,080)	3.1 (2.7–3.6)	−1.6 (−1.9 to −1.3)	<0.001	530,818 (453,794–597,125)	357.8 (305.9–402.5)	368,956 (318,574–424,421)	215.8 (186.3–248.2)	−1.6 (−1.9 to −1.3)	<0.001
Southern Latin America	1,050 (995–1,101)	7.9 (7.5–8.3)	1,504 (1,409–1,598)	9.8 (9.2–10.4)	0.6 (−0.2 to 1.4)	0.116	73,536 (69,687–77,096)	555.5 (526.5–582.4)	104,979 (98,460–111,570)	684.5 (642.0–727.5)	0.6 (−0.2 to 1.4)	0.126
Southern sub-Saharan Africa	1,404 (1,109–1,768)	8.2 (6.5–10.4)	2,283 (1,865–2,726)	10.5 (8.6–12.5)	0.8 (0.4–1.2)	<0.001	97,504 (77,091–122,666)	570.7 (451.3–718.0)	158,810 (129,898–189,669)	728.0 (595.5–869.5)	0.8 (0.4 to 1.2)	<0.001
Tropical Latin America	1,882 (1,803–1,966)	3.9 (3.8–4.1)	3,043 (2,920–3,167)	6.0 (5.8–6.3)	1.5 (0.9–2.0)	<0.001	131,606 (126,063–137,571)	275.0 (263.4–287.4)	212,430 (203,729–220,938)	420.0 (402.8–436.8)	1.5 (0.9–2.0)	<0.001
Western Europe	5,572 (5,454–5,689)	6.8 (6.6–6.9)	2,791 (2,671–2,893)	3.9 (3.7–4.0)	−1.9 (−2.1 to −1.7)	<0.001	386,219 (378,020–394,343)	469.9 (459.9–479.7)	193,806 (185,524–200,931)	268.9 (257.4–278.8)	−1.9 (−2.1 to −1.6)	<0.001
Western sub-Saharan Africa	1,654 (1,338–1,951)	2.8 (2.2–3.3)	4,365 (3,189–5,606)	2.7 (2.0–3.5)	−0.1 (−0.3 to 0.1)	0.417	118,332 (95,648–139,392)	197.7 (159.8–232.9)	310,522 (226,913–399,083)	192.4 (140.6–247.3)	−0.1 (−0.3 to 0.1)	0.285

#### Global trends by SDI

The mortality of suicide by SDI quintiles all showed a declining trend from 1990 to 2021 (Table 1, Figure 1). The largest downward mortality was observed in the high-middle SDI region (AAPC: −2.3 [−2.7 to −1.8]), which caused the lowest suicide mortality in 2021 (4.4 per 100,000 population [4.1–4.7]). Meanwhile, the low-middle SDI region had the highest suicide mortality (7.8 per 100,000 population [6.9–8.6]). The high SDI region had the lowest rate of decline (AAPC: −0.3 [−0.6 to 0]), where the suicide mortality (7.6 per 100,000 population [7.4–7.8]) was second only to the low-middle SDI region in 2021. In terms of the number of deaths by suicide, a significant increase in the high-middle SDI region occurred during the study period (death cases in 1990: 24,835 [20,997–26,932]; death cases in 2021: 28,914 [26,624–31,574]). Low-middle SDI region caused the most number of suicide deaths (43,131 [37,967–47,490]) and accounted for 3,024,640 (38.2%) YLLs in 2021.

#### Regional trends

The mortality of suicide decreased across most regions from 1990 to 2021, with a significant increase observed only in Central Latin America (AAPC: 1.7 [1.1–2.3]), Tropical Latin America (AAPC: 1.5 [0.9–2.0]), High-income Asia Pacific (AAPC: 1.2 [0.7–1.7]) and Southern sub-Saharan Africa (AAPC: 0.8 [0.4–1.2]). The largest decrease was observed in East Asia (AAPC: −4.3 [−4.7 to −4.0]) (Table 1). A similar pattern was observed for the AAPC of the rate of YLLs. Southern sub-Saharan Africa had the highest suicide mortality (10.5 per 100,000 population [8.6–12.5]), and Western sub-Saharan Africa had the lowest (2.7 [2.0–3.5]) in 2021. In terms of trends in suicide mortality, the most dramatic changes occurred in Eastern Europe, where two sharp increases were experienced before 2001 (APC in 1990–1994: 13.7 [10.7–16.9], APC in 1994–2001: 2.3 [0.5–4.1]), thus resulting in high-level suicide mortality (9.7 per 100,000 population [8.8–10.5]) in 2021. The region that caused the most suicide deaths was South Asia (52,159 [46,504–57,787]), which brought about 3,654,785 [3,261,969–4,057,141] YLLs and accounted for 49.8% of the total YLLs in 2021.

#### National trends

**Figure 2.**
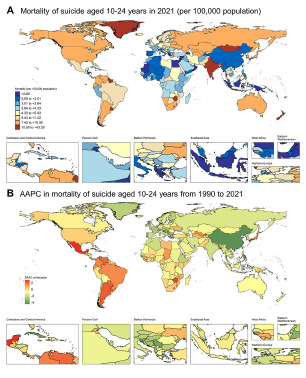
Global map of mortality of suicide in 2021 (A) and AAPC in mortality of suicide (B) in 10-24 years population from 1990 to 2021.

From 1990 to 2021, a total of 105 countries experienced a significant decrease in mortality and rate of YLLs. The fastest descent rate of suicide mortality was observed in China (AAPC: −4.5 [−4.8 to −4.1]), Cuba (AAPC: −3.9 [−5.6 to −2.1]), Luxembourg (AAPC: −3.9 [−6.2 to −1.5]), Serbia (AAPC: −3.9 [−4.7 to −3.0]) and Slovenia (AAPC: −3.8 [−4.5 to −3.1]) (Figure 2B, Supplementary Table S2). Besides, 29 countries experienced a significant increase in suicide mortality and the rate of YLLs. The most substantial increase in suicide mortality was observed in Mexico (AAPC: 2.8 [2.3–3.3]), Lesotho (AAPC: 2.7 [2.3–3.1]), Zimbabwe (AAPC: 2.6 [2.0–3.2]), Argentina (AAPC: 2.3 [1.4–3.1]) and Uruguay (AAPC: 2.2 [1.7–2.7]). In 2021, the country with the lowest suicide mortality was Jamaica (0.5 per 100,000 population [0.4–0.7]), and the highest was Greenland (43.3 per 100,000 population [33.3–51.3]). Alarmingly, half of the 30 countries with the highest global suicide mortality were from Oceania, even though Oceania did not have a high suicide mortality in 2021 (5.1 per 100,000 population [4.1–7.1]) (Figure 2A, Supplementary Table 1). Deaths from suicide in India (44,616 [38,126–49,874]), United States of America (6,697 [6,445–6,937]) and China (6,582 [5,419–8,340]), as the most populous countries, together constituted 57,895 of global 10–24 years population suicide death cases in 2021, which brought about 4,053,355 YLLs and accounted for 51.2% of total YLLs.

### Association with SDI

**Figure 3.**
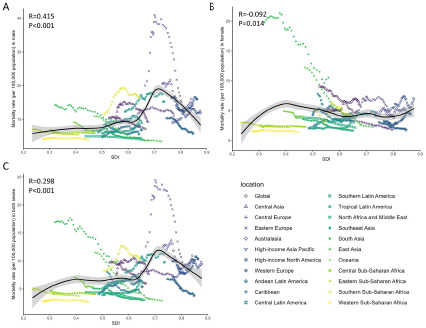
Trends for mortality of suicide among 21 regions by SDI for males (A), females (B), and both sexes (C) in 10-24 years population combined from 1990 to 2021 Black line represents the expected mortality based on SDI in all locations.

Figure 3 shows the observed versus the expected mortality attributable to suicide based on SDI at the regional level from 1990 to 2021. Generally, the patterns observed vary widely across sexes and regions. Suicide mortality showed a decreasing trend according to SDI in some regions, whereas others showed increasing rates or did not have monotonic associations with SDI (Figure 3C). In males, suicide mortality was positively correlated with SDI (Figure 3A). Among these regions, Southern sub-Saharan Africa and Eastern Europe had notably higher mortality than expected based on their SDIs from 1990 to 2021. In females, suicide mortality was inversely associated with SDI, and the observed mortality was higher than expected globally (Figure 3B). South Asia and Central Asia had higher observed mortality than expected rates. Supplementary Figure S17 shows the observed versus expected mortality at the national level based on SDI values in 2021, and a large variation between countries related to SDI was observed. Countries with low SDI remained low mortality rates, and when SDI increased to low-middle and high-middle levels, a much higher than expected suicide mortality rate was observed in certain countries (e.g. Zimbabwe, Greenland and Nauru). Meanwhile, there was no clear association between SDI and suicide mortality. A similar pattern was observed for the rate of YLLs associated with SDI (Supplementary Figure S16, S18).

### Age, period and cohort effects on suicide mortality

#### Age, period and cohort effects by SDI

**Figure 4.**
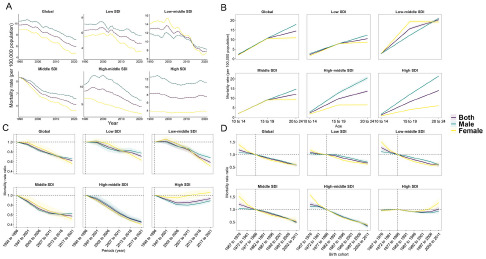
Age, period, and cohort effects on suicide mortality aged 10–24 years in global and 5 SDI regions (A) showed the trends in suicide mortality from 1990 to 2021. Age effects of mortality (B) are illustrated by the fitted longitudinal age-specific rates for a given number of birth cohorts adjusted for period deviations. Period effects of mortality (C) are illustrated by the period relative risk of mortality (mortality rate ratio) and calculated as the ratio of age-specific rates from 1992-1996 period to 2017-2021 period, with the reference period set at 1992-1996. Birth cohort effects of mortality (D) are illustrated by the cohort relative risk of mortality (mortality rate ratio) and calculated as the ratio of age-specific rates from 1967-1976 cohort to 2002-2011 cohort, with the reference cohort set at 1977-1986. The shaded areas indicate the corresponding 95% CIs of each point estimate.

Figure 4 demonstrates the age, period and cohort effects of mortality derived from the age, period and cohort model in global and all SDI regions. Generally, the mortality of suicide declined across all SDI regions (Figure 4A). Males in low, high-middle and high SDI regions had higher suicide mortality than females and were consistent with the global trend. In the low-middle SDI region, mortality in females declined significantly faster than in males, thus in 2014, the mortality in females started to lag males and remained at a lower rate till 2021. In the middle SDI region, suicide mortality for males and females was initially roughly equal in 1990, but as time continued, mortality in females became progressively lower than in males, and the gap continued to widen till 2021. All SDI regions reveal similar age effects that the risk of suicide mortality increased with increasing age (Figure 4B). Males showed a significantly higher risk of age effect compared with females, but in the low-middle SDI region was the opposite. Period effects presented a downward risk of suicide mortality across different SDI quintiles during the study period (Figure 4C). In the high SDI region, the risk of females remained nearly constant over the past three decades (period effect in 2017–2021: 1.06 [1.00–1.12]), indicating little improvement in suicide mortality across the study period. Regarding cohort effects, a continuous decline risk of mortality was observed in most SDI regions, except in high SDI region (Figure 4D). Notably, in the high SDI region, the cohort risk did not change significantly for males (cohort effect in 2002–2011: 0.9 [0.8–1.0]) but increased slightly for females (cohort effect in 2002–2011: 1.3 [1.1–1.5]). The suicide mortality trend from 1990 to 2021, and its age, period and birth cohort effects for each region and country were shown in Supplementary Tables S19–S42.

#### Age, period and cohort effects in exemplary countries

**Figure 5.**
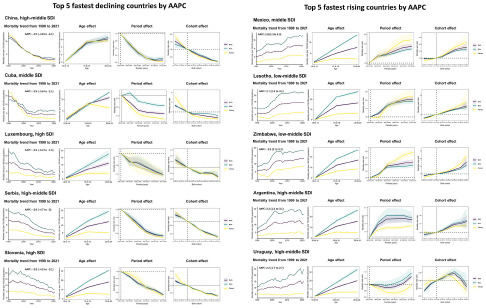
Age, period, and cohort effects on exemplary countries by the five fastest declining and rising AAPC values Mortality trends shows trends of mortality in both sexes, males, and females from 1990 to 2021. Age effects of mortality are illustrated by the fitted longitudinal age-specific rates for a given number of birth cohorts adjusted for period deviations. Period effects of mortality are illustrated by the period relative risk of mortality (mortality rate ratio) and calculated as the ratio of age-specific rates from 1992-1996 period to 2017-2021 period, with the reference period set at 1992-1996. Birth cohort effects of mortality are illustrated by the cohort relative risk of mortality (mortality rate ratio) and calculated as the ratio of age-specific rates from 1967-1976 cohort to 2002-2011 cohort, with the reference cohort set at 1977-1986. The shaded areas indicate the corresponding 95% CIs of each point estimate.

Figure 5 presents the age, period and birth cohort effects based on AAPC values for the five fastest-declining and five fastest-rising countries. Generally, age effects showed the same pattern in the vast majority of countries: the risk of suicide mortality increased with age, and the risk for males was higher than for females, except for China listed in the figure. Specifically, no significant difference was observed in age effect between males and females in China. The five countries with the fastest declines in AAPC showed trends in period and cohort effects remained nearly identical, with both declining extremely rapidly and continuously. Notably, in China, the risk of period effect in females declined faster than in males, which was different from the other countries.

Among the five countries with the fastest rising AAPC, all countries fluctuated upward with increasing years. Besides, suicide mortality rose significantly faster for males than for females, leading to a widening of the sex gap in suicide mortality as time going. The upward trends in period and cohort effects were slightly different in these five countries. Specifically, both period and cohort effects rose continuously in Mexiko and Letoso. The risk of period effect in Zimbabwe for females rose significantly in the 2002–2021 timeframe, but the risk for males remained stable at the same time. Both Argentina and Uruguay, as high-middle SDI region countries, had hugely different trends in period and cohort effects. In Argentina, the period effect rose first, then fell, like an inverted ‘V’ shape, while the effect in Uruguay showed a positive ‘V’ structure. The cohort effect in Argentina increased continuously from 1967 to 2011, while in Uruguay, it showed a rising and then falling trend.

## Discussion

To our knowledge, this is the first study to describe the burden and trends of suicide mortality and YLLs among adolescents and young adults aged 10–24 years, from 1990 to 2021, at global, regional and national levels. Although the suicide mortality and rate of YLLs decreased significantly globally across sexes and age groups, obvious variability was observed across regions and countries. Low-middle SDI region had the highest burden of suicide mortality and death cases in 2021. In the high SDI region, a more unfavourable mortality risk was shown in period and cohort effect, especially in females. Regionally, the suicide mortality trends in Central Latin America, Tropical Latin America, High-income Asia Pacific and Southern sub-Saharan Africa increased fast. Countries with extreme anomalies in suicide mortality and the rate of YLLs were mostly concentrated in low-middle, middle and high-middle SDI regions. A total of 29 countries had a significant upward trend in suicide mortality and the rate of YLLs over the past three decades.

In the nearly three decades since 1990, there has been a significant downward trend in suicide mortality and the rate of YLLs among adolescents and young adults globally. Global suicide mortality showed the most rapid decline in 2012–2015. This may be due to the effective support and guidance provided by WHO and the United Nations for the implementation of national suicide prevention initiatives. In 1996, the WHO issued national strategies and guidelines for suicide prevention (UN. Department for Policy Coordination and Sustainable Development, 1996). In 2013, WHO promulgated the Comprehensive Mental Health Action Plan 2013–2020 (Saxena *et al.*, 2013), which set a goal of reducing global suicide mortality by 10% between 2012 and 2020. Subsequently, in 2014, WHO released its first World Suicide Report (Fleischmann and De Leo, 2014). These initiatives contributed to a positive worldwide response to suicide as a serious public health problem. Additionally, we observed no significant increase in global suicide mortality occurred between 2019 and 2021, implying that COVID-19 did not seem to have an impact on suicide risk in adolescents and young adults worldwide. However, given the short observation period since the pandemic, this may require more time to observe the potential impact of the COVID-19 pandemic on suicide among adolescents and young adults.

There was a huge disparity in the mortality of suicide in males and females. Although the burden of suicide mortality in females fell nearly twice as fast as in males, males had higher mortality and accounted for the majority of death cases in 2021. This finding is broadly consistent with previous research (Naghavi, 2019), which may involve a ‘sex paradox’ (Brokke *et al.*, 2022). Despite the prevalence of suicidal ideation and planning is higher in females (Nock *et al.*, 2008; Voss *et al.*, 2019), males tend to be more impulsive and aggressive, choosing more lethal methods of suicide, such as using a gun or jumping from a building (Brokke *et al.*, 2022; Nock and Kessler, 2006), which results in a higher suicide mortality in males. However, this is not applicable in all regions. For example, South Asia is the only region where suicide mortality for females was consistently higher than for males regionally, perhaps because of the pessimistic sex inequality in females (e.g. child marriage, malnutrition, low levels of education) (Fan and Koski, 2022; Levy *et al.*, 2020; World Economic Forum, 2023). Therefore, sex-specific interventions and management of suicide are needed in different regions.

We found the mortality of suicide rose with the increasing of age. The lowest decline in mortality was observed in 20–24-year-old young adults, and about half of the death cases in the 10–24-year-old population were concentrated in this age group. We generally agree that the incidence of suicide increases with time during adolescence, peaks in late adolescence, and finally stabilizes in early adulthood (Boričević Maršanić *et al.*, 2022; Hawton *et al.*, 2012). Given the increase in drug and alcohol abuse among young people, and their greater vulnerability to social deprivation and stressful life events (Bridge *et al.*, 2006; Kim *et al.*, 2019), as well as the fact that intervention policies tend to focus on the underage population (Feiss *et al.*, 2019), it is not surprising that the suicide mortality was higher in 20–24 years age group. Therefore, more attention should be paid to the risk of suicide as young people grow in age.

The characteristics of the suicide mortality burden among adolescents and young adults varied by different SDI quintiles. Despite the overall decline of mortality in low SDI region, the majority of low SDI countries come from Africa, where mental health remains vulnerable in certain countries due to insufficient investment in mental health services (World Health Organization, 2022). Over the past three decades, suicide mortality has remained at the highest levels and resulted in the greatest number of death cases in low-middle SDI region. High suicide mortality may be related to socio-economic status and income, with lower socio-economic status increasing the likelihood that individuals will commit and attempt suicide (Knipe *et al.*, 2015). Furthermore, the prevalence of communicable diseases and poor social health services are also important causes. Middle and high-middle SDI regions had the fastest decline rate in suicide mortality. Interestingly, some countries with the fastest rising mortality rate (Mexico, Argentina and Uruguay) or extremely unusual suicide mortality (Nepal, Guyana, e.g.) also came from middle and high-middle SDI region. This suggests the changes in national suicide mortality in middle and high-middle SDI regions show a multipolar trend. Moreover, there was a significant increase in suicide death cases among the 10–24-year-old population in the high-middle SDI region, which may be related to the population growth (United Nations, 2024) and the increase in the number of countries in the high-middle SDI region (GBD 2021 Diseases and Injuries Collaborators, 2024). The suicide mortality in the high SDI region was second only to the low-middle SDI region and had the slowest reduction of mortality rate. One possible explanation is that being more developed and individualistic societies, economically developed regions may be more competitive and stressful (Barbalat and Franck, 2020; Dückers *et al.*, 2019). Mental disorders and self-harming behaviours are significant contributors to the disease burden among young people in high-income countries (GBD 2017 Child and Adolescent Health Collaborators, 2017; Mokdad *et al.*, 2016). Besides, people in high-income regions with mental and behavioural disability issues may be less stigmatized, thus increasing rates of detection and reporting of suicide (Dückers and Brewin, 2016). We also found the age and period effect in female suicide mortality exhibited unfavourable trends in high SDI region, which is correlated with the increase of female suicide mortality in some countries such as the United States of America, Australia and Canada. Another reason is that in the high SDI region, the burden of disease for anxiety and depression grows faster for females (Li *et al.*, 2022; Xiong *et al.*, 2022), thus increasing the likelihood of suicide in females.

Regionally, Central Latin America, Tropical Latin America, High-income Asia Pacific and Southern sub-Saharan Africa had a rapid upward suicide mortality trend. In Latin America, low socio-economic status and natural disasters had an impact on the mental health of children and adolescents (Duarte *et al.*, 2003). Mental health treatment gaps are also a significant problem, and the treatment gap for severe mental disorders in children and adolescents is more than 50% (Kohn *et al.*, 2018). In Southern sub-Saharan Africa, bad weather, social stigma and religious culture are the important reasons for high suicide mortality (Jidong *et al.*, 2024). Economic uncertainty and higher academic pressure (Goto *et al.*, 2024; Lee *et al.*, 2020) contributed to the rising suicide mortality among young people in High-income Asia Pacific. East Asia had the fastest declining suicide mortality, which could be attributed to the favourable decline in suicide trends in China. Fluctuating changes in suicide mortality in Eastern Europe may be due to the privatization of communism and the economic crisis (Kõlves *et al.*, 2013; Mäkinen, 2006). Furthermore, despite the decline in suicide mortality in South Asia is not negligible, the burden of suicide from his large population remains heavy. More mental health services should be implemented to continue to reduce the number of suicides and improve the well-being of young people in these regions.

Nationally, suicide mortality increased in 29 countries and decreased in 105 countries significantly from 1990 to 2021. The five countries with the fastest declines in suicide mortality have all experienced improved economic conditions, upgraded health services, and positive cultural and social evolution (Corona-Miranda *et al.*, 2018; Ilic and Ilic, 2022; Zhang *et al.*, 2014). In contrast to the five fastest declining countries, poor economic climate, political turmoil and raging AIDS epidemic increased the burden of mental disorders and risk of suicide in Lesotho and Zimbabwe (Kabir *et al.*, 2023; Kidia *et al.*, 2017). The poor suicide mortality trend in Mexico may be due to the unequal distribution of mental health resources and the slow growth of community services (Carmona-Huerta *et al.*, 2021). Political instability and economic difficulties are important reasons for the increase in suicide mortality in some Latin American countries (Argentina, Uruguay, e.g.) (Dávila-Cervantes, 2022). Taken as a whole, these patterns reflected the complex interplay of country-specific factors on suicide. Despite strong global efforts to reduce suicide mortality globally, the mortality gap between countries remained large in 2021, which ranged from 0.51 per 100,000 population in Jamaica to 43.29 per 100,000 population in Greenland. Most strikingly, half of the 30 countries with the highest global suicide mortality were from Oceania, which may be related to substance abuse among local young people (Peltzer and Pengpid, 2015). Therefore, there are complex social, economic, cultural and individual mental health issues behind the rising suicide mortality among adolescents and young adults. Suicide prevention needs to take these factors into account by strengthening measures such as mental health education, provision of effective mental health services, alcohol/substance management and improvement of the economic situation, to reduce the rate of suicide among young people and to enhance the overall level of mental health of the society.

The present study still has certain limitations that need to be acknowledged. Firstly, raw data on suicide mortality are lacking in parts of the world, so mortality estimates for these countries are composed of modelling results from specific suicide models derived from data from other countries, this uncertainty may affect the accuracy of estimates of age, period and birth cohort effects. Furthermore, suicide is often under-reported, or miscoded for reasons of stigma, and although GBD has adjusted for this miscoding, the low suicide rate should be interpreted with caution. Additionally, we did not conduct further analysis of the subtypes of suicide, in GBD, the method of suicide is further divided into ‘firearm’ and ‘by other specified means’, detailed analysis of the subclassification data can better understand the prevalence characteristics of suicide methods in different regions, which should be further investigated in subsequent studies.

## Conclusion

This study provides comprehensive estimates of suicide mortality and YLLs among adolescents and young adults by age, sex, SDI quintiles, region and country, and explores the age, period and cohort effects of suicide mortality from 1990 to 2021. The study found suicide mortality and rate of YLLs among adolescents and young adults decreased significantly globally in the past three decades, but trends were not consistent across sexes, regions, and countries. Therefore, an urgent need is required to scale up mental health services and to designate region-specific and sex-specific policies to screen for suicidal behaviour among adolescents and young adults. More importantly, the study also gives new insights into suicide trends after the COVID-19 pandemic. Despite the pandemic did not impact suicidal behaviour among young people in this short time, the long-term effect cannot be ignored in the future.

## Supporting information

Yan et al. supplementary material Yan et al. supplementary material

## Data Availability

The data presented in this study are available on the IHME website (https://vizhub.healthdata.org/gbd-results/).

## Supplementary material

The supplementary material for this article can be found at https://doi.org/10.1017/S2045796024000532.

## References

[ref1] Barbalat G and Franck N (2020) Ecological study of the association between mental illness with human development, income inequalities and unemployment across OECD countries. *BMJ Open* 10(4), e035055. 10.1136/bmjopen-2019-035055 PMC7204933 32317261

[ref2] Bell A (2020) Age period cohort analysis: A review of what we should and shouldn’t do. *Annals of Human Biology* 47(2), 208–217. 32429768 10.1080/03014460.2019.1707872

[ref3] Boričević Maršanić V, Silobrčić Radić M and Flander Tadić M (2022) Trends in adolescent completed suicide in Croatia for the period of 2000 to 2020. *Psychiatria Danubina* 34(4), 715–718. 36548886 10.24869/psyd.2022.715

[ref4] Bridge JA, Goldstein TR and Brent DA (2006) Adolescent suicide and suicidal behavior. *Journal of Child Psychology and Psychiatry* 47(3-4), 372–394. 16492264 10.1111/j.1469-7610.2006.01615.x

[ref5] Brokke SS, Landrø NI and Haaland V (2022) Impulsivity and aggression in suicide ideators and suicide attempters of high and low lethality. *BMC Psychiatry* 22(1), 753. 10.1186/s12888-022-04398-w PMC9714086 36457001

[ref6] Carmona-Huerta J, Durand-Arias S, Rodriguez A, Guarner-Catalá C, Cardona-Muller D, Madrigal-de-león E and Alvarado R (2021) Community mental health care in Mexico: A regional perspective from a mid-income country. *International Journal of Mental Health Systems* 15(1), 7. 10.1186/s13033-020-00429-9 PMC7802166 33430918

[ref7] Centers for Disease Control and Prevention (2023) Suicide and homicide death rates among youth and young adults aged 10–24: United States, 2001–2021. Atlanta. https://www.cdc.gov/nchs/products/databriefs/db471.htm (accessed 15 June 2023).

[ref8] Corona-Miranda B, Alfonso-Sagué K, Hernández-Sánchez M and Cortés-Alfaro A (2018) Attempted and completed suicide in Cuban adolescents, 2011-2014. *MEDICC Review* 20(1), 36. 10.37757/MR2018.V20.N1.8 34229420

[ref9] Dávila-Cervantes CA (2022) Suicide burden in Latin America, 1990-2019: Findings from the Global Burden of Disease Study 2019. *Public Health* 205, 28–36. 35219840 10.1016/j.puhe.2022.01.014

[ref10] Duarte C, Hoven C, Berganza C, Bordin I, Bird H and Miranda CT (2003) Child mental health in Latin America: Present and future epidemiologic research. *International Journal of Psychiatry in Medicine* 33(3), 203–222. 15089004 10.2190/4WJB-BW16-2TGE-565W

[ref11] Dückers MLA, Reifels L, De Beurs DP and Brewin CR (2019) The vulnerability paradox in global mental health and its applicability to suicide. *British Journal of Psychiatry* 20, 1–6. 10.1192/bjp.2019.41 30890196

[ref12] Dückers ML and Brewin CR (2016) A paradox in individual versus national mental health vulnerability: Are higher resource levels associated with higher disorder prevalence? *Journal of Traumatic Stress* 29(6), 572–576. 27859656 10.1002/jts.22144

[ref13] Fan S and Koski A (2022) The health consequences of child marriage: A systematic review of the evidence. *BMC Public Health* 22(1), 309. 10.1186/s12889-022-12707-x PMC8845223 35164724

[ref14] Feiss R, Dolinger SB, Merritt M, Reiche E, Martin K, Yanes JA, Thomas CM and Pangelinan M (2019) A systematic review and meta-analysis of school-based stress, anxiety, and depression prevention programs for adolescents. *Journal of Youth and Adolescence* 48(9), 1668–1685. 31346924 10.1007/s10964-019-01085-0 PMC7548227

[ref15] Fleischmann A and De Leo D (2014) The World Health Organization’s report on suicide: A fundamental step in worldwide suicide prevention. *Crisis: The Journal of Crisis Intervention and Suicide Prevention* 35(5), 289–291. 10.1027/0227-5910/a000293 25297514

[ref16] GBD 2016 Causes of Death Collaborators (2017) Global, regional, and national age-sex specific mortality for 264 causes of death, 1980-2016: A systematic analysis for the Global Burden of Disease Study 2016. *Lancet* 390(10100), 1151–1210. 28919116 10.1016/S0140-6736(17)32152-9 PMC5605883

[ref17] GBD 2017 Child and Adolescent Health Collaborators (2017) Child and adolescent health from 1990 to 2015: Findings from the Global Burden of Diseases, Injuries, and Risk Factors 2015 Study. *JAMA Pediatrics* 171(6), 573–592. 28384795 10.1001/jamapediatrics.2017.0250 PMC5540012

[ref18] GBD 2019 Diseases and Injuries Collaborators (2020) Global burden of 369 diseases and injuries in 204 countries and territories, 1990–2019: A systematic analysis for the Global Burden of Disease Study 2019. *The Lancet* 396(10258), 1204–1222. 10.1016/S0140-6736(20)30925-9 PMC7567026 33069326

[ref19] GBD 2021 Diseases and Injuries Collaborators (2024) Global incidence, prevalence, years lived with disability (YLDs), disability-adjusted life-years (DALYs), and healthy life expectancy (HALE) for 371 diseases and injuries in 204 countries and territories and 811 subnational locations, 1990-2021: A systematic analysis for the Global Burden of Disease Study 2021. *Lancet* 403(10440), 2133–2161. 38642570 10.1016/S0140-6736(24)00757-8 PMC11122111

[ref20] Goto H, Kawachi I and Vandoros S (2024) The association between economic uncertainty and suicide in Japan by age, sex, employment status, and population density: An observational study. *The Lancet Regional Health Western Pacific* 46, 101069. 10.1016/j.lanwpc.2024.101069 PMC11070334 38711964

[ref21] Hawton K, Saunders KE and O’Connor RC (2012) Self-harm and suicide in adolescents. *Lancet* 379(9834), 2373–2382. 22726518 10.1016/S0140-6736(12)60322-5

[ref22] Ilic M and Ilic I (2022) Trends in suicide by hanging, strangulation, and suffocation in Serbia, 1991-2020: A joinpoint regression and age-period-cohort analysis. *World Journal of Psychiatry* 12(3), 505–520. 35433320 10.5498/wjp.v12.i3.505 PMC8968500

[ref23] India State-Level Disease Burden Initiative Suicide Collaborators (2018) Gender differentials and state variations in suicide deaths in India: The Global Burden of Disease Study 1990-2016. *Lancet Public Health* 3(10), e478–e489. 30219340 10.1016/S2468-2667(18)30138-5 PMC6178873

[ref24] Jidong DE, Ike TJ, Murshed M, Nyam PP, Husain N, Jidong JE, Pwajok JY, Francis C, Mwankon SB and Okoli E (2024) Interventions for self-harm and suicidal ideation in Africa: A systematic review. *Archives of Suicide Research* 20, 1–25. 10.1080/13811118.2024.2316168 38506246

[ref25] Jin X, Ren J, Li R, Gao Y, Zhang H, Li J, Zhang J, Wang X and Wang G (2021) Global burden of upper respiratory infections in 204 countries and territories, from 1990 to 2019. *EClinical Medicine* 37, 100986. 10.1016/j.eclinm.2021.100986 PMC8343248 34386754

[ref26] Kabir H, Wayland S and Maple M (2023) Qualitative research in suicidology: A systematic review of the literature of low-and middle-income countries. *BMC Public Health* 23(1), 918. 10.1186/s12889-023-15767-9 PMC10199541 37208634

[ref27] Kidia K, Machando D, Mangezi W, Hendler R, Crooks M, Abas M, Chibanda D, Thornicroft G, Semrau M and Jack H (2017) Mental health in Zimbabwe: A health systems analysis. *The Lancet Psychiatry* 4(11), 876–886. 28625876 10.1016/S2215-0366(17)30128-1

[ref28] Kim GM, Kim J, Hyun MK, Choi S and Woo JM (2019) Comparison of the risk factors of Korean adolescent suicide residing in high suicidal regions versus those in low suicidal regions. *Psychiatria Danubina* 31(4), 397–404. 31698395 10.24869/psyd.2019.397

[ref29] Kim S, Park J, Lee H, Lee H, Woo S, Kwon R, Kim S, Koyanagi A, Smith L, Rahmati M, Fond G, Boyer L, Kang J, Lee JH, Oh J and Yon DK (2024) Global public concern of childhood and adolescence suicide: A new perspective and new strategies for suicide prevention in the post-pandemic era. *World Journal of Pediatrics* 20 (9), 872–900. 39008157 10.1007/s12519-024-00828-9

[ref30] Knipe DW, Carroll R, Thomas KH, Pease A, Gunnell D and Metcalfe C (2015) Association of socio-economic position and suicide/attempted suicide in low and middle income countries in South and South-East Asia – A systematic review. *BMC Public Health* 15, 1055. 10.1186/s12889-015-2301-5 PMC4608117 26472204

[ref31] Kohn R, Ali AA, Puac-Polanco V, Figueroa C, López-Soto V, Morgan K, Saldivia S and Vicente B (2018) Mental health in the Americas: An overview of the treatment gap. *Revista Panamericana de Salud Publica-Pan American Journal of Public Health* 42, e165. 10.26633/RPSP.2018.165 PMC6386160 31093193

[ref32] Kõlves K and de Leo D (2017) Suicide methods in children and adolescents. *European Child and Adolescent Psychiatry* 26(2), 155–164. 27194156 10.1007/s00787-016-0865-y

[ref33] Kõlves K, Milner A and Värnik P (2013) Suicide rates and socioeconomic factors in Eastern European countries after the collapse of the Soviet Union: Trends between 1990 and 2008. *Sociology of Health and Illness* 35(6), 956–970. 23398609 10.1111/1467-9566.12011

[ref34] Lee D, Jung S, Park S, Lee K, Kweon YS, Lee EJ, Yoon KH, Cho H, Jung H, Kim AR, Shin BR and Hong HJ (2020) Youth suicide in Korea across the educational stages. *Crisis* 41(3), 187–195. 31512944 10.1027/0227-5910/a000624

[ref35] Levy JK, Darmstadt GL, Ashby C, Quandt M, Halsey E, Nagar A and Greene ME (2020) Characteristics of successful programmes targeting gender inequality and restrictive gender norms for the health and wellbeing of children, adolescents, and young adults: A systematic review. *The Lancet Global Health* 8(2), e225–e236. 31879212 10.1016/S2214-109X(19)30495-4 PMC7025324

[ref36] Li S, Xu Y, Zheng L, Pang H, Zhang Q, Lou L and Huang X (2022) Sex difference in global burden of major depressive disorder: Findings from the Global Burden of Disease Study 2019. *Frontiers in Psychiatry* 13, 789305. 10.3389/fpsyt.2022.789305 PMC8898927 35264985

[ref37] Liu L, Villavicencio F, Yeung D, Perin J, Lopez G, Strong KL and Black RE (2022) National, regional, and global causes of mortality in 5-19-year-olds from 2000 to 2019: A systematic analysis. *The Lancet Global Health* 10(3), e337–e347. 35180417 10.1016/S2214-109X(21)00566-0 PMC8864304

[ref38] Mäkinen IH (2006) Suicide mortality of Eastern European regions before and after the Communist period. *Social Science & Medicine* 63(2), 307–319. 16473447 10.1016/j.socscimed.2006.01.002

[ref39] Mokdad AH, Forouzanfar MH, Daoud F, Mokdad AA, El Bcheraoui C, Moradi-Lakeh M, Kyu HH, Barber RM, Wagner J, Cercy K, Kravitz H, Coggeshall M, Chew A, O’Rourke KF, Steiner C, Tuffaha M, Charara R, Al-Ghamdi EA, Adi Y, Afifi RA, Alahmadi H, AlBuhairan F, Allen N, AlMazroa M, Al-Nehmi AA, AlRayess Z, Arora M, Azzopardi P, Barroso C, Basulaiman M, Bhutta ZA, Bonell C, Breinbauer C, Degenhardt L, Denno D, Fang J, Fatusi A, Feigl AB, Kakuma R, Karam N, Kennedy E, Khoja TA, Maalouf F, Obermeyer CM, Mattoo A, McGovern T, Memish ZA, Mensah GA, Patel V, Petroni S, Reavley N, Zertuche DR, Saeedi M, Santelli J, Sawyer SM, Ssewamala F, Taiwo K, Tantawy M, Viner RM, Waldfogel J, Zuñiga MP, Naghavi M, Wang H, Vos T, Lopez AD, Al Rabeeah AA, Patton GC and Murray CJ (2016) Global burden of diseases, injuries, and risk factors for young people’s health during 1990-2013: A systematic analysis for the Global Burden of Disease Study 2013. *Lancet* 387(10036), 2383–2401. 27174305 10.1016/S0140-6736(16)00648-6

[ref40] Naghavi M (2019) Global, regional, and national burden of suicide mortality 1990 to 2016: Systematic analysis for the Global Burden of Disease Study 2016. *BMJ* 364, l94. 10.1136/bmj.l94 PMC6598639 31339847

[ref41] National Cancer Institute (2024) Joinpoint Trend Analysis Software. Bethesda. https://surveillance.cancer.gov/joinpoint/ (accessed 25 May 2023).

[ref42] Nock MK, Borges G, Bromet EJ, Cha CB, Kessler RC and Lee S (2008) Suicide and suicidal behavior. *Epidemiologic Reviews*, 30(1), 133–154. 18653727 10.1093/epirev/mxn002 PMC2576496

[ref43] Nock MK and Kessler RC (2006) Prevalence of and risk factors for suicide attempts versus suicide gestures: Analysis of the National Comorbidity Survey. *Journal of Abnormal Psychology* 115(3), 616–623. 16866602 10.1037/0021-843X.115.3.616

[ref44] Peltzer K and Pengpid S (2015) Early substance use initiation and suicide ideation and attempts among school-aged adolescents in four Pacific Island countries in Oceania. *International Journal of Environmental Research & Public Health* 12(10), 12291–12,303. 10.3390/ijerph121012291 PMC4626969 26437423

[ref45] Rosenberg PS, Check DP and Anderson WF (2014) A web tool for age-period-cohort analysis of cancer incidence and mortality rates. *Cancer Epidemiology Biomarkers and Prevention* 23(11), 2296–2302. 10.1158/1055-9965.EPI-14-0300 PMC4221491 25146089

[ref46] Saxena S, Funk M and Chisholm D (2013) World Health Assembly adopts Comprehensive Mental Health Action Plan 2013-2020. *Lancet* 381(9882), 1970–1971. 23746771 10.1016/S0140-6736(13)61139-3

[ref47] Townsend E (2014) Self-harm in young people. *Evidence-Based Mental Health* 17(4), 97–99. 25114299 10.1136/eb-2014-101840 PMC13404014

[ref48] Turecki G and Brent DA (2016) Suicide and suicidal behaviour. *Lancet* 387(10024), 1227–1239. 26385066 10.1016/S0140-6736(15)00234-2 PMC5319859

[ref49] UN. Department for Policy Coordination and Sustainable Development (1996) Prevention of suicide: Guidelines for the formulation and implementation of national strategies. New York. https://digitallibrary.un.org/record/215713 (accessed 12 July 2024).

[ref50] United Nations (2019) Globally, 20% of adolescents suffer from mental illness and 15% of adolescents in low- and middle-income countries have had suicidal thoughts. New York. https://news.un.org/zh/story/2019/11/1045021 (accessed 5 November 2019).

[ref51] United Nations (2024) World Population Prospects 2024. Washington. https://population.un.org/wpp/Graphs/ (accessed 20 May 2024).

[ref52] Voss C, Ollmann TM, Miché M, Venz J, Hoyer J, Pieper L, Höfler M and Beesdo-Baum K (2019) Prevalence, onset, and course of suicidal behavior among adolescents and young adults in Germany. *JAMA Network Open* 2(10), e1914,386. 10.1001/jamanetworkopen.2019.14386 PMC6824228 31664450

[ref53] World Economic Forum (2023) Global Gender Gap Report 2023. Beijing. https://www.weforum.org/publications/global-gender-gap-report-2023/in-full/ (accessed 20 June 2023).

[ref54] World Health Organization (2022) WHO calls for attention to suicide in Africa and greater investment in mental health. Geneva. https://news.un.org/zh/story/2022/10/1111147 (accessed 6 October 2022).

[ref55] World Health Organization (2023) Suicide. Geneva. https://www.who.int/news-room/fact-sheets/detail/suicide (accessed 28 August 2023).

[ref56] Xiong P, Liu M, Liu B and Hall BJ (2022) Trends in the incidence and DALYs of anxiety disorders at the global, regional, and national levels: Estimates from the Global Burden of Disease Study 2019. *Journal of Affective Disorders* 297, 83–93. 34678404 10.1016/j.jad.2021.10.022

[ref57] Yang J, Zhang Y, Luo L, Meng R and Yu C (2018) Global mortality burden of cirrhosis and liver cancer attributable to injection drug use, 1990-2016: An age-period-cohort and spatial autocorrelation analysis. *International Journal of Environmental Research & Public Health* 15(1), 170. 10.3390/ijerph15010170 PMC5800269 29361804

[ref58] Yip PSF, Zheng Y and Wong C (2022) Demographic and epidemiological decomposition analysis of global changes in suicide rates and numbers over the period 1990-2019. *Injury Prevention* 28(2), 117–124. 34400542 10.1136/injuryprev-2021-044263

[ref59] Zhang J, Sun L, Liu Y and Zhang J (2014) The change in suicide rates between 2002 and 2011 in China. *Suicide and Life-Threatening Behavior* 44(5), 560–568. 24690079 10.1111/sltb.12090

