# Supplementary material for: Global patterns and trends of suicide mortality and years of life lost among adolescents and young adults from 1990 to 2021: a systematic analysis for the Global Burden of Disease Study 2021
Source: Epidemiol Psychiatr Sci. 2024 Oct 21;33:e52. doi: 10.1017/S2045796024000532 (PMC11561676; doi:10.1017/S2045796024000532)
Supplement: Yan et al. supplementary material [file S2045796024000532sup001.pdf]

Content

Method S1. GBD 2021 methods for suicide mortality estimation 3
Method S2. Age, period, and cohort analysis of suicide mortality... 6
Table S1. The mortality and death cases from suicide among whole population between WHO estimates in 2021 and GBD estimates in 2021 8
Table S2. The rate of suicide mortality and their AAPC from 1990 to 2021 in 204 regions..... 9
Table S3. The rate of YLLs of suicide and their AAPC from 1990 to 2021 in 204 regions.... 20
Table S4. The trends in rate of suicide mortality in 204 countries by Joinpoint regression.... 31
Table S5. The trends in rate of suicide YLLs in 204 countries by Joinpoint regression 54
Table S6. Wald χ^2 test of suicide mortality in age, period, and cohort models in global and 5 SDI regions.. 76
Figure S1. Joinpoint regression analysis of mortality from suicide in different sexes from 1990 to 2021 79
Figure S2. Joinpoint regression analysis of rate of YLLs from suicide in different sexes from 1990 to 2021 80
Figure S3. Joinpoint regression analysis of mortality from suicide in different age groups from 1990 to 2021 81
Figure S4. Joinpoint regression analysis of rate of YLLs from suicide in different age groups from 1990 to 2021 82
Figure S5. Joinpoint regression analysis of rate of suicide YLLs aged 10–24 years in global and 5 SDI regions from 1990 to 2021 83
Figure S6. Joinpoint regression analysis of mortality from suicide in 21 regions from 1990 to 2021 84
Figure S7. Joinpoint regression analysis of rate of YLLs from suicide in 21 regions from 1990 to 2021 85
Figure S8. Joinpoint regression analysis of mortality from suicide among males and females in global and different regions, 1990-2021.. 86
Figure S9. Joinpoint regression analysis of rate of YLLs from suicide among males and females in global and different regions, 1990-2021 86
Figure S10. Joinpoint regression analysis of mortality from suicide among different age groups in global and different regions, 1990-2021 87
Figure S11. Joinpoint regression analysis of rate of YLLs from suicide among different age groups in global and different regions, 1990-2021.. 88
Figure S12. Global map of death cases from suicide in 2021 89
Figure S13. Global map of YLLs from suicide in 2021 90
Figure S14. Global map of rate of YLLs from suicide in 2021 91
Figure S15. Global map of AAPC in rate of YLLs from suicide from 1990 to 2021.............. 92
Figure S16. Trends for rate of YLLs of suicide among 21 regions by SDI for males (A), females (B), and both sexes (C) in 10-24 years population combined from 1990 to 2021 93
Figure S17. Trends for mortality of suicide among 204 countries by SDI for both sexes in 10-24 years population in 2021 94
Figure S18. Trends for rate of YLLs of suicide among 204 countries by SDI for both sexes in

10-24 years population in 2021 95
Figure S19. The trends in suicide mortality in 21 regions, 1990-2021 96
Figure S20. The age effects of suicide mortality in 21 regions, 1990-2021 97
Figure S21.The period effects of suicide mortality in 21 regions, 1990-2021 98
Figure S22. The cohort effects of suicide mortality in 21 regions, 1990-2021 99
Figure S23. The trends in suicide mortality in low SDI countries, 1990-2021 100
Figure S24. The trends in suicide mortality in low-middle SDI countries, 1990-2021 101
Figure S25. The trends in suicide mortality in middle SDI countries, 1990-2021 102
Figure S26. The trends in suicide mortality in high-middle SDI countries, 1990-2021 103
Figure S27. The trends in suicide mortality in high SDI countries, 1990-2021 104
Figure S28. The age effects of suicide mortality in low SDI countries, 1990-2021 105
Figure S29. The age effects of suicide mortality in low-middle SDI countries, 1990-2021 . 106
Figure S30. The age effects of suicide mortality in middle SDI countries, 1990-2021 107
Figure S31. The age effects of suicide mortality in high-middle SDI countries, 1990-2021 108
Figure S32. The age effects of suicide mortality in high SDI countries, 1990-2021 109
Figure S33. The period effects of suicide mortality in low SDI countries, 1990-2021 110
Figure S34. The period effects of suicide mortality in low-middle SDI countries, 1990-2021 111
Figure S35. The period effects of suicide mortality in middle SDI countries, 1990-2021 112
Figure S36. The period effects of suicide mortality in high-middle SDI countries, 1990-2021 113
Figure S37. The period effects of suicide mortality in high SDI countries, 1990-2021 114
Figure S38. The cohort effects of suicide mortality in low SDI countries, 1990-2021 115
Figure S39. The cohort effects of suicide mortality in low-middle SDI countries, 1990-2021 116
Figure S40. The cohort effects of suicide mortality in middle SDI countries, 1990-2021 117
Figure S41. The cohort effects of suicide mortality in high-middle SDI countries, 1990-2021 118
Figure S42. The cohort effects of suicide mortality in high SDI countries, 1990-2021 119

Method S1. GBD 2021 methods for suicide mortality estimation

The Global Burden of Disease Study (GBD 2021) study estimated mortality due to 371 causes by age, sex, and location between 1990 and 2021 for 204 countries and territories, the detailed methods for which are available in the GBD 2021 cause of death capstone paper.(GBD 2021 Diseases and Injuries Collaborators, 2024) In GBD 2021, suicide was defined following the International Classification of Diseases (ICD) as death caused by purposely self-inflicted poisoning or injury (ICD-10 codes X60-X64.9, X66-X84.9, Y87.0; ICD-9 codes E950-E959); deaths from unintentional drug overdoses are not included in this definition. For this analysis, we present results for suicide as an aggregate cause of death.

Suicide mortality data sources

A detailed description of the data sources for the cause of death database can be found in the appendix to GBD 2016 cause of death capstone paper.(GBD 2016 Causes of Death Collaborators, 2017) These sources include vital registration systems and verbal autopsy reports.

Addressing bias in input data

Variation in data quality was addressed through a series of methods that include data standardisation and the redistribution of inappropriately coded deaths or "garbage codes" that are not possible causes of death, or that are not specific underlying causes of death but have been entered as the underlying cause of death on death certificates. Undercounting or miss-assignment of deaths from suicide is a known problem in suicide death estimation, and the level and type of miss-assignment differs by location, age, and sex. Correction of miss-assignment is accounted for in GBD in part by reassignment from ICD codes that may include suicide deaths, such as undetermined intent injury codes (Y10-Y34 in ICD-10 and E980-E988 in ICD-9) or exposure to unspecified factor (X59 in ICD-10; E887 in ICD-9), some intermediate causes of death that cannot be specific underlying causes of death (eg septicemia or peritonitis), or as poorly defined or unknown causes of mortality (R99). For distribution of intermediate causes, we used a regression between suicide fractions and intermediate causes by age and sex in each location for each cause of injury. The same regressions were implemented for homicide and

unintentional injuries. Based on scale up betas from these three regressions to one, we redistributed deaths coded to indeterminate causes to suicide, homicide, and unintentional injury. Redistribution of garbage codes is explained in the GBD 2016 cause of death capstone paper.(GBD 2016 Causes of Death Collaborators, 2017)

Suicide mortality estimation

Mortality from suicide was estimated using the cause of death ensemble model (CODEm) developed for the GBD study. Ensemble modelling is a method where a large number of model specifications are systematically tested and reviewed based on their out-of-sample predictive validity; models that perform best are subsequently incorporated into a weighted ensemble model with the highest weights assigned to models with the best out-of sample prediction error. The model for suicide was age-limited such that deaths from self-harm are restricted to a lower limit of age 10 years, due to the difficulty of determining intent for deaths at younger ages.

CODEm models estimate the individual cause-level mortality without taking into account the all-cause mortality. GBD uses the CodCorrect algorithm to ensure that all individual causes add up to the all-cause mortality. After generating underlying cause of death estimates and accompanying uncertainty, this algorithm combines these models into estimates that are consistent with the levels of all-cause mortality estimated for each age-sex-year-location group. Using 1000 draws from the posterior distribution of each cause and 1000 draws from the posterior distribution of the estimation of all-cause mortality, CoDCorrect rescales the sum of cause specific estimates to equal the draws from the all cause distribution.

The approach to cause of death estimation for injuries, including suicide, was as shown in this flowchart:

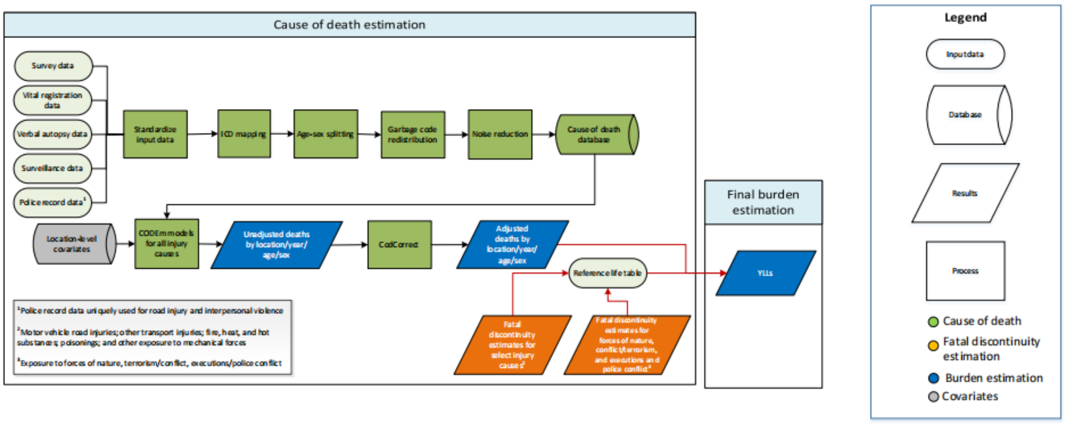

Method Figure S1. Flowchart for suicide mortality estimation

Reference

1. GBD 2016 Causes of Death Collaborators (2017) Global, regional, and national age-sex specific mortality for 264 causes of death, 1980-2016: a systematic analysis for the Global Burden of Disease Study 2016. Lancet 390(10100), 1151-1210.
2. GBD 2021 Diseases and Injuries Collaborators (2024) Global incidence, prevalence, years lived with disability (YLDs), disability-adjusted life-years (DALYs), and healthy life expectancy (HALE) for 371 diseases and injuries in 204 countries and territories and 811 subnational locations, 1990-2021: a systematic analysis for the Global Burden of Disease Study 2021. Lancet 403(10440), 2133-2161.

Method S2. Age, period, and cohort analysis of suicide mortality

We used the web-based Age, period, and cohort model (https://analysistools.cancer.gov/apc/) to analysis the effect of age, period, and cohort on suicide mortality trends across SDI qualities, regions, and countries. Age effect refers to the influence of physiological and social processes on the individual's aging. Period effect is the result of external factors affecting all age groups at a given time. Cohort effect is changes caused by unique experiences in different periods.(Bell, 2020; Yang et al., 2018) These three basic temporal dimensions can provide insight into the epidemiology of the disease and help identify potential gaps in different aspects of the prevention, management and treatment programs for diseases. However, the covariance of these three variables (age = period - birth cohort) leads to a statistical impossibility to estimate the independent effects of age, period and birth cohort.(Bell, 2020; Rosenberg and Anderson, 2011) To avoid this problem, we generate estimable APC parameters and functions without imposing arbitrary constraints on the model.

GBD 2021 estimates for suicide death cases and population data of each country/region were used as data inputs for the age, period, and cohort model. Before formal analysis, a total of 15 countries were excluded because the number of suicide deaths among 10-24 years was less than 1 for several consecutive years **(Method Table S1)**. In a typical age, period, and cohort model, the age and period interval must be equal, so ages are divided into 5-year age groups (10-14 years, 15-19 years, and 20-24 years), and periods are divided into 5-year groups (1992-1996, 1997-2001, ···, 2017-2021). Finally, eight partially coincidental birth cohorts are generated by age groups and period groups (1967-1976, 1972-1981, ···, 2022-2011) **(Method Table S2)**. Furthermore, in the age, period, and cohort model, the age effect is represented longitudinally by a fit of a given number of birth cohorts adjusted for period bias to a specific age rate. The period/cohort effect is expressed by the period/cohort relative mortality, which is calculated as the rate of age-specific mortality in each period/cohort relative to the reference period/cohort. In our study, we chose 10 years old as the reference age and 1990 as the reference period. The choice of reference did not affect the interpretation of the results.(Rosenberg et al., 2014) Statistical significance of these parameters was tested by the Wald χ^2, with a significance level of 0.05. These processes were completed in R 4.2.1.

Method Table S1. List of countries screened out due to unsatisfactory data quality

SDI quintile regions	Country
Low SDI	
Low-middle SDI	Sao Tome and Principe, Tuvalu
Middle SDI	Grenada, Saint Vincent and the Grenadines, Tokelau
High-middle SDI	Saint Kitts and Nevis, Antigua and Barbuda, Cook Islands, Dominica, Niue, Seychelles
High SDI	Andorra, Bermuda, Monaco, San Marino

Method Table S2. The lexis diagram of suicide data for the age, period, and cohort model

Cohort group		Period group					
		1992-1996	1997-2001	2002-2006	2007-2011	2012-2016	2017-2021
Age group	10-14	1977-1986	1982-1991	1987-1996	1992-2001	1997-2006	2002-2011
	15-19	1972-1981	1977-1986	1982-1991	1987-1996	1992-2001	1997-2006
	20-24	1967-1976	1972-1981	1977-1986	1982-1991	1987-1996	1992-2001

Reference

1. Bell A (2020) Age period cohort analysis: a review of what we should and shouldn't do. Ann Hum Biol 47(2), 208-217.
2. Rosenberg PS and Anderson WF (2011) Age-period-cohort models in cancer surveillance research: ready for prime time? Cancer Epidemiol Biomarkers Prev 20(7), 1263-1268.
3. Rosenberg PS, Check DP and Anderson WF (2014) A web tool for age-period-cohort analysis of cancer incidence and mortality rates. Cancer Epidemiol Biomarkers Prev 23(11), 2296-2302.
4. Yang J, Zhang Y, Luo L, Meng R and Yu C (2018) Global Mortality Burden of Cirrhosis and Liver Cancer Attributable to Injection Drug Use, 1990-2016: An Age-Period-Cohort and Spatial Autocorrelation Analysis. Int J Environ Res Public Health 15(1).

Table S1. The mortality and death cases from suicide among whole population between WHO estimates in 2021 and GBD estimates in 2021

	WHO estimates in 2021			GBD estimates in 2021		
	Both	Male	Female	Both	Male	Female
Estimated number of death cases from suicide in whole population	717,000 (590,000 to 892,000)	-	-	746,379 (691,760 to 799,847)	-	-
Estimated crude rate of suicide mortality in whole population	9.1 (6.9 to 11.6)	12.3 (9.6 to 15.4)	5.9 (4.2 to 7.7)	9.5 (8.8 to 10.1)	13.1 (12.3 to 14.0)	5.8 (5.1 to 6.5)

Reference:

1. World Health Organization (2024) World health statistics 2024: monitoring health for the SDGs, sustainable development goals. Geneva. https://www.who.int/publications/i/item/9789240094703 (accessed 21 May 2024).
2. Global Burden of Disease (2024) GBD Results. Washington. https://vizhub.healthdata.org/gbd-results/ (accessed 16 May 2024).

Table S2. The rate of suicide mortality and their AAPC from 1990 to 2021 in 204 regions

	Cases (n), 1990	Mortality (per 100,000 population), 1990	Cases (n), 2021	Mortality (per 100,000 population), 2021	AAPC: 1990-2021	P value	Order
**Andean Latin America**							
Bolivia (Plurinational State of)	132 (101 - 169)	6.6 (5 - 8.5)	177 (120 - 247)	5.5 (3.7 - 7.6)	-0.6 (-0.7 to -0.5)	<0.001	74
Ecuador	193 (180 - 205)	5.9 (5.5 - 6.3)	469 (389 - 555)	9.7 (8 - 11.4)	1.2 (-1.3 to 3.8)	0.332	36
Peru	234 (186 - 286)	3.3 (2.6 - 4.1)	278 (188 - 370)	3 (2.1 - 4)	-0.3 (-1.5 to 1)	0.698	152
**Australasia**							
Australia	471 (450 - 493)	11.9 (11.3 - 12.4)	448 (418 - 475)	9.5 (8.8 - 10)	-0.7 (-1.4 to -0.1)	0.032	38
New Zealand	135 (127 - 141)	16 (15.1 - 16.8)	108 (100 - 115)	10.7 (9.9 - 11.3)	-1.4 (-2.7 to 0)	0.045	**26**
**Caribbean**							
Antigua and Barbuda	0 (0 - 0)	0.9 (0.8 - 1.1)	0 (0 - 0)	0.8 (0.6 - 0.9)	-0.2 (-1.6 to 1.3)	0.784	202
Bahamas	1 (1 - 1)	1.6 (1.4 - 1.8)	1 (1 - 2)	1.4 (1 - 1.9)	-0.3 (-0.9 to 0.2)	0.183	193
Barbados	2 (2 - 2)	3.1 (2.7 - 3.4)	1 (1 - 1)	1.9 (1.4 - 2.6)	-1.2 (-1.7 to -0.6)	<0.001	182
Belize	2 (2 - 2)	3 (2.7 - 3.4)	4 (3 - 5)	2.9 (2.5 - 3.5)	0.3 (-0.1 to 0.7)	0.173	157
Bermuda	0 (0 - 0)	3.4 (3 - 3.8)	0 (0 - 0)	1.3 (1.1 - 1.6)	-3.3 (-3.8 to -2.7)	<0.001	194
Cuba	448 (426 - 469)	14.4 (13.7 - 15.1)	82 (69 - 94)	4.2 (3.5 - 4.8)	-3.9 (-5.6 to -2.1)	<0.001	110
Dominica	0 (0 - 1)	2 (1.6 - 2.5)	0 (0 - 0)	2 (1.4 - 2.7)	0 (-0.2 to 0.2)	0.751	180
Dominican Republic	84 (69 - 101)	3.5 (2.9 - 4.2)	104 (72 - 139)	3.6 (2.5 - 4.9)	0.1 (-0.6 to 0.7)	0.844	129
Grenada	1 (1 - 1)	3.3 (2.8 - 3.8)	0 (0 - 1)	1.9 (1.6 - 2.3)	-1.8 (-2.3 to -1.2)	<0.001	181
Guyana	46 (37 - 54)	17.8 (14.1 - 21)	43 (32 - 55)	20.3 (15.5 - 26.5)	0.5 (-0.7 to 1.6)	0.433	**10**
Haiti	127 (62 - 178)	6.5 (3.2 - 9.2)	186 (99 - 267)	5 (2.6 - 7.1)	-0.5 (-0.7 to -0.3)	<0.001	85
Jamaica	4 (3 - 5)	0.5 (0.4 - 0.7)	4 (3 - 5)	0.5 (0.4 - 0.7)	0.2 (-1.5 to 1.9)	0.809	204
Puerto Rico	44 (41 - 48)	4.5 (4.1 - 4.9)	13 (10 - 16)	2.1 (1.7 - 2.6)	-2.6 (-3.3 to -1.9)	<0.001	177

Saint Kitts and Nevis	0 (0 - 0)	1.8 (1.6 - 2)	0 (0 - 0)	1.4 (1.1 - 2.1)	-1.3 (-2.1 to -0.6)	0.001	191
Saint Lucia	2 (1 - 2)	3.6 (3.2 - 4)	1 (1 - 1)	3 (2.4 - 3.8)	-0.7 (-1.2 to -0.2)	0.007	151
Saint Vincent and the Grenadines	1 (1 - 1)	3 (2.6 - 3.4)	1 (1 - 1)	2.7 (2.2 - 3.2)	-0.4 (-1.7 to 0.9)	0.529	165
Suriname	22 (12 - 26)	18.1 (10 - 21.6)	22 (18 - 27)	15.6 (12.6 - 19.1)	-0.4 (-1.7 to 1)	0.569	**14**
Trinidad and Tobago	43 (40 - 46)	12.5 (11.6 - 13.4)	21 (16 - 27)	7.6 (5.7 - 9.8)	-1.4 (-3 to 0.2)	0.08	49
United States Virgin Islands	1 (1 - 2)	4.5 (3.4 - 5.8)	1 (1 - 1)	6.1 (4.1 - 9.2)	1.1 (-0.1 to 2.3)	0.081	64
**Central Europe**							
Albania	37 (30 - 44)	3.7 (3.1 - 4.5)	9 (7 - 12)	1.7 (1.2 - 2.2)	-2.8 (-4 to -1.5)	<0.001	186
Bosnia and Herzegovina	110 (93 - 126)	9.6 (8.1 - 11)	24 (19 - 29)	4.3 (3.4 - 5.3)	-2.7 (-3.9 to -1.5)	<0.001	101
Bulgaria	168 (156 - 180)	9.1 (8.5 - 9.7)	47 (39 - 53)	4.8 (4 - 5.5)	-2.4 (-3 to -1.7)	<0.001	91
Croatia	85 (79 - 90)	8.2 (7.7 - 8.8)	24 (21 - 27)	3.7 (3.2 - 4.2)	-2.4 (-3.3 to -1.5)	<0.001	126
Czechia	184 (172 - 195)	7.7 (7.2 - 8.2)	68 (61 - 74)	4.3 (3.9 - 4.7)	-2 (-3.5 to -0.5)	0.007	102
Hungary	245 (231 - 259)	10.6 (10 - 11.2)	74 (66 - 81)	5.1 (4.5 - 5.6)	-2.6 (-3.6 to -1.5)	<0.001	84
Montenegro	13 (11 - 15)	8.2 (6.8 - 9.7)	6 (5 - 8)	5.4 (4.4 - 6.6)	-1.3 (-4.1 to 1.7)	0.398	78
North Macedonia	24 (20 - 30)	4.8 (3.8 - 5.9)	12 (8 - 16)	3.1 (2.2 - 4.2)	-1.3 (-1.7 to -0.9)	<0.001	143
Poland	683 (660 - 706)	8 (7.7 - 8.2)	417 (390 - 440)	7.2 (6.7 - 7.6)	-0.3 (-1 to 0.4)	0.349	54
Romania	288 (267 - 311)	4.9 (4.5 - 5.3)	121 (104 - 140)	3.9 (3.4 - 4.5)	-0.9 (-2 to 0.2)	0.098	115
Serbia	233 (187 - 272)	10.6 (8.5 - 12.4)	50 (40 - 61)	3.1 (2.5 - 3.8)	-3.9 (-4.7 to -3)	<0.001	146
Slovakia	84 (72 - 99)	6.6 (5.6 - 7.7)	35 (29 - 43)	4.2 (3.4 - 5.1)	-1.5 (-2.4 to -0.5)	0.002	107
Slovenia	53 (49 - 56)	11.9 (11.2 - 12.7)	12 (11 - 13)	4 (3.6 - 4.5)	-3.8 (-4.5 to -3.1)	<0.001	112
**Central Asia**							
Armenia	25 (24 - 27)	2.9 (2.7 - 3)	13 (12 - 15)	2.5 (2.2 - 2.8)	-0.2 (-1.9 to 1.6)	0.849	170
Azerbaijan	52 (46 - 64)	2.5 (2.2 - 3)	40 (30 - 53)	1.8 (1.4 - 2.4)	-0.9 (-2 to 0.1)	0.088	184
Georgia	49 (45 - 54)	3.8 (3.5 - 4.2)	27 (24 - 31)	4.4 (3.8 - 5)	0.6 (-0.4 to 1.7)	0.258	99
Kazakhstan	608 (559 - 659)	13.9 (12.8 - 15.1)	401 (362 - 439)	9.8 (8.8 - 10.7)	-1 (-1.6 to -0.4)	0.001	34

Kyrgyzstan	119 (104 - 132)	9.2 (8.1 - 10.2)	106 (87 - 126)	6 (4.9 - 7.1)	-1.3 (-2 to -0.6)	<0.001	66
Mongolia	93 (70 - 117)	13.1 (9.9 - 16.5)	83 (63 - 98)	10.9 (8.2 - 12.8)	-0.6 (-2.2 to 1.1)	0.49	**25**
Tajikistan	64 (56 - 75)	3.9 (3.4 - 4.5)	71 (51 - 110)	2.6 (1.8 - 4)	-1.2 (-1.8 to -0.7)	<0.001	169
Turkmenistan	89 (80 - 99)	7.8 (7 - 8.6)	126 (98 - 160)	9.4 (7.4 - 12)	0.6 (-0.5 to 1.7)	0.276	39
Uzbekistan	413 (375 - 451)	6.5 (5.9 - 7.1)	821 (702 - 951)	10.3 (8.8 - 11.9)	1.5 (0.8 to 2.3)	<0.001	31
**Central Latin America**							
Colombia	454 (423 - 490)	4.4 (4.1 - 4.8)	893 (751 - 1043)	7.5 (6.3 - 8.7)	1.6 (0.6 to 2.6)	0.001	51
Costa Rica	42 (38 - 46)	4.5 (4.1 - 4.9)	84 (76 - 94)	7.7 (6.9 - 8.6)	1.9 (0.1 to 3.7)	0.038	46
El Salvador	222 (185 - 256)	12.5 (10.4 - 14.4)	135 (104 - 171)	7.7 (5.9 - 9.8)	-2.2 (-3.9 to -0.5)	0.013	47
Guatemala	125 (114 - 137)	4.7 (4.3 - 5.1)	303 (258 - 349)	6.1 (5.2 - 7.1)	0.5 (-1.2 to 2.3)	0.57	62
Honduras	72 (59 - 89)	4.7 (3.9 - 5.8)	88 (55 - 133)	2.7 (1.7 - 4.2)	-1.7 (-1.9 to -1.5)	<0.001	162
Mexico	879 (842 - 919)	3 (2.9 - 3.2)	2324 (2137 - 2516)	7 (6.4 - 7.6)	2.8 (2.3 to 3.3)	<0.001	56
Nicaragua	68 (58 - 90)	5.2 (4.4 - 6.9)	102 (80 - 128)	5.4 (4.2 - 6.8)	0.1 (-0.6 to 0.7)	0.85	75
Panama	32 (29 - 35)	4.2 (3.8 - 4.6)	53 (44 - 63)	4.8 (4 - 5.8)	0.5 (0 to 0.9)	0.041	89
Venezuela (Bolivarian Republic of)	334 (310 - 359)	5.6 (5.2 - 6)	519 (401 - 681)	8.8 (6.8 - 11.5)	2.1 (1.1 to 3)	<0.001	41
**Central Sub-Saharan Africa**							
Angola	164 (119 - 221)	5.1 (3.7 - 6.9)	396 (264 - 555)	3.8 (2.5 - 5.3)	-0.9 (-1.8 to 0)	0.062	124
Central African Republic	55 (39 - 74)	6.5 (4.6 - 8.8)	107 (74 - 154)	5.9 (4.1 - 8.5)	-0.4 (-0.5 to -0.3)	<0.001	69
Congo	42 (30 - 58)	5.1 (3.7 - 7.1)	72 (51 - 101)	4.3 (3.1 - 6)	-0.4 (-1.1 to 0.3)	0.279	104
Democratic Republic of the Congo	519 (379 - 723)	4.3 (3.2 - 6)	1212 (835 - 1881)	4.1 (2.8 - 6.3)	-0.3 (-0.6 to 0.1)	0.12	111
Equatorial Guinea	8 (5 - 11)	5.9 (4 - 8.3)	23 (13 - 37)	4.3 (2.4 - 6.8)	-1 (-1.3 to -0.6)	<0.001	105
Gabon	13 (9 - 18)	4.3 (3 - 5.9)	22 (15 - 33)	4 (2.6 - 5.8)	-0.1 (-0.4 to 0.1)	0.208	113

**East Asia**							
China	42202 (27618 - 48504)	11.7 (7.7 - 13.4)	6582 (5419 - 8340)	2.8 (2.3 - 3.6)	-4.5 (-4.8 to -4.1)	<0.001	160
Democratic People's Republic of Korea	404 (271 - 581)	7.3 (4.9 - 10.6)	265 (184 - 394)	4.9 (3.4 - 7.2)	-1.3 (-1.4 to -1.2)	<0.001	88
Taiwan (Province of China)	238 (225 - 254)	4.2 (3.9 - 4.4)	188 (171 - 206)	5.4 (4.8 - 5.9)	0.9 (0.2 to 1.6)	0.011	79
**Eastern Europe**							
Belarus	198 (184 - 211)	9 (8.3 - 9.5)	113 (93 - 135)	8 (6.5 - 9.5)	-0.6 (-1.3 to 0)	0.057	45
Estonia	47 (42 - 51)	14.3 (13 - 15.6)	13 (11 - 15)	6.5 (5.5 - 7.3)	-2.7 (-4.5 to -0.8)	0.005	60
Latvia	69 (64 - 73)	12.7 (11.9 - 13.5)	16 (14 - 18)	6 (5.2 - 6.6)	-2.4 (-2.8 to -1.9)	<0.001	67
Lithuania	97 (92 - 103)	11.9 (11.2 - 12.6)	38 (34 - 42)	9.2 (8.2 - 10.2)	-0.7 (-2.4 to 1.1)	0.452	40
Republic of Moldova	83 (77 - 89)	8.1 (7.5 - 8.7)	30 (26 - 34)	5.5 (4.8 - 6.2)	-1.4 (-2.2 to -0.5)	0.002	73
Russian Federation	4566 (4487 - 4641)	14.5 (14.3 - 14.8)	2289 (2111 - 2454)	9.7 (9 - 10.4)	-1.5 (-2.2 to -0.8)	<0.001	35
Ukraine	891 (837 - 951)	8.2 (7.7 - 8.8)	688 (504 - 875)	10.5 (7.7 - 13.3)	0.7 (-0.2 to 1.5)	0.11	**27**
**Eastern Sub-Saharan Africa**							
Burundi	125 (84 - 184)	7.5 (5 - 11)	184 (124 - 295)	4.3 (2.9 - 6.8)	-1.9 (-2.3 to -1.4)	<0.001	106
Comoros	6 (2 - 9)	3.6 (1.2 - 5.8)	7 (4 - 10)	3.1 (2 - 4.5)	-1.2 (-6.4 to 4.2)	0.647	147
Djibouti	4 (2 - 6)	2.4 (1.4 - 4.3)	9 (5 - 16)	2.6 (1.4 - 4.6)	0.4 (-0.1 to 0.9)	0.089	167
Eritrea	64 (46 - 86)	5.7 (4.1 - 7.6)	111 (73 - 161)	5.3 (3.5 - 7.7)	-0.2 (-0.6 to 0.1)	0.233	81
Ethiopia	1088 (742 - 1345)	6.8 (4.6 - 8.4)	1260 (988 - 1536)	3.3 (2.6 - 4.1)	-2.3 (-2.4 to -2.1)	<0.001	139
Kenya	225 (180 - 351)	2.8 (2.3 - 4.4)	520 (390 - 786)	3 (2.3 - 4.6)	0.2 (-0.1 to 0.5)	0.162	153
Madagascar	181 (136 - 231)	4.7 (3.6 - 6)	403 (271 - 561)	4.2 (2.8 - 5.8)	-0.4 (-0.5 to -0.3)	<0.001	109
Malawi	168 (125 - 225)	5.3 (4 - 7.1)	349 (243 - 485)	4.9 (3.4 - 6.8)	-0.2 (-0.6 to 0.2)	0.257	87

Mozambique	169 (116 - 224)	4.1 (2.8 - 5.4)	575 (377 - 835)	5.4 (3.6 - 7.9)	0.9 (0.7 to 1.1)	<0.001	76
Rwanda	182 (131 - 236)	8 (5.8 - 10.4)	167 (110 - 246)	3.9 (2.6 - 5.7)	-2.4 (-2.8 to -1.9)	<0.001	118
Somalia	89 (52 - 152)	3.5 (2 - 6)	273 (157 - 459)	3.8 (2.2 - 6.3)	0.3 (0.1 to 0.5)	0.01	123
South Sudan	72 (47 - 101)	3.6 (2.4 - 5.1)	158 (107 - 224)	4.6 (3.1 - 6.6)	1 (-0.1 to 2.1)	0.088	95
Uganda	170 (114 - 241)	3 (2 - 4.3)	554 (393 - 769)	3.6 (2.6 - 5.1)	0.5 (0.2 to 0.9)	0.001	127
United Republic of Tanzania	348 (264 - 450)	4.1 (3.1 - 5.3)	591 (409 - 840)	3.1 (2.1 - 4.4)	-0.9 (-1.1 to -0.7)	<0.001	148
Zambia	138 (105 - 176)	5 (3.8 - 6.3)	305 (204 - 446)	4.6 (3.1 - 6.8)	-0.2 (-0.6 to 0.1)	0.224	96
**High-income Asia Pacific**							
Brunei Darussalam	3 (2 - 4)	3.5 (2.2 - 4.8)	2 (1 - 2)	1.6 (1.2 - 2)	-2.7 (-3.5 to -1.9)	<0.001	189
Japan	1679 (1645 - 1711)	6 (5.9 - 6.1)	1762 (1728 - 1790)	10 (9.8 - 10.2)	1.8 (0.9 to 2.6)	<0.001	33
Republic of Korea	1257 (1078 - 2194)	9.6 (8.2 - 16.7)	648 (470 - 714)	8.4 (6.1 - 9.3)	-0.1 (-1 to 0.7)	0.744	44
Singapore	73 (68 - 77)	8.8 (8.3 - 9.4)	51 (47 - 55)	7.1 (6.6 - 7.6)	-0.7 (-1.9 to 0.6)	0.293	55
**High-income North America**							
Canada	688 (655 - 721)	11.8 (11.2 - 12.4)	656 (612 - 699)	10.1 (9.5 - 10.8)	-0.5 (-0.9 to -0.1)	0.018	32
Greenland	13 (9 - 15)	91.8 (67.1 - 105.4)	5 (4 - 6)	43.3 (33.3 - 51.3)	-2.3 (-2.5 to -2.2)	<0.001	**1**
United States of America	5394 (5302 - 5498)	9.7 (9.6 - 9.9)	6697 (6445 - 6937)	10.3 (9.9 - 10.7)	0.2 (-0.1 to 0.5)	0.244	**30**
**North Africa and Middle East**							
Afghanistan	134 (90 - 257)	4 (2.7 - 7.6)	339 (229 - 665)	3.2 (2.2 - 6.2)	-0.6 (-1.2 to 0.1)	0.081	142
Algeria	415 (255 - 539)	4.9 (3 - 6.3)	276 (180 - 360)	2.7 (1.8 - 3.5)	-1.9 (-2 to -1.7)	<0.001	164
Bahrain	8 (6 - 9)	5.9 (4.6 - 7.3)	11 (8 - 15)	3.4 (2.4 - 4.6)	-1.7 (-2.4 to -1.1)	<0.001	133
Egypt	224 (163 - 290)	1.3 (0.9 - 1.7)	303 (217 - 393)	1 (0.7 - 1.3)	-0.7 (-1.4 to -0.1)	0.026	196

Iran (Islamic Republic of)	1405 (989 - 1567)	7.6 (5.4 - 8.5)	833 (711 - 926)	4.7 (4 - 5.2)	-1.6 (-1.8 to -1.3)	<0.001	94
Iraq	320 (233 - 419)	5.2 (3.8 - 6.8)	429 (306 - 584)	3.4 (2.4 - 4.7)	-1.4 (-1.6 to -1.2)	<0.001	132
Jordan	35 (26 - 44)	2.6 (1.9 - 3.3)	34 (25 - 44)	0.9 (0.7 - 1.2)	-3.3 (-3.8 to -2.8)	<0.001	197
Kuwait	7 (6 - 8)	1.5 (1.3 - 1.7)	13 (11 - 16)	1.6 (1.3 - 2)	0.8 (-2.3 to 4)	0.61	188
Lebanon	14 (8 - 19)	1.6 (0.9 - 2.2)	9 (7 - 12)	0.8 (0.6 - 1)	-2.2 (-2.4 to -1.9)	<0.001	199
Libya	60 (37 - 79)	4.1 (2.5 - 5.4)	84 (54 - 116)	4.8 (3.1 - 6.6)	0.6 (-0.1 to 1.2)	0.08	90
Morocco	444 (252 - 599)	5.5 (3.1 - 7.4)	301 (186 - 459)	3.2 (2 - 4.9)	-1.7 (-1.8 to -1.6)	<0.001	141
Oman	8 (5 - 11)	1.4 (1 - 2)	7 (5 - 9)	0.8 (0.6 - 1)	-1.8 (-2.4 to -1.1)	<0.001	201
Palestine	6 (4 - 9)	0.9 (0.7 - 1.3)	13 (9 - 17)	0.8 (0.6 - 1)	-0.5 (-0.8 to -0.2)	0.002	200
Qatar	7 (5 - 9)	6.7 (5.2 - 8.6)	15 (11 - 21)	3.4 (2.4 - 4.6)	-2.4 (-3.1 to -1.6)	<0.001	137
Saudi Arabia	298 (205 - 399)	5.9 (4.1 - 7.9)	346 (246 - 496)	4.2 (3 - 6)	-1.1 (-1.4 to -0.9)	<0.001	108
Sudan	436 (225 - 621)	6.8 (3.5 - 9.6)	680 (373 - 1076)	4.7 (2.6 - 7.5)	-1.1 (-1.4 to -0.9)	<0.001	92
Syrian Arab Republic	66 (48 - 87)	1.5 (1.1 - 2)	37 (27 - 52)	0.8 (0.6 - 1.1)	-2.1 (-2.7 to -1.5)	<0.001	198
Tunisia	89 (58 - 130)	3.3 (2.2 - 4.9)	62 (39 - 95)	2.5 (1.6 - 3.8)	-1 (-1.1 to -0.8)	<0.001	171
Türkiye	769 (484 - 1086)	4.2 (2.6 - 5.9)	737 (501 - 925)	3.8 (2.6 - 4.8)	-0.2 (-0.8 to 0.4)	0.448	120
United Arab Emirates	20 (14 - 29)	4.7 (3.4 - 6.9)	34 (25 - 48)	3.1 (2.2 - 4.3)	-1 (-3.3 to 1.3)	0.385	149
Yemen	123 (33 - 203)	2.9 (0.8 - 4.8)	303 (101 - 502)	2.8 (0.9 - 4.6)	-0.2 (-1.3 to 0.8)	0.661	161
**Oceania**							
American Samoa	1 (1 - 2)	8.3 (6.2 - 10.9)	1 (1 - 2)	8.6 (6.7 - 11)	0.1 (-0.1 to 0.3)	0.373	43
Cook Islands	1 (1 - 1)	17.2 (13.3 - 21.9)	0 (0 - 1)	10.4 (8 - 13.3)	-1.6 (-1.8 to -1.4)	<0.001	**29**
Fiji	33 (26 - 42)	14 (10.8 - 17.5)	27 (20 - 36)	11.3 (8.3 - 15)	-0.6 (-1.2 to -0.1)	0.033	**23**
Guam	10 (8 - 11)	24.3 (21.3 - 27.9)	7 (6 - 8)	18.7 (15.8 - 21.4)	-1.1 (-2.6 to 0.5)	0.168	**13**
Kiribati	6 (4 - 7)	24.7 (18.9 - 31)	8 (6 - 10)	22 (16.2 - 28.1)	-0.4 (-0.4 to -0.3)	<0.001	**8**
Marshall Islands	4 (2 - 6)	27.4 (15.2 - 36.5)	4 (2 - 5)	23.2 (14.3 - 31.3)	-0.8 (-1.2 to -0.4)	<0.001	**5**

Micronesia (Federated States of)	9 (6 - 11)	25.6 (17.4 - 32.8)	7 (5 - 9)	22.4 (15.5 - 30)	-0.4 (-0.5 to -0.3)	<0.001	**6**
Nauru	1 (1 - 1)	30.5 (18.1 - 39.8)	1 (1 - 1)	28.3 (16.3 - 37.4)	-0.2 (-0.4 to -0.1)	<0.001	**2**
Niue	0 (0 - 0)	18 (12.9 - 24)	0 (0 - 0)	25.3 (20.1 - 30.3)	1.1 (0.4 to 1.8)	0.002	**4**
Northern Mariana Islands	2 (1 - 2)	12.9 (9.6 - 17.3)	2 (1 - 2)	13.4 (10.9 - 15.7)	-0.2 (-1.1 to 0.7)	0.62	**19**
Palau	1 (1 - 1)	21.6 (14 - 31.7)	1 (1 - 1)	22.3 (17.1 - 29.1)	0.2 (-0.2 to 0.5)	0.356	**7**
Papua New Guinea	24 (14 - 43)	1.9 (1.1 - 3.3)	63 (41 - 150)	2.1 (1.3 - 4.9)	0.5 (-0.3 to 1.3)	0.202	179
Samoa	11 (8 - 13)	18.1 (13.5 - 22.8)	9 (7 - 12)	14.5 (10.5 - 19.1)	-0.7 (-0.8 to -0.6)	<0.001	**16**
Solomon Islands	24 (10 - 35)	21.2 (8.3 - 30.5)	45 (30 - 60)	21.3 (14.4 - 28.7)	-0.1 (-0.7 to 0.5)	0.672	**9**
Tokelau	0 (0 - 0)	19.2 (13.7 - 25.3)	0 (0 - 0)	26.8 (21.6 - 33.1)	1.5 (0.4 to 2.6)	0.006	**3**
Tonga	2 (1 - 2)	4.8 (3.8 - 6.1)	2 (1 - 2)	5.4 (3.8 - 7.5)	0.3 (0 to 0.6)	0.024	77
Tuvalu	1 (0 - 1)	27.5 (17.6 - 35.3)	1 (0 - 1)	19 (13.9 - 24.6)	-1.2 (-1.3 to -1.1)	<0.001	**12**
Vanuatu	9 (6 - 12)	19.7 (12 - 26.3)	19 (14 - 24)	20 (14.7 - 25.7)	0 (-0.3 to 0.3)	0.851	**11**
**South Asia**							
Bangladesh	4037 (2921 - 4998)	11.6 (8.4 - 14.3)	1980 (1447 - 2873)	4.3 (3.2 - 6.3)	-3 (-3.6 to -2.4)	<0.001	103
Bhutan	11 (7 - 16)	4.6 (2.9 - 7.1)	7 (4 - 15)	3.3 (2 - 7.1)	-1.1 (-1.3 to -0.9)	<0.001	140
India	49543 (37522 - 56352)	19.2 (14.6 - 21.9)	44616 (38126 - 49874)	11.2 (9.6 - 12.5)	-1.8 (-2.8 to -0.7)	0.001	**24**
Nepal	920 (598 - 1252)	15.4 (10 - 21)	1126 (808 - 1559)	11.9 (8.6 - 16.5)	-0.8 (-1 to -0.7)	<0.001	**21**
Pakistan	2072 (1445 - 2854)	5.8 (4.1 - 8)	4431 (2910 - 6517)	6.1 (4 - 9)	0.2 (0 to 0.3)	0.01	63
**Southern Latin America**							
Argentina	446 (415 - 475)	5.1 (4.8 - 5.5)	1114 (1027 - 1199)	10.4 (9.6 - 11.2)	2.3 (1.4 to 3.1)	<0.001	**28**
Chile	544 (503 - 587)	14.5 (13.4 - 15.6)	281 (260 - 304)	7.2 (6.6 - 7.7)	-2.2 (-2.7 to -1.7)	<0.001	53

Uruguay	60 (56 - 65)	7.9 (7.3 - 8.4)	108 (99 - 119)	15.1 (13.7 - 16.5)	2.2 (1.7 to 2.7)	<0.001	**15**
**Southeast Asia**							
Cambodia	145 (107 - 189)	4.5 (3.3 - 5.9)	143 (94 - 211)	3.1 (2 - 4.5)	-1.2 (-1.3 to -1.1)	<0.001	145
Indonesia	1026 (756 - 1238)	1.7 (1.3 - 2.1)	1050 (843 - 1348)	1.5 (1.2 - 1.9)	-0.4 (-0.6 to -0.2)	<0.001	190
Lao People's Democratic Republic	118 (65 - 164)	9.1 (5 - 12.7)	112 (77 - 158)	5.4 (3.7 - 7.5)	-1.7 (-1.9 to -1.5)	<0.001	80
Malaysia	206 (161 - 266)	3.8 (3 - 5)	220 (168 - 294)	2.7 (2.1 - 3.6)	-1.5 (-2.1 to -0.9)	<0.001	163
Maldives	2 (1 - 3)	2.9 (1.9 - 3.8)	1 (1 - 2)	1.4 (1 - 1.9)	-2.2 (-2.9 to -1.5)	<0.001	192
Mauritius	38 (36 - 41)	11.7 (11 - 12.5)	19 (17 - 22)	7.5 (6.5 - 8.4)	-1.4 (-2 to -0.8)	<0.001	50
Myanmar	599 (351 - 857)	4.6 (2.7 - 6.6)	429 (283 - 605)	2.8 (1.9 - 4)	-1.6 (-1.8 to -1.5)	<0.001	159
Philippines	839 (583 - 932)	4.1 (2.8 - 4.6)	1098 (752 - 1313)	3.4 (2.3 - 4)	-0.6 (-0.8 to -0.4)	<0.001	135
Seychelles	1 (1 - 1)	4.8 (3.9 - 5.9)	0 (0 - 0)	1.2 (0.9 - 1.7)	-3.3 (-4.8 to -1.9)	<0.001	195
Sri Lanka	1793 (1465 - 2053)	34.6 (28.3 - 39.6)	613 (451 - 809)	11.7 (8.6 - 15.5)	-3.1 (-4.8 to -1.4)	<0.001	**22**
Thailand	1695 (1308 - 2216)	9.5 (7.3 - 12.4)	754 (563 - 1025)	6.4 (4.8 - 8.7)	-1.3 (-1.8 to -0.7)	<0.001	61
Timor-Leste	14 (9 - 19)	6 (4 - 8.3)	21 (15 - 29)	4.4 (3.1 - 6.1)	-1 (-2.5 to 0.6)	0.215	98
Viet Nam	1088 (723 - 1474)	5.1 (3.4 - 6.8)	815 (586 - 1095)	3.8 (2.8 - 5.2)	-0.9 (-1 to -0.8)	<0.001	119
**Southern Sub-Saharan Africa**							
Botswana	41 (27 - 59)	9.1 (5.9 - 13.1)	43 (29 - 62)	6.5 (4.4 - 9.4)	-1.1 (-1.3 to -0.8)	<0.001	59
Eswatini	23 (17 - 30)	8.2 (6 - 11)	49 (31 - 71)	13.4 (8.4 - 19.3)	1.7 (1.5 to 1.9)	<0.001	**18**
Lesotho	30 (20 - 49)	6.3 (4.2 - 10.3)	88 (60 - 117)	14.3 (9.8 - 19.2)	2.7 (2.3 to 3.1)	<0.001	**17**
Namibia	35 (26 - 47)	7.4 (5.5 - 9.8)	57 (39 - 87)	7.6 (5.2 - 11.6)	0.1 (-0.3 to 0.6)	0.522	48

South Africa	1057 (858 - 1269)	8.9 (7.3 - 10.7)	1367 (1151 - 1605)	9.5 (8 - 11.2)	0.3 (-0.3 to 1)	0.316	37
Zimbabwe	217 (134 - 344)	6.1 (3.7 - 9.6)	679 (432 - 940)	13.3 (8.5 - 18.4)	2.6 (2 to 3.2)	<0.001	**20**
**Tropical Latin America**							
Brazil	1843 (1764 - 1927)	4 (3.8 - 4.1)	2930 (2808 - 3052)	6 (5.8 - 6.3)	1.5 (0.8 to 2.1)	<0.001	65
Paraguay	39 (33 - 47)	3.2 (2.7 - 3.8)	113 (76 - 143)	5.7 (3.8 - 7.2)	2 (1 to 2.9)	<0.001	71
**Western Europe**							
Andorra	1 (0 - 1)	5.3 (3.6 - 7.2)	0 (0 - 1)	3.1 (2.2 - 4.3)	-1.7 (-2.1 to -1.3)	<0.001	144
Austria	190 (178 - 203)	11.7 (11 - 12.5)	83 (75 - 92)	5.9 (5.3 - 6.5)	-2.3 (-2.6 to -2)	<0.001	68
Belgium	171 (161 - 181)	8.5 (8 - 9)	109 (98 - 119)	5.6 (5 - 6.1)	-1.4 (-2.1 to -0.8)	<0.001	72
Cyprus	5 (4 - 7)	2.6 (1.9 - 3.5)	4 (3 - 5)	1.8 (1.3 - 2.4)	-1.1 (-2 to -0.2)	0.017	183
Denmark	84 (79 - 89)	7.7 (7.2 - 8.2)	38 (34 - 42)	3.6 (3.3 - 4)	-2.5 (-3.1 to -2)	<0.001	128
Finland	171 (161 - 180)	17.5 (16.5 - 18.5)	80 (73 - 87)	8.7 (8 - 9.5)	-2.2 (-3.6 to -0.7)	0.003	42
France	1046 (988 - 1108)	8.3 (7.8 - 8.8)	430 (386 - 473)	3.5 (3.2 - 3.9)	-2.6 (-3.1 to -2.1)	<0.001	130
Germany	1177 (1099 - 1250)	7.9 (7.4 - 8.4)	550 (501 - 601)	4.4 (4 - 4.8)	-1.8 (-2.5 to -1.2)	<0.001	100
Greece	47 (43 - 52)	2 (1.8 - 2.2)	25 (22 - 29)	1.6 (1.4 - 1.9)	-0.7 (-1.9 to 0.6)	0.303	187
Iceland	6 (6 - 7)	9.8 (8.9 - 10.7)	4 (3 - 4)	5.3 (4.6 - 6)	-2 (-3.7 to -0.2)	0.025	82
Ireland	64 (58 - 70)	6.5 (6 - 7.2)	38 (32 - 44)	3.9 (3.3 - 4.5)	-1.8 (-2.5 to -1.1)	<0.001	117
Israel	60 (56 - 64)	4.4 (4.1 - 4.7)	55 (48 - 61)	2.4 (2.2 - 2.7)	-1.8 (-3.1 to -0.5)	0.006	172
Italy	417 (403 - 432)	3.3 (3.2 - 3.5)	181 (172 - 192)	2.1 (2 - 2.2)	-1.7 (-2.3 to -1)	<0.001	178
Luxembourg	8 (7 - 9)	11 (9.7 - 12.6)	3 (3 - 4)	3 (2.6 - 3.4)	-3.9 (-6.2 to -1.5)	0.001	154
Malta	2 (2 - 3)	3 (2.6 - 3.3)	2 (1 - 2)	2.4 (2.1 - 2.7)	-0.7 (-2.1 to 0.8)	0.349	174
Monaco	0 (0 - 0)	8.9 (6.7 - 11.4)	0 (0 - 0)	6.7 (5.1 - 9.2)	-0.9 (-1 to -0.8)	<0.001	58

Netherlands	192 (180 - 203)	5.8 (5.5 - 6.2)	149 (138 - 161)	5 (4.6 - 5.3)	-0.6 (-2 to 0.8)	0.369	86
Norway	118 (113 - 122)	12.8 (12.4 - 13.3)	73 (70 - 76)	7.4 (7.1 - 7.7)	-1.7 (-3.5 to 0.2)	0.073	52
Portugal	212 (196 - 226)	8.5 (7.9 - 9.1)	43 (37 - 48)	2.6 (2.3 - 3)	-3.5 (-4.8 to -2.3)	<0.001	166
San Marino	0 (0 - 0)	5.6 (4.2 - 7.1)	0 (0 - 0)	3.4 (2.4 - 4.5)	-1.8 (-1.9 to -1.6)	<0.001	136
Spain	394 (369 - 422)	4 (3.8 - 4.3)	167 (149 - 183)	2.4 (2.1 - 2.6)	-1.8 (-2.9 to -0.7)	0.001	173
Sweden	163 (156 - 171)	9.9 (9.4 - 10.3)	120 (109 - 132)	6.7 (6.2 - 7.4)	-1.4 (-2.7 to -0.1)	0.04	57
Switzerland	185 (176 - 196)	13.9 (13.2 - 14.7)	61 (56 - 66)	4.5 (4.1 - 4.9)	-3.4 (-4.5 to -2.3)	<0.001	97
United Kingdom	854 (840 - 868)	7.2 (7.1 - 7.3)	572 (550 - 590)	4.7 (4.6 - 4.9)	-1.2 (-2 to -0.5)	0.001	93
**Western Sub-Saharan Africa**							
Benin	45 (33 - 60)	3.1 (2.3 - 4.1)	133 (83 - 215)	3 (1.9 - 4.9)	-0.1 (-0.2 to 0)	0.175	150
Burkina Faso	106 (80 - 142)	3.6 (2.7 - 4.8)	243 (171 - 334)	3.3 (2.3 - 4.6)	-0.3 (-0.6 to 0)	0.064	138
Cabo Verde	6 (4 - 7)	5.2 (3.7 - 6.5)	9 (6 - 11)	5.7 (4 - 7.6)	0.4 (-0.2 to 1.1)	0.185	70
Cameroon	109 (79 - 144)	3.4 (2.4 - 4.5)	408 (245 - 610)	3.9 (2.4 - 5.9)	0.5 (0.3 to 0.6)	<0.001	114
Chad	44 (29 - 67)	2.4 (1.6 - 3.6)	202 (129 - 305)	3.5 (2.2 - 5.2)	1.3 (1 to 1.6)	<0.001	131
Côte d'Ivoire	146 (105 - 206)	3.8 (2.8 - 5.4)	320 (202 - 500)	3.7 (2.3 - 5.8)	-0.1 (-0.5 to 0.4)	0.778	125
Gambia	8 (4 - 13)	2.5 (1.4 - 4.2)	28 (17 - 45)	3.4 (2.1 - 5.5)	1 (-0.7 to 2.7)	0.242	134
Ghana	121 (88 - 167)	2.6 (1.9 - 3.5)	311 (219 - 450)	3 (2.1 - 4.3)	0.5 (0.2 to 0.7)	<0.001	156
Guinea	39 (26 - 59)	2.3 (1.5 - 3.5)	129 (75 - 211)	3 (1.7 - 4.9)	0.8 (0.6 to 1)	<0.001	155
Guinea-Bissau	16 (11 - 24)	5.1 (3.2 - 7.3)	34 (24 - 48)	5.1 (3.6 - 7.1)	0 (-0.1 to 0.2)	0.583	83
Liberia	23 (16 - 32)	3.2 (2.2 - 4.3)	68 (41 - 104)	3.8 (2.3 - 5.8)	0.5 (-0.4 to 1.5)	0.256	121
Mali	66 (44 - 97)	2.6 (1.7 - 3.8)	206 (137 - 311)	2.6 (1.7 - 3.9)	0 (-0.3 to 0.4)	0.938	168
Mauritania	14 (9 - 22)	2.2 (1.4 - 3.4)	26 (16 - 43)	1.8 (1.1 - 3)	-0.6 (-0.9 to -0.4)	<0.001	185
Niger	60 (38 - 95)	2.5 (1.5 - 3.9)	190 (109 - 354)	2.3 (1.3 - 4.2)	-0.2 (-0.6 to 0.1)	0.213	175
Nigeria	686 (493 - 882)	2.4 (1.7 - 3.1)	1674 (1160 - 2320)	2.1 (1.5 - 3)	-0.4 (-0.5 to -0.3)	<0.001	176

Sao Tome and Principe	0 (0 - 0)	0.4 (0.2 - 0.7)	0 (0 - 1)	0.6 (0.4 - 0.9)	1.2 (0.6 to 1.9)	<0.001	203
Senegal	99 (73 - 131)	4.1 (3 - 5.4)	197 (139 - 291)	3.8 (2.7 - 5.6)	-0.1 (-0.3 to 0.1)	0.523	122
Sierra Leone	27 (16 - 44)	2.2 (1.3 - 3.6)	85 (46 - 144)	2.9 (1.6 - 5)	0.8 (0.3 to 1.3)	0.001	158
Togo	38 (27 - 52)	3.2 (2.3 - 4.3)	102 (64 - 152)	3.9 (2.5 - 5.8)	0.7 (0.4 to 0.9)	<0.001	116

Note: Order is ranked according to the highest to lowest suicide mortality for 10-24 years population in each country around the world in 2021. The top 30 countries in the world are marked in red.

Table S3. The rate of YLLs of suicide and their AAPC from 1990 to 2021 in 204 regions

	Cases (n), 1990	YLLs (per 100,000 population), 1990	Cases (n), 2021	YLLs (per 100,000 population), 2021	AAPC: 1990-2021	P value	Order
**Andean Latin America**							
Bolivia (Plurinational State of)	9437 (7210 - 12112)	471 (359.8 - 604.5)	12524 (8488 - 17457)	387.2 (262.4 - 539.7)	-0.6 (-0.7 to -0.5)	<0.001	73
Ecuador	13614 (12726 - 14478)	417.6 (390.3 - 444.1)	33149 (27578 - 39122)	682.1 (567.5 - 805)	1.2 (-1.3 to 3.8)	0.337	35
Peru	16549 (13250 - 20309)	234.9 (188 - 288.2)	19583 (13241 - 25941)	213.6 (144.4 - 282.9)	-0.3 (-1.5 to 1)	0.686	150
**Australasia**							
Australia	32792 (31285 - 34312)	826 (788 - 864.2)	31220 (29103 - 33067)	660.8 (616 - 699.9)	-0.7 (-1.4 to -0.1)	0.035	37
New Zealand	9400 (8889 - 9850)	1117.8 (1057 - 1171.2)	7560 (7013 - 8015)	746.7 (692.7 - 791.6)	-1.3 (-2.6 to 0)	0.048	**26**
**Caribbean**							
Antigua and Barbuda	11 (10 - 13)	64.4 (54.8 - 75)	10 (9 - 12)	52.7 (44 - 62)	-0.2 (-1.6 to 1.2)	0.762	202
Bahamas	87 (77 - 99)	108.1 (94.9 - 122.8)	93 (67 - 130)	94.2 (67.6 - 130.8)	-0.3 (-0.9 to 0.3)	0.268	193
Barbados	144 (128 - 161)	212.5 (189.6 - 238.3)	75 (55 - 102)	132.1 (96.1 - 178.1)	-1.2 (-1.7 to -0.6)	<0.001	182
Belize	134 (120 - 150)	214.2 (192.4 - 239.9)	269 (232 - 319)	204 (175.6 - 241.5)	0.3 (-0.2 to 0.7)	0.212	158
Bermuda	29 (26 - 33)	233.9 (208.1 - 263.8)	8 (7 - 10)	89.4 (74.1 - 110.8)	-3.2 (-3.8 to -2.7)	<0.001	194
Cuba	31253 (29722 - 32727)	1004.9 (955.6 - 1052.3)	5697 (4807 - 6570)	291.9 (246.3 - 336.6)	-3.9 (-5.6 to -2.1)	<0.001	109
Dominica	32 (25 - 40)	139.2 (109.8 - 174.4)	23 (17 - 31)	139.2 (100.5 - 186.3)	0 (-0.2 to 0.2)	0.877	180
Dominican Republic	5865 (4867 - 7064)	246.2 (204.3 - 296.5)	7226 (5028 - 9665)	252.9 (176 - 338.3)	0 (-0.6 to 0.7)	0.887	128
Grenada	60 (52 - 69)	229.4 (197.4 - 262.2)	34 (28 - 40)	133.3 (111.4 - 157.7)	-1.8 (-2.3 to -1.2)	<0.001	181
Guyana	3239 (2556 - 3808)	1249.3 (985.7 - 1468.6)	2944 (2249 - 3838)	1409 (1076.5 - 1837)	0.4 (-0.7 to 1.6)	0.468	**10**
Haiti	9003 (4381 - 12625)	463.8 (225.7 - 650.4)	13144 (6967 - 18830)	351.4 (186.3 - 503.4)	-0.5 (-0.7 to -0.3)	<0.001	85
Jamaica	261 (197 - 348)	34.2 (25.8 - 45.6)	250 (187 - 333)	35.2 (26.3 - 46.9)	0.2 (-1.5 to 1.9)	0.85	204
Puerto Rico	3073 (2820 - 3334)	312.8 (287 - 339.3)	891 (716 - 1097)	145.7 (117 - 179.4)	-2.6 (-3.3 to -1.9)	<0.001	177
Saint Kitts and Nevis	16 (14 - 18)	126.8 (113.4 - 142.2)	12 (9 - 18)	100.5 (73.9 - 145.5)	-1.4 (-2.1 to -0.6)	<0.001	191

Saint Lucia	114 (103 - 128)	251.9 (226.4 - 282.9)	76 (61 - 95)	209.8 (167.5 - 261.7)	-0.8 (-1.3 to -0.2)	0.011	154
Saint Vincent and the Grenadines	79 (69 - 89)	210.8 (184.4 - 237.9)	49 (41 - 58)	185 (153.6 - 220.2)	-0.5 (-1.5 to 0.6)	0.37	165
Suriname	1551 (851 - 1847)	1269.1 (696.5 - 1511.4)	1542 (1251 - 1887)	1084.9 (880.2 - 1327.5)	-0.4 (-1.7 to 0.9)	0.56	**14**
Trinidad and Tobago	2981 (2782 - 3206)	868.6 (810.5 - 934)	1441 (1082 - 1857)	525.8 (394.9 - 677.5)	-1.4 (-3 to 0.1)	0.074	50
United States Virgin Islands	89 (67 - 115)	317.6 (240 - 408)	58 (39 - 88)	416.4 (280 - 631.2)	1 (-0.2 to 2.2)	0.111	67
**Central Europe**							
Albania	2572 (2114 - 3075)	262.6 (215.9 - 313.9)	621 (454 - 824)	117.5 (86 - 155.9)	-2.8 (-4 to -1.6)	<0.001	186
Bosnia and Herzegovina	7705 (6514 - 8788)	670.1 (566.5 - 764.3)	1628 (1294 - 1982)	300.7 (239 - 366.1)	-2.7 (-3.9 to -1.5)	<0.001	106
Bulgaria	11869 (11029 - 12708)	644 (598.4 - 689.6)	3249 (2742 - 3707)	334.7 (282.5 - 381.9)	-2.4 (-3 to -1.8)	<0.001	91
Croatia	5904 (5526 - 6310)	571.4 (534.9 - 610.7)	1695 (1462 - 1912)	255.9 (220.8 - 288.8)	-2.4 (-3.3 to -1.5)	<0.001	127
Czechia	12893 (12068 - 13720)	542.7 (508 - 577.5)	4720 (4251 - 5155)	302.2 (272.2 - 330.1)	-2 (-3.5 to -0.6)	0.006	104
Hungary	17187 (16206 - 18111)	745.6 (703 - 785.7)	5168 (4554 - 5655)	355.8 (313.5 - 389.3)	-2.5 (-3.7 to -1.3)	<0.001	84
Montenegro	905 (756 - 1063)	570.6 (476.5 - 670.4)	443 (360 - 542)	378.2 (307.1 - 462.6)	-1.3 (-4.1 to 1.7)	0.395	78
North Macedonia	1710 (1379 - 2109)	334.9 (270.2 - 413)	809 (562 - 1091)	219.1 (152.3 - 295.4)	-1.3 (-1.7 to -0.9)	<0.001	143
Poland	47857 (46273 - 49474)	558.2 (539.7 - 577.1)	28880 (27029 - 30480)	496.2 (464.4 - 523.7)	-0.3 (-0.9 to 0.4)	0.439	54
Romania	20270 (18810 - 21900)	343.6 (318.8 - 371.2)	8509 (7320 - 9808)	274.3 (236 - 316.2)	-0.9 (-2 to 0.2)	0.098	116
Serbia	16363 (13125 - 19140)	748.3 (600.2 - 875.2)	3474 (2820 - 4229)	215.1 (174.6 - 261.9)	-3.9 (-4.7 to -3)	<0.001	148
Slovakia	5924 (5050 - 6932)	465.4 (396.8 - 544.7)	2463 (2001 - 2980)	294.7 (239.4 - 356.5)	-1.5 (-2.4 to -0.5)	0.002	108
Slovenia	3674 (3449 - 3921)	829 (778.3 - 884.8)	835 (745 - 926)	282.4 (251.8 - 312.9)	-3.9 (-4.6 to -3.2)	<0.001	112
**Central Asia**							
Armenia	1780 (1689 - 1881)	204.4 (193.9 - 216)	943 (830 - 1056)	175 (154 - 196)	-0.2 (-2 to 1.6)	0.835	170
Azerbaijan	3688 (3220 - 4521)	174.4 (152.2 - 213.8)	2830 (2109 - 3738)	128.3 (95.6 - 169.4)	-0.9 (-2 to 0.1)	0.089	183
Georgia	3478 (3180 - 3832)	270.8 (247.6 - 298.3)	1916 (1666 - 2182)	307.5 (267.4 - 350.2)	0.6 (-0.5 to 1.7)	0.278	99

Kazakhstan	42896 (39423 - 46527)	982 (902.5 - 1065.1)	28272 (25488 - 30929)	689.2 (621.3 - 753.9)	-1 (-1.6 to -0.5)	<0.001	34
Kyrgyzstan	8452 (7419 - 9386)	654.2 (574.3 - 726.5)	7494 (6154 - 8875)	422.3 (346.9 - 500.2)	-1.4 (-2.1 to -0.6)	<0.001	64
Mongolia	6601 (4989 - 8261)	931.9 (704.2 - 1166.2)	5836 (4412 - 6896)	768.5 (581 - 908.2)	-0.6 (-1.8 to 0.7)	0.386	**25**
Tajikistan	4567 (3952 - 5338)	275.5 (238.4 - 322)	5040 (3583 - 7796)	181.3 (128.9 - 280.5)	-1.3 (-1.8 to -0.7)	<0.001	169
Turkmenistan	6309 (5677 - 6994)	550.4 (495.2 - 610.1)	8794 (6871 - 11185)	659.1 (515 - 838.4)	0.6 (-0.6 to 1.7)	0.324	39
Uzbekistan	29412 (26684 - 32136)	460.4 (417.7 - 503)	58085 (49672 - 67159)	725.2 (620.1 - 838.4)	1.5 (0.8 to 2.3)	<0.001	**29**
**Central Latin America**							
Colombia	31874 (29642 - 34373)	311.3 (289.5 - 335.7)	62995 (53028 - 73369)	526.3 (443.1 - 613)	1.7 (0.7 to 2.7)	0.001	49
Costa Rica	2925 (2670 - 3195)	317.5 (289.8 - 346.8)	5893 (5304 - 6530)	538.9 (485 - 597.1)	2 (-0.1 to 4)	0.058	47
El Salvador	15659 (13121 - 18011)	881.3 (738.5 - 1013.7)	9476 (7324 - 11963)	542 (418.9 - 684.3)	-2.2 (-3.9 to -0.5)	0.013	46
Guatemala	8868 (8093 - 9676)	332.5 (303.4 - 362.8)	21388 (18234 - 24635)	432.5 (368.7 - 498.2)	0.5 (-1.2 to 2.2)	0.562	62
Honduras	5146 (4210 - 6318)	335.7 (274.6 - 412.1)	6164 (3886 - 9313)	193.6 (122 - 292.4)	-1.8 (-2 to -1.5)	<0.001	162
Mexico	61910 (59272 - 64674)	213.1 (204 - 222.6)	163040 (149876 - 176854)	491.4 (451.7 - 533)	2.8 (2.3 to 3.3)	<0.001	56
Nicaragua	4860 (4115 - 6450)	370.4 (313.7 - 491.6)	7204 (5598 - 8948)	382.9 (297.5 - 475.6)	0 (-0.6 to 0.7)	0.928	76
Panama	2238 (2024 - 2468)	296.1 (267.9 - 326.6)	3749 (3106 - 4454)	340.1 (281.8 - 404)	0.5 (0 to 0.9)	0.038	88
Venezuela (Bolivarian Republic of)	23515 (21859 - 25219)	392.4 (364.8 - 420.9)	36560 (28257 - 47963)	616.8 (476.7 - 809.1)	2.1 (1.1 to 3)	<0.001	41
**Central Sub-Saharan Africa**							
Angola	11747 (8509 - 15781)	366.6 (265.5 - 492.5)	28108 (18772 - 39347)	266.2 (177.8 - 372.6)	-0.9 (-1.8 to 0)	0.048	124
Central African Republic	3900 (2779 - 5291)	463 (330 - 628.2)	7618 (5241 - 10889)	419.9 (288.9 - 600.1)	-0.4 (-0.5 to -0.3)	<0.001	66
Congo	2966 (2143 - 4053)	364.1 (263 - 497.4)	5040 (3630 - 7043)	302.3 (217.7 - 422.4)	-0.4 (-1.2 to 0.3)	0.264	103
Democratic Republic of the Congo	37021 (27090 - 51501)	308.3 (225.6 - 428.9)	85857 (59265 - 134252)	288.2 (198.9 - 450.6)	-0.3 (-0.7 to 0.1)	0.179	111
Equatorial Guinea	554 (378 - 770)	424.8 (289.7 - 590.7)	1647 (941 - 2596)	300.9 (171.9 - 474.2)	-1 (-1.4 to -0.7)	<0.001	105

Gabon	926 (648 - 1272)	301.4 (211.1 - 414.2)	1580 (1024 - 2310)	279.9 (181.5 - 409.5)	-0.2 (-0.4 to 0.1)	0.152	113
**East Asia**							
China	2970859 (1947414 - 3411865)	823 (539.5 - 945.2)	461196 (379915 - 583162)	197.1 (162.3 - 249.2)	-4.5 (-4.8 to -4.1)	<0.001	159
Democratic People's Republic of Korea	28429 (19115 - 40948)	516.6 (347.3 - 744)	18535 (12889 - 27449)	339.8 (236.3 - 503.3)	-1.3 (-1.5 to -1.2)	<0.001	89
Taiwan (Province of China)	16627 (15729 - 17740)	290.8 (275.1 - 310.3)	12988 (11759 - 14216)	369.2 (334.3 - 404.1)	0.8 (0.2 to 1.5)	0.015	81
**Eastern Europe**							
Belarus	13896 (12884 - 14809)	627.6 (581.9 - 668.9)	7892 (6477 - 9449)	555.1 (455.5 - 664.6)	-0.6 (-1.3 to 0)	0.069	45
Estonia	3274 (2979 - 3590)	1002.4 (912 - 1099.2)	921 (778 - 1034)	455.2 (384.6 - 511.1)	-2.7 (-4.6 to -0.9)	0.005	60
Latvia	4832 (4511 - 5145)	895.4 (835.8 - 953.4)	1117 (978 - 1239)	411.8 (360.5 - 456.7)	-2.4 (-2.9 to -2)	<0.001	68
Lithuania	6839 (6426 - 7222)	835.8 (785.4 - 882.7)	2636 (2353 - 2914)	638.7 (570.2 - 706.1)	-0.7 (-2.4 to 1)	0.422	40
Republic of Moldova	5860 (5417 - 6277)	570.2 (527.1 - 610.8)	2092 (1841 - 2353)	383.3 (337.5 - 431.2)	-1.4 (-2.3 to -0.5)	0.002	75
Russian Federation	320191 (314645 - 325322)	1018.1 (1000.4 - 1034.4)	159822 (147644 - 171142)	678.4 (626.7 - 726.4)	-1.6 (-2.5 to -0.7)	<0.001	36
Ukraine	62557 (58763 - 66737)	576.5 (541.5 - 615)	47869 (35034 - 60778)	727.2 (532.3 - 923.4)	0.6 (0 to 1.3)	0.066	**28**
**Eastern Sub-Saharan Africa**							
Burundi	8948 (6025 - 13063)	533.5 (359.2 - 778.8)	13106 (8873 - 20962)	303.6 (205.5 - 485.6)	-1.9 (-2.3 to -1.4)	<0.001	101
Comoros	395 (128 - 638)	256.4 (83.1 - 414.1)	479 (307 - 704)	217.2 (139.4 - 319.5)	-1.3 (-6.4 to 4.1)	0.638	145
Djibouti	257 (150 - 454)	174.6 (101.8 - 309)	653 (353 - 1156)	183.4 (99.3 - 324.7)	0.4 (-0.1 to 0.8)	0.125	167
Eritrea	4569 (3297 - 6138)	405.4 (292.6 - 544.7)	7833 (5153 - 11387)	375.7 (247.2 - 546.3)	-0.3 (-0.6 to 0.1)	0.137	79
Ethiopia	77892 (52850 - 96581)	484.2 (328.5 - 600.3)	89280 (70201 - 108661)	236.7 (186.1 - 288.1)	-2.3 (-2.4 to -2.2)	<0.001	135
Kenya	16044 (12841 - 25011)	202.3 (161.9 - 315.4)	36767 (27524 - 55324)	213.1 (159.5 - 320.6)	0.2 (-0.1 to 0.4)	0.243	151

Madagascar	12938 (9712 - 16443)	337.8 (253.6 - 429.4)	28543 (19239 - 39668)	297.1 (200.2 - 412.8)	-0.4 (-0.5 to -0.3)	<0.001	107
Malawi	11981 (8950 - 16032)	379.1 (283.2 - 507.3)	24694 (17266 - 34402)	347.1 (242.7 - 483.6)	-0.3 (-0.7 to 0.1)	0.147	86
Mozambique	12206 (8376 - 16119)	295.4 (202.7 - 390)	40744 (26827 - 59252)	385 (253.5 - 559.9)	0.9 (0.7 to 1.1)	<0.001	74
Rwanda	12985 (9342 - 16858)	573.5 (412.6 - 744.6)	11782 (7819 - 17417)	273 (181.2 - 403.5)	-2.4 (-2.8 to -1.9)	<0.001	117
Somalia	6395 (3706 - 10927)	251.1 (145.5 - 429)	19469 (11181 - 32794)	267.6 (153.7 - 450.8)	0.2 (0 to 0.4)	0.016	121
South Sudan	5148 (3398 - 7196)	259.4 (171.2 - 362.6)	11342 (7653 - 16118)	333.5 (225 - 473.9)	1 (-0.1 to 2.1)	0.073	93
Uganda	12172 (8194 - 17236)	215.4 (145 - 305.1)	39314 (28117 - 54762)	259.1 (185.3 - 361)	0.5 (0.2 to 0.9)	0.002	126
United Republic of Tanzania	24931 (18915 - 32305)	292.5 (221.9 - 379)	41912 (29060 - 59528)	217.3 (150.7 - 308.7)	-0.9 (-1.1 to -0.8)	<0.001	144
Zambia	9820 (7500 - 12565)	354 (270.4 - 453)	21514 (14313 - 31720)	325.9 (216.8 - 480.5)	-0.2 (-0.6 to 0.1)	0.175	96
**High-income Asia Pacific**							
Brunei Darussalam	183 (118 - 253)	240.7 (154.4 - 331.7)	113 (88 - 146)	106.7 (82.6 - 138.1)	-2.7 (-3.5 to -1.9)	<0.001	189
Japan	116723 (114355 - 119029)	415.6 (407.2 - 423.8)	122371 (120013 - 124343)	696.6 (683.2 - 707.8)	1.8 (0.9 to 2.6)	<0.001	33
Republic of Korea	88277 (75687 - 154159)	671.7 (575.9 - 1173)	44810 (32431 - 49416)	581.4 (420.7 - 641.1)	-0.2 (-1 to 0.7)	0.67	44
Singapore	5039 (4723 - 5348)	611.1 (572.7 - 648.5)	3578 (3321 - 3859)	494.2 (458.7 - 533)	-0.7 (-1.8 to 0.5)	0.237	55
**High-income North America**							
Canada	48138 (45797 - 50398)	825.9 (785.7 - 864.7)	45745 (42682 - 48702)	707.6 (660.2 - 753.3)	-0.5 (-0.9 to -0.1)	0.015	32
Greenland	880 (641 - 1010)	6365.9 (4637.4 - 7303.6)	340 (262 - 402)	3022.7 (2329.8 - 3573.7)	-2.3 (-2.5 to -2.2)	<0.001	**1**
United States of America	377462 (371081 - 384746)	682.2 (670.7 - 695.4)	468512 (450916 - 485523)	723 (695.8 - 749.2)	0.2 (-0.1 to 0.5)	0.247	**30**

**North Africa and Middle East**							
Afghanistan	9521 (6374 - 18281)	281.9 (188.7 - 541.3)	23868 (16135 - 46839)	224.1 (151.5 - 439.8)	-0.6 (-1.2 to 0)	0.048	142
Bahrain	534 (419 - 667)	413.4 (324.3 - 516.1)	792 (568 - 1066)	236 (169.3 - 317.5)	-1.8 (-2.8 to -0.8)	<0.001	137
Egypt	15931 (11593 - 20570)	92.6 (67.4 - 119.6)	21352 (15316 - 27598)	71.8 (51.5 - 92.8)	-0.8 (-1.4 to -0.2)	0.011	196
Iran (Islamic Republic of)	100071 (70331 - 111519)	543 (381.6 - 605.2)	58477 (49984 - 64954)	330.1 (282.2 - 366.7)	-1.6 (-1.9 to -1.3)	<0.001	94
Iraq	22562 (16468 - 29544)	368.3 (268.8 - 482.3)	30151 (21486 - 40991)	240.9 (171.7 - 327.5)	-1.4 (-1.6 to -1.2)	<0.001	132
Jordan	2471 (1848 - 3125)	182.3 (136.3 - 230.5)	2359 (1741 - 3042)	62.3 (46 - 80.3)	-3.3 (-3.9 to -2.8)	<0.001	197
Kuwait	491 (433 - 554)	106.5 (94 - 120.2)	934 (762 - 1144)	112 (91.4 - 137.2)	0.8 (-2.3 to 4)	0.62	187
Lebanon	966 (561 - 1356)	111.3 (64.6 - 156.2)	664 (507 - 854)	56 (42.7 - 72.1)	-2.2 (-2.4 to -2)	<0.001	199
Libya	4251 (2642 - 5633)	288.9 (179.6 - 382.9)	5908 (3773 - 8077)	338.9 (216.4 - 463.3)	0.5 (-0.1 to 1.2)	0.097	90
Morocco	31405 (17799 - 42461)	387.9 (219.9 - 524.5)	21220 (13139 - 32157)	227.1 (140.6 - 344.2)	-1.7 (-1.9 to -1.6)	<0.001	141
Oman	547 (369 - 785)	100.6 (68 - 144.4)	499 (368 - 657)	53.8 (39.7 - 70.8)	-1.8 (-2.5 to -1.2)	<0.001	201
Palestine	449 (318 - 625)	66.1 (46.9 - 92.1)	894 (663 - 1169)	54.1 (40.1 - 70.7)	-0.5 (-0.9 to -0.2)	0.001	200
Qatar	462 (358 - 596)	468.3 (363.3 - 604.2)	1031 (741 - 1429)	231.5 (166.4 - 320.9)	-2.4 (-3.2 to -1.7)	<0.001	140
Saudi Arabia	20941 (14392 - 28064)	415.2 (285.4 - 556.5)	23826 (16986 - 33983)	289.3 (206.3 - 412.7)	-1.2 (-1.4 to -0.9)	<0.001	110
Sudan	31005 (15971 - 44096)	480 (247.3 - 682.7)	47955 (26294 - 75796)	334.1 (183.2 - 528)	-1.2 (-1.4 to -1)	<0.001	92
Syrian Arab Republic	4673 (3386 - 6151)	107.8 (78.1 - 142)	2651 (1921 - 3728)	57.3 (41.5 - 80.6)	-2.1 (-2.7 to -1.4)	<0.001	198
Tunisia	6359 (4128 - 9236)	237.8 (154.4 - 345.4)	4343 (2729 - 6732)	173 (108.7 - 268.1)	-1 (-1.2 to -0.8)	<0.001	171
Türkiye	54484 (34143 - 76886)	296.8 (186 - 418.9)	51668 (35117 - 64930)	268.4 (182.4 - 337.3)	-0.3 (-0.8 to 0.3)	0.394	119
United Arab Emirates	1396 (1006 - 2039)	331.9 (239.1 - 484.6)	2348 (1698 - 3296)	210.5 (152.2 - 295.6)	-1.1 (-3.4 to 1.2)	0.344	153
Yemen	8864 (2386 - 14576)	210.4 (56.6 - 346)	21548 (7232 - 35664)	195.8 (65.7 - 324.1)	-0.3 (-1.3 to 0.8)	0.593	161
**Oceania**							
American Samoa	88 (65 - 114)	584 (432.7 - 760.6)	85 (66 - 108)	606.8 (473 - 773.5)	0.1 (-0.1 to 0.3)	0.354	42
Cook Islands	72 (55 - 91)	1215.8 (936.9 - 1537.9)	29 (23 - 38)	721 (557.7 - 921.5)	-1.7 (-1.9 to -1.5)	<0.001	31

Fiji	2337 (1820 - 2932)	982.5 (765.2 - 1232.5)	1900 (1391 - 2533)	793.8 (581 - 1058)	-0.6 (-1.2 to -0.1)	0.033	**23**
Guam	666 (583 - 767)	1697.7 (1486.4 - 1953.8)	468 (398 - 539)	1302.8 (1106.9 - 1498.9)	-1.1 (-2.7 to 0.5)	0.18	**13**
Kiribati	388 (298 - 485)	1735.2 (1332.5 - 2171.7)	545 (401 - 698)	1540.8 (1135.1 - 1973.4)	-0.4 (-0.4 to -0.3)	<0.001	**8**
Marshall Islands	290 (162 - 384)	1902.9 (1064.4 - 2518.7)	269 (167 - 362)	1622.9 (1009.6 - 2186.6)	-0.7 (-1.1 to -0.3)	<0.001	**5**
Micronesia (Federated States of)	622 (422 - 797)	1809.5 (1229.2 - 2321)	492 (341 - 656)	1569.2 (1088.1 - 2094.6)	-0.5 (-0.5 to -0.4)	<0.001	**6**
Nauru	65 (39 - 84)	2142.7 (1274 - 2789)	68 (40 - 90)	1988.2 (1154.6 - 2639.6)	-0.2 (-0.4 to -0.1)	<0.001	**2**
Niue	8 (6 - 11)	1269.7 (912.2 - 1683.6)	7 (6 - 9)	1806.5 (1430.1 - 2158.1)	1.2 (0.4 to 1.9)	0.001	**4**
Northern Mariana Islands	114 (85 - 153)	898.3 (671.6 - 1206.4)	107 (88 - 126)	936.5 (766.4 - 1097.8)	-0.2 (-1.1 to 0.7)	0.702	**19**
Palau	71 (46 - 104)	1513.1 (985 - 2220.8)	55 (42 - 71)	1562.5 (1201.3 - 2027.1)	0.1 (-0.2 to 0.5)	0.374	**7**
Papua New Guinea	1722 (1017 - 3047)	131.5 (77.7 - 232.7)	4445 (2887 - 10591)	145.6 (94.5 - 346.8)	0.5 (-0.3 to 1.3)	0.208	178
Samoa	753 (560 - 947)	1276.5 (949.3 - 1605)	637 (464 - 839)	1017.4 (741.2 - 1340.8)	-0.7 (-0.8 to -0.6)	<0.001	**16**
Solomon Islands	1731 (683 - 2490)	1500.7 (592.3 - 2158.5)	3151 (2140 - 4244)	1495.4 (1015.6 - 2014.3)	-0.1 (-0.7 to 0.4)	0.627	**9**
Tokelau	6 (4 - 8)	1358.3 (967.5 - 1786.6)	7 (6 - 9)	1913.7 (1541.8 - 2366.5)	1.5 (0.4 to 2.6)	0.006	**3**
Tonga	114 (90 - 145)	342.6 (269.1 - 434.4)	119 (84 - 164)	381.8 (268.7 - 528.1)	0.3 (0 to 0.6)	0.046	77

Tuvalu	48 (30 - 61)	1939.4 (1238.1 - 2484.9)	46 (34 - 60)	1327.2 (981 - 1710.3)	-1.2 (-1.3 to -1.1)	<0.001	**12**
Vanuatu	658 (401 - 875)	1389.7 (846.5 - 1848.2)	1313 (966 - 1682)	1400.3 (1030.4 - 1793.7)	0 (-0.3 to 0.3)	0.94	**11**
**South Asia**							
Bangladesh	287179 (207243 - 355923)	822.9 (593.8 - 1019.8)	140800 (102841 - 203816)	307.5 (224.6 - 445.1)	-3 (-3.6 to -2.4)	<0.001	100
Bhutan	740 (470 - 1135)	325.3 (206.7 - 498.8)	476 (288 - 1025)	232.7 (140.5 - 500.5)	-1.1 (-1.3 to -0.9)	<0.001	139
India	3485621 (2634271 - 3967233)	1352.2 (1022 - 1539.1)	3123647 (2669898 - 3495433)	785.2 (671.1 - 878.6)	-1.8 (-2.8 to -0.7)	0.001	**24**
Nepal	64951 (42252 - 88350)	1090.4 (709.3 - 1483.3)	78867 (56877 - 108910)	834.5 (601.8 - 1152.3)	-0.9 (-1 to -0.7)	<0.001	**21**
Pakistan	146204 (102065 - 201393)	410.5 (286.5 - 565.4)	310995 (204513 - 458292)	428.4 (281.7 - 631.3)	0.2 (0 to 0.3)	0.02	63
**Southern Latin America**							
Argentina	31400 (29266 - 33420)	360.4 (335.9 - 383.6)	77918 (71955 - 83861)	728.8 (673 - 784.3)	2.2 (1.4 to 3.1)	<0.001	**27**
Chile	37867 (35043 - 40855)	1007.8 (932.6 - 1087.3)	19527 (18036 - 21102)	497.6 (459.6 - 537.7)	-2.2 (-2.7 to -1.7)	<0.001	53
Uruguay	4265 (3955 - 4557)	556.4 (516 - 594.6)	7528 (6876 - 8230)	1046.4 (955.6 - 1143.9)	2.1 (1.5 to 2.7)	<0.001	**15**
**Southeast Asia**							
Cambodia	10192 (7529 - 13333)	317.8 (234.8 - 415.7)	10008 (6552 - 14744)	216.2 (141.5 - 318.4)	-1.2 (-1.4 to -1.1)	<0.001	146
Indonesia	72365 (53323 - 86803)	121.7 (89.7 - 146)	73752 (59165 - 94694)	106.7 (85.6 - 137)	-0.4 (-0.6 to -0.2)	<0.001	190
Lao People's Democratic Republic	8328 (4552 - 11617)	645.6 (352.9 - 900.6)	7833 (5383 - 11052)	375.4 (258 - 529.6)	-1.7 (-1.9 to -1.5)	<0.001	80
Malaysia	14440 (11286 - 18683)	268.8 (210.1 - 347.8)	15308 (11726 - 20480)	189.2 (144.9 - 253.1)	-1.5 (-2.1 to -0.9)	<0.001	164

Maldives	148 (94 - 193)	207.3 (131.4 - 268.8)	96 (72 - 131)	95.8 (71.8 - 131.4)	-2.3 (-3 to -1.6)	<0.001	192
Mauritius	2665 (2488 - 2836)	821.2 (766.6 - 874)	1334 (1160 - 1496)	516.2 (448.9 - 579.1)	-1.4 (-2 to -0.8)	<0.001	52
Myanmar	42077 (24693 - 59964)	322.1 (189 - 459)	30031 (19865 - 41960)	197 (130.3 - 275.3)	-1.6 (-1.9 to -1.4)	<0.001	160
Philippines	58803 (40840 - 65393)	287.2 (199.4 - 319.3)	76762 (52377 - 91715)	236.2 (161.2 - 282.2)	-0.6 (-0.8 to -0.4)	<0.001	136
Seychelles	74 (59 - 91)	330.5 (266.3 - 406.7)	18 (13 - 24)	83.6 (58.2 - 111.2)	-3.4 (-4.9 to -1.8)	<0.001	195
Sri Lanka	125274 (102387 - 142824)	2418.8 (1976.9 - 2757.7)	42687 (31435 - 56291)	815.5 (600.6 - 1075.5)	-3.5 (-5.5 to -1.3)	0.001	**22**
Thailand	118688 (91730 - 154953)	662.4 (511.9 - 864.8)	52313 (39107 - 71063)	445.1 (332.7 - 604.6)	-1.3 (-1.8 to -0.7)	<0.001	61
Timor-Leste	974 (648 - 1343)	423.3 (281.6 - 583.6)	1476 (1031 - 2033)	311.9 (217.7 - 429.4)	-1 (-2.5 to 0.6)	0.214	98
Viet Nam	76023 (50964 - 102877)	352.9 (236.6 - 477.5)	56824 (40867 - 76187)	267.5 (192.4 - 358.7)	-0.9 (-1 to -0.8)	<0.001	122
**Southern Sub-Saharan Africa**							
Botswana	2899 (1894 - 4158)	642.8 (419.9 - 921.8)	2984 (2012 - 4321)	456.4 (307.7 - 660.9)	-1.1 (-1.3 to -0.9)	<0.001	59
Eswatini	1601 (1174 - 2141)	581.3 (426.2 - 777)	3460 (2170 - 4973)	940.7 (589.8 - 1352)	1.6 (1.4 to 1.8)	<0.001	**18**
Lesotho	2138 (1420 - 3481)	447.6 (297.3 - 728.5)	6145 (4208 - 8201)	1004.1 (687.6 - 1340.1)	2.7 (2.3 to 3.1)	<0.001	**17**
Namibia	2480 (1846 - 3286)	522.5 (389.1 - 692.4)	3990 (2696 - 6034)	535.1 (361.6 - 809.3)	0.1 (-0.3 to 0.6)	0.564	48
South Africa	73083 (59453 - 87748)	618.7 (503.3 - 742.8)	94560 (79618 - 111413)	659.8 (555.5 - 777.4)	0.2 (-0.5 to 0.9)	0.618	38
Zimbabwe	15303 (9462 - 24085)	426.1 (263.4 - 670.6)	47672 (30400 - 65974)	934.2 (595.7 - 1292.8)	2.6 (2 to 3.2)	<0.001	**20**
**Tropical Latin America**							
Brazil	128829 (123419 - 134713)	276.3 (264.7 - 288.9)	204450 (195972 - 213122)	420.8 (403.3 - 438.6)	1.5 (0.8 to 2.1)	<0.001	65

Paraguay	2777 (2342 - 3315)	225.1 (189.9 - 268.7)	7980 (5373 - 10073)	400.9 (269.9 - 506.1)	1.9 (1 to 2.9)	<0.001	70
**Western Europe**							
Andorra	45 (31 - 61)	366.5 (252.6 - 502.3)	28 (20 - 39)	214.9 (153.3 - 296.3)	-1.7 (-2.1 to -1.3)	<0.001	149
Austria	13177 (12358 - 14062)	810.2 (759.9 - 864.6)	5794 (5245 - 6402)	410.1 (371.3 - 453.2)	-2.3 (-2.6 to -2)	<0.001	69
Belgium	11863 (11176 - 12548)	589.3 (555.2 - 623.3)	7608 (6835 - 8303)	387.3 (347.9 - 422.7)	-1.4 (-2.1 to -0.8)	<0.001	72
Cyprus	342 (253 - 457)	179.6 (132.9 - 240.4)	271 (191 - 354)	125 (88.1 - 163.3)	-1.2 (-2.1 to -0.3)	0.011	185
Denmark	5820 (5485 - 6187)	532.3 (501.6 - 565.9)	2652 (2381 - 2921)	252.5 (226.8 - 278.2)	-2.3 (-3 to -1.6)	<0.001	129
Finland	11840 (11155 - 12486)	1216.3 (1145.9 - 1282.7)	5557 (5089 - 6066)	606 (554.9 - 661.5)	-2 (-3.2 to -0.7)	0.002	43
France	72417 (68426 - 76608)	572.9 (541.3 - 606)	29856 (26787 - 32775)	244.3 (219.2 - 268.2)	-2.6 (-3.1 to -2.1)	<0.001	131
Germany	81314 (75978 - 86313)	545.9 (510.1 - 579.5)	38288 (34885 - 41847)	303.3 (276.3 - 331.5)	-1.8 (-2.5 to -1.2)	<0.001	102
Greece	3281 (2978 - 3633)	140 (127.1 - 155)	1724 (1488 - 1988)	111.7 (96.4 - 128.8)	-0.7 (-1.9 to 0.6)	0.296	188
Iceland	431 (392 - 473)	681.4 (619.6 - 748.6)	252 (221 - 283)	369.2 (323.6 - 414.8)	-2 (-3.7 to -0.2)	0.027	82
Ireland	4442 (4082 - 4889)	456.1 (419.1 - 502)	2636 (2235 - 3082)	268.1 (227.3 - 313.4)	-1.8 (-2.5 to -1.1)	<0.001	120
Israel	4182 (3899 - 4478)	307.1 (286.3 - 328.8)	3784 (3374 - 4205)	167.9 (149.7 - 186.6)	-1.8 (-3.1 to -0.5)	0.006	172
Italy	28955 (27979 - 30007)	231.3 (223.5 - 239.7)	12626 (11960 - 13334)	145.2 (137.5 - 153.3)	-1.7 (-2.3 to -1)	<0.001	179
Luxembourg	553 (484 - 629)	765.7 (670 - 870.1)	223 (191 - 255)	208.3 (178.2 - 238.2)	-3.9 (-5.7 to -2)	<0.001	156
Malta	168 (150 - 190)	205.7 (183 - 232.2)	106 (92 - 121)	166 (144.3 - 190.3)	-0.7 (-2.1 to 0.8)	0.371	174
Monaco	27 (20 - 34)	612.9 (466.8 - 783.1)	24 (19 - 33)	464.9 (353.6 - 630.6)	-0.9 (-1 to -0.8)	<0.001	58
Netherlands	13294 (12528 - 14050)	405.8 (382.4 - 428.9)	10318 (9570 - 11109)	342.8 (318 - 369.1)	-0.6 (-2 to 0.9)	0.421	87
Norway	8251 (7958 - 8568)	900.1 (868.1 - 934.6)	5128 (4910 - 5348)	519.3 (497.2 - 541.6)	-1.7 (-3.5 to 0.1)	0.068	51
Portugal	14797 (13689 - 15772)	593.1 (548.7 - 632.2)	2979 (2610 - 3365)	181.6 (159.1 - 205.1)	-3.6 (-4.8 to -2.4)	<0.001	168
San Marino	22 (16 - 28)	392.3 (293.5 - 494.3)	13 (9 - 17)	232.7 (168.9 - 309.6)	-1.8 (-1.9 to -1.6)	<0.001	138
Spain	27469 (25701 - 29400)	282 (263.9 - 301.9)	11621 (10416 - 12780)	166.1 (148.9 - 182.7)	-1.8 (-2.9 to -0.7)	0.001	173
Sweden	11327 (10830 - 11884)	684.2 (654.2 - 717.8)	8297 (7587 - 9151)	467.6 (427.6 - 515.7)	-1.4 (-2.7 to -0.1)	0.036	57
Switzerland	12824 (12153 - 13538)	964.5 (914 - 1018.2)	4269 (3878 - 4599)	314 (285.2 - 338.2)	-3.4 (-4.4 to -2.3)	<0.001	97

United Kingdom	59062 (58060 - 60036)	498.3 (489.9 - 506.5)	39581 (38104 - 40814)	327.2 (315 - 337.4)	-1.2 (-2 to -0.5)	0.002	95
**Western Sub-Saharan Africa**							
Benin	3211 (2364 - 4276)	222.5 (163.9 - 296.4)	9435 (5924 - 15338)	215.5 (135.3 - 350.3)	-0.1 (-0.3 to 0)	0.071	147
Burkina Faso	7651 (5779 - 10275)	261 (197.2 - 350.6)	17386 (12173 - 23907)	239.2 (167.4 - 328.9)	-0.3 (-0.6 to 0)	0.048	134
Cabo Verde	413 (300 - 518)	361.9 (263.2 - 453.8)	598 (426 - 794)	398.5 (283.8 - 529.4)	0.4 (-0.2 to 1.1)	0.193	71
Cameroon	7801 (5634 - 10235)	242 (174.7 - 317.4)	28911 (17372 - 43118)	278.4 (167.3 - 415.2)	0.4 (0.2 to 0.7)	<0.001	114
Chad	3123 (2075 - 4793)	171.1 (113.6 - 262.5)	14438 (9237 - 21841)	247.5 (158.3 - 374.4)	1.3 (1 to 1.6)	<0.001	130
Côte d'Ivoire	10408 (7536 - 14734)	272.3 (197.2 - 385.5)	22777 (14429 - 35408)	264.8 (167.7 - 411.6)	-0.1 (-0.5 to 0.4)	0.723	125
Gambia	555 (314 - 924)	177.3 (100.3 - 295.2)	1949 (1196 - 3154)	239.3 (146.8 - 387.2)	1 (-0.7 to 2.7)	0.253	133
Ghana	8628 (6245 - 11868)	183.3 (132.6 - 252.1)	21943 (15508 - 31782)	208.9 (147.7 - 302.6)	0.4 (0.2 to 0.7)	<0.001	155
Guinea	2776 (1850 - 4211)	165.7 (110.4 - 251.4)	9166 (5390 - 15053)	211.6 (124.4 - 347.6)	0.8 (0.6 to 0.9)	<0.001	152
Guinea-Bissau	1172 (750 - 1703)	362.5 (232 - 526.8)	2440 (1711 - 3394)	363 (254.5 - 505)	0 (-0.2 to 0.2)	0.928	83
Liberia	1661 (1153 - 2262)	228.5 (158.6 - 311.1)	4833 (2887 - 7393)	268.7 (160.5 - 411.1)	0.5 (-0.4 to 1.4)	0.291	118
Mali	4742 (3167 - 6954)	185.9 (124.1 - 272.6)	14685 (9722 - 22117)	183.7 (121.6 - 276.7)	0 (-0.4 to 0.4)	0.993	166
Mauritania	994 (635 - 1559)	156.2 (99.7 - 244.9)	1830 (1128 - 3074)	127.4 (78.6 - 214)	-0.7 (-0.9 to -0.4)	<0.001	184
Niger	4325 (2726 - 6838)	176.7 (111.4 - 279.4)	13609 (7829 - 25262)	161.6 (92.9 - 299.9)	-0.3 (-0.6 to 0.1)	0.167	175
Nigeria	49079 (35006 - 63064)	173.9 (124 - 223.4)	119301 (83102 - 164729)	153.1 (106.6 - 211.4)	-0.4 (-0.5 to -0.3)	<0.001	176
Sao Tome and Principe	13 (6 - 20)	31.4 (15.5 - 49.3)	31 (18 - 45)	43.3 (26.1 - 64)	1.1 (0.5 to 1.8)	0.001	203
Senegal	7109 (5223 - 9393)	293.5 (215.6 - 387.7)	13992 (9842 - 20595)	267.4 (188.1 - 393.7)	-0.1 (-0.3 to 0.1)	0.296	123
Sierra Leone	1945 (1150 - 3177)	159.9 (94.6 - 261.2)	6002 (3276 - 10269)	206.3 (112.6 - 352.9)	0.8 (0.3 to 1.3)	0.002	157
Togo	2722 (1944 - 3711)	227.8 (162.7 - 310.6)	7193 (4569 - 10797)	274.7 (174.5 - 412.3)	0.6 (0.4 to 0.9)	<0.001	115

Note: Order is ranked according to the highest to lowest suicide mortality for 10-24 years population in each country around the world in 2021. The top 30 countries in the world are marked in red.

Table S4. The trends in rate of suicide mortality in 204 countries by Joinpoint regression

location	Year	Estimate (95%CI)	P value
Afghanistan	1990 - 1995	4.3 (3.2 to 5.3)	<0.001
	1995 - 1999	-1.7 (-3.7 to 0.4)	0.103
	1999 - 2002	-7.7 (-11.5 to -3.8)	0.001
	2002 - 2005	1.4 (-2.5 to 5.4)	0.469
	2005 - 2009	-3.1 (-5.1 to -1.1)	0.005
	2009 - 2021	0.1 (-0.2 to 0.3)	0.548
Albania	1990 - 1998	2.9 (1.4 to 4.5)	0.001
	1998 - 2018	-2.9 (-3.4 to -2.3)	<0.001
	2018 - 2021	-15.7 (-25.6 to -4.6)	0.009
Algeria	1990 - 1992	-3.1 (-5 to -1.2)	0.004
	1992 - 2001	0 (-0.2 to 0.2)	0.812
	2001 - 2005	-3.5 (-4.5 to -2.5)	<0.001
	2005 - 2015	-1.9 (-2.1 to -1.7)	<0.001
	2015 - 2021	-2.9 (-3.2 to -2.6)	<0.001
American Samoa	1990 - 2008	-0.8 (-1 to -0.7)	<0.001
	2008 - 2015	2.2 (1.5 to 2.9)	<0.001
	2015 - 2021	0.5 (-0.3 to 1.3)	0.182
Andorra	1990 - 1992	1 (-1.8 to 3.9)	0.464
	1992 - 2000	-1.9 (-2.3 to -1.6)	<0.001
	2000 - 2003	4.2 (1.4 to 7.1)	0.006
	2003 - 2006	0.6 (-2.3 to 3.5)	0.681
	2006 - 2011	-8.1 (-8.8 to -7.3)	<0.001
	2011 - 2021	-1.2 (-1.3 to -1)	<0.001
Angola	1990 - 1994	0.9 (-1.3 to 3.2)	0.399
	1994 - 1997	-7.5 (-13.7 to -0.8)	0.031
	1997 - 2000	3.5 (-3.3 to 10.8)	0.304
	2000 - 2021	-0.9 (-1 to -0.7)	<0.001
Antigua and Barbuda	1990 - 1998	5.3 (1.8 to 9)	0.004
	1998 - 2007	-4.9 (-8.2 to -1.6)	0.006
	2007 - 2021	-0.2 (-1.8 to 1.5)	0.844
Argentina	1990 - 2002	7.3 (6.8 to 7.7)	<0.001
	2002 - 2005	-3.6 (-9.3 to 2.4)	0.222
	2005 - 2012	2 (0.9 to 3)	0.001
	2012 - 2019	-0.1 (-1.2 to 1.1)	0.897
	2019 - 2021	-8.2 (-15.3 to -0.5)	0.038
Armenia	1990 - 1994	11.6 (6.7 to 16.8)	<0.001
	1994 - 2003	-10 (-11.8 to -8.1)	<0.001
	2003 - 2006	33.4 (14.9 to 54.8)	0.001
	2006 - 2010	3.8 (-2.8 to 10.8)	0.245
	2010 - 2021	-5 (-6 to -3.9)	<0.001
Australia	1990 - 1994	-1.7 (-3.2 to -0.1)	0.034
	1994 - 1997	4.4 (-0.4 to 9.5)	0.069

	1997 - 2003	-7.6 (-8.7 to -6.5)	<0.001
	2003 - 2009	-0.9 (-2 to 0.3)	0.128
	2009 - 2019	3 (2.5 to 3.4)	<0.001
	2019 - 2021	-3 (-9.2 to 3.7)	0.35
Austria	1990 - 2006	-2.9 (-3.3 to -2.4)	<0.001
	2006 - 2021	-1.7 (-2.2 to -1.2)	<0.001
Azerbaijan	1990 - 1993	25.5 (19.7 to 31.6)	<0.001
	1993 - 1997	-18.9 (-24.1 to -13.3)	<0.001
	1997 - 2006	2.8 (1.8 to 3.7)	<0.001
	2006 - 2017	-0.3 (-1 to 0.5)	0.445
	2017 - 2021	-8.4 (-11.9 to -4.8)	<0.001
Bahamas	1990 - 1999	-0.3 (-0.9 to 0.4)	0.396
	1999 - 2005	-3.4 (-4.9 to -1.9)	<0.001
	2005 - 2011	4.5 (2.9 to 6.2)	<0.001
	2011 - 2021	-1.4 (-2.3 to -0.6)	0.002
Bahrain	1990 - 1995	-2.5 (-4.3 to -0.6)	0.014
	1995 - 2001	6.3 (4.3 to 8.3)	<0.001
	2001 - 2017	-4.6 (-5 to -4.2)	<0.001
	2017 - 2021	-0.5 (-3.9 to 3.1)	0.784
Bangladesh	1990 - 1996	-1.8 (-2.5 to -1)	<0.001
	1996 - 2004	-3.3 (-3.8 to -2.7)	<0.001
	2004 - 2007	-0.6 (-4 to 2.9)	0.71
	2007 - 2010	-3.6 (-6.8 to -0.4)	0.03
	2010 - 2013	-8.7 (-12.2 to -5)	<0.001
	2013 - 2021	-2.2 (-2.7 to -1.6)	<0.001
Barbados	1990 - 1997	0.5 (-0.9 to 2)	0.455
	1997 - 2007	-4.7 (-5.7 to -3.7)	<0.001
	2007 - 2021	0.5 (-0.2 to 1.3)	0.164
Belarus	1990 - 1993	10.2 (6.7 to 13.8)	<0.001
	1993 - 2000	5.1 (4.1 to 6.2)	<0.001
	2000 - 2011	-0.5 (-1 to 0)	0.044
	2011 - 2017	-10.7 (-12.2 to -9.2)	<0.001
	2017 - 2021	-2.7 (-6.1 to 1)	0.138
Belgium	1990 - 1995	4.8 (2.7 to 7.1)	<0.001
	1995 - 2006	-3.8 (-4.5 to -3.1)	<0.001
	2006 - 2011	1.1 (-1.8 to 4.2)	0.437
	2011 - 2021	-3.2 (-4 to -2.3)	<0.001
Belize	1990 - 1998	7.6 (6.1 to 9.2)	<0.001
	1998 - 2021	-2.1 (-2.5 to -1.8)	<0.001
Benin	1990 - 1999	1.1 (0.9 to 1.2)	<0.001
	1999 - 2006	-1.9 (-2.2 to -1.5)	<0.001
	2006 - 2013	1.8 (1.4 to 2.2)	<0.001
	2013 - 2021	-1.5 (-1.7 to -1.2)	<0.001
Bermuda	1990 - 2006	-4.8 (-5.1 to -4.4)	<0.001

	2006 - 2011	0.7 (-2.4 to 3.9)	0.641
	2011 - 2021	-2.7 (-3.7 to -1.8)	<0.001
Bhutan	1990 - 1997	0 (-0.6 to 0.5)	0.896
	1997 - 2014	-1 (-1.2 to -0.8)	<0.001
	2014 - 2021	-2.3 (-3.1 to -1.6)	<0.001
Bolivia (Plurinational State of)	1990 - 1994	0.2 (0 to 0.5)	0.088
	1994 - 2000	0.8 (0.6 to 1)	<0.001
	2000 - 2006	-0.6 (-0.8 to -0.4)	<0.001
	2006 - 2009	-0.1 (-1.1 to 1)	0.894
	2009 - 2018	-1.5 (-1.7 to -1.4)	<0.001
	2018 - 2021	-2.3 (-2.9 to -1.7)	<0.001
Bosnia and Herzegovina	1990 - 1992	14.5 (3.5 to 26.7)	0.012
	1992 - 1997	-8.7 (-12.7 to -4.4)	0.001
	1997 - 2013	-2.1 (-2.6 to -1.6)	<0.001
	2013 - 2018	1.4 (-2 to 5)	0.401
	2018 - 2021	-12.4 (-17.7 to -6.7)	<0.001
Botswana	1990 - 1994	3 (2.3 to 3.7)	<0.001
	1994 - 2002	0.1 (-0.2 to 0.5)	0.341
	2002 - 2006	-5.1 (-6.1 to -4)	<0.001
	2006 - 2011	2.1 (1.5 to 2.7)	<0.001
	2011 - 2014	-2.9 (-4.5 to -1.2)	0.003
	2014 - 2018	-5.3 (-6.1 to -4.5)	<0.001
	2018 - 2021	-1.7 (-2.7 to -0.7)	0.004
Brazil	1990 - 1994	2.5 (0.8 to 4.2)	0.007
	1994 - 1997	-1.7 (-6.6 to 3.4)	0.489
	1997 - 2013	0.4 (0.2 to 0.6)	<0.001
	2013 - 2019	6.6 (5.5 to 7.8)	<0.001
	2019 - 2021	-2.3 (-7 to 2.7)	0.344
Brunei Darussalam	1990 - 2001	-4.5 (-6 to -3)	<0.001
	2001 - 2013	-0.3 (-1.5 to 0.9)	0.596
	2013 - 2021	-3.7 (-5.7 to -1.7)	0.001
Bulgaria	1990 - 1997	2.9 (1.6 to 4.2)	<0.001
	1997 - 2003	-6.1 (-8.1 to -4.2)	<0.001
	2003 - 2012	-1 (-2.1 to 0.1)	0.076
	2012 - 2021	-5.1 (-6.2 to -4)	<0.001
Burkina Faso	1990 - 1992	-1.9 (-4.9 to 1.1)	0.197
	1992 - 2000	0.1 (-0.3 to 0.5)	0.695
	2000 - 2010	-1 (-1.3 to -0.7)	<0.001
	2010 - 2016	2.7 (1.9 to 3.4)	<0.001
	2016 - 2021	-2.2 (-3 to -1.5)	<0.001
Burundi	1990 - 1996	-0.9 (-1.5 to -0.2)	0.013
	1996 - 2008	-3 (-3.2 to -2.7)	<0.001

	2008 - 2011	0.6 (-4.1 to 5.4)	0.808
	2011 - 2021	-1.9 (-2.2 to -1.5)	<0.001
Cabo Verde	1990 - 2005	0.9 (0.6 to 1.2)	<0.001
	2005 - 2008	-3.3 (-9 to 2.8)	0.269
	2008 - 2016	-0.1 (-0.9 to 0.8)	0.897
	2016 - 2021	2.2 (0.6 to 3.8)	0.008
Cambodia	1990 - 1998	-0.9 (-1.2 to -0.6)	<0.001
	1998 - 2006	-1.9 (-2.2 to -1.5)	<0.001
	2006 - 2021	-1 (-1.2 to -0.9)	<0.001
Cameroon	1990 - 1992	4 (2.6 to 5.4)	<0.001
	1992 - 2000	2.6 (2.4 to 2.8)	<0.001
	2000 - 2006	-0.1 (-0.4 to 0.3)	0.726
	2006 - 2010	1.3 (0.5 to 2.1)	0.003
	2010 - 2014	-0.6 (-1.4 to 0.2)	0.141
	2014 - 2021	-2.3 (-2.5 to -2.1)	<0.001
Canada	1990 - 1995	-0.2 (-1.1 to 0.7)	0.661
	1995 - 2007	-2.7 (-2.9 to -2.4)	<0.001
	2007 - 2013	1 (0.1 to 1.9)	0.031
	2013 - 2018	3.4 (2.1 to 4.7)	<0.001
	2018 - 2021	-1.2 (-3.8 to 1.6)	0.376
Central African Republic	1990 - 2021	-0.4 (-0.5 to -0.3)	<0.001
Chad	1990 - 1995	1.4 (0.2 to 2.6)	0.025
	1995 - 2001	4.8 (3.6 to 6)	<0.001
	2001 - 2021	0.2 (0.1 to 0.3)	0.007
Chile	1990 - 2003	-4.6 (-5 to -4.2)	<0.001
	2003 - 2009	4.7 (3.3 to 6.1)	<0.001
	2009 - 2013	-7.3 (-10.1 to -4.4)	<0.001
	2013 - 2021	-0.7 (-1.6 to 0.1)	0.077
China	1990 - 1993	-1.4 (-3.1 to 0.3)	0.094
	1993 - 2001	-6.6 (-6.9 to -6.2)	<0.001
	2001 - 2004	-5.3 (-6.9 to -3.6)	<0.001
	2004 - 2007	-7.4 (-8.6 to -6.2)	<0.001
	2007 - 2010	-2.4 (-4.1 to -0.7)	0.011
	2010 - 2013	-7.1 (-9.3 to -4.9)	<0.001
	2013 - 2021	-1.8 (-2.1 to -1.5)	<0.001
Colombia	1990 - 1993	1.7 (-1.9 to 5.3)	0.335
	1993 - 1996	13.4 (5.8 to 21.6)	0.002
	1996 - 2008	0 (-0.5 to 0.5)	0.997
	2008 - 2014	-2.4 (-3.7 to -1)	0.002
	2014 - 2018	8.9 (5.3 to 12.5)	<0.001
	2018 - 2021	-3.8 (-9.1 to 1.7)	0.157
Comoros	1990 - 2008	-3 (-4 to -2)	<0.001
	2008 - 2011	-21.3 (-47.3 to 17.6)	0.229

	2011 - 2014	36.8 (-10 to 107.9)	0.135
	2014 - 2021	-0.8 (-4.3 to 2.7)	0.629
Congo	1990 - 1997	3.1 (1.9 to 4.4)	<0.001
	1997 - 2002	-4.2 (-7 to -1.4)	0.006
	2002 - 2006	1 (-3.1 to 5.2)	0.618
	2006 - 2021	-1.1 (-1.5 to -0.7)	<0.001
Cook Islands	1990 - 1997	-2.9 (-3 to -2.7)	<0.001
	1997 - 2000	-1.7 (-2.8 to -0.6)	0.006
	2000 - 2006	-3.3 (-3.5 to -3.1)	<0.001
	2006 - 2009	-1.2 (-2.2 to -0.1)	0.029
	2009 - 2012	1.4 (0.3 to 2.5)	0.019
	2012 - 2015	0.4 (-0.8 to 1.6)	0.522
	2015 - 2021	-1.2 (-1.4 to -0.9)	<0.001
Costa Rica	1990 - 1992	-4.3 (-19.5 to 13.7)	0.599
	1992 - 1996	10.4 (1.6 to 20)	0.022
	1996 - 2004	1.6 (-0.4 to 3.7)	0.103
	2004 - 2008	-4.3 (-11.1 to 3.1)	0.23
	2008 - 2021	2.5 (1.7 to 3.3)	<0.001
Côte d'Ivoire	1990 - 1995	2.8 (2.1 to 3.4)	<0.001
	1995 - 1999	1.3 (-0.1 to 2.7)	0.062
	1999 - 2006	-0.6 (-1.1 to -0.1)	0.016
	2006 - 2011	-2.1 (-3 to -1.2)	<0.001
	2011 - 2014	3.8 (0.9 to 6.7)	0.014
	2014 - 2017	-1.6 (-4.3 to 1.2)	0.227
	2017 - 2021	-3 (-4.1 to -1.9)	<0.001
Croatia	1990 - 1992	17 (5.4 to 29.8)	0.005
	1992 - 2005	-3.6 (-4.2 to -2.9)	<0.001
	2005 - 2014	-2 (-3.2 to -0.7)	0.003
	2014 - 2021	-5.9 (-7.9 to -3.8)	<0.001
Cuba	1990 - 1992	-0.3 (-9.2 to 9.5)	0.948
	1992 - 1999	-7.5 (-9.1 to -5.8)	<0.001
	1999 - 2002	-13.8 (-23.3 to -3.2)	0.016
	2002 - 2007	-5.7 (-9.6 to -1.8)	0.008
	2007 - 2010	4.8 (-7.8 to 19)	0.447
	2010 - 2021	-0.8 (-1.8 to 0.3)	0.146
Cyprus	1990 - 1997	3.8 (2.1 to 5.5)	<0.001
	1997 - 2007	-1.4 (-2.4 to -0.5)	0.006
	2007 - 2013	-3.9 (-6 to -1.9)	0.001
	2013 - 2017	0.9 (-3.6 to 5.7)	0.686
	2017 - 2021	-6.2 (-9.5 to -2.9)	0.001
Czechia	1990 - 1994	3.5 (0.4 to 6.8)	0.031
	1994 - 2005	-1.7 (-2.5 to -1)	<0.001
	2005 - 2012	0.7 (-0.9 to 2.3)	0.35
	2012 - 2015	-7.4 (-16.1 to 2.2)	0.118

	2015 - 2018	0.5 (-9 to 11.1)	0.912
	2018 - 2021	-12.7 (-18.3 to -6.8)	0.001
Democratic People's Republic of Korea	1990 - 1994	-1 (-1.4 to -0.7)	<0.001
	1994 - 2000	-1.9 (-2.1 to -1.6)	<0.001
	2000 - 2005	-3 (-3.4 to -2.7)	<0.001
	2005 - 2010	1 (0.7 to 1.3)	<0.001
	2010 - 2014	-2.5 (-3 to -2)	<0.001
	2014 - 2021	-0.8 (-0.9 to -0.6)	<0.001
Democratic Republic of the Congo	1990 - 1994	-2.2 (-3.6 to -0.8)	0.004
	1994 - 2009	-0.2 (-0.5 to 0)	0.046
	2009 - 2015	1.3 (0.1 to 2.4)	0.034
	2015 - 2021	-0.6 (-1.5 to 0.3)	0.205
Denmark	1990 - 2000	-1.9 (-2.7 to -1)	<0.001
	2000 - 2007	-5.4 (-7.3 to -3.6)	<0.001
	2007 - 2021	-1.6 (-2.2 to -0.9)	<0.001
Djibouti	1990 - 2001	3 (1.8 to 4.1)	<0.001
	2001 - 2021	-1 (-1.4 to -0.5)	<0.001
Dominica	1990 - 1993	0.7 (-0.1 to 1.5)	0.096
	1993 - 1996	-1.1 (-2.6 to 0.5)	0.177
	1996 - 2005	-2.1 (-2.3 to -1.9)	<0.001
	2005 - 2013	1.9 (1.7 to 2.1)	<0.001
	2013 - 2021	0.8 (0.6 to 1)	<0.001
Dominican Republic	1990 - 2011	0.2 (0 to 0.5)	0.053
	2011 - 2017	6.5 (4 to 9.1)	<0.001
	2017 - 2021	-9.7 (-13.2 to -6)	<0.001
Ecuador	1990 - 2002	3.8 (3 to 4.7)	<0.001
	2002 - 2005	8.7 (-9.8 to 31.1)	0.362
	2005 - 2019	-0.6 (-1.4 to 0.3)	0.176
	2019 - 2021	-11.3 (-33.6 to 18.5)	0.399
Egypt	1990 - 1992	-7.3 (-11.9 to -2.4)	0.007
	1992 - 1995	2.3 (-2.5 to 7.4)	0.326
	1995 - 2005	-0.8 (-1.2 to -0.3)	0.002
	2005 - 2010	1.9 (0.3 to 3.5)	0.022
	2010 - 2015	-0.2 (-1.7 to 1.3)	0.755
	2015 - 2021	-2.5 (-3.2 to -1.7)	<0.001
El Salvador	1990 - 2013	-2.4 (-2.7 to -2.1)	<0.001
	2013 - 2016	10.4 (-6.7 to 30.6)	0.237
	2016 - 2021	-8 (-12.4 to -3.3)	0.002
Equatorial Guinea	1990 - 1996	-1.7 (-2.5 to -0.9)	<0.001
	1996 - 2002	-4.8 (-5.9 to -3.6)	<0.001
	2002 - 2014	-0.7 (-1.2 to -0.3)	0.002
	2014 - 2021	2.6 (1.7 to 3.6)	<0.001

Eritrea	1990 - 1993	-0.5 (-1.5 to 0.5)	0.325
	1993 - 1996	2.8 (0.4 to 5.2)	0.023
	1996 - 2001	-1.4 (-2.1 to -0.6)	0.001
	2001 - 2004	1.2 (-1.4 to 3.9)	0.342
	2004 - 2021	-0.6 (-0.7 to -0.5)	<0.001
Estonia	1990 - 1994	6.2 (2.2 to 10.3)	0.004
	1994 - 2008	-2.7 (-3.4 to -2.1)	<0.001
	2008 - 2011	-8.7 (-17.9 to 1.6)	0.089
	2011 - 2014	0.2 (-10 to 11.4)	0.974
	2014 - 2017	-12.4 (-22.6 to -0.8)	0.038
	2017 - 2021	-1 (-5.6 to 3.9)	0.676
Eswatini	1990 - 1994	1.6 (1 to 2.2)	<0.001
	1994 - 1998	5.8 (4.8 to 6.8)	<0.001
	1998 - 2003	9 (8.4 to 9.7)	<0.001
	2003 - 2009	0.4 (-0.1 to 0.8)	0.115
	2009 - 2021	-1.9 (-2.1 to -1.8)	<0.001
Ethiopia	1990 - 1995	-1.3 (-1.7 to -1)	<0.001
	1995 - 1998	-2.9 (-4.1 to -1.8)	<0.001
	1998 - 2006	-4.1 (-4.2 to -4)	<0.001
	2006 - 2010	-3.1 (-3.6 to -2.7)	<0.001
	2010 - 2015	-1.6 (-1.9 to -1.3)	<0.001
	2015 - 2021	-0.1 (-0.3 to 0.1)	0.173
Fiji	1990 - 1998	-1.8 (-2.4 to -1.2)	<0.001
	1998 - 2001	3.2 (-1.3 to 7.8)	0.157
	2001 - 2009	-1.8 (-2.3 to -1.3)	<0.001
	2009 - 2012	4.1 (0 to 8.4)	0.048
	2012 - 2021	-1.4 (-2 to -0.8)	<0.001
Finland	1990 - 1992	-9.7 (-17 to -1.7)	0.021
	1992 - 2007	-1.9 (-2.3 to -1.5)	<0.001
	2007 - 2010	2.5 (-7 to 13)	0.597
	2010 - 2015	-6.4 (-8.9 to -3.7)	<0.001
	2015 - 2019	4.3 (-1 to 9.8)	0.107
	2019 - 2021	-5.3 (-17.1 to 8.1)	0.392
France	1990 - 1993	3.7 (2 to 5.4)	<0.001
	1993 - 2000	-5.3 (-5.8 to -4.8)	<0.001
	2000 - 2003	-1 (-4.1 to 2.3)	0.528
	2003 - 2007	-3.9 (-5.3 to -2.3)	<0.001
	2007 - 2010	0.9 (-2.4 to 4.2)	0.569
	2010 - 2016	-5.6 (-6.3 to -4.9)	<0.001
	2016 - 2021	-0.9 (-2.1 to 0.2)	0.11
Gabon	1990 - 1998	1.3 (0.5 to 2.1)	0.002
	1998 - 2021	-0.7 (-0.8 to -0.5)	<0.001
Gambia	1990 - 1999	2.2 (1.1 to 3.3)	0.001
	1999 - 2002	-3 (-13.6 to 9)	0.589

	2002 - 2005	7.8 (-3.4 to 20.3)	0.165
	2005 - 2012	-4.2 (-6.1 to -2.2)	0.001
	2012 - 2017	5.4 (1.2 to 9.9)	0.015
	2017 - 2021	0.5 (-3.2 to 4.2)	0.798
Georgia	1990 - 1997	5.6 (3.8 to 7.4)	<0.001
	1997 - 2005	-8.4 (-10.1 to -6.6)	<0.001
	2005 - 2009	12.9 (5.4 to 20.9)	0.001
	2009 - 2021	0.2 (-0.6 to 1)	0.581
Germany	1990 - 2001	-1.9 (-2.2 to -1.6)	<0.001
	2001 - 2007	-3.4 (-4.2 to -2.6)	<0.001
	2007 - 2010	2.1 (-1.7 to 6.2)	0.261
	2010 - 2013	-3.3 (-6.9 to 0.5)	0.08
	2013 - 2016	1.2 (-2.2 to 4.8)	0.463
	2016 - 2021	-3.1 (-4.2 to -1.9)	<0.001
Ghana	1990 - 1995	0 (-0.5 to 0.6)	0.869
	1995 - 2000	1 (0.4 to 1.6)	0.004
	2000 - 2004	3.2 (2.3 to 4.2)	<0.001
	2004 - 2008	-1.7 (-2.6 to -0.8)	0.001
	2008 - 2017	1.7 (1.5 to 1.9)	<0.001
	2017 - 2021	-2.9 (-3.7 to -2.2)	<0.001
Greece	1990 - 1992	-7.1 (-16.7 to 3.6)	0.172
	1992 - 1997	1.5 (-2.1 to 5.2)	0.408
	1997 - 2008	-2.3 (-3.2 to -1.4)	<0.001
	2008 - 2012	4.7 (-1.9 to 11.7)	0.159
	2012 - 2021	-0.6 (-1.8 to 0.7)	0.345
Greenland	1990 - 2000	-4.8 (-5 to -4.6)	<0.001
	2000 - 2006	-1.4 (-1.9 to -0.8)	<0.001
	2006 - 2012	0.1 (-0.4 to 0.6)	0.792
	2012 - 2021	-1.8 (-2 to -1.5)	<0.001
Grenada	1990 - 1994	-6.3 (-8.5 to -4.1)	<0.001
	1994 - 2000	-2.5 (-4 to -1)	0.003
	2000 - 2008	2.1 (1.1 to 3.2)	<0.001
	2008 - 2014	-6.1 (-7.6 to -4.6)	<0.001
	2014 - 2021	1 (-0.2 to 2.3)	0.095
Guam	1990 - 1994	1.6 (-2 to 5.3)	0.365
	1994 - 1999	-5.4 (-8.3 to -2.5)	0.002
	1999 - 2007	-1.4 (-2.6 to -0.1)	0.033
	2007 - 2012	7.3 (4.5 to 10.2)	<0.001
	2012 - 2015	-5.7 (-13.7 to 3.1)	0.177
	2015 - 2018	11.3 (-0.5 to 24.5)	0.06
	2018 - 2021	-15.7 (-20.7 to -10.4)	<0.001
Guatemala	1990 - 1998	0.9 (-0.6 to 2.3)	0.212
	1998 - 2001	25.3 (9.8 to 42.9)	0.002
	2001 - 2012	-4.8 (-5.7 to -3.9)	<0.001

	2012 - 2019	2.8 (0.9 to 4.8)	0.007
	2019 - 2021	-11.5 (-26.3 to 6.3)	0.178
Guinea	1990 - 2007	1.7 (1.6 to 1.8)	<0.001
	2007 - 2015	0.5 (0.1 to 0.9)	0.018
	2015 - 2021	-1.4 (-2 to -0.8)	<0.001
Guinea-Bissau	1990 - 1997	0.6 (0.4 to 0.9)	<0.001
	1997 - 2002	1.5 (0.9 to 2.1)	<0.001
	2002 - 2009	0.5 (0.1 to 0.8)	0.006
	2009 - 2018	-0.9 (-1.1 to -0.7)	<0.001
	2018 - 2021	-1.9 (-2.8 to -1.1)	<0.001
Guyana	1990 - 1995	-4.7 (-7.5 to -1.8)	0.003
	1995 - 1999	12.1 (4.4 to 20.4)	0.004
	1999 - 2009	-2.5 (-3.3 to -1.8)	<0.001
	2009 - 2014	6.1 (3.3 to 9)	<0.001
	2014 - 2021	-1.6 (-3.4 to 0.2)	0.076
Haiti	1990 - 2021	-0.5 (-0.7 to -0.3)	<0.001
Honduras	1990 - 1997	-1.3 (-1.5 to -1.2)	<0.001
	1997 - 2003	-0.4 (-0.8 to 0)	0.05
	2003 - 2009	-2.9 (-3.3 to -2.5)	<0.001
	2009 - 2012	-6.5 (-8.4 to -4.5)	<0.001
	2012 - 2021	-0.5 (-0.7 to -0.3)	<0.001
Hungary	1990 - 1997	-3 (-4.1 to -1.9)	<0.001
	1997 - 2000	1 (-6.9 to 9.6)	0.8
	2000 - 2006	-5.6 (-7.3 to -3.8)	<0.001
	2006 - 2009	4.5 (-3.4 to 13)	0.255
	2009 - 2021	-3.3 (-3.9 to -2.7)	<0.001
Iceland	1990 - 1995	-6 (-8.4 to -3.5)	<0.001
	1995 - 2000	2.4 (-1.2 to 6.2)	0.181
	2000 - 2012	-3.2 (-4 to -2.5)	<0.001
	2012 - 2015	3.2 (-8 to 15.7)	0.567
	2015 - 2019	-6.7 (-12.7 to -0.2)	0.044
	2019 - 2021	7.5 (-8.2 to 26)	0.344
India	1990 - 1996	-0.6 (-2 to 0.7)	0.339
	1996 - 1999	2.8 (-4.3 to 10.5)	0.418
	1999 - 2002	-4.9 (-10.8 to 1.3)	0.111
	2002 - 2005	1.2 (-3.9 to 6.6)	0.612
	2005 - 2012	-2.3 (-3.2 to -1.3)	<0.001
	2012 - 2019	-5.3 (-6.2 to -4.5)	<0.001
	2019 - 2021	3 (-3 to 9.4)	0.299
Indonesia	1990 - 2003	0.3 (0.2 to 0.4)	<0.001
	2003 - 2011	-1.1 (-1.4 to -0.9)	<0.001
	2011 - 2014	0.2 (-1.4 to 1.9)	0.765
	2014 - 2021	-1 (-1.2 to -0.8)	<0.001
Iran (Islamic Republic	1990 - 1996	-1.1 (-1.6 to -0.6)	<0.001

of)			
	1996 - 2002	0.8 (0.2 to 1.3)	0.007
	2002 - 2006	-1.1 (-2 to -0.1)	0.029
	2006 - 2010	0.6 (-0.3 to 1.4)	0.173
	2010 - 2019	-3.5 (-3.7 to -3.3)	<0.001
	2019 - 2021	-6 (-7.8 to -4.2)	<0.001
Iraq	1990 - 1996	1.7 (1.3 to 2)	<0.001
	1996 - 2005	-2.1 (-2.3 to -1.9)	<0.001
	2005 - 2008	-3.8 (-5.8 to -1.7)	0.001
	2008 - 2017	-0.8 (-1 to -0.6)	<0.001
	2017 - 2021	-3.8 (-4.4 to -3.2)	<0.001
Ireland	1990 - 1993	2.7 (-0.5 to 6)	0.095
	1993 - 1997	10.9 (7.8 to 14)	<0.001
	1997 - 2006	-0.7 (-1.3 to -0.2)	0.013
	2006 - 2011	-3.6 (-5.1 to -2.2)	<0.001
	2011 - 2014	-11.3 (-15.8 to -6.6)	<0.001
	2014 - 2021	-6.2 (-7.4 to -5)	<0.001
Israel	1990 - 1997	4.6 (2.8 to 6.5)	<0.001
	1997 - 2009	-3.9 (-4.8 to -3.1)	<0.001
	2009 - 2012	-10.1 (-20.5 to 1.8)	0.09
	2012 - 2021	-0.9 (-2.4 to 0.6)	0.217
Italy	1990 - 1997	3.6 (2.3 to 4.9)	<0.001
	1997 - 2005	-6.4 (-7.6 to -5.3)	<0.001
	2005 - 2017	0.2 (-0.5 to 0.9)	0.497
	2017 - 2021	-6.3 (-10.1 to -2.5)	0.003
Jamaica	1990 - 2000	2.5 (0.9 to 4.2)	0.004
	2000 - 2006	-11.4 (-13.5 to -9.2)	<0.001
	2006 - 2009	22.6 (5.4 to 42.6)	0.011
	2009 - 2014	-4.4 (-7.8 to -0.9)	0.017
	2014 - 2021	2.2 (-0.6 to 5.1)	0.115
Japan	1990 - 1996	2.8 (1.7 to 4)	<0.001
	1996 - 1999	9.6 (3.8 to 15.6)	0.003
	1999 - 2002	-2.6 (-7.6 to 2.7)	0.3
	2002 - 2006	5.4 (2.7 to 8.1)	0.001
	2006 - 2011	0.7 (-0.7 to 2.1)	0.325
	2011 - 2016	-3.7 (-5 to -2.4)	<0.001
	2016 - 2021	2.5 (1.3 to 3.7)	0.001
Jordan	1990 - 1999	0.4 (0 to 0.8)	0.029
	1999 - 2004	-3 (-4.2 to -1.7)	<0.001
	2004 - 2009	-11.4 (-12.2 to -10.6)	<0.001
	2009 - 2012	-4.2 (-6.7 to -1.6)	0.004
	2012 - 2015	-1.3 (-4.1 to 1.6)	0.344
	2015 - 2018	-5 (-7.9 to -2)	0.004
	2018 - 2021	-0.4 (-2.1 to 1.5)	0.68

Kazakhstan	1990 - 1993	14.2 (8.2 to 20.5)	<0.001
	1993 - 2007	2.1 (1.6 to 2.7)	<0.001
	2007 - 2021	-6.9 (-7.4 to -6.5)	<0.001
Kenya	1990 - 1996	-0.7 (-1.1 to -0.3)	0.001
	1996 - 1999	0.9 (-1.4 to 3.2)	0.411
	1999 - 2004	3.3 (2.6 to 4)	<0.001
	2004 - 2011	0.6 (0.4 to 0.9)	<0.001
	2011 - 2015	-1.8 (-2.5 to -1.1)	<0.001
	2015 - 2021	-1 (-1.3 to -0.7)	<0.001
Kiribati	1990 - 1993	-0.3 (-0.6 to -0.1)	0.015
	1993 - 1998	-0.9 (-1 to -0.7)	<0.001
	1998 - 2002	-0.6 (-0.8 to -0.4)	<0.001
	2002 - 2005	0.2 (-0.3 to 0.7)	0.461
	2005 - 2011	1.1 (1 to 1.2)	<0.001
	2011 - 2015	-0.9 (-1.2 to -0.6)	<0.001
	2015 - 2021	-1.2 (-1.3 to -1.1)	<0.001
Kuwait	1990 - 1998	6.4 (3.5 to 9.3)	<0.001
	1998 - 2002	16.3 (4 to 30.1)	0.011
	2002 - 2005	-8.7 (-24.9 to 11.1)	0.344
	2005 - 2008	-33.1 (-46.7 to -16.1)	0.002
	2008 - 2021	5 (3.4 to 6.6)	<0.001
Kyrgyzstan	1990 - 2001	1.2 (0.7 to 1.7)	<0.001
	2001 - 2004	-7.7 (-13.7 to -1.2)	0.023
	2004 - 2011	1.6 (0.5 to 2.8)	0.007
	2011 - 2021	-4.1 (-4.7 to -3.4)	<0.001
Lao People's Democratic Republic	1990 - 1995	-1 (-1.8 to -0.1)	0.025
	1995 - 2006	-2.2 (-2.5 to -2)	<0.001
	2006 - 2021	-1.5 (-1.7 to -1.4)	<0.001
Latvia	1990 - 1994	8.9 (6.5 to 11.3)	<0.001
	1994 - 2003	-4.4 (-5.1 to -3.7)	<0.001
	2003 - 2012	-0.9 (-1.7 to 0)	0.041
	2012 - 2021	-6.4 (-7.2 to -5.6)	<0.001
Lebanon	1990 - 2003	-3.5 (-3.7 to -3.4)	<0.001
	2003 - 2010	-0.2 (-0.6 to 0.1)	0.171
	2010 - 2015	1.7 (1 to 2.4)	<0.001
	2015 - 2018	-6.7 (-8.6 to -4.8)	<0.001
	2018 - 2021	-2.7 (-3.7 to -1.6)	<0.001
Lesotho	1990 - 1993	-2.5 (-4.1 to -0.8)	0.007
	1993 - 1997	2.1 (0.5 to 3.7)	0.014
	1997 - 2000	12.5 (9.1 to 15.9)	<0.001
	2000 - 2005	7.5 (6.6 to 8.4)	<0.001
	2005 - 2015	1 (0.8 to 1.2)	<0.001
	2015 - 2021	0 (-0.4 to 0.5)	0.832

Liberia	1990 - 1992	-7.8 (-15.1 to 0.1)	0.052
	1992 - 1999	3.9 (2.2 to 5.6)	<0.001
	1999 - 2006	-0.8 (-2.8 to 1.3)	0.434
	2006 - 2013	4.3 (2.1 to 6.5)	0.001
	2013 - 2021	-2.2 (-3.5 to -0.9)	0.003
Libya	1990 - 1996	-0.3 (-1.3 to 0.6)	0.464
	1996 - 2003	2.2 (1.3 to 3.2)	<0.001
	2003 - 2006	-7.2 (-11.7 to -2.5)	0.006
	2006 - 2010	9 (6.3 to 11.9)	<0.001
	2010 - 2021	-0.8 (-1.2 to -0.4)	0.001
Lithuania	1990 - 1994	14.8 (9.5 to 20.3)	<0.001
	1994 - 2014	-0.7 (-1.1 to -0.3)	0.001
	2014 - 2017	-14 (-26.1 to 0.1)	0.051
	2017 - 2021	-3.9 (-10.3 to 2.9)	0.239
Luxembourg	1990 - 1994	-0.9 (-6.1 to 4.5)	0.711
	1994 - 1998	-9.9 (-16 to -3.4)	0.007
	1998 - 2005	-2.5 (-4.9 to 0)	0.049
	2005 - 2008	-11.1 (-24.5 to 4.6)	0.142
	2008 - 2011	2.3 (-11.3 to 17.9)	0.739
	2011 - 2015	-9.7 (-16.1 to -2.8)	0.011
	2015 - 2021	1.7 (-1.2 to 4.7)	0.221
Madagascar	1990 - 2006	-0.9 (-1 to -0.8)	<0.001
	2006 - 2021	0.2 (0 to 0.3)	0.015
Malawi	1990 - 2000	1.6 (1.4 to 1.8)	<0.001
	2000 - 2004	-0.4 (-1.7 to 1)	0.584
	2004 - 2008	-2.5 (-3.8 to -1.3)	0.001
	2008 - 2011	-0.8 (-3.1 to 1.6)	0.489
	2011 - 2014	-2.4 (-4.8 to 0.1)	0.06
	2014 - 2021	-0.3 (-0.6 to 0.1)	0.194
Malaysia	1990 - 2009	-2.3 (-3 to -1.7)	<0.001
	2009 - 2021	-0.1 (-1.5 to 1.3)	0.849
Maldives	1990 - 1997	-5.3 (-6.1 to -4.5)	<0.001
	1997 - 2000	8.6 (2 to 15.7)	0.013
	2000 - 2006	-4.2 (-5.5 to -2.8)	<0.001
	2006 - 2014	-0.4 (-1.2 to 0.4)	0.271
	2014 - 2021	-3.7 (-4.6 to -2.8)	<0.001
Mali	1990 - 1996	0.4 (0 to 0.8)	0.031
	1996 - 1999	-1.1 (-3.4 to 1.2)	0.316
	1999 - 2002	1.2 (-1.2 to 3.7)	0.299
	2002 - 2006	-0.7 (-1.9 to 0.5)	0.225
	2006 - 2017	0.5 (0.3 to 0.7)	<0.001
	2017 - 2021	-1.3 (-2 to -0.5)	0.003
Malta	1990 - 2013	-0.7 (-1.1 to -0.3)	0.001
	2013 - 2019	-7.9 (-11.4 to -4.2)	<0.001

	2019 - 2021	24.3 (1.8 to 51.7)	0.034
Marshall Islands	1990 - 1995	-5.4 (-7.2 to -3.6)	<0.001
	1995 - 2005	1.6 (0.8 to 2.3)	<0.001
	2005 - 2021	-0.7 (-1 to -0.4)	<0.001
Mauritania	1990 - 2018	-0.9 (-0.9 to -0.8)	<0.001
	2018 - 2021	1.4 (-1.3 to 4.2)	0.301
Mauritius	1990 - 1997	-1.1 (-2.6 to 0.4)	0.129
	1997 - 2004	-9 (-10.9 to -7.2)	<0.001
	2004 - 2021	1.9 (1.3 to 2.4)	<0.001
Mexico	1990 - 1993	4 (1.2 to 7)	0.008
	1993 - 1997	8.1 (5.6 to 10.6)	<0.001
	1997 - 2007	0.5 (0.1 to 1)	0.02
	2007 - 2011	7.3 (4.9 to 9.7)	<0.001
	2011 - 2021	1 (0.5 to 1.4)	<0.001
Micronesia (Federated States of)	1990 - 1993	0 (-0.3 to 0.4)	0.977
	1993 - 1999	-0.8 (-1 to -0.7)	<0.001
	1999 - 2002	-1.6 (-2.2 to -0.9)	<0.001
	2002 - 2021	-0.2 (-0.2 to -0.1)	<0.001
Monaco	1990 - 1999	-0.2 (-0.3 to -0.1)	0.004
	1999 - 2008	-1.5 (-1.6 to -1.4)	<0.001
	2008 - 2012	-0.9 (-1.3 to -0.4)	0.001
	2012 - 2017	-1.8 (-2.1 to -1.5)	<0.001
	2017 - 2021	0 (-0.4 to 0.3)	0.88
Mongolia	1990 - 1994	8.1 (2.6 to 14)	0.006
	1994 - 2001	-5.7 (-8.4 to -3)	<0.001
	2001 - 2008	2.3 (-0.1 to 4.8)	0.059
	2008 - 2011	10.2 (-3.8 to 26.3)	0.15
	2011 - 2021	-5.2 (-6.2 to -4.2)	<0.001
Montenegro	1990 - 1996	-3.7 (-7.1 to -0.1)	0.044
	1996 - 1999	23.7 (1.7 to 50.5)	0.035
	1999 - 2002	-13 (-27.4 to 4.2)	0.122
	2002 - 2005	3.5 (-12.8 to 22.9)	0.675
	2005 - 2021	-3.1 (-3.8 to -2.3)	<0.001
Morocco	1990 - 1993	1 (0.3 to 1.6)	0.005
	1993 - 2002	-0.5 (-0.7 to -0.4)	<0.001
	2002 - 2009	-1.8 (-2.1 to -1.6)	<0.001
	2009 - 2014	-3.1 (-3.5 to -2.7)	<0.001
	2014 - 2018	-4.2 (-4.8 to -3.6)	<0.001
	2018 - 2021	-1.8 (-2.4 to -1.1)	<0.001
Mozambique	1990 - 1993	-0.9 (-1.8 to 0)	0.055
	1993 - 1997	2.2 (1.3 to 3.2)	<0.001
	1997 - 2003	0.6 (0.2 to 1.1)	0.006
	2003 - 2009	3.8 (3.4 to 4.2)	<0.001

	2009 - 2015	1.1 (0.7 to 1.5)	<0.001
	2015 - 2021	-1.6 (-1.9 to -1.3)	<0.001
Myanmar	1990 - 1998	-0.3 (-0.6 to 0)	0.092
	1998 - 2005	-1.7 (-2.2 to -1.3)	<0.001
	2005 - 2011	-4.5 (-5 to -4)	<0.001
	2011 - 2021	-0.9 (-1.1 to -0.7)	<0.001
Namibia	1990 - 1997	1.9 (1.6 to 2.2)	<0.001
	1997 - 2000	4 (1.4 to 6.7)	0.005
	2000 - 2003	1.6 (-0.9 to 4.2)	0.195
	2003 - 2012	-2.1 (-2.4 to -1.9)	<0.001
	2012 - 2015	0.9 (-2.1 to 4)	0.544
	2015 - 2021	-1.4 (-2 to -0.8)	<0.001
Nauru	1990 - 1994	0.4 (0 to 0.8)	0.043
	1994 - 1999	2.1 (1.7 to 2.5)	<0.001
	1999 - 2004	0.8 (0.4 to 1.3)	0.001
	2004 - 2009	-0.4 (-0.8 to 0.1)	0.085
	2009 - 2018	-2.1 (-2.3 to -2)	<0.001
	2018 - 2021	-0.7 (-1.4 to -0.1)	0.024
Nepal	1990 - 1994	-0.5 (-1 to 0)	0.044
	1994 - 1999	-1.6 (-2 to -1.1)	<0.001
	1999 - 2002	-2.4 (-3.6 to -1.2)	<0.001
	2002 - 2009	0.4 (0.2 to 0.6)	0.001
	2009 - 2021	-1 (-1 to -0.9)	<0.001
Netherlands	1990 - 1997	-0.6 (-1.8 to 0.6)	0.299
	1997 - 2003	-4 (-6 to -1.9)	0.001
	2003 - 2009	-0.7 (-2.8 to 1.4)	0.458
	2009 - 2012	4.6 (-3.3 to 13.1)	0.237
	2012 - 2015	-3.7 (-10.4 to 3.6)	0.287
	2015 - 2018	6.4 (-2.1 to 15.6)	0.133
	2018 - 2021	-2.5 (-7.4 to 2.7)	0.305
New Zealand	1990 - 1996	2.6 (0.9 to 4.3)	0.005
	1996 - 2002	-6.5 (-8.4 to -4.5)	<0.001
	2002 - 2012	0.6 (-0.2 to 1.4)	0.116
	2012 - 2015	-4.7 (-11.9 to 3.2)	0.221
	2015 - 2018	2.8 (-6.2 to 12.6)	0.536
	2018 - 2021	-5.7 (-11.4 to 0.4)	0.066
Nicaragua	1990 - 1996	9.5 (8.2 to 10.9)	<0.001
	1996 - 2000	2.4 (-1.2 to 6.2)	0.187
	2000 - 2018	-1.8 (-2.1 to -1.5)	<0.001
	2018 - 2021	-9.3 (-13.2 to -5.4)	<0.001
Niger	1990 - 2003	0.1 (-0.1 to 0.3)	0.181
	2003 - 2010	-2.3 (-3 to -1.6)	<0.001
	2010 - 2014	2.6 (0.2 to 5)	0.035
	2014 - 2021	-0.4 (-1.1 to 0.3)	0.244

Nigeria	1990 - 2001	1 (0.9 to 1.1)	<0.001
	2001 - 2008	-1.9 (-2.1 to -1.6)	<0.001
	2008 - 2014	0.3 (0 to 0.7)	0.066
	2014 - 2021	-1.9 (-2.1 to -1.7)	<0.001
Niue	1990 - 2018	-0.7 (-1 to -0.4)	<0.001
	2018 - 2021	19.7 (11.5 to 28.6)	<0.001
North Macedonia	1990 - 1999	1.8 (0.6 to 3)	0.005
	1999 - 2021	-2.5 (-2.9 to -2.2)	<0.001
Northern Mariana Islands	1990 - 1999	-5.1 (-7.8 to -2.4)	0.001
	1999 - 2021	1.8 (1.2 to 2.5)	<0.001
Norway	1990 - 2007	-2.8 (-3.3 to -2.3)	<0.001
	2007 - 2010	3.2 (-10.1 to 18.6)	0.639
	2010 - 2013	-7.6 (-19.7 to 6.4)	0.257
	2013 - 2021	1.2 (-0.6 to 2.9)	0.177
Oman	1990 - 2008	-0.6 (-1 to -0.1)	0.01
	2008 - 2017	4.5 (3.1 to 5.8)	<0.001
	2017 - 2021	-19.1 (-22.4 to -15.7)	<0.001
Pakistan	1990 - 1995	3.9 (3.6 to 4.2)	<0.001
	1995 - 2003	0.9 (0.7 to 1.1)	<0.001
	2003 - 2011	-1.5 (-1.7 to -1.3)	<0.001
	2011 - 2015	0.6 (-0.3 to 1.4)	0.187
	2015 - 2021	-1.8 (-2 to -1.5)	<0.001
Palau	1990 - 1995	2.6 (0.9 to 4.4)	0.005
	1995 - 2014	0.2 (0 to 0.4)	0.092
	2014 - 2021	-1.6 (-2.3 to -0.9)	<0.001
Palestine	1990 - 2003	-0.7 (-0.8 to -0.5)	<0.001
	2003 - 2007	-3.8 (-4.8 to -2.7)	<0.001
	2007 - 2010	-9.1 (-11.1 to -7.1)	<0.001
	2010 - 2014	18.4 (17.2 to 19.6)	<0.001
	2014 - 2017	-9.2 (-10.7 to -7.6)	<0.001
	2017 - 2021	-0.3 (-1 to 0.3)	0.306
Panama	1990 - 1998	4.5 (3.3 to 5.8)	<0.001
	1998 - 2008	0.4 (-0.4 to 1.3)	0.297
	2008 - 2021	-1.9 (-2.5 to -1.3)	<0.001
Papua New Guinea	1990 - 2002	0.4 (-0.2 to 1)	0.155
	2002 - 2006	-2.5 (-8.1 to 3.5)	0.387
	2006 - 2021	1.4 (0.9 to 1.9)	<0.001
Paraguay	1990 - 1995	7.3 (5.8 to 8.9)	<0.001
	1995 - 1998	-0.9 (-6.8 to 5.4)	0.764
	1998 - 2004	4.3 (2.5 to 6.1)	<0.001
	2004 - 2019	1.4 (1 to 1.8)	<0.001
	2019 - 2021	-8.9 (-17.7 to 0.8)	0.069
Peru	1990 - 1994	3.7 (1.5 to 6.1)	0.004

	1994 - 1998	0.2 (-2.8 to 3.4)	0.878
	1998 - 2006	-2.2 (-3.2 to -1.2)	<0.001
	2006 - 2011	1.7 (-0.9 to 4.4)	0.187
	2011 - 2016	-3.1 (-5.7 to -0.3)	0.03
	2016 - 2019	8.6 (-1.3 to 19.4)	0.084
	2019 - 2021	-11 (-20 to -0.9)	0.036
Philippines	1990 - 1997	-3.6 (-4.4 to -2.8)	<0.001
	1997 - 2021	0.3 (0.2 to 0.4)	<0.001
Poland	1990 - 1994	0.9 (-1.2 to 3.1)	0.361
	1994 - 1999	3.5 (1.6 to 5.4)	0.001
	1999 - 2014	0.3 (0 to 0.5)	0.024
	2014 - 2017	-7.3 (-11.8 to -2.6)	0.005
	2017 - 2021	-3 (-5.4 to -0.4)	0.025
Portugal	1990 - 2001	-3.5 (-4.1 to -2.9)	<0.001
	2001 - 2004	-14.3 (-23 to -4.6)	0.007
	2004 - 2009	1.5 (-1.7 to 4.7)	0.339
	2009 - 2013	-6.2 (-10.3 to -1.8)	0.008
	2013 - 2021	-1.1 (-2.3 to 0.2)	0.094
Puerto Rico	1990 - 1995	2.5 (-0.3 to 5.4)	0.078
	1995 - 2012	-2.9 (-3.4 to -2.3)	<0.001
	2012 - 2021	-4.8 (-6.4 to -3.1)	<0.001
Qatar	1990 - 1995	-2.4 (-4.5 to -0.3)	0.029
	1995 - 2001	7 (4.7 to 9.2)	<0.001
	2001 - 2018	-2.8 (-3.2 to -2.4)	<0.001
	2018 - 2021	-16.6 (-21.4 to -11.4)	<0.001
Republic of Korea	1990 - 1997	1.1 (-0.6 to 2.8)	0.186
	1997 - 2001	-4.8 (-7.2 to -2.4)	0.001
	2001 - 2006	1.4 (0.4 to 2.5)	0.013
	2006 - 2009	4.6 (-0.8 to 10.2)	0.087
	2009 - 2015	-3 (-4.3 to -1.8)	<0.001
	2015 - 2019	3 (-0.1 to 6.1)	0.053
	2019 - 2021	-3 (-9.1 to 3.5)	0.326
Republic of Moldova	1990 - 1994	2.7 (-0.2 to 5.6)	0.069
	1994 - 2006	-1.9 (-2.5 to -1.3)	<0.001
	2006 - 2010	7.5 (2.6 to 12.6)	0.004
	2010 - 2016	-3.2 (-5.2 to -1.2)	0.004
	2016 - 2021	-7.6 (-10.1 to -5.2)	<0.001
Romania	1990 - 1994	4.2 (0.9 to 7.7)	0.016
	1994 - 2007	-0.3 (-0.9 to 0.3)	0.302
	2007 - 2010	8 (-2.9 to 20.2)	0.147
	2010 - 2021	-5.7 (-6.6 to -4.9)	<0.001
Russian Federation	1990 - 1994	15.2 (11.4 to 19)	<0.001
	1994 - 2005	1.4 (0.6 to 2.2)	0.001
	2005 - 2012	-3.4 (-4.9 to -1.9)	<0.001

	2012 - 2021	-10 (-11.3 to -8.7)	<0.001
Rwanda	1990 - 1993	6.9 (5.2 to 8.6)	<0.001
	1993 - 1996	-5 (-8.4 to -1.6)	0.007
	1996 - 1999	-0.8 (-4.1 to 2.6)	0.624
	1999 - 2010	-5.6 (-5.9 to -5.4)	<0.001
	2010 - 2021	-1.1 (-1.4 to -0.8)	<0.001
Saint Kitts and Nevis	1990 - 1996	2.7 (0.6 to 4.8)	0.012
	1996 - 2002	-6.9 (-9.9 to -3.8)	<0.001
	2002 - 2021	-0.8 (-1.3 to -0.3)	0.006
Saint Lucia	1990 - 2001	-0.6 (-1.1 to -0.2)	0.006
	2001 - 2006	-2.9 (-5.1 to -0.7)	0.012
	2006 - 2012	3.5 (1.9 to 5.1)	<0.001
	2012 - 2021	-2.3 (-3.2 to -1.4)	<0.001
Saint Vincent and the Grenadines	1990 - 2002	0.6 (0.1 to 1.1)	0.019
	2002 - 2006	-3.6 (-7.8 to 0.6)	0.09
	2006 - 2009	3.2 (-5.5 to 12.8)	0.46
	2009 - 2012	-4 (-11.9 to 4.5)	0.324
	2012 - 2021	-0.2 (-1.1 to 0.7)	0.612
Samoa	1990 - 1995	-0.9 (-1.1 to -0.7)	<0.001
	1995 - 2000	-2.2 (-2.4 to -1.9)	<0.001
	2000 - 2003	-3 (-3.8 to -2.1)	<0.001
	2003 - 2007	-1 (-1.4 to -0.5)	<0.001
	2007 - 2016	1.4 (1.3 to 1.5)	<0.001
	2016 - 2021	-1.2 (-1.4 to -1)	<0.001
San Marino	1990 - 1999	-0.7 (-0.9 to -0.5)	<0.001
	1999 - 2006	-3.5 (-3.7 to -3.2)	<0.001
	2006 - 2019	-0.5 (-0.6 to -0.4)	<0.001
	2019 - 2021	-8.8 (-10.8 to -6.8)	<0.001
Sao Tome and Principe	1990 - 2001	4.6 (2.9 to 6.3)	<0.001
	2001 - 2021	-0.6 (-1.1 to 0)	0.042
Saudi Arabia	1990 - 1998	-1 (-1.2 to -0.7)	<0.001
	1998 - 2002	1.2 (0.4 to 2.1)	0.009
	2002 - 2006	-1.6 (-2.3 to -0.9)	<0.001
	2006 - 2009	-4 (-5.4 to -2.6)	<0.001
	2009 - 2012	-2.3 (-3.9 to -0.6)	0.011
	2012 - 2017	0.2 (-0.4 to 0.8)	0.439
	2017 - 2021	-1.8 (-2.5 to -1)	<0.001
Senegal	1990 - 2021	-0.1 (-0.3 to 0.1)	0.523
Serbia	1990 - 1997	-0.8 (-1.7 to 0.1)	0.072
	1997 - 2003	-3.2 (-4.2 to -2.2)	<0.001
	2003 - 2008	-7.7 (-9.1 to -6.3)	<0.001
	2008 - 2016	-4.5 (-5.2 to -3.7)	<0.001

	2016 - 2019	0.3 (-6.6 to 7.8)	0.922
	2019 - 2021	-9.9 (-17.1 to -2.1)	0.017
Seychelles	1990 - 2012	-1 (-1.3 to -0.6)	<0.001
	2012 - 2019	8.3 (4.8 to 12)	<0.001
	2019 - 2021	-50.3 (-59.7 to -38.7)	<0.001
Sierra Leone	1990 - 1994	-1.5 (-2.9 to 0)	0.044
	1994 - 2000	1 (-0.1 to 2.1)	0.064
	2000 - 2005	5.5 (3.9 to 7)	<0.001
	2005 - 2009	-1.6 (-3.7 to 0.6)	0.143
	2009 - 2017	1.1 (0.5 to 1.7)	0.002
	2017 - 2021	-0.9 (-2.5 to 0.6)	0.213
Singapore	1990 - 1995	-3.9 (-6.2 to -1.6)	0.003
	1995 - 2002	1.7 (-0.1 to 3.5)	0.056
	2002 - 2005	-9.1 (-17.3 to -0.1)	0.048
	2005 - 2019	0 (-0.6 to 0.5)	0.9
	2019 - 2021	8.5 (-3.9 to 22.4)	0.175
Slovakia	1990 - 1995	-1.4 (-2.8 to 0.1)	0.059
	1995 - 1998	2.8 (-2.3 to 8.3)	0.265
	1998 - 2001	-3.4 (-8.3 to 1.8)	0.183
	2001 - 2008	1.3 (0.4 to 2.3)	0.011
	2008 - 2019	-2.4 (-2.9 to -1.9)	<0.001
	2019 - 2021	-9.1 (-17.4 to 0)	0.05
Slovenia	1990 - 1999	0.1 (-1.1 to 1.3)	0.884
	1999 - 2015	-4 (-4.5 to -3.5)	<0.001
	2015 - 2021	-8.9 (-11.6 to -6.1)	<0.001
Solomon Islands	1990 - 2010	-0.7 (-0.9 to -0.5)	<0.001
	2010 - 2013	4.1 (-2.1 to 10.6)	0.19
	2013 - 2021	-0.2 (-0.9 to 0.4)	0.49
Somalia	1990 - 1992	-1.2 (-2.5 to 0.2)	0.089
	1992 - 1996	5.2 (4.5 to 5.9)	<0.001
	1996 - 2000	1.6 (0.8 to 2.3)	<0.001
	2000 - 2006	-1.4 (-1.7 to -1)	<0.001
	2006 - 2009	0.7 (-0.7 to 2.1)	0.32
	2009 - 2015	-1.2 (-1.5 to -0.9)	<0.001
	2015 - 2021	-0.4 (-0.6 to -0.2)	0.003
South Africa	1990 - 2004	3.1 (2.7 to 3.4)	<0.001
	2004 - 2008	-0.9 (-3.8 to 2.1)	0.523
	2008 - 2011	-4.8 (-10 to 0.7)	0.083
	2011 - 2021	-1.3 (-1.9 to -0.8)	<0.001
South Sudan	1990 - 1995	4.2 (2.1 to 6.4)	<0.001
	1995 - 2002	-4.6 (-6.1 to -3.1)	<0.001
	2002 - 2007	3.6 (0.5 to 6.7)	0.024
	2007 - 2010	-1.8 (-10.8 to 8.2)	0.704
	2010 - 2021	2.7 (2.1 to 3.4)	<0.001

Spain	1990 - 1997	0.7 (-0.7 to 2.1)	0.332
	1997 - 2010	-4.1 (-4.7 to -3.5)	<0.001
	2010 - 2013	3.8 (-7 to 15.9)	0.485
	2013 - 2021	-2.4 (-3.8 to -1)	0.002
Sri Lanka	1990 - 1999	0.9 (-1.2 to 3.1)	0.388
	1999 - 2004	-10.9 (-15.2 to -6.2)	<0.001
	2004 - 2015	-5.4 (-6.6 to -4.2)	<0.001
	2015 - 2021	2.2 (-5.4 to 10.4)	0.564
Sudan	1990 - 1994	0.2 (-0.2 to 0.6)	0.325
	1994 - 2001	-0.8 (-1 to -0.6)	<0.001
	2001 - 2006	-2.3 (-2.7 to -1.8)	<0.001
	2006 - 2012	-0.6 (-0.9 to -0.3)	0.001
	2012 - 2016	-2.1 (-2.8 to -1.3)	<0.001
	2016 - 2019	-0.8 (-2.4 to 0.7)	0.267
	2019 - 2021	-2.5 (-4 to -1)	0.004
Suriname	1990 - 1993	5.6 (-1.3 to 12.9)	0.103
	1993 - 2001	-6.6 (-7.9 to -5.2)	<0.001
	2001 - 2007	6.8 (4.8 to 8.8)	<0.001
	2007 - 2011	-0.3 (-3.9 to 3.5)	0.877
	2011 - 2014	-7.6 (-14.5 to -0.2)	0.046
	2014 - 2017	5.8 (-2.3 to 14.5)	0.149
	2017 - 2021	-1.2 (-4.1 to 1.8)	0.388
Sweden	1990 - 1992	-7.4 (-14.8 to 0.6)	0.065
	1992 - 2001	-2.7 (-3.6 to -1.8)	<0.001
	2001 - 2011	2.6 (1.8 to 3.4)	<0.001
	2011 - 2016	-2.4 (-5.1 to 0.4)	0.088
	2016 - 2019	2.4 (-6.1 to 11.7)	0.563
	2019 - 2021	-11.3 (-22.8 to 1.9)	0.084
Switzerland	1990 - 1992	1 (-7.2 to 9.9)	0.813
	1992 - 2004	-5.4 (-5.8 to -4.9)	<0.001
	2004 - 2007	0.4 (-7 to 8.2)	0.922
	2007 - 2010	-7.1 (-13.9 to 0.4)	0.061
	2010 - 2021	-1.9 (-2.6 to -1.3)	<0.001
Syrian Arab Republic	1990 - 2000	-1.2 (-1.6 to -0.9)	<0.001
	2000 - 2003	-7.8 (-11.2 to -4.2)	<0.001
	2003 - 2008	-4.1 (-5.2 to -3.1)	<0.001
	2008 - 2011	0.5 (-3.6 to 4.8)	0.8
	2011 - 2016	-4.4 (-5.9 to -2.9)	<0.001
	2016 - 2021	2.9 (1.6 to 4.2)	<0.001
Taiwan (Province of China)	1990 - 1998	-0.1 (-0.9 to 0.7)	0.776
	1998 - 2005	5.3 (4 to 6.6)	<0.001
	2005 - 2010	-6.4 (-8.4 to -4.4)	<0.001
	2010 - 2014	0.4 (-3 to 4)	0.814

	2014 - 2021	3.4 (2.1 to 4.7)	<0.001
Tajikistan	1990 - 1995	7.5 (6.3 to 8.7)	<0.001
	1995 - 2000	-7.3 (-8.9 to -5.5)	<0.001
	2000 - 2007	-3.9 (-5 to -2.8)	<0.001
	2007 - 2015	1.3 (0.1 to 2.5)	0.032
	2015 - 2021	-3.3 (-5 to -1.6)	0.001
Thailand	1990 - 1996	7.3 (6.5 to 8.1)	<0.001
	1996 - 1999	-11.3 (-15.8 to -6.6)	<0.001
	1999 - 2008	-4.5 (-5 to -4)	<0.001
	2008 - 2013	-1.9 (-3.2 to -0.5)	0.01
	2013 - 2021	0.6 (0.1 to 1.2)	0.024
Timor-Leste	1990 - 2000	-3 (-4.5 to -1.5)	0.001
	2000 - 2008	-8.3 (-10.9 to -5.8)	<0.001
	2008 - 2012	17.5 (6.1 to 30.1)	0.004
	2012 - 2021	0.5 (-1.2 to 2.3)	0.529
Togo	1990 - 1998	2.6 (2.5 to 2.8)	<0.001
	1998 - 2003	0.8 (0.3 to 1.4)	0.004
	2003 - 2006	-0.7 (-2.4 to 1)	0.379
	2006 - 2009	2.8 (1.1 to 4.4)	0.002
	2009 - 2017	-0.5 (-0.7 to -0.3)	<0.001
	2017 - 2021	-1.7 (-2.3 to -1.1)	<0.001
Tokelau	1990 - 2018	-1.2 (-1.7 to -0.8)	<0.001
	2018 - 2021	30.9 (17.6 to 45.8)	<0.001
Tonga	1990 - 1992	-4 (-6.4 to -1.6)	0.002
	1992 - 1996	-0.6 (-1.8 to 0.5)	0.27
	1996 - 1999	6.9 (4.7 to 9.1)	<0.001
	1999 - 2021	0 (0 to 0.1)	0.195
Trinidad and Tobago	1990 - 1994	-8.4 (-10.8 to -6)	<0.001
	1994 - 2006	-0.4 (-1 to 0.3)	0.239
	2006 - 2009	4.3 (-6.8 to 16.7)	0.442
	2009 - 2012	-7.5 (-17.5 to 3.7)	0.17
	2012 - 2021	0.8 (-1.1 to 2.7)	0.408
Tunisia	1990 - 1992	5.2 (3.8 to 6.6)	<0.001
	1992 - 1995	-1.4 (-2.6 to -0.2)	0.023
	1995 - 2003	-0.2 (-0.4 to 0)	0.02
	2003 - 2014	-1.6 (-1.7 to -1.4)	<0.001
	2014 - 2021	-2.3 (-2.5 to -2.1)	<0.001
Türkiye	1990 - 1999	-1.5 (-1.9 to -1.1)	<0.001
	1999 - 2002	-5.3 (-9.6 to -0.9)	0.022
	2002 - 2005	4.9 (1.3 to 8.6)	0.011
	2005 - 2009	0.9 (-0.5 to 2.3)	0.176
	2009 - 2012	-1.9 (-4.1 to 0.3)	0.089
	2012 - 2017	2.8 (2.1 to 3.5)	<0.001
	2017 - 2021	-0.7 (-1.7 to 0.3)	0.171

Turkmenistan	1990 - 1995	2.4 (0.7 to 4)	0.007
	1995 - 1998	13.8 (6.3 to 21.9)	0.001
	1998 - 2003	-1.7 (-3.9 to 0.6)	0.127
	2003 - 2006	5.3 (-2.6 to 13.8)	0.182
	2006 - 2010	-7.3 (-10.3 to -4.3)	<0.001
	2010 - 2021	-0.7 (-1.4 to 0.1)	0.066
Tuvalu	1990 - 1994	-2.8 (-3.1 to -2.6)	<0.001
	1994 - 1998	-1.2 (-1.6 to -0.9)	<0.001
	1998 - 2002	-2.4 (-2.8 to -2)	<0.001
	2002 - 2006	-1.1 (-1.4 to -0.7)	<0.001
	2006 - 2012	0.1 (-0.1 to 0.2)	0.468
	2012 - 2016	-0.5 (-0.9 to -0.1)	0.022
	2016 - 2021	-1.1 (-1.2 to -0.9)	<0.001
Uganda	1990 - 1995	7.5 (6.3 to 8.7)	<0.001
	1995 - 2001	1.8 (0.6 to 3)	0.004
	2001 - 2011	-2.9 (-3.4 to -2.5)	<0.001
	2011 - 2021	-0.1 (-0.4 to 0.3)	0.663
Ukraine	1990 - 1995	9.9 (8.3 to 11.6)	<0.001
	1995 - 2000	1.9 (-0.2 to 4)	0.077
	2000 - 2003	-4 (-10.2 to 2.7)	0.221
	2003 - 2013	1.1 (0.5 to 1.7)	0.001
	2013 - 2021	-4.1 (-5.7 to -2.6)	<0.001
United Arab Emirates	1990 - 2002	0 (-1 to 1.1)	0.996
	2002 - 2005	11.9 (-4.7 to 31.5)	0.159
	2005 - 2008	-17.5 (-30.8 to -1.6)	0.034
	2008 - 2016	4.4 (1.7 to 7.1)	0.002
	2016 - 2021	-8.2 (-11.8 to -4.4)	<0.001
United Kingdom	1990 - 1999	-1.2 (-2.1 to -0.4)	0.006
	1999 - 2004	-6 (-9 to -2.9)	0.001
	2004 - 2016	-0.7 (-1.3 to 0)	0.041
	2016 - 2021	2.4 (-0.7 to 5.7)	0.124
United Republic of Tanzania	1990 - 1998	0.4 (0.2 to 0.5)	<0.001
	1998 - 2001	-2 (-3.2 to -0.8)	0.003
	2001 - 2004	-5.1 (-6.2 to -3.9)	<0.001
	2004 - 2009	-0.2 (-0.5 to 0.2)	0.318
	2009 - 2014	-1.5 (-1.9 to -1.1)	<0.001
	2014 - 2021	-0.1 (-0.3 to 0.1)	0.288
United States of America	1990 - 1994	0.7 (-0.1 to 1.6)	0.07
	1994 - 1999	-4.8 (-5.6 to -3.9)	<0.001
	1999 - 2007	-0.4 (-0.8 to 0)	0.037
	2007 - 2014	2.7 (2.2 to 3.1)	<0.001
	2014 - 2017	5.9 (3.3 to 8.5)	<0.001

	2017 - 2021	-1.2 (-2.4 to 0)	0.053
United States Virgin Islands	1990 - 1996	3.4 (1.2 to 5.7)	0.004
	1996 - 2005	-1.1 (-2.5 to 0.3)	0.111
	2005 - 2018	5.6 (4.5 to 6.7)	<0.001
	2018 - 2021	-14.8 (-23.4 to -5.3)	0.005
Uruguay	1990 - 1999	3.3 (2.4 to 4.3)	<0.001
	1999 - 2006	-0.9 (-2.5 to 0.6)	0.224
	2006 - 2021	3 (2.6 to 3.5)	<0.001
Uzbekistan	1990 - 1995	0 (-0.9 to 0.9)	0.926
	1995 - 2000	4.9 (3.4 to 6.3)	<0.001
	2000 - 2004	-2.9 (-5.1 to -0.6)	0.02
	2004 - 2007	6.2 (0.9 to 11.8)	0.024
	2007 - 2010	-1.8 (-6 to 2.6)	0.392
	2010 - 2015	8.1 (6.8 to 9.4)	<0.001
	2015 - 2021	-2.7 (-3.4 to -1.9)	<0.001
Vanuatu	1990 - 1992	5.8 (2.8 to 8.9)	0.001
	1992 - 2000	0 (-0.4 to 0.3)	0.807
	2000 - 2003	-4.1 (-6.3 to -1.9)	0.001
	2003 - 2007	1.7 (0.5 to 2.9)	0.007
	2007 - 2021	-0.3 (-0.4 to -0.2)	<0.001
Venezuela (Bolivarian Republic of)	1990 - 1998	6.3 (5.3 to 7.4)	<0.001
	1998 - 2001	-3.6 (-12.3 to 5.9)	0.428
	2001 - 2021	1.3 (0.9 to 1.7)	<0.001
Viet Nam	1990 - 1997	-2.6 (-2.7 to -2.5)	<0.001
	1997 - 2001	-2 (-2.3 to -1.7)	<0.001
	2001 - 2005	1.2 (0.9 to 1.5)	<0.001
	2005 - 2008	0.5 (-0.2 to 1.2)	0.138
	2008 - 2013	1.4 (1.2 to 1.6)	<0.001
	2013 - 2016	-1 (-1.7 to -0.3)	0.01
	2016 - 2021	-2.2 (-2.4 to -2.1)	<0.001
Yemen	1990 - 2008	2.5 (2.1 to 2.9)	<0.001
	2008 - 2011	-10.4 (-19.6 to -0.1)	0.047
	2011 - 2021	-1.9 (-2.9 to -0.9)	0.001
Zambia	1990 - 1995	4.7 (4.3 to 5.1)	<0.001
	1995 - 1998	1.1 (-0.7 to 3)	0.198
	1998 - 2008	-1.5 (-1.7 to -1.3)	<0.001
	2008 - 2011	-0.3 (-1.9 to 1.4)	0.725
	2011 - 2014	-2.6 (-4.4 to -0.8)	0.008
	2014 - 2017	-0.6 (-2.7 to 1.5)	0.544
	2017 - 2021	-1.8 (-2.6 to -1)	<0.001
Zimbabwe	1990 - 1997	0.9 (-0.6 to 2.4)	0.243
	1997 - 2002	12.6 (9.3 to 16)	<0.001

	2002 - 2021	0.7 (0.5 to 1)	<0.001

Table S5. The trends in rate of suicide YLLs in 204 countries by Joinpoint regression

location	Year	Estimate (95%UI)	P value
Afghanistan	1990 - 1995	3.9 (2.9 to 4.9)	<0.001
	1995 - 1999	-1.6 (-3.5 to 0.3)	0.091
	1999 - 2002	-7.4 (-11 to -3.6)	0.001
	2002 - 2005	1.4 (-2.2 to 5.3)	0.423
	2005 - 2009	-3.1 (-5 to -1.2)	0.004
	2009 - 2021	0 (-0.3 to 0.2)	0.914
Albania	1990 - 1998	3 (1.4 to 4.6)	0.001
	1998 - 2018	-2.9 (-3.4 to -2.3)	<0.001
	2018 - 2021	-16.1 (-25.9 to -5)	0.007
Algeria	1990 - 1992	-3.2 (-5.2 to -1.1)	0.004
	1992 - 2001	-0.1 (-0.3 to 0.1)	0.337
	2001 - 2006	-3.3 (-4 to -2.7)	<0.001
	2006 - 2015	-1.9 (-2.1 to -1.7)	<0.001
	2015 - 2021	-2.9 (-3.2 to -2.6)	<0.001
American Samoa	1990 - 2007	-0.9 (-1.1 to -0.7)	<0.001
	2007 - 2015	1.8 (1.3 to 2.4)	<0.001
	2015 - 2021	0.6 (-0.1 to 1.4)	0.091
Andorra	1990 - 1992	0.8 (-2.1 to 3.9)	0.561
	1992 - 2000	-1.9 (-2.3 to -1.5)	<0.001
	2000 - 2003	4 (1.1 to 7)	0.009
	2003 - 2006	0.5 (-2.4 to 3.5)	0.718
	2006 - 2011	-7.9 (-8.6 to -7.1)	<0.001
	2011 - 2021	-1.2 (-1.4 to -1)	<0.001
Angola	1990 - 1994	0.9 (-1.2 to 3.1)	0.39
	1994 - 1997	-7.5 (-13.6 to -1)	0.027
	1997 - 2000	3.5 (-3.2 to 10.7)	0.301
	2000 - 2021	-0.9 (-1.1 to -0.7)	<0.001
Antigua and Barbuda	1990 - 1998	5.3 (1.9 to 8.9)	0.004
	1998 - 2007	-4.9 (-8.1 to -1.7)	0.005
	2007 - 2021	-0.2 (-1.8 to 1.4)	0.794
Argentina	1990 - 2002	7.2 (6.8 to 7.6)	<0.001
	2002 - 2005	-3.5 (-9.2 to 2.6)	0.242
	2005 - 2012	1.9 (0.9 to 3)	0.001
	2012 - 2019	-0.1 (-1.3 to 1.1)	0.848
	2019 - 2021	-8.2 (-15.3 to -0.4)	0.04
Armenia	1990 - 1994	11.8 (6.8 to 17)	<0.001
	1994 - 2003	-9.9 (-11.7 to -8)	<0.001
	2003 - 2006	32.7 (14.1 to 54.4)	0.001
	2006 - 2010	3.7 (-2.9 to 10.8)	0.262
	2010 - 2021	-5 (-6 to -3.9)	<0.001
Australia	1990 - 1994	-1.8 (-3.3 to -0.2)	0.028
	1994 - 1997	4.5 (-0.3 to 9.6)	0.065

	1997 - 2003	-7.6 (-8.7 to -6.4)	<0.001
	2003 - 2009	-0.9 (-2 to 0.3)	0.136
	2009 - 2019	3 (2.5 to 3.4)	<0.001
	2019 - 2021	-2.9 (-9.2 to 3.8)	0.359
Austria	1990 - 2006	-2.8 (-3.2 to -2.4)	<0.001
	2006 - 2021	-1.7 (-2.3 to -1.2)	<0.001
Azerbaijan	1990 - 1993	25.5 (19.7 to 31.5)	<0.001
	1993 - 1997	-18.6 (-23.9 to -13)	<0.001
	1997 - 2006	2.7 (1.8 to 3.7)	<0.001
	2006 - 2017	-0.4 (-1.2 to 0.3)	0.253
	2017 - 2021	-8.2 (-11.7 to -4.5)	<0.001
Bahamas	1990 - 1999	-0.4 (-1 to 0.3)	0.238
	1999 - 2006	-2.8 (-4 to -1.6)	<0.001
	2006 - 2010	6.8 (3 to 10.7)	0.001
	2010 - 2021	-1.2 (-2 to -0.5)	0.003
Bahrain	1990 - 1994	-4 (-6.3 to -1.6)	0.003
	1994 - 2001	5.2 (3.9 to 6.6)	<0.001
	2001 - 2013	-4.3 (-4.8 to -3.7)	<0.001
	2013 - 2016	-7.3 (-15.4 to 1.5)	0.097
	2016 - 2021	-0.2 (-2.4 to 2.1)	0.86
Bangladesh	1990 - 1996	-1.7 (-2.5 to -1)	<0.001
	1996 - 2004	-3.3 (-3.8 to -2.7)	<0.001
	2004 - 2007	-0.5 (-3.8 to 2.9)	0.768
	2007 - 2010	-3.5 (-6.6 to -0.3)	0.033
	2010 - 2013	-8.7 (-12.2 to -5.2)	<0.001
	2013 - 2021	-2.3 (-2.8 to -1.7)	<0.001
Barbados	1990 - 1997	0.5 (-0.9 to 2)	0.446
	1997 - 2007	-4.7 (-5.7 to -3.7)	<0.001
	2007 - 2021	0.6 (-0.2 to 1.4)	0.152
Belarus	1990 - 1993	10.7 (7 to 14.5)	<0.001
	1993 - 2001	4.4 (3.6 to 5.3)	<0.001
	2001 - 2011	-0.8 (-1.4 to -0.3)	0.006
	2011 - 2017	-10.5 (-12 to -8.9)	<0.001
	2017 - 2021	-2.5 (-6.1 to 1.3)	0.186
Belgium	1990 - 1995	4.9 (2.7 to 7.1)	<0.001
	1995 - 2006	-3.8 (-4.5 to -3.1)	<0.001
	2006 - 2011	1.1 (-1.9 to 4.2)	0.45
	2011 - 2021	-3.2 (-4 to -2.3)	<0.001
Belize	1990 - 1998	7.5 (6 to 9.1)	<0.001
	1998 - 2021	-2.1 (-2.4 to -1.8)	<0.001
Benin	1990 - 1999	1.1 (0.9 to 1.2)	<0.001
	1999 - 2006	-1.9 (-2.2 to -1.5)	<0.001
	2006 - 2013	1.7 (1.3 to 2.1)	<0.001
	2013 - 2021	-1.5 (-1.8 to -1.2)	<0.001

Bermuda	1990 - 2006	-4.7 (-5.1 to -4.4)	<0.001
	2006 - 2011	0.6 (-2.3 to 3.7)	0.665
	2011 - 2021	-2.8 (-3.7 to -1.8)	<0.001
Bhutan	1990 - 1997	0 (-0.5 to 0.5)	0.951
	1997 - 2014	-1.1 (-1.3 to -0.9)	<0.001
	2014 - 2021	-2.3 (-3.1 to -1.6)	<0.001
Bolivia (Plurinational State of)	1990 - 1994	0.2 (-0.1 to 0.6)	0.173
	1994 - 2000	0.7 (0.5 to 1)	<0.001
	2000 - 2010	-0.5 (-0.6 to -0.4)	<0.001
	2010 - 2018	-1.6 (-1.8 to -1.4)	<0.001
	2018 - 2021	-2.3 (-3 to -1.5)	<0.001
Bosnia and Herzegovina	1990 - 1992	14.7 (3.6 to 27.1)	0.011
	1992 - 1997	-8.7 (-12.8 to -4.4)	0.001
	1997 - 2013	-2.2 (-2.7 to -1.6)	<0.001
	2013 - 2018	1.4 (-2 to 5)	0.4
	2018 - 2021	-12.6 (-17.9 to -7)	<0.001
Botswana	1990 - 1994	2.9 (2.3 to 3.5)	<0.001
	1994 - 2002	0.1 (-0.2 to 0.4)	0.356
	2002 - 2006	-5.1 (-6 to -4.2)	<0.001
	2006 - 2011	2 (1.5 to 2.5)	<0.001
	2011 - 2014	-2.7 (-4.1 to -1.2)	0.002
	2014 - 2017	-5.8 (-7.2 to -4.5)	<0.001
	2017 - 2021	-2.4 (-2.9 to -1.9)	<0.001
Brazil	1990 - 1994	2.5 (0.8 to 4.2)	0.006
	1994 - 1997	-1.7 (-6.6 to 3.5)	0.495
	1997 - 2013	0.4 (0.2 to 0.7)	<0.001
	2013 - 2019	6.6 (5.5 to 7.8)	<0.001
	2019 - 2021	-2.5 (-7.2 to 2.5)	0.306
Brunei Darussalam	1990 - 2001	-4.4 (-5.9 to -3)	<0.001
	2001 - 2013	-0.4 (-1.5 to 0.8)	0.483
	2013 - 2021	-3.8 (-5.7 to -1.8)	0.001
Bulgaria	1990 - 1997	2.8 (1.5 to 4.1)	<0.001
	1997 - 2003	-6.2 (-8.1 to -4.2)	<0.001
	2003 - 2012	-1.1 (-2.2 to 0.1)	0.062
	2012 - 2021	-5 (-6.1 to -3.9)	<0.001
Burkina Faso	1990 - 1992	-1.9 (-5 to 1.2)	0.212
	1992 - 2000	0.1 (-0.4 to 0.5)	0.78
	2000 - 2010	-1 (-1.3 to -0.7)	<0.001
	2010 - 2016	2.6 (1.9 to 3.4)	<0.001
	2016 - 2021	-2.3 (-3.1 to -1.5)	<0.001
Burundi	1990 - 1996	-0.8 (-1.4 to -0.1)	0.019
	1996 - 2008	-3 (-3.3 to -2.8)	<0.001
	2008 - 2011	0.4 (-4 to 4.9)	0.858

	2011 - 2021	-1.8 (-2.2 to -1.5)	<0.001
Cabo Verde	1990 - 2004	1 (0.7 to 1.3)	<0.001
	2004 - 2007	-3.2 (-9.1 to 3.2)	0.306
	2007 - 2016	-0.3 (-1 to 0.4)	0.363
	2016 - 2021	2.5 (0.9 to 4.1)	0.004
Cambodia	1990 - 1998	-0.8 (-1.1 to -0.5)	<0.001
	1998 - 2006	-1.9 (-2.3 to -1.6)	<0.001
	2006 - 2021	-1.1 (-1.2 to -0.9)	<0.001
Cameroon	1990 - 1992	4.1 (2.8 to 5.3)	<0.001
	1992 - 2000	2.5 (2.3 to 2.7)	<0.001
	2000 - 2003	0.3 (-1 to 1.6)	0.63
	2003 - 2006	-0.5 (-1.9 to 0.9)	0.435
	2006 - 2010	1.4 (0.7 to 2.1)	0.001
	2010 - 2014	-0.6 (-1.4 to 0.1)	0.087
	2014 - 2021	-2.3 (-2.5 to -2.1)	<0.001
Canada	1990 - 1995	-0.1 (-1 to 0.8)	0.806
	1995 - 2007	-2.7 (-3 to -2.5)	<0.001
	2007 - 2013	1 (0.1 to 1.9)	0.035
	2013 - 2018	3.4 (2.1 to 4.7)	<0.001
	2018 - 2021	-1.2 (-3.8 to 1.5)	0.353
Central African Republic	1990 - 2021	-0.4 (-0.5 to -0.3)	<0.001
Chad	1990 - 1995	1.4 (0.2 to 2.6)	0.023
	1995 - 2001	4.8 (3.6 to 6)	<0.001
	2001 - 2021	0.2 (0.1 to 0.3)	0.008
Chile	1990 - 2003	-4.5 (-4.9 to -4.1)	<0.001
	2003 - 2009	4.7 (3.4 to 6)	<0.001
	2009 - 2013	-7.3 (-10.1 to -4.5)	<0.001
	2013 - 2021	-0.8 (-1.6 to 0)	0.049
China	1990 - 1993	-1.5 (-3.2 to 0.2)	0.074
	1993 - 2001	-6.4 (-6.8 to -6.1)	<0.001
	2001 - 2004	-5.3 (-6.9 to -3.6)	<0.001
	2004 - 2007	-7.7 (-8.9 to -6.5)	<0.001
	2007 - 2010	-2.6 (-4.2 to -0.9)	0.006
	2010 - 2013	-7.2 (-9.3 to -5)	<0.001
	2013 - 2021	-1.8 (-2 to -1.5)	<0.001
Colombia	1990 - 1993	1.7 (-1.8 to 5.4)	0.315
	1993 - 1996	13.5 (5.9 to 21.6)	0.001
	1996 - 2008	0 (-0.4 to 0.5)	0.968
	2008 - 2014	-2.4 (-3.8 to -1.1)	0.002
	2014 - 2018	8.9 (5.3 to 12.5)	<0.001
	2018 - 2021	-3.7 (-8.9 to 1.7)	0.159
Comoros	1990 - 2008	-3 (-4 to -2)	<0.001
	2008 - 2011	-21.1 (-46.9 to 17.4)	0.229
	2011 - 2014	36.2 (-10.1 to 106.4)	0.137

	2014 - 2021	-0.9 (-4.3 to 2.7)	0.61
Congo	1990 - 1997	3.1 (1.8 to 4.4)	<0.001
	1997 - 2002	-4.3 (-7 to -1.4)	0.005
	2002 - 2006	1 (-3 to 5.2)	0.61
	2006 - 2021	-1.1 (-1.5 to -0.7)	<0.001
Cook Islands	1990 - 1997	-2.9 (-3.1 to -2.7)	<0.001
	1997 - 2000	-1.7 (-2.8 to -0.5)	0.009
	2000 - 2006	-3.3 (-3.6 to -3.1)	<0.001
	2006 - 2009	-1.3 (-2.3 to -0.2)	0.02
	2009 - 2012	1.3 (0.1 to 2.5)	0.03
	2012 - 2015	0.3 (-0.9 to 1.5)	0.599
	2015 - 2021	-1.2 (-1.5 to -1)	<0.001
Costa Rica	1990 - 1993	-0.4 (-8.8 to 8.7)	0.922
	1993 - 1996	12.6 (-4.4 to 32.6)	0.146
	1996 - 2004	1.5 (-0.6 to 3.6)	0.157
	2004 - 2008	-4.3 (-11.4 to 3.4)	0.248
	2008 - 2021	2.5 (1.6 to 3.3)	<0.001
Côte d'Ivoire	1990 - 1995	2.8 (2.1 to 3.4)	<0.001
	1995 - 1999	1.2 (-0.2 to 2.6)	0.08
	1999 - 2006	-0.6 (-1.1 to -0.1)	0.015
	2006 - 2011	-2.1 (-3 to -1.2)	<0.001
	2011 - 2014	3.8 (0.9 to 6.7)	0.014
	2014 - 2017	-1.6 (-4.2 to 1.1)	0.227
	2017 - 2021	-3 (-4.1 to -1.9)	<0.001
Croatia	1990 - 1992	17.3 (5.7 to 30.2)	0.005
	1992 - 2005	-3.6 (-4.3 to -2.9)	<0.001
	2005 - 2014	-2 (-3.2 to -0.7)	0.003
	2014 - 2021	-5.9 (-7.9 to -3.8)	<0.001
Cuba	1990 - 1992	-0.5 (-9.4 to 9.4)	0.919
	1992 - 1999	-7.5 (-9.1 to -5.8)	<0.001
	1999 - 2002	-13.6 (-23.2 to -2.8)	0.018
	2002 - 2007	-5.7 (-9.6 to -1.7)	0.008
	2007 - 2010	4.7 (-7.8 to 18.9)	0.457
	2010 - 2021	-0.8 (-1.8 to 0.3)	0.14
Cyprus	1990 - 1997	3.9 (2.2 to 5.6)	<0.001
	1997 - 2007	-1.5 (-2.5 to -0.6)	0.004
	2007 - 2013	-4 (-6 to -2)	0.001
	2013 - 2017	0.9 (-3.6 to 5.7)	0.679
	2017 - 2021	-6.5 (-9.7 to -3.2)	0.001
Czechia	1990 - 1994	3.4 (0.3 to 6.6)	0.035
	1994 - 2005	-1.8 (-2.5 to -1.1)	<0.001
	2005 - 2012	0.7 (-0.8 to 2.3)	0.342
	2012 - 2015	-7.5 (-16.1 to 2)	0.109
	2015 - 2018	0.6 (-8.8 to 11)	0.891

	2018 - 2021	-12.7 (-18.1 to -6.8)	<0.001
Democratic People's Republic of Korea	1990 - 1994	-1 (-1.4 to -0.7)	<0.001
	1994 - 2000	-1.8 (-2 to -1.5)	<0.001
	2000 - 2005	-3.1 (-3.4 to -2.7)	<0.001
	2005 - 2010	1 (0.6 to 1.3)	<0.001
	2010 - 2014	-2.6 (-3.1 to -2.1)	<0.001
	2014 - 2021	-0.9 (-1 to -0.7)	<0.001
Democratic Republic of the Congo	1990 - 1994	-2.2 (-3.6 to -0.8)	0.004
	1994 - 2010	-0.2 (-0.4 to 0)	0.041
	2010 - 2014	1.8 (-0.8 to 4.5)	0.175
	2014 - 2021	-0.5 (-1.2 to 0.2)	0.177
Denmark	1990 - 2000	-1.8 (-2.6 to -1)	<0.001
	2000 - 2006	-5.4 (-7.6 to -3.2)	<0.001
	2006 - 2016	-2.4 (-3.3 to -1.5)	<0.001
	2016 - 2021	0.9 (-2.2 to 4)	0.559
Djibouti	1990 - 2001	2.9 (1.8 to 4)	<0.001
	2001 - 2021	-1 (-1.5 to -0.5)	<0.001
Dominica	1990 - 1994	0.3 (-0.1 to 0.8)	0.154
	1994 - 1999	-1.6 (-2.1 to -1.2)	<0.001
	1999 - 2002	-2.6 (-4.1 to -1)	0.004
	2002 - 2006	-1.3 (-2.1 to -0.5)	0.002
	2006 - 2012	2.2 (1.9 to 2.6)	<0.001
	2012 - 2021	0.8 (0.7 to 1)	<0.001
Dominican Republic	1990 - 2011	0.2 (0 to 0.4)	0.078
	2011 - 2017	6.5 (4 to 9)	<0.001
	2017 - 2021	-9.6 (-13.1 to -6)	<0.001
Ecuador	1990 - 2002	3.9 (3.1 to 4.7)	<0.001
	2002 - 2005	8.7 (-10.1 to 31.4)	0.37
	2005 - 2019	-0.6 (-1.4 to 0.3)	0.175
	2019 - 2021	-11.5 (-33.8 to 18.4)	0.393
Egypt	1990 - 1992	-7.1 (-12.3 to -1.7)	0.013
	1992 - 1996	2.1 (-0.6 to 4.8)	0.115
	1996 - 2000	-2.2 (-4.7 to 0.4)	0.088
	2000 - 2015	0.5 (0.3 to 0.8)	<0.001
	2015 - 2021	-2.7 (-3.5 to -1.8)	<0.001
El Salvador	1990 - 2013	-2.4 (-2.7 to -2.1)	<0.001
	2013 - 2016	10.1 (-7 to 30.4)	0.252
	2016 - 2021	-8 (-12.4 to -3.4)	0.002
Equatorial Guinea	1990 - 1996	-1.6 (-2.4 to -0.8)	<0.001
	1996 - 2002	-4.9 (-6 to -3.8)	<0.001
	2002 - 2014	-0.8 (-1.3 to -0.4)	0.001
	2014 - 2021	2.6 (1.6 to 3.5)	<0.001

Eritrea	1990 - 1993	-0.6 (-1.6 to 0.4)	0.255
	1993 - 1996	2.7 (0.3 to 5)	0.026
	1996 - 2001	-1.4 (-2.1 to -0.7)	0.001
	2001 - 2004	1.2 (-1.4 to 3.8)	0.339
	2004 - 2021	-0.6 (-0.7 to -0.5)	<0.001
Estonia	1990 - 1994	6.1 (2.1 to 10.2)	0.005
	1994 - 2008	-2.8 (-3.4 to -2.2)	<0.001
	2008 - 2011	-8.8 (-18 to 1.4)	0.085
	2011 - 2014	0.3 (-9.9 to 11.5)	0.957
	2014 - 2017	-12.2 (-22.5 to -0.5)	0.043
	2017 - 2021	-0.9 (-5.5 to 4)	0.702
Eswatini	1990 - 1994	1.6 (1 to 2.2)	<0.001
	1994 - 1998	5.8 (4.8 to 6.7)	<0.001
	1998 - 2003	9 (8.3 to 9.6)	<0.001
	2003 - 2009	0.3 (-0.1 to 0.8)	0.136
	2009 - 2021	-1.9 (-2.1 to -1.8)	<0.001
Ethiopia	1990 - 1996	-1.4 (-1.7 to -1.2)	<0.001
	1996 - 2007	-4 (-4.1 to -3.9)	<0.001
	2007 - 2010	-3 (-3.8 to -2.2)	<0.001
	2010 - 2015	-1.7 (-1.9 to -1.5)	<0.001
	2015 - 2021	-0.2 (-0.3 to 0)	0.051
Fiji	1990 - 1998	-1.7 (-2.3 to -1.1)	<0.001
	1998 - 2001	3 (-1.4 to 7.7)	0.174
	2001 - 2009	-1.8 (-2.3 to -1.3)	<0.001
	2009 - 2012	4.1 (0 to 8.4)	0.051
	2012 - 2021	-1.4 (-2 to -0.8)	<0.001
Finland	1990 - 1992	-9.5 (-18 to -0.1)	0.048
	1992 - 2006	-1.9 (-2.4 to -1.4)	<0.001
	2006 - 2011	0.5 (-3 to 4.1)	0.763
	2011 - 2014	-8.5 (-17.1 to 1)	0.076
	2014 - 2021	1.4 (-0.4 to 3.2)	0.115
France	1990 - 1993	3.5 (1.8 to 5.3)	0.001
	1993 - 2000	-5.2 (-5.7 to -4.6)	<0.001
	2000 - 2003	-1 (-4.3 to 2.4)	0.522
	2003 - 2007	-3.9 (-5.4 to -2.3)	<0.001
	2007 - 2010	0.9 (-2.4 to 4.4)	0.565
	2010 - 2016	-5.6 (-6.3 to -4.9)	<0.001
	2016 - 2021	-1 (-2.2 to 0.3)	0.111
Gabon	1990 - 1998	1.3 (0.5 to 2.1)	0.002
	1998 - 2021	-0.7 (-0.9 to -0.5)	<0.001
Gambia	1990 - 1999	2.1 (1 to 3.3)	0.001
	1999 - 2002	-3 (-13.6 to 9)	0.589
	2002 - 2005	7.7 (-3.4 to 20.1)	0.167
	2005 - 2012	-4.2 (-6.1 to -2.2)	0.001

	2012 - 2017	5.4 (1.2 to 9.8)	0.015
	2017 - 2021	0.4 (-3.2 to 4.2)	0.817
Georgia	1990 - 1997	5.5 (3.7 to 7.4)	<0.001
	1997 - 2005	-8.4 (-10.2 to -6.6)	<0.001
	2005 - 2009	12.8 (5.4 to 20.7)	0.001
	2009 - 2021	0.2 (-0.6 to 1)	0.564
Germany	1990 - 2001	-1.8 (-2.1 to -1.5)	<0.001
	2001 - 2007	-3.5 (-4.3 to -2.6)	<0.001
	2007 - 2010	2.1 (-2 to 6.3)	0.298
	2010 - 2013	-3.3 (-7.1 to 0.6)	0.093
	2013 - 2016	1.3 (-2.3 to 5.1)	0.447
	2016 - 2021	-3.1 (-4.2 to -1.9)	<0.001
Ghana	1990 - 1995	0 (-0.5 to 0.5)	0.963
	1995 - 2000	1 (0.3 to 1.6)	0.005
	2000 - 2004	3.3 (2.3 to 4.2)	<0.001
	2004 - 2008	-1.7 (-2.6 to -0.8)	0.001
	2008 - 2017	1.6 (1.4 to 1.8)	<0.001
	2017 - 2021	-3 (-3.7 to -2.2)	<0.001
Greece	1990 - 1992	-7 (-16.5 to 3.7)	0.181
	1992 - 1997	1.4 (-2.2 to 5.2)	0.417
	1997 - 2008	-2.4 (-3.2 to -1.5)	<0.001
	2008 - 2012	4.7 (-1.9 to 11.7)	0.158
	2012 - 2021	-0.6 (-1.8 to 0.7)	0.343
Greenland	1990 - 2000	-4.6 (-4.8 to -4.4)	<0.001
	2000 - 2006	-1.4 (-1.9 to -0.9)	<0.001
	2006 - 2012	-0.1 (-0.6 to 0.5)	0.842
	2012 - 2021	-1.8 (-2 to -1.6)	<0.001
Grenada	1990 - 1994	-6.2 (-8.4 to -4)	<0.001
	1994 - 2000	-2.6 (-4.1 to -1)	0.002
	2000 - 2008	2.2 (1.2 to 3.2)	<0.001
	2008 - 2014	-6.1 (-7.6 to -4.6)	<0.001
	2014 - 2021	1 (-0.2 to 2.3)	0.089
Guam	1990 - 1994	1.7 (-2 to 5.5)	0.343
	1994 - 1999	-5.3 (-8.3 to -2.3)	0.003
	1999 - 2007	-1.4 (-2.6 to -0.1)	0.032
	2007 - 2012	7.2 (4.3 to 10.2)	<0.001
	2012 - 2015	-5.3 (-13.6 to 3.8)	0.221
	2015 - 2018	11.4 (-0.8 to 25.1)	0.065
	2018 - 2021	-16.2 (-21.3 to -10.8)	<0.001
Guatemala	1990 - 1998	0.9 (-0.5 to 2.3)	0.202
	1998 - 2001	25 (9.9 to 42.2)	0.002
	2001 - 2012	-4.7 (-5.6 to -3.8)	<0.001
	2012 - 2019	2.7 (0.8 to 4.7)	0.008
	2019 - 2021	-11.5 (-26.2 to 6.3)	0.179

Guinea	1990 - 2007	1.7 (1.6 to 1.8)	<0.001
	2007 - 2015	0.5 (0 to 0.9)	0.034
	2015 - 2021	-1.4 (-2 to -0.8)	<0.001
Guinea-Bissau	1990 - 1997	0.6 (0.3 to 0.9)	<0.001
	1997 - 2001	1.6 (0.6 to 2.6)	0.003
	2001 - 2008	0.6 (0.3 to 0.9)	0.001
	2008 - 2018	-0.8 (-1 to -0.7)	<0.001
	2018 - 2021	-2 (-2.8 to -1.1)	<0.001
Guyana	1990 - 1995	-4.8 (-7.6 to -2)	0.002
	1995 - 1999	11.9 (4.3 to 20.2)	0.004
	1999 - 2009	-2.3 (-3.1 to -1.6)	<0.001
	2009 - 2014	5.9 (3.1 to 8.7)	<0.001
	2014 - 2021	-1.8 (-3.5 to 0)	0.054
Haiti	1990 - 2021	-0.5 (-0.7 to -0.3)	<0.001
Honduras	1990 - 1997	-1.4 (-1.6 to -1.2)	<0.001
	1997 - 2003	-0.5 (-0.8 to -0.1)	0.02
	2003 - 2009	-2.9 (-3.4 to -2.5)	<0.001
	2009 - 2012	-6.2 (-8.1 to -4.3)	<0.001
	2012 - 2021	-0.6 (-0.8 to -0.4)	<0.001
Hungary	1990 - 1993	-0.4 (-4.2 to 3.6)	0.843
	1993 - 1996	-6.1 (-12.9 to 1.2)	0.094
	1996 - 1999	2.3 (-4.8 to 10)	0.505
	1999 - 2006	-5 (-6.2 to -3.8)	<0.001
	2006 - 2009	4.1 (-3 to 11.7)	0.247
	2009 - 2021	-3.4 (-3.9 to -2.8)	<0.001
Iceland	1990 - 1995	-5.9 (-8.4 to -3.4)	<0.001
	1995 - 2000	2.2 (-1.5 to 6.1)	0.225
	2000 - 2012	-3.2 (-4 to -2.4)	<0.001
	2012 - 2015	3.3 (-8.1 to 16.1)	0.564
	2015 - 2019	-6.7 (-12.8 to -0.1)	0.047
	2019 - 2021	7.5 (-8.4 to 26.2)	0.35
India	1990 - 1996	-0.6 (-2 to 0.7)	0.311
	1996 - 1999	2.9 (-4.2 to 10.4)	0.403
	1999 - 2002	-4.9 (-10.7 to 1.2)	0.104
	2002 - 2005	1.2 (-3.8 to 6.5)	0.617
	2005 - 2012	-2.3 (-3.2 to -1.4)	<0.001
	2012 - 2019	-5.4 (-6.2 to -4.5)	<0.001
	2019 - 2021	2.9 (-3 to 9.2)	0.307
Indonesia	1990 - 2003	0.2 (0.2 to 0.3)	<0.001
	2003 - 2011	-1.1 (-1.3 to -0.9)	<0.001
	2011 - 2014	0.3 (-1.3 to 2)	0.715
	2014 - 2021	-1 (-1.3 to -0.8)	<0.001
Iran (Islamic Republic of)	1990 - 1995	-1.3 (-2.2 to -0.4)	0.009
	1995 - 2001	0.5 (-0.3 to 1.3)	0.182

	2001 - 2011	-0.5 (-0.8 to -0.3)	<0.001
	2011 - 2019	-3.6 (-4 to -3.3)	<0.001
	2019 - 2021	-5.6 (-8.1 to -3.1)	<0.001
Iraq	1990 - 1996	1.6 (1.2 to 1.9)	<0.001
	1996 - 2005	-2.1 (-2.3 to -1.9)	<0.001
	2005 - 2008	-3.7 (-5.7 to -1.7)	0.001
	2008 - 2017	-0.8 (-1.1 to -0.6)	<0.001
	2017 - 2021	-3.7 (-4.3 to -3.1)	<0.001
Ireland	1990 - 1993	2.6 (-0.6 to 5.8)	0.103
	1993 - 1997	10.8 (7.8 to 13.9)	<0.001
	1997 - 2006	-0.8 (-1.3 to -0.2)	0.01
	2006 - 2011	-3.6 (-5 to -2.2)	<0.001
	2011 - 2014	-11.5 (-15.9 to -6.8)	<0.001
	2014 - 2021	-6.1 (-7.3 to -4.9)	<0.001
Israel	1990 - 1997	4.5 (2.8 to 6.4)	<0.001
	1997 - 2009	-4 (-4.8 to -3.1)	<0.001
	2009 - 2012	-10 (-20.5 to 2)	0.095
	2012 - 2021	-0.9 (-2.4 to 0.6)	0.208
Italy	1990 - 1997	3.5 (2.2 to 4.8)	<0.001
	1997 - 2005	-6.4 (-7.5 to -5.3)	<0.001
	2005 - 2017	0.3 (-0.4 to 1)	0.465
	2017 - 2021	-6.3 (-10 to -2.5)	0.003
Jamaica	1990 - 2000	2.5 (0.9 to 4.1)	0.004
	2000 - 2006	-11.3 (-13.4 to -9.2)	<0.001
	2006 - 2009	22.6 (5.3 to 42.8)	0.011
	2009 - 2014	-4.5 (-7.9 to -1)	0.016
	2014 - 2021	2.1 (-0.7 to 5)	0.131
Japan	1990 - 1996	2.8 (1.6 to 3.9)	<0.001
	1996 - 1999	9.6 (3.8 to 15.6)	0.003
	1999 - 2002	-2.6 (-7.6 to 2.7)	0.298
	2002 - 2006	5.3 (2.7 to 8.1)	0.001
	2006 - 2011	0.7 (-0.7 to 2.1)	0.314
	2011 - 2016	-3.6 (-5 to -2.3)	<0.001
	2016 - 2021	2.5 (1.3 to 3.8)	0.001
Jordan	1990 - 1999	0.4 (0 to 0.8)	0.034
	1999 - 2004	-3 (-4.2 to -1.7)	<0.001
	2004 - 2009	-11.4 (-12.3 to -10.6)	<0.001
	2009 - 2012	-4.2 (-6.8 to -1.6)	0.005
	2012 - 2015	-1.3 (-4.1 to 1.6)	0.351
	2015 - 2018	-5 (-7.9 to -2)	0.004
	2018 - 2021	-0.4 (-2.1 to 1.5)	0.681
Kazakhstan	1990 - 1993	14.1 (8.2 to 20.3)	<0.001
	1993 - 2007	2 (1.5 to 2.6)	<0.001
	2007 - 2021	-6.9 (-7.3 to -6.4)	<0.001

Kenya	1990 - 1996	-0.8 (-1.2 to -0.4)	0.001
	1996 - 1999	0.8 (-1.5 to 3.2)	0.459
	1999 - 2004	3.3 (2.6 to 4)	<0.001
	2004 - 2011	0.6 (0.4 to 0.9)	<0.001
	2011 - 2015	-1.8 (-2.5 to -1)	<0.001
	2015 - 2021	-1 (-1.3 to -0.7)	<0.001
Kiribati	1990 - 1993	-0.2 (-0.5 to 0)	0.074
	1993 - 1999	-0.8 (-0.9 to -0.7)	<0.001
	1999 - 2004	-0.4 (-0.6 to -0.2)	<0.001
	2004 - 2011	1 (0.9 to 1.1)	<0.001
	2011 - 2015	-1 (-1.3 to -0.6)	<0.001
	2015 - 2021	-1.2 (-1.3 to -1.1)	<0.001
Kuwait	1990 - 1998	6.3 (3.4 to 9.3)	<0.001
	1998 - 2002	16.4 (4 to 30.3)	0.011
	2002 - 2005	-8.7 (-24.8 to 11)	0.341
	2005 - 2008	-33.2 (-46.8 to -16.3)	0.001
	2008 - 2021	5 (3.4 to 6.6)	<0.001
Kyrgyzstan	1990 - 2001	1.1 (0.6 to 1.6)	<0.001
	2001 - 2004	-7.3 (-13.7 to -0.5)	0.037
	2004 - 2012	1.1 (0.1 to 2.1)	0.027
	2012 - 2021	-4.5 (-5.2 to -3.7)	<0.001
Lao People's Democratic Republic	1990 - 1994	-0.9 (-2 to 0.2)	0.095
	1994 - 2012	-2.1 (-2.2 to -2)	<0.001
	2012 - 2021	-1.3 (-1.6 to -1.1)	<0.001
Latvia	1990 - 1994	8.6 (6.3 to 11)	<0.001
	1994 - 2003	-4.4 (-5.2 to -3.7)	<0.001
	2003 - 2012	-1 (-1.8 to -0.2)	0.022
	2012 - 2021	-6.4 (-7.1 to -5.6)	<0.001
Lebanon	1990 - 2003	-3.5 (-3.7 to -3.4)	<0.001
	2003 - 2010	-0.3 (-0.7 to 0.1)	0.108
	2010 - 2015	1.5 (0.8 to 2.2)	<0.001
	2015 - 2018	-6.7 (-8.5 to -4.7)	<0.001
	2018 - 2021	-2.4 (-3.4 to -1.4)	<0.001
Lesotho	1990 - 1993	-2.4 (-4.1 to -0.8)	0.007
	1993 - 1997	2 (0.5 to 3.6)	0.014
	1997 - 2000	12.3 (9 to 15.8)	<0.001
	2000 - 2005	7.5 (6.5 to 8.4)	<0.001
	2005 - 2015	1 (0.8 to 1.2)	<0.001
	2015 - 2021	0 (-0.4 to 0.4)	0.922
Liberia	1990 - 1992	-7.1 (-14.4 to 0.9)	0.079
	1992 - 1999	3.6 (1.9 to 5.3)	<0.001
	1999 - 2006	-0.9 (-2.9 to 1.1)	0.365
	2006 - 2013	4.3 (2.1 to 6.5)	0.001

	2013 - 2021	-2.2 (-3.5 to -0.9)	0.003
Libya	1990 - 1996	-0.4 (-1.4 to 0.6)	0.392
	1996 - 2003	2.2 (1.3 to 3.1)	<0.001
	2003 - 2006	-7.2 (-11.8 to -2.4)	0.006
	2006 - 2010	8.9 (6.2 to 11.8)	<0.001
	2010 - 2021	-0.7 (-1.1 to -0.3)	0.001
Lithuania	1990 - 1994	14.6 (9.3 to 20.1)	<0.001
	1994 - 2014	-0.7 (-1.1 to -0.3)	0.001
	2014 - 2017	-14.1 (-26.1 to 0)	0.049
	2017 - 2021	-3.9 (-10.2 to 2.8)	0.233
Luxembourg	1990 - 1994	-1.4 (-6.1 to 3.5)	0.542
	1994 - 1999	-8.9 (-12.5 to -5.2)	<0.001
	1999 - 2004	-1.1 (-5.2 to 3.3)	0.597
	2004 - 2008	-9.6 (-15.8 to -2.9)	0.009
	2008 - 2011	1.8 (-10.6 to 16)	0.765
	2011 - 2015	-9.4 (-15.3 to -3.1)	0.008
	2015 - 2021	1.6 (-1.1 to 4.3)	0.217
Madagascar	1990 - 2006	-0.9 (-1 to -0.8)	<0.001
	2006 - 2021	0.2 (0 to 0.3)	0.038
Malawi	1990 - 2000	1.6 (1.3 to 1.8)	<0.001
	2000 - 2004	-0.4 (-1.7 to 0.9)	0.489
	2004 - 2008	-2.5 (-3.7 to -1.3)	0.001
	2008 - 2011	-0.7 (-3 to 1.5)	0.493
	2011 - 2014	-2.5 (-4.8 to -0.2)	0.038
	2014 - 2019	0 (-0.8 to 0.9)	0.967
	2019 - 2021	-1.8 (-4.7 to 1.3)	0.236
Malaysia	1990 - 2009	-2.3 (-3 to -1.7)	<0.001
	2009 - 2021	-0.2 (-1.6 to 1.3)	0.797
Maldives	1990 - 1997	-5.4 (-6.2 to -4.6)	<0.001
	1997 - 2000	8.7 (2 to 15.9)	0.013
	2000 - 2006	-4.2 (-5.6 to -2.8)	<0.001
	2006 - 2014	-0.6 (-1.4 to 0.2)	0.123
	2014 - 2021	-3.7 (-4.6 to -2.8)	<0.001
Mali	1990 - 1996	0.4 (0.1 to 0.8)	0.028
	1996 - 1999	-1.1 (-3.4 to 1.2)	0.304
	1999 - 2002	1.3 (-1.2 to 3.7)	0.294
	2002 - 2006	-0.7 (-1.9 to 0.5)	0.238
	2006 - 2017	0.5 (0.3 to 0.7)	<0.001
	2017 - 2021	-1.4 (-2.1 to -0.6)	0.002
Malta	1990 - 2013	-0.7 (-1.1 to -0.3)	0.001
	2013 - 2019	-8 (-11.6 to -4.3)	<0.001
	2019 - 2021	25.4 (2.4 to 53.5)	0.03
Marshall Islands	1990 - 1995	-5 (-6.7 to -3.3)	<0.001
	1995 - 2005	1.5 (0.8 to 2.2)	<0.001

	2005 - 2021	-0.7 (-1 to -0.4)	<0.001
Mauritania	1990 - 2018	-0.9 (-1 to -0.8)	<0.001
	2018 - 2021	1.3 (-1.3 to 4.1)	0.318
Mauritius	1990 - 1997	-1.2 (-2.6 to 0.3)	0.111
	1997 - 2004	-9.1 (-10.9 to -7.3)	<0.001
	2004 - 2021	1.9 (1.3 to 2.4)	<0.001
Mexico	1990 - 1993	4 (1.2 to 6.9)	0.008
	1993 - 1997	8.1 (5.7 to 10.6)	<0.001
	1997 - 2007	0.6 (0.1 to 1)	0.013
	2007 - 2011	7.3 (4.9 to 9.7)	<0.001
	2011 - 2021	0.9 (0.5 to 1.4)	<0.001
Micronesia (Federated States of)	1990 - 1994	-0.2 (-0.4 to 0)	0.069
	1994 - 1999	-1 (-1.1 to -0.8)	<0.001
	1999 - 2003	-1.4 (-1.6 to -1.1)	<0.001
	2003 - 2006	0.1 (-0.4 to 0.6)	0.758
	2006 - 2021	-0.2 (-0.2 to -0.2)	<0.001
Monaco	1990 - 1999	-0.1 (-0.2 to 0)	0.054
	1999 - 2008	-1.5 (-1.6 to -1.4)	<0.001
	2008 - 2012	-0.9 (-1.3 to -0.5)	<0.001
	2012 - 2017	-1.8 (-2.1 to -1.5)	<0.001
	2017 - 2021	-0.1 (-0.4 to 0.3)	0.618
Mongolia	1990 - 1994	8.6 (1.6 to 16)	0.018
	1994 - 2001	-6.5 (-9.8 to -3)	0.001
	2001 - 2012	4 (2.5 to 5.4)	<0.001
	2012 - 2021	-5 (-6.5 to -3.5)	<0.001
Montenegro	1990 - 1996	-3.6 (-7 to -0.1)	0.042
	1996 - 1999	23.9 (2.2 to 50.1)	0.031
	1999 - 2002	-12.9 (-27.1 to 4.2)	0.122
	2002 - 2005	3.1 (-12.8 to 22.1)	0.705
	2005 - 2021	-3 (-3.8 to -2.3)	<0.001
Morocco	1990 - 1993	0.9 (0.3 to 1.6)	0.006
	1993 - 2002	-0.6 (-0.7 to -0.4)	<0.001
	2002 - 2009	-1.9 (-2.1 to -1.7)	<0.001
	2009 - 2014	-3.1 (-3.5 to -2.7)	<0.001
	2014 - 2018	-4.2 (-4.7 to -3.6)	<0.001
	2018 - 2021	-1.8 (-2.5 to -1.1)	<0.001
Mozambique	1990 - 1993	-0.9 (-1.8 to 0)	0.048
	1993 - 1997	2 (1.1 to 3)	<0.001
	1997 - 2003	0.6 (0.2 to 1)	0.011
	2003 - 2009	3.8 (3.4 to 4.2)	<0.001
	2009 - 2015	1 (0.6 to 1.4)	<0.001
	2015 - 2021	-1.6 (-2 to -1.3)	<0.001
Myanmar	1990 - 1998	-0.3 (-0.5 to -0.1)	0.021

	1998 - 2005	-1.7 (-2.1 to -1.4)	<0.001
	2005 - 2009	-4.9 (-5.8 to -4)	<0.001
	2009 - 2012	-3 (-4.8 to -1.2)	0.003
	2012 - 2021	-0.8 (-1 to -0.6)	<0.001
Namibia	1990 - 1997	1.8 (1.5 to 2.2)	<0.001
	1997 - 2000	4 (1.5 to 6.6)	0.004
	2000 - 2003	1.6 (-0.9 to 4.2)	0.185
	2003 - 2012	-2.1 (-2.4 to -1.9)	<0.001
	2012 - 2015	0.8 (-2.1 to 3.9)	0.57
	2015 - 2021	-1.4 (-1.9 to -0.8)	<0.001
Nauru	1990 - 1994	0.4 (0 to 0.8)	0.037
	1994 - 1999	2.1 (1.7 to 2.5)	<0.001
	1999 - 2004	0.8 (0.4 to 1.3)	0.001
	2004 - 2009	-0.4 (-0.8 to 0)	0.069
	2009 - 2018	-2.1 (-2.3 to -2)	<0.001
	2018 - 2021	-0.8 (-1.4 to -0.1)	0.022
Nepal	1990 - 1994	-0.5 (-1 to 0)	0.037
	1994 - 1999	-1.6 (-2 to -1.1)	<0.001
	1999 - 2002	-2.4 (-3.6 to -1.2)	<0.001
	2002 - 2009	0.4 (0.2 to 0.6)	0.001
	2009 - 2021	-1 (-1.1 to -0.9)	<0.001
Netherlands	1990 - 1994	0.4 (-2.8 to 3.8)	0.779
	1994 - 2008	-2.6 (-3.1 to -2)	<0.001
	2008 - 2012	4.4 (-0.4 to 9.4)	0.072
	2012 - 2015	-3.6 (-11.3 to 4.7)	0.359
	2015 - 2018	6.3 (-3.4 to 17)	0.192
	2018 - 2021	-2.7 (-8.3 to 3.3)	0.346
New Zealand	1990 - 1996	2.7 (1 to 4.4)	0.004
	1996 - 2002	-6.4 (-8.4 to -4.4)	<0.001
	2002 - 2012	0.6 (-0.2 to 1.4)	0.124
	2012 - 2015	-4.7 (-12.1 to 3.2)	0.217
	2015 - 2018	2.7 (-6.3 to 12.6)	0.546
	2018 - 2021	-5.6 (-11.2 to 0.5)	0.067
Nicaragua	1990 - 1996	9.5 (8.1 to 10.9)	<0.001
	1996 - 2000	2.3 (-1.3 to 6.1)	0.196
	2000 - 2018	-1.8 (-2.1 to -1.6)	<0.001
	2018 - 2021	-9.3 (-13.1 to -5.4)	<0.001
Niger	1990 - 2003	0.1 (-0.1 to 0.3)	0.361
	2003 - 2010	-2.3 (-3 to -1.6)	<0.001
	2010 - 2014	2.6 (0.2 to 5)	0.036
	2014 - 2021	-0.5 (-1.1 to 0.2)	0.178
Nigeria	1990 - 2001	1 (0.9 to 1.1)	<0.001
	2001 - 2008	-1.9 (-2.1 to -1.6)	<0.001
	2008 - 2014	0.3 (0 to 0.7)	0.061

	2014 - 2021	-1.9 (-2.1 to -1.7)	<0.001
Niue	1990 - 2018	-0.7 (-1 to -0.4)	<0.001
	2018 - 2021	20.3 (11.9 to 29.3)	<0.001
North Macedonia	1990 - 1999	1.7 (0.5 to 2.9)	0.006
	1999 - 2021	-2.5 (-2.9 to -2.2)	<0.001
Northern Mariana Islands	1990 - 1999	-5.1 (-7.8 to -2.2)	0.001
	1999 - 2021	1.9 (1.2 to 2.5)	<0.001
Norway	1990 - 2007	-2.8 (-3.3 to -2.3)	<0.001
	2007 - 2010	3.4 (-9.9 to 18.5)	0.621
	2010 - 2013	-7.8 (-19.7 to 6)	0.24
	2013 - 2021	1.2 (-0.5 to 3)	0.15
Oman	1990 - 2008	-0.7 (-1.1 to -0.3)	0.003
	2008 - 2017	4.4 (3.1 to 5.8)	<0.001
	2017 - 2021	-19 (-22.3 to -15.6)	<0.001
Pakistan	1990 - 1995	3.9 (3.6 to 4.2)	<0.001
	1995 - 2003	0.9 (0.7 to 1.1)	<0.001
	2003 - 2011	-1.5 (-1.8 to -1.3)	<0.001
	2011 - 2015	0.5 (-0.3 to 1.4)	0.208
	2015 - 2021	-1.8 (-2.1 to -1.6)	<0.001
Palau	1990 - 1995	2.5 (0.8 to 4.2)	0.007
	1995 - 2014	0.2 (0 to 0.4)	0.062
	2014 - 2021	-1.6 (-2.2 to -0.9)	<0.001
Palestine	1990 - 2003	-0.7 (-0.8 to -0.5)	<0.001
	2003 - 2007	-3.7 (-4.8 to -2.6)	<0.001
	2007 - 2010	-9.2 (-11.2 to -7.2)	<0.001
	2010 - 2014	18.2 (17 to 19.4)	<0.001
	2014 - 2017	-9.2 (-10.8 to -7.6)	<0.001
	2017 - 2021	-0.4 (-1.1 to 0.3)	0.24
Panama	1990 - 1998	4.5 (3.3 to 5.8)	<0.001
	1998 - 2008	0.5 (-0.4 to 1.3)	0.277
	2008 - 2021	-1.9 (-2.5 to -1.3)	<0.001
Papua New Guinea	1990 - 2002	0.4 (-0.2 to 1)	0.152
	2002 - 2006	-2.4 (-8 to 3.5)	0.396
	2006 - 2021	1.4 (0.9 to 1.9)	<0.001
Paraguay	1990 - 1995	7.4 (5.8 to 9)	<0.001
	1995 - 1998	-0.8 (-6.7 to 5.4)	0.782
	1998 - 2004	4.2 (2.4 to 6)	<0.001
	2004 - 2019	1.4 (0.9 to 1.8)	<0.001
	2019 - 2021	-8.8 (-17.6 to 1)	0.073
Peru	1990 - 1994	3.7 (1.4 to 6)	0.004
	1994 - 1998	0.2 (-2.8 to 3.3)	0.887
	1998 - 2006	-2.2 (-3.1 to -1.2)	<0.001
	2006 - 2011	1.7 (-0.9 to 4.4)	0.18
	2011 - 2016	-3.1 (-5.8 to -0.4)	0.027

	2016 - 2019	8.5 (-1.3 to 19.3)	0.085
	2019 - 2021	-10.9 (-19.9 to -1)	0.035
Philippines	1990 - 1997	-3.6 (-4.3 to -2.8)	<0.001
	1997 - 2021	0.3 (0.2 to 0.4)	<0.001
Poland	1990 - 1999	2.6 (2 to 3.2)	<0.001
	1999 - 2014	0.3 (0 to 0.5)	0.03
	2014 - 2017	-7.5 (-12.4 to -2.3)	0.007
	2017 - 2021	-2.9 (-5.6 to -0.2)	0.039
Portugal	1990 - 2001	-3.5 (-4.1 to -2.9)	<0.001
	2001 - 2004	-14.3 (-22.9 to -4.7)	0.007
	2004 - 2009	1.5 (-1.6 to 4.7)	0.333
	2009 - 2013	-6.1 (-10.3 to -1.7)	0.009
	2013 - 2021	-1.1 (-2.4 to 0.2)	0.084
Puerto Rico	1990 - 1995	2.5 (-0.3 to 5.4)	0.076
	1995 - 2012	-2.9 (-3.4 to -2.4)	<0.001
	2012 - 2021	-4.8 (-6.4 to -3.2)	<0.001
Qatar	1990 - 1995	-2.5 (-4.6 to -0.4)	0.021
	1995 - 2001	7 (4.8 to 9.2)	<0.001
	2001 - 2018	-2.9 (-3.3 to -2.6)	<0.001
	2018 - 2021	-16.1 (-21 to -10.9)	<0.001
Republic of Korea	1990 - 1997	1.1 (-0.6 to 2.8)	0.191
	1997 - 2001	-5 (-7.3 to -2.6)	0.001
	2001 - 2006	1.4 (0.3 to 2.4)	0.017
	2006 - 2009	4.8 (-0.5 to 10.4)	0.074
	2009 - 2015	-3.1 (-4.3 to -1.8)	<0.001
	2015 - 2019	2.9 (-0.2 to 6)	0.062
	2019 - 2021	-3 (-9.2 to 3.5)	0.322
Republic of Moldova	1990 - 1994	2.7 (-0.1 to 5.7)	0.062
	1994 - 2006	-1.9 (-2.5 to -1.3)	<0.001
	2006 - 2010	7.4 (2.5 to 12.4)	0.005
	2010 - 2016	-3.2 (-5.2 to -1.2)	0.003
	2016 - 2021	-7.6 (-10 to -5.1)	<0.001
Romania	1990 - 1994	4.1 (0.8 to 7.5)	0.016
	1994 - 2007	-0.3 (-0.9 to 0.3)	0.385
	2007 - 2010	7.6 (-3.2 to 19.4)	0.163
	2010 - 2021	-5.6 (-6.5 to -4.8)	<0.001
Russian Federation	1990 - 1994	14.2 (10.8 to 17.7)	<0.001
	1994 - 2001	2.2 (0.1 to 4.3)	0.038
	2001 - 2008	-0.8 (-2.2 to 0.7)	0.282
	2008 - 2013	-5.3 (-8.1 to -2.4)	0.001
	2013 - 2021	-10.1 (-11.7 to -8.6)	<0.001
Rwanda	1990 - 1993	6.8 (5.2 to 8.5)	<0.001
	1993 - 1996	-4.8 (-8 to -1.5)	0.007
	1996 - 1999	-0.9 (-4.1 to 2.4)	0.562

	1999 - 2010	-5.7 (-6 to -5.5)	<0.001
	2010 - 2021	-1.1 (-1.4 to -0.8)	<0.001
Saint Kitts and Nevis	1990 - 1996	2.8 (0.8 to 4.9)	0.009
	1996 - 2002	-6.9 (-9.8 to -3.9)	<0.001
	2002 - 2021	-0.8 (-1.4 to -0.3)	0.004
Saint Lucia	1990 - 2002	-0.7 (-1.1 to -0.3)	0.001
	2002 - 2006	-3.4 (-6.7 to 0.1)	0.058
	2006 - 2012	3.5 (1.9 to 5.1)	<0.001
	2012 - 2021	-2.4 (-3.2 to -1.5)	<0.001
Saint Vincent and the Grenadines	1990 - 1997	-0.3 (-1.2 to 0.7)	0.526
	1997 - 2001	2.3 (-1.3 to 6.1)	0.2
	2001 - 2006	-3.5 (-5.8 to -1)	0.009
	2006 - 2009	2.7 (-5.3 to 11.4)	0.495
	2009 - 2014	-2.6 (-4.9 to -0.3)	0.032
	2014 - 2021	0.1 (-1 to 1.3)	0.804
Samoa	1990 - 1995	-0.9 (-1.1 to -0.8)	<0.001
	1995 - 2000	-2.2 (-2.4 to -1.9)	<0.001
	2000 - 2003	-3 (-3.8 to -2.1)	<0.001
	2003 - 2007	-0.9 (-1.4 to -0.5)	<0.001
	2007 - 2016	1.3 (1.2 to 1.4)	<0.001
	2016 - 2021	-1.2 (-1.4 to -1)	<0.001
San Marino	1990 - 1999	-0.7 (-0.9 to -0.6)	<0.001
	1999 - 2006	-3.4 (-3.7 to -3.2)	<0.001
	2006 - 2019	-0.5 (-0.6 to -0.4)	<0.001
	2019 - 2021	-8.8 (-10.7 to -6.9)	<0.001
Sao Tome and Principe	1990 - 2001	4.3 (2.6 to 6)	<0.001
	2001 - 2021	-0.6 (-1.1 to 0)	0.041
Saudi Arabia	1990 - 1998	-1 (-1.2 to -0.7)	<0.001
	1998 - 2002	1.2 (0.3 to 2)	0.011
	2002 - 2006	-1.8 (-2.4 to -1.1)	<0.001
	2006 - 2009	-4.2 (-5.6 to -2.8)	<0.001
	2009 - 2012	-2.4 (-4 to -0.7)	0.008
	2012 - 2017	0.1 (-0.4 to 0.7)	0.596
	2017 - 2021	-1.8 (-2.5 to -1.1)	<0.001
Senegal	1990 - 2021	-0.1 (-0.3 to 0.1)	0.296
Serbia	1990 - 1997	-0.9 (-1.8 to 0)	0.051
	1997 - 2003	-3.2 (-4.1 to -2.3)	<0.001
	2003 - 2008	-7.8 (-9.2 to -6.4)	<0.001
	2008 - 2016	-4.4 (-5.2 to -3.7)	<0.001
	2016 - 2019	0.3 (-6.6 to 7.7)	0.921
	2019 - 2021	-10 (-17.1 to -2.3)	0.016
Seychelles	1990 - 2012	-0.9 (-1.3 to -0.5)	<0.001
	2012 - 2019	8.1 (4.4 to 12)	<0.001

	2019 - 2021	-50.5 (-60.4 to -38.1)	<0.001
Sierra Leone	1990 - 1994	-1.4 (-2.8 to 0.1)	0.057
	1994 - 2000	1 (0 to 2.1)	0.058
	2000 - 2005	5.3 (3.8 to 6.9)	<0.001
	2005 - 2009	-1.6 (-3.7 to 0.6)	0.136
	2009 - 2017	1 (0.4 to 1.6)	0.002
	2017 - 2021	-1 (-2.5 to 0.5)	0.175
Singapore	1990 - 1995	-3.9 (-6.2 to -1.6)	0.003
	1995 - 2002	1.8 (0 to 3.6)	0.053
	2002 - 2005	-9.1 (-17.3 to -0.1)	0.048
	2005 - 2018	-0.1 (-0.7 to 0.5)	0.656
	2018 - 2021	5.6 (-0.5 to 12)	0.068
Slovakia	1990 - 1995	-1.4 (-2.9 to 0.1)	0.066
	1995 - 1998	2.6 (-2.7 to 8.2)	0.313
	1998 - 2001	-3.4 (-8.3 to 1.9)	0.186
	2001 - 2008	1.3 (0.3 to 2.3)	0.015
	2008 - 2019	-2.4 (-2.9 to -1.8)	<0.001
	2019 - 2021	-8.9 (-17.4 to 0.4)	0.059
Slovenia	1990 - 1999	0.1 (-1 to 1.3)	0.803
	1999 - 2016	-4.1 (-4.6 to -3.7)	<0.001
	2016 - 2021	-9.9 (-13.4 to -6.3)	<0.001
Solomon Islands	1990 - 2010	-0.7 (-0.9 to -0.5)	<0.001
	2010 - 2013	4 (-2 to 10.5)	0.185
	2013 - 2021	-0.2 (-0.9 to 0.4)	0.434
Somalia	1990 - 1992	-0.8 (-2.2 to 0.5)	0.207
	1992 - 1996	5.1 (4.4 to 5.8)	<0.001
	1996 - 2000	1.3 (0.6 to 2)	0.001
	2000 - 2006	-1.4 (-1.7 to -1.1)	<0.001
	2006 - 2009	0.8 (-0.5 to 2.2)	0.208
	2009 - 2015	-1.1 (-1.4 to -0.9)	<0.001
	2015 - 2021	-0.5 (-0.7 to -0.3)	<0.001
South Africa	1990 - 1993	0.8 (-3.5 to 5.2)	0.704
	1993 - 2004	3.3 (2.8 to 3.9)	<0.001
	2004 - 2008	-1.2 (-3.8 to 1.6)	0.381
	2008 - 2011	-4.7 (-9.5 to 0.4)	0.067
	2011 - 2021	-1.3 (-1.9 to -0.8)	<0.001
South Sudan	1990 - 1995	4.3 (2.2 to 6.4)	<0.001
	1995 - 2002	-4.6 (-6.1 to -3.1)	<0.001
	2002 - 2007	3.6 (0.5 to 6.7)	0.023
	2007 - 2010	-1.8 (-10.8 to 8)	0.69
	2010 - 2021	2.8 (2.2 to 3.5)	<0.001
Spain	1990 - 1997	0.6 (-0.8 to 2.1)	0.377
	1997 - 2010	-4.1 (-4.7 to -3.5)	<0.001
	2010 - 2013	4 (-6.7 to 16)	0.462

	2013 - 2021	-2.4 (-3.8 to -0.9)	0.003
Sri Lanka	1990 - 1993	-7.2 (-17.2 to 3.9)	0.182
	1993 - 1997	8.5 (-2.5 to 20.7)	0.126
	1997 - 2004	-9.5 (-11.8 to -7.1)	<0.001
	2004 - 2015	-5.6 (-6.7 to -4.5)	<0.001
	2015 - 2021	2.3 (-4.6 to 9.6)	0.503
Sudan	1990 - 1994	0.2 (-0.2 to 0.6)	0.358
	1994 - 2001	-0.8 (-1 to -0.6)	<0.001
	2001 - 2006	-2.3 (-2.7 to -1.8)	<0.001
	2006 - 2012	-0.6 (-1 to -0.3)	0.001
	2012 - 2016	-2.1 (-2.8 to -1.3)	<0.001
	2016 - 2019	-0.9 (-2.4 to 0.6)	0.224
	2019 - 2021	-2.5 (-4 to -1)	0.003
Suriname	1990 - 1993	5.5 (-1.3 to 12.8)	0.105
	1993 - 2001	-6.6 (-8 to -5.3)	<0.001
	2001 - 2007	6.7 (4.8 to 8.7)	<0.001
	2007 - 2011	-0.3 (-3.9 to 3.5)	0.875
	2011 - 2014	-7.4 (-14.2 to 0.1)	0.053
	2014 - 2017	5.5 (-2.6 to 14.2)	0.17
	2017 - 2021	-1.2 (-4 to 1.8)	0.402
Sweden	1990 - 1992	-7.5 (-14.8 to 0.3)	0.058
	1992 - 2001	-2.7 (-3.6 to -1.8)	<0.001
	2001 - 2011	2.6 (1.8 to 3.4)	<0.001
	2011 - 2016	-2.5 (-5.2 to 0.2)	0.065
	2016 - 2019	2.6 (-5.7 to 11.7)	0.526
	2019 - 2021	-11.3 (-22.6 to 1.7)	0.081
Switzerland	1990 - 1992	0.9 (-7.1 to 9.6)	0.822
	1992 - 2004	-5.3 (-5.8 to -4.8)	<0.001
	2004 - 2007	0.4 (-6.9 to 8.2)	0.92
	2007 - 2010	-7 (-13.8 to 0.3)	0.059
	2010 - 2021	-2 (-2.6 to -1.3)	<0.001
Syrian Arab Republic	1990 - 2000	-1.2 (-1.6 to -0.9)	<0.001
	2000 - 2003	-8 (-11.4 to -4.4)	<0.001
	2003 - 2008	-4.2 (-5.3 to -3.2)	<0.001
	2008 - 2011	0.5 (-3.6 to 4.9)	0.792
	2011 - 2016	-4 (-5.5 to -2.4)	<0.001
	2016 - 2021	2.6 (1.3 to 3.9)	0.001
Taiwan (Province of China)	1990 - 1998	-0.1 (-0.9 to 0.7)	0.788
	1998 - 2005	5.1 (3.8 to 6.4)	<0.001
	2005 - 2010	-6.4 (-8.3 to -4.4)	<0.001
	2010 - 2014	0.4 (-3.1 to 3.9)	0.829
	2014 - 2021	3.4 (2.1 to 4.6)	<0.001
Tajikistan	1990 - 1995	7.5 (6.3 to 8.7)	<0.001

	1995 - 2000	-7.2 (-8.9 to -5.4)	<0.001
	2000 - 2007	-3.9 (-5 to -2.8)	<0.001
	2007 - 2015	1.1 (-0.1 to 2.4)	0.064
	2015 - 2021	-3.2 (-4.9 to -1.4)	0.001
Thailand	1990 - 1996	7.2 (6.4 to 8)	<0.001
	1996 - 1999	-11.4 (-15.9 to -6.8)	<0.001
	1999 - 2008	-4.4 (-4.9 to -3.9)	<0.001
	2008 - 2013	-1.9 (-3.2 to -0.5)	0.01
	2013 - 2021	0.5 (0 to 1.1)	0.044
Timor-Leste	1990 - 2000	-3 (-4.5 to -1.4)	0.001
	2000 - 2008	-8.3 (-10.8 to -5.7)	<0.001
	2008 - 2012	17.4 (6 to 30)	0.004
	2012 - 2021	0.5 (-1.2 to 2.3)	0.546
Togo	1990 - 1998	2.6 (2.4 to 2.8)	<0.001
	1998 - 2003	0.8 (0.3 to 1.4)	0.004
	2003 - 2006	-0.7 (-2.4 to 0.9)	0.37
	2006 - 2009	2.7 (1.1 to 4.4)	0.003
	2009 - 2017	-0.5 (-0.8 to -0.3)	<0.001
	2017 - 2021	-1.7 (-2.4 to -1.1)	<0.001
Tokelau	1990 - 2018	-1.3 (-1.7 to -0.8)	<0.001
	2018 - 2021	31.7 (18 to 47)	<0.001
Tonga	1990 - 1992	-4.1 (-6.5 to -1.6)	0.003
	1992 - 1996	-0.7 (-1.9 to 0.6)	0.276
	1996 - 1999	7 (4.7 to 9.3)	<0.001
	1999 - 2021	0 (-0.1 to 0.1)	0.852
Trinidad and Tobago	1990 - 1994	-8.4 (-10.7 to -6)	<0.001
	1994 - 2006	-0.4 (-1.1 to 0.2)	0.186
	2006 - 2009	4.1 (-6.8 to 16.3)	0.453
	2009 - 2012	-7.4 (-17.3 to 3.7)	0.17
	2012 - 2021	0.8 (-1.1 to 2.7)	0.399
Tunisia	1990 - 1992	5.1 (3.7 to 6.5)	<0.001
	1992 - 1995	-1.5 (-2.7 to -0.3)	0.021
	1995 - 2003	-0.3 (-0.5 to -0.1)	0.002
	2003 - 2014	-1.6 (-1.7 to -1.5)	<0.001
	2014 - 2021	-2.3 (-2.5 to -2.1)	<0.001
Türkiye	1990 - 1999	-1.6 (-2 to -1.1)	<0.001
	1999 - 2002	-5.4 (-9.6 to -1)	0.022
	2002 - 2005	4.9 (1.3 to 8.6)	0.011
	2005 - 2009	1 (-0.4 to 2.3)	0.155
	2009 - 2012	-1.9 (-4.1 to 0.4)	0.094
	2012 - 2017	2.7 (2 to 3.4)	<0.001
	2017 - 2021	-0.8 (-1.8 to 0.3)	0.134
Turkmenistan	1990 - 1995	2.4 (0.8 to 4.1)	0.007
	1995 - 1998	13.8 (6.2 to 21.8)	0.001

	1998 - 2003	-1.8 (-4 to 0.4)	0.107
	2003 - 2006	5.2 (-2.7 to 13.8)	0.183
	2006 - 2010	-7.4 (-10.4 to -4.3)	<0.001
	2010 - 2021	-0.8 (-1.5 to 0)	0.042
Tuvalu	1990 - 1994	-2.8 (-3 to -2.5)	<0.001
	1994 - 1998	-1.2 (-1.6 to -0.8)	<0.001
	1998 - 2002	-2.4 (-2.8 to -2.1)	<0.001
	2002 - 2006	-1.1 (-1.5 to -0.7)	<0.001
	2006 - 2012	0 (-0.2 to 0.2)	0.815
	2012 - 2016	-0.5 (-0.9 to -0.1)	0.017
	2016 - 2021	-1.1 (-1.3 to -0.9)	<0.001
Uganda	1990 - 1995	7.5 (6.3 to 8.7)	<0.001
	1995 - 2001	1.8 (0.6 to 3)	0.004
	2001 - 2011	-2.9 (-3.4 to -2.5)	<0.001
	2011 - 2021	-0.1 (-0.5 to 0.3)	0.533
Ukraine	1990 - 1995	9.8 (8.2 to 11.4)	<0.001
	1995 - 2000	1.7 (-0.4 to 3.8)	0.104
	2000 - 2004	-3 (-6.1 to 0.2)	0.063
	2004 - 2013	1.2 (0.5 to 1.9)	0.002
	2013 - 2021	-4.1 (-5.6 to -2.6)	<0.001
United Arab Emirates	1990 - 2002	-0.1 (-1.1 to 1)	0.879
	2002 - 2005	12.5 (-4.1 to 31.9)	0.137
	2005 - 2008	-18.6 (-31.6 to -3.2)	0.023
	2008 - 2016	4.5 (1.9 to 7.2)	0.002
	2016 - 2021	-8.2 (-11.8 to -4.5)	<0.001
United Kingdom	1990 - 1999	-1.2 (-2 to -0.4)	0.008
	1999 - 2004	-6 (-9 to -2.9)	0.001
	2004 - 2016	-0.7 (-1.3 to 0)	0.039
	2016 - 2021	2.4 (-0.7 to 5.6)	0.124
United Republic of Tanzania	1990 - 1998	0.3 (0.1 to 0.4)	0.001
	1998 - 2001	-2.1 (-3.3 to -0.8)	0.003
	2001 - 2004	-5.1 (-6.3 to -3.9)	<0.001
	2004 - 2009	-0.2 (-0.5 to 0.2)	0.406
	2009 - 2014	-1.5 (-1.9 to -1.1)	<0.001
	2014 - 2021	-0.2 (-0.4 to 0)	0.109
United States of America	1990 - 1994	0.8 (-0.1 to 1.6)	0.069
	1994 - 1999	-4.7 (-5.6 to -3.9)	<0.001
	1999 - 2007	-0.5 (-0.8 to -0.1)	0.016
	2007 - 2014	2.7 (2.2 to 3.1)	<0.001
	2014 - 2017	6 (3.4 to 8.6)	<0.001
	2017 - 2021	-1.2 (-2.4 to 0.1)	0.063
United States Virgin Islands	1990 - 1996	3.4 (1.2 to 5.7)	0.005

	1996 - 2005	-1.2 (-2.6 to 0.2)	0.091
	2005 - 2018	5.4 (4.3 to 6.6)	<0.001
	2018 - 2021	-14.7 (-23.5 to -5)	0.006
Uruguay	1990 - 2000	3 (2.1 to 3.9)	<0.001
	2000 - 2005	-2 (-4.9 to 1)	0.187
	2005 - 2021	2.8 (2.4 to 3.3)	<0.001
Uzbekistan	1990 - 1995	-0.2 (-1.1 to 0.7)	0.652
	1995 - 2000	4.9 (3.5 to 6.4)	<0.001
	2000 - 2004	-2.9 (-5.2 to -0.6)	0.018
	2004 - 2007	6.3 (0.9 to 11.9)	0.025
	2007 - 2010	-1.8 (-6 to 2.7)	0.402
	2010 - 2015	8.1 (6.8 to 9.4)	<0.001
	2015 - 2021	-2.7 (-3.4 to -2)	<0.001
Vanuatu	1990 - 1992	5.7 (2.7 to 8.8)	0.001
	1992 - 2000	0 (-0.4 to 0.3)	0.852
	2000 - 2003	-4.1 (-6.2 to -1.9)	0.001
	2003 - 2007	1.7 (0.5 to 2.9)	0.008
	2007 - 2021	-0.3 (-0.4 to -0.2)	<0.001
Venezuela (Bolivarian Republic of)	1990 - 1998	6.3 (5.3 to 7.3)	<0.001
	1998 - 2001	-3.6 (-12.2 to 5.7)	0.415
	2001 - 2021	1.3 (0.9 to 1.7)	<0.001
Viet Nam	1990 - 1997	-2.6 (-2.7 to -2.5)	<0.001
	1997 - 2001	-2 (-2.3 to -1.7)	<0.001
	2001 - 2005	1.2 (0.8 to 1.5)	<0.001
	2005 - 2008	0.4 (-0.2 to 1.1)	0.182
	2008 - 2013	1.3 (1.1 to 1.5)	<0.001
	2013 - 2016	-1 (-1.7 to -0.3)	0.009
	2016 - 2021	-2.1 (-2.3 to -2)	<0.001
Yemen	1990 - 2008	2.4 (2 to 2.8)	<0.001
	2008 - 2011	-10.3 (-19.4 to -0.2)	0.046
	2011 - 2021	-1.8 (-2.8 to -0.9)	0.001
Zambia	1990 - 1995	4.7 (4.3 to 5.1)	<0.001
	1995 - 1998	1.1 (-0.8 to 3)	0.233
	1998 - 2008	-1.5 (-1.7 to -1.3)	<0.001
	2008 - 2011	-0.3 (-2 to 1.4)	0.699
	2011 - 2014	-2.7 (-4.6 to -0.9)	0.008
	2014 - 2017	-0.7 (-2.8 to 1.5)	0.513
	2017 - 2021	-1.8 (-2.7 to -1)	<0.001
Zimbabwe	1990 - 1997	0.8 (-0.7 to 2.3)	0.28
	1997 - 2002	12.5 (9.3 to 15.9)	<0.001
	2002 - 2021	0.8 (0.5 to 1)	<0.001

Table S6. Wald χ^2 test of suicide mortality in age, period, and cohort models in global and 5 SDI regions

SDI	Sex	χ^2test	χ^2	df	P value
Global	Both	All Age Deviations = 0	2995.011005	1	<0.001
		All Cohort Deviations = 0	3.148493979	6	0.790
		All Cohort RR = 1	1416.518558	7	<0.001
		All Local Drifts = Net Drift	2.050099544	3	0.562
		All Period Deviations = 0	10.34440803	4	0.035
		All Period RR = 1	871.1414084	5	<0.001
		NetDrift = 0	787.9582978	1	<0.001
	Male	All Age Deviations = 0	4366.920373	1	<0.001
		All Cohort Deviations = 0	53.15597945	6	<0.001
		All Cohort RR = 1	2376.073527	7	<0.001
		All Local Drifts = Net Drift	49.34366504	3	<0.001
		All Period Deviations = 0	28.71667292	4	<0.001
		All Period RR = 1	1565.680221	5	<0.001
		NetDrift = 0	1473.465765	1	<0.001
	Female	All Age Deviations = 0	1459.236562	1	<0.001
		All Cohort Deviations = 0	6.682091438	6	0.351
		All Cohort RR = 1	645.7503303	7	<0.001
		All Local Drifts = Net Drift	5.196416625	3	0.158
		All Period Deviations = 0	14.7081822	4	0.005
		All Period RR = 1	374.3464104	5	<0.001
		NetDrift = 0	316.2720957	1	<0.001
Low SDI	Both	All Age Deviations = 0	2187.915784	1	<0.001
		All Cohort Deviations = 0	16.67405947	6	0.011
		All Cohort RR = 1	668.8712556	7	<0.001
		All Local Drifts = Net Drift	10.28185273	3	0.016
		All Period Deviations = 0	20.09939153	4	<0.001
		All Period RR = 1	647.1285841	5	<0.001
		NetDrift = 0	536.1618165	1	<0.001
	Male	All Age Deviations = 0	333.3321428	1	<0.001
		All Cohort Deviations = 0	15.68834457	6	0.016
		All Cohort RR = 1	139.1191826	7	<0.001
		All Local Drifts = Net Drift	12.56095746	3	0.006
		All Period Deviations = 0	14.32923245	4	0.006
		All Period RR = 1	150.0053259	5	<0.001
		NetDrift = 0	128.6618158	1	<0.001
	Female	All Age Deviations = 0	2034.802958	1	<0.001
		All Cohort Deviations = 0	8.673000025	6	0.193
		All Cohort RR = 1	568.6481187	7	<0.001
		All Local Drifts = Net Drift	7.441187671	3	0.059
		All Period Deviations = 0	27.28886669	4	<0.001
		All Period RR = 1	484.2065374	5	<0.001

		NetDrift = 0	359.964061	1	<0.001
Low-middle SDI	Both	All Age Deviations = 0	2247.989003	1	<0.001
		All Cohort Deviations = 0	1.255648609	6	0.974
		All Cohort RR = 1	1036.46032	7	<0.001
		All Local Drifts = Net Drift	0.275104687	3	0.965
		All Period Deviations = 0	43.74810234	4	<0.001
		All Period RR = 1	684.2599719	5	<0.001
		NetDrift = 0	523.9547257	1	<0.001
	Male	All Age Deviations = 0	1438.854342	1	<0.001
		All Cohort Deviations = 0	11.16731482	6	0.083
		All Cohort RR = 1	630.6965204	7	<0.001
		All Local Drifts = Net Drift	10.12656326	3	0.018
		All Period Deviations = 0	24.72828824	4	<0.001
		All Period RR = 1	521.688706	5	<0.001
		NetDrift = 0	406.4914314	1	<0.001
	Female	All Age Deviations = 0	2543.645764	1	<0.001
		All Cohort Deviations = 0	16.25327826	6	0.012
		All Cohort RR = 1	1259.562899	7	<0.001
		All Local Drifts = Net Drift	14.00457884	3	0.003
		All Period Deviations = 0	76.19982275	4	<0.001
		All Period RR = 1	731.7155456	5	<0.001
		NetDrift = 0	534.4525099	1	<0.001
Middle SDI	Both	All Age Deviations = 0	1254.232671	1	<0.001
		All Cohort Deviations = 0	7.203638869	6	0.302
		All Cohort RR = 1	746.5190757	7	<0.001
		All Local Drifts = Net Drift	6.392635439	3	0.094
		All Period Deviations = 0	18.00912042	4	0.001
		All Period RR = 1	449.4114983	5	<0.001
		NetDrift = 0	441.4675452	1	<0.001
	Male	All Age Deviations = 0	2877.788678	1	<0.001
		All Cohort Deviations = 0	113.0821768	6	<0.001
		All Cohort RR = 1	1558.811914	7	<0.001
		All Local Drifts = Net Drift	111.5396058	3	<0.001
		All Period Deviations = 0	160.2741971	4	<0.001
		All Period RR = 1	1108.28327	5	<0.001
		NetDrift = 0	1071.728624	1	<0.001
	Female	All Age Deviations = 0	532.7201086	1	<0.001
		All Cohort Deviations = 0	5.185404727	6	0.520
		All Cohort RR = 1	355.5788809	7	<0.001
		All Local Drifts = Net Drift	4.521186611	3	0.210
		All Period Deviations = 0	1.213085582	4	0.876
		All Period RR = 1	192.722118	5	<0.001
		NetDrift = 0	181.6966658	1	<0.001
High-middle SDI	Both	All Age Deviations = 0	823.0813886	1	<0.001

		All Cohort Deviations = 0	15.14802254	6	0.019
		All Cohort RR = 1	1173.958155	7	<0.001
		All Local Drifts = Net Drift	11.14454952	3	0.011
		All Period Deviations = 0	12.97897984	4	0.011
		All Period RR = 1	612.4385871	5	<0.001
		NetDrift = 0	572.179916	1	<0.001
	Male	All Age Deviations = 0	384.9593461	1	<0.001
		All Cohort Deviations = 0	18.30833462	6	0.006
		All Cohort RR = 1	601.1861773	7	<0.001
		All Local Drifts = Net Drift	13.0898038	3	0.004
		All Period Deviations = 0	10.34579145	4	0.035
		All Period RR = 1	309.0251109	5	<0.001
		NetDrift = 0	280.4245493	1	<0.001
	Female	All Age Deviations = 0	2257.984898	1	<0.001
		All Cohort Deviations = 0	17.63935306	6	0.007
		All Cohort RR = 1	2794.414996	7	<0.001
		All Local Drifts = Net Drift	12.39721107	3	0.006
		All Period Deviations = 0	16.65391197	4	0.002
		All Period RR = 1	1520.089788	5	<0.001
		NetDrift = 0	1463.084095	1	<0.001
High SDI	Both	All Age Deviations = 0	3248.960804	1	<0.001
		All Cohort Deviations = 0	28.11572194	6	<0.001
		All Cohort RR = 1	120.1583768	7	<0.001
		All Local Drifts = Net Drift	26.36980175	3	<0.001
		All Period Deviations = 0	66.49141206	4	<0.001
		All Period RR = 1	68.89905736	5	<0.001
		NetDrift = 0	15.29937942	1	<0.001
	Male	All Age Deviations = 0	3013.44654	1	<0.001
		All Cohort Deviations = 0	19.1162837	6	0.004
		All Cohort RR = 1	178.7896051	7	<0.001
		All Local Drifts = Net Drift	17.43655862	3	0.001
		All Period Deviations = 0	84.65538084	4	<0.001
		All Period RR = 1	103.0770469	5	<0.001
		NetDrift = 0	47.32271587	1	<0.001
	Female	All Age Deviations = 0	984.2296309	1	<0.001
		All Cohort Deviations = 0	25.7876288	6	<0.001
		All Cohort RR = 1	25.78883629	7	0.001
		All Local Drifts = Net Drift	23.83506614	3	<0.001
		All Period Deviations = 0	5.952670944	4	0.203
		All Period RR = 1	18.17274853	5	0.003
		NetDrift = 0	7.890004068	1	0.005

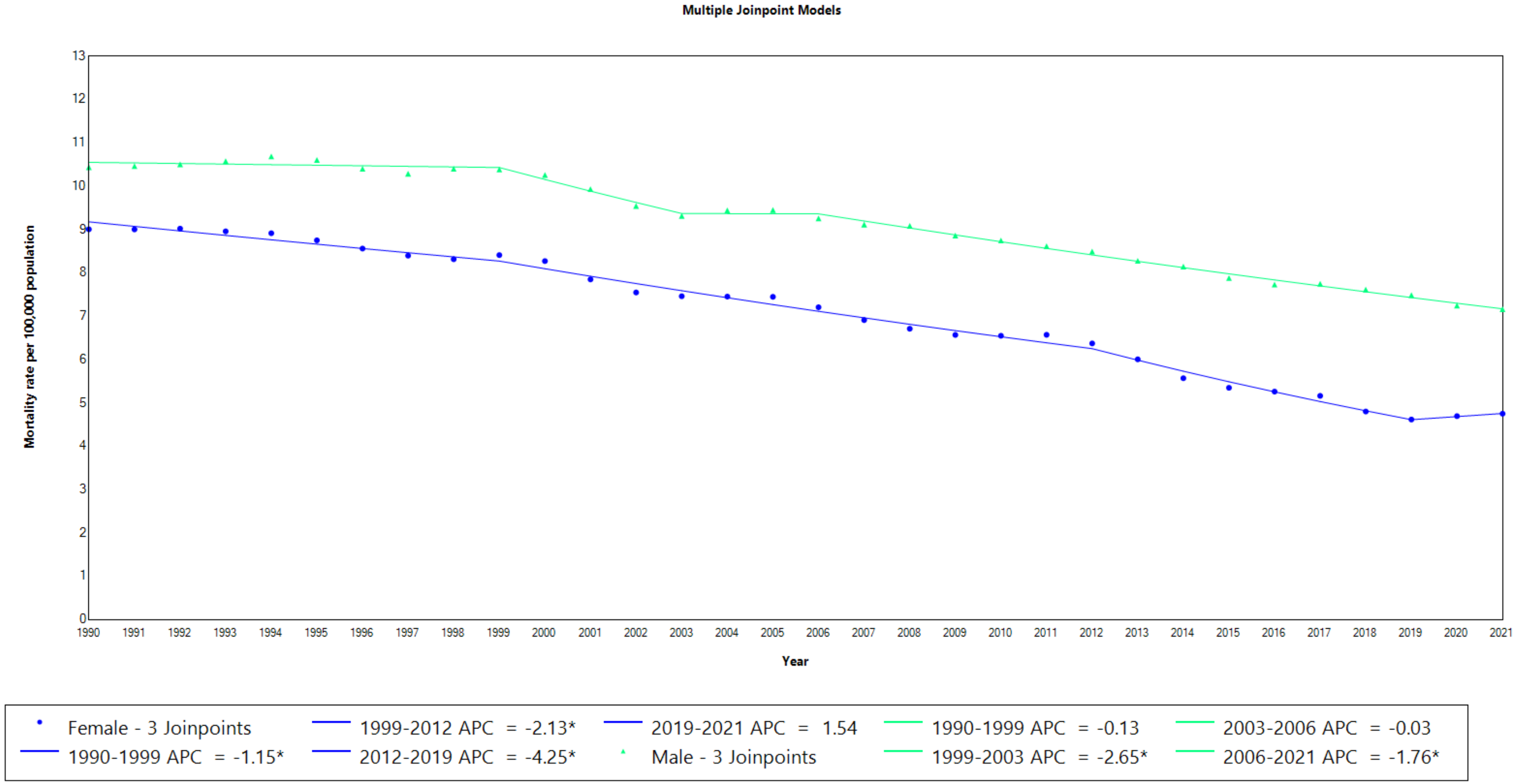

Figure S1. Joinpoint regression analysis of mortality from suicide in different sexes from 1990 to 2021

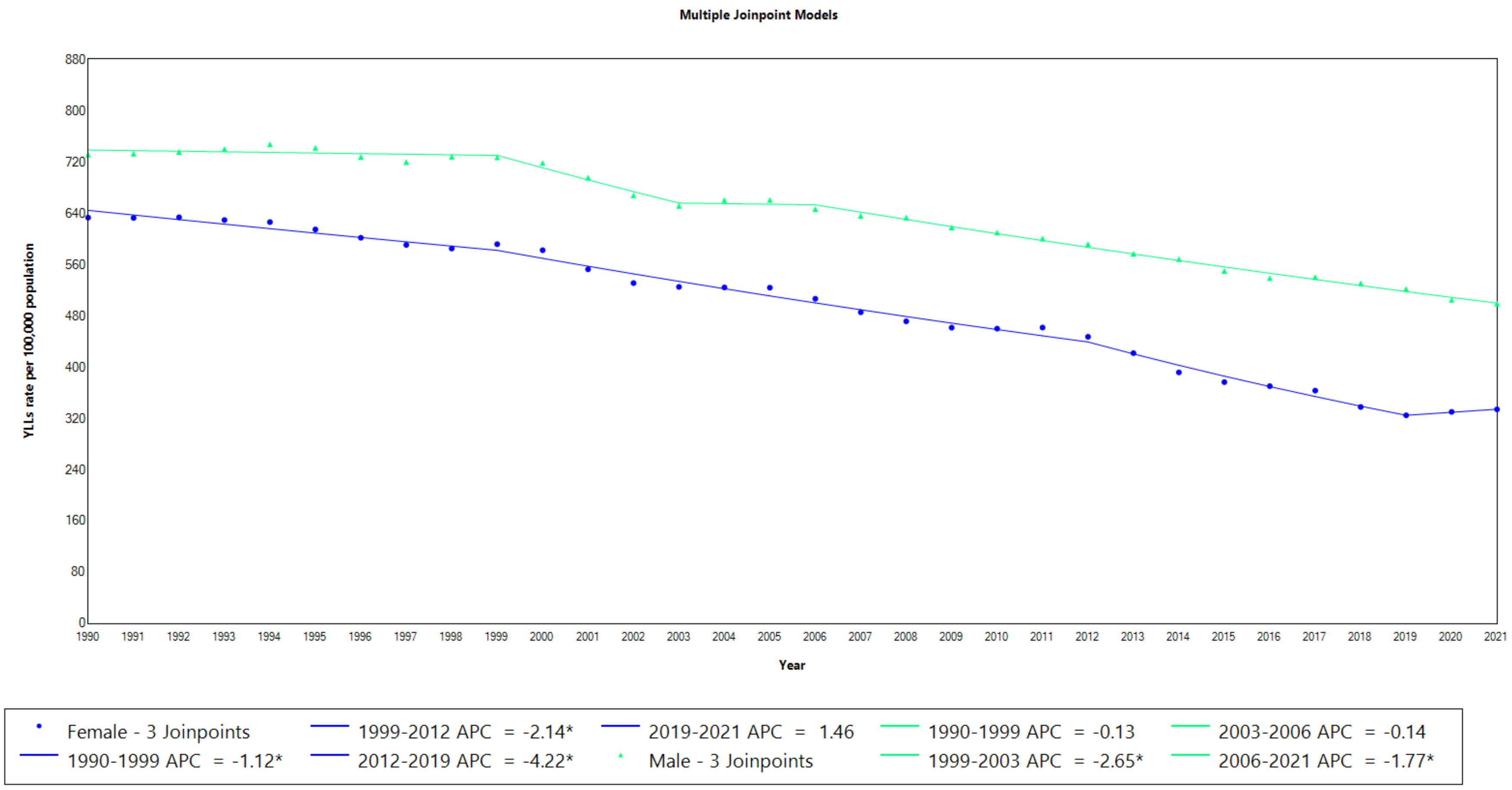

Figure S2. Joinpoint regression analysis of rate of YLLs from suicide in different sexes from 1990 to 2021

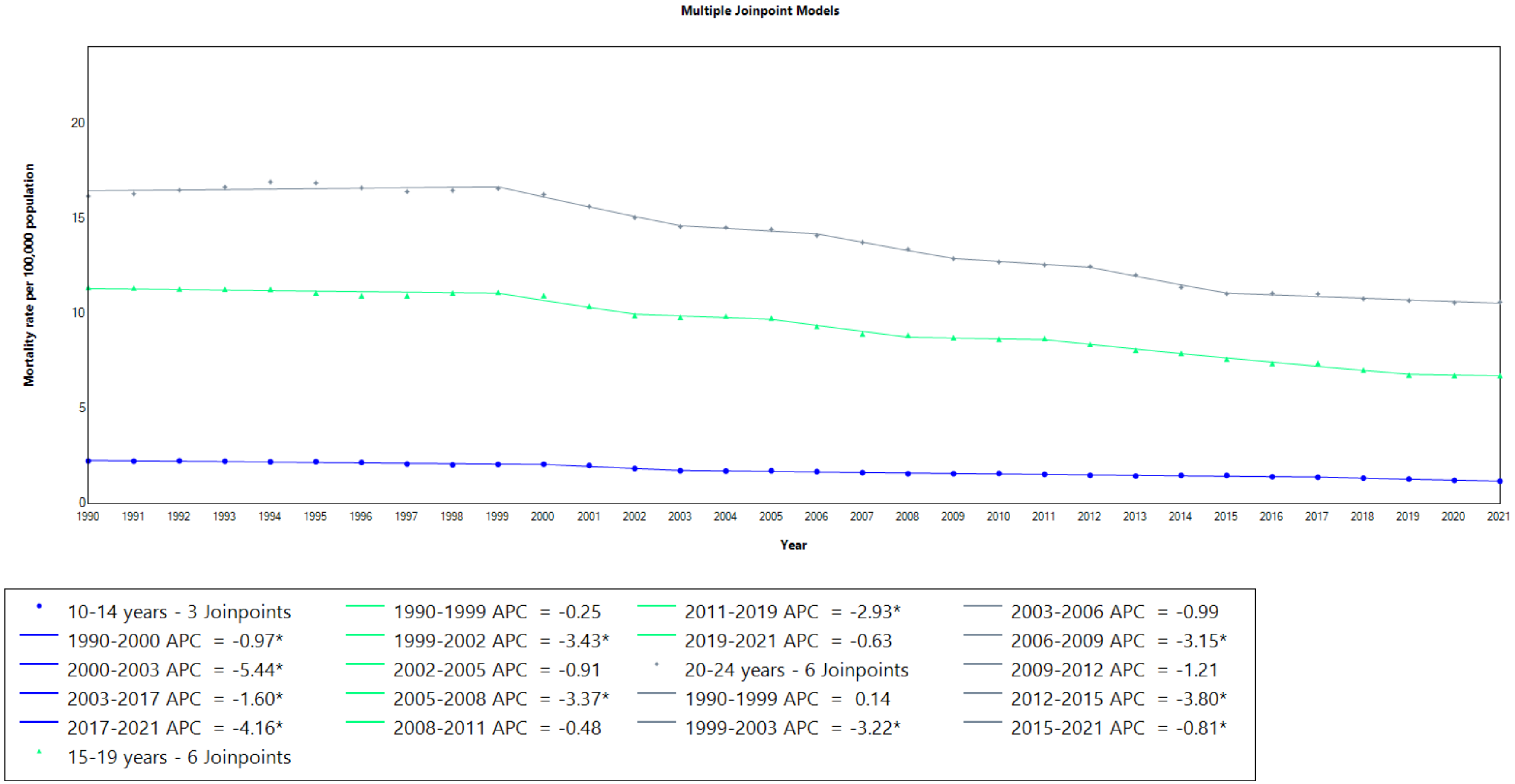

Figure S3. Joinpoint regression analysis of mortality from suicide in different age groups from 1990 to 2021

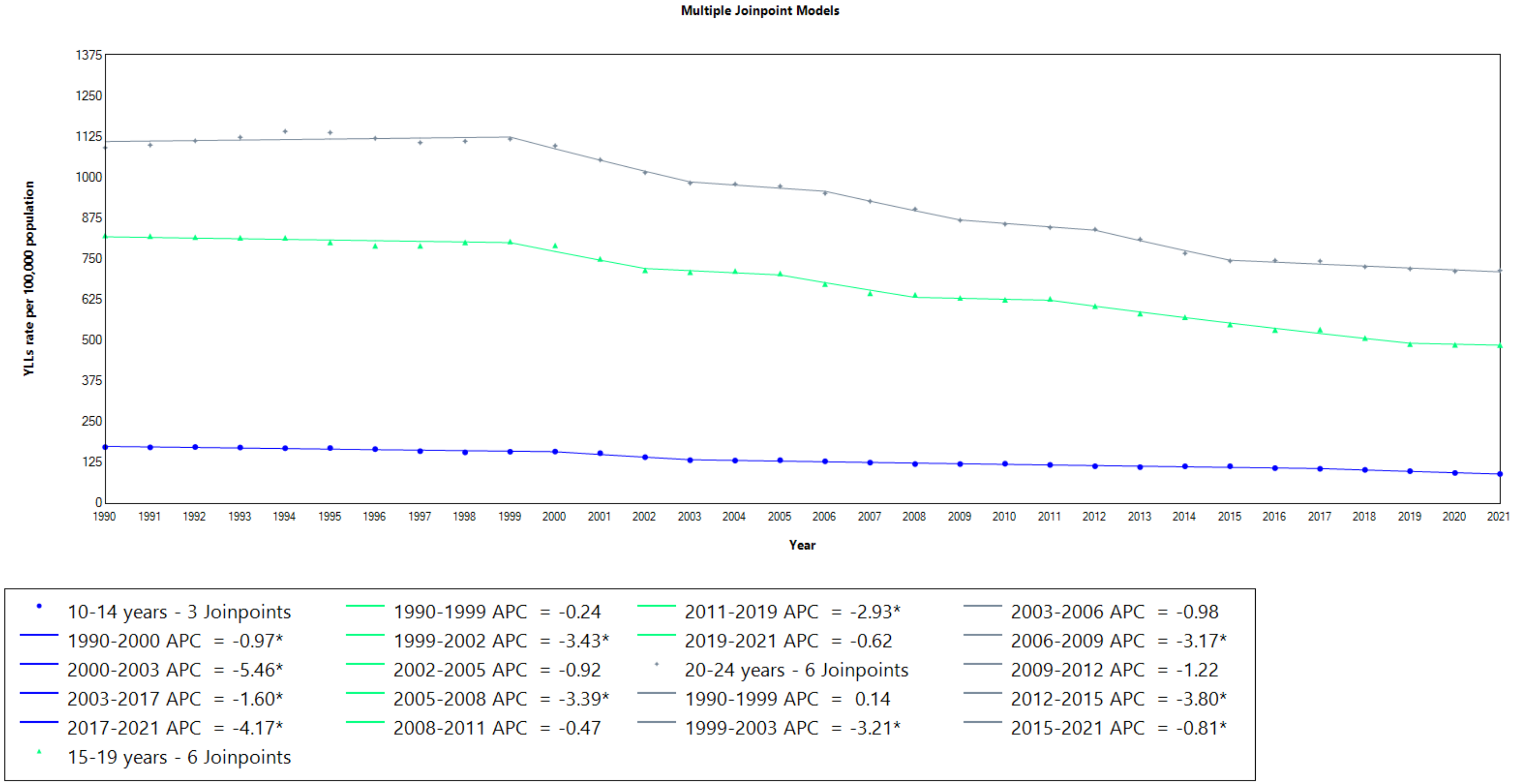

Figure S4. Joinpoint regression analysis of rate of YLLs from suicide in different age groups from 1990 to 2021

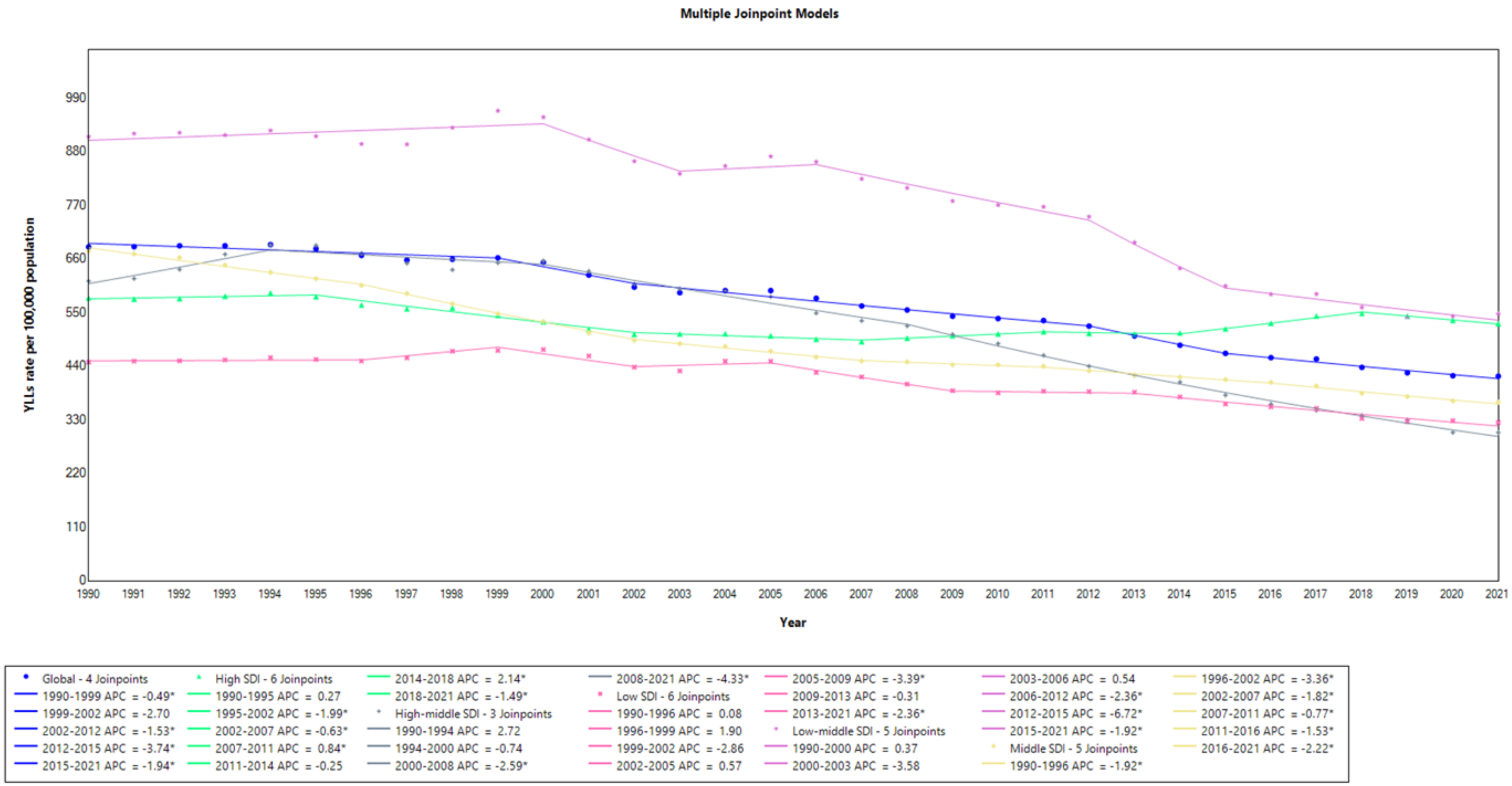

Figure S5. Joinpoint regression analysis of rate of suicide YLLs aged 10–24 years in global and 5 SDI regions from 1990 to 2021

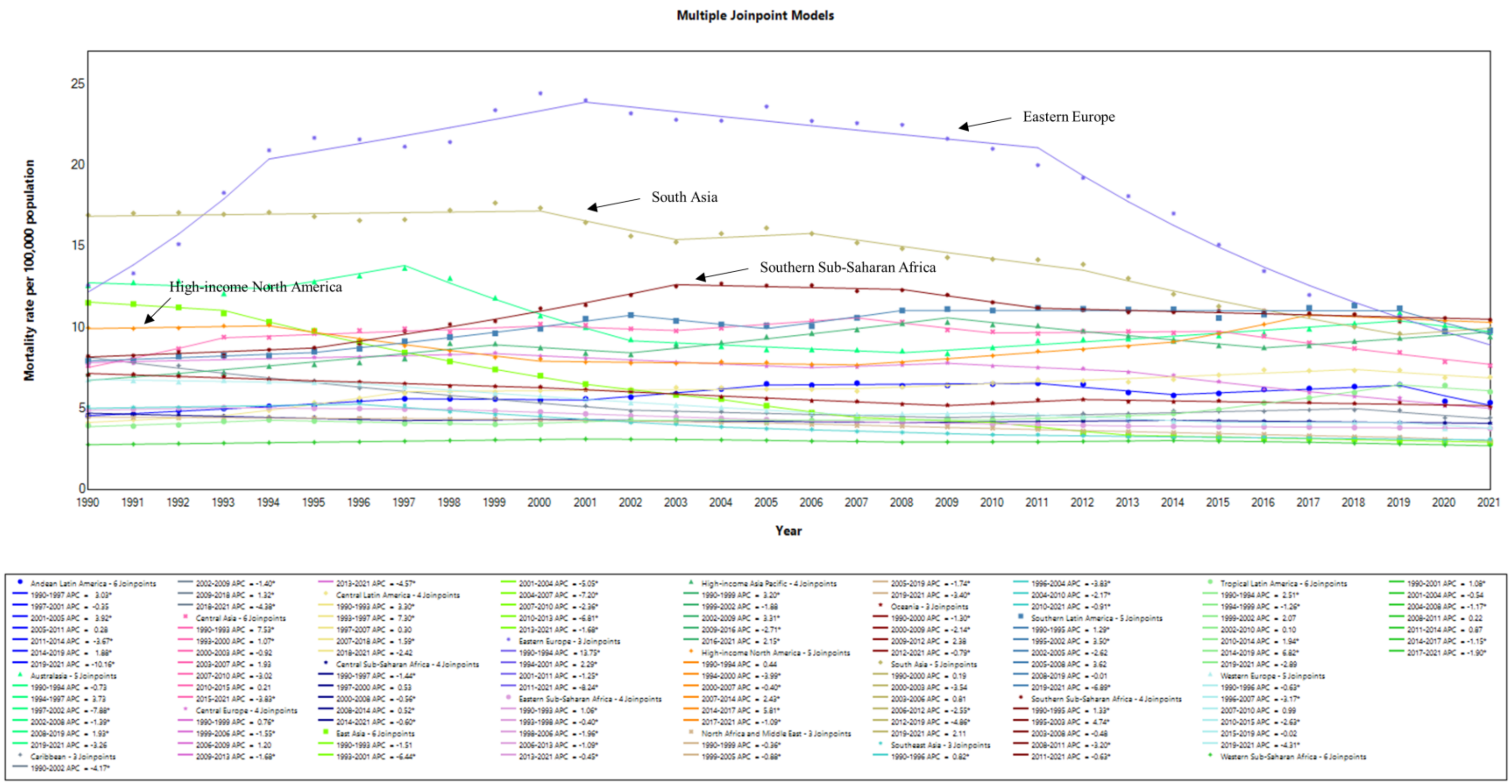

Figure S6. Joinpoint regression analysis of mortality from suicide in 21 regions from 1990 to 2021

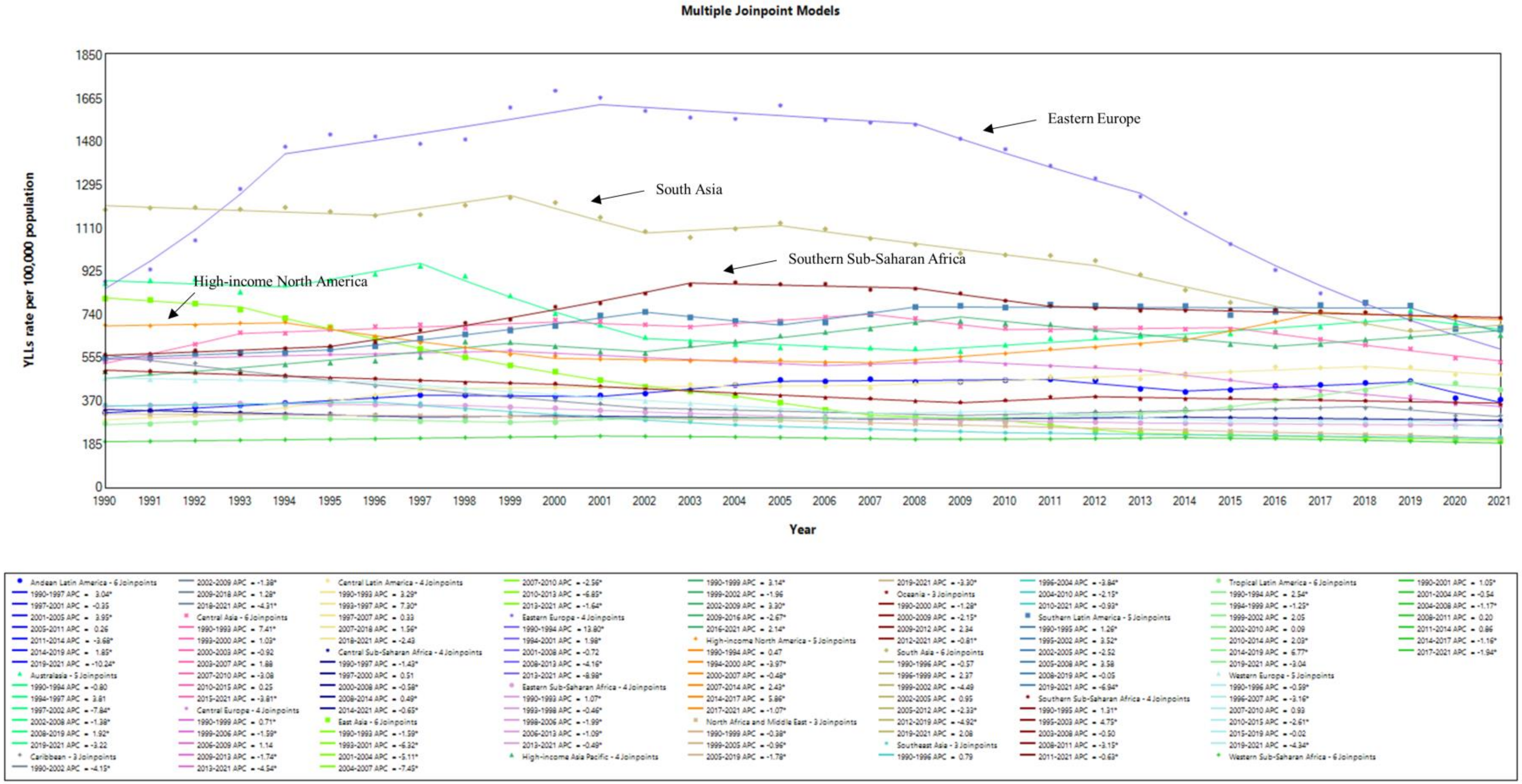

Figure S7. Joinpoint regression analysis of rate of YLLs from suicide in 21 regions from 1990 to 2021

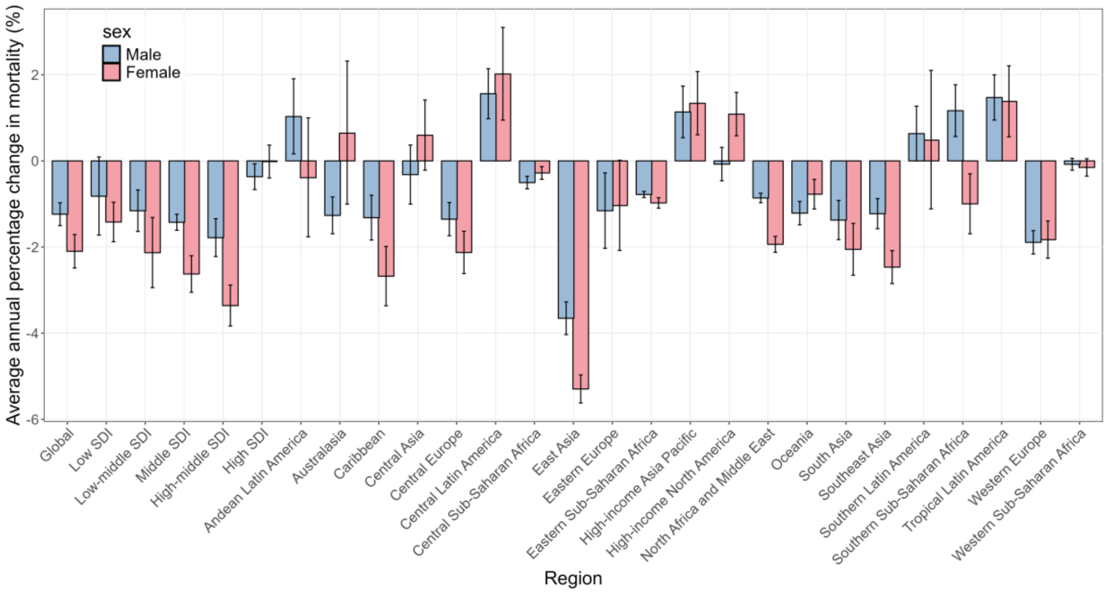

Figure S8. Joinpoint regression analysis of mortality from suicide among males and females in global and different regions, 1990-2021

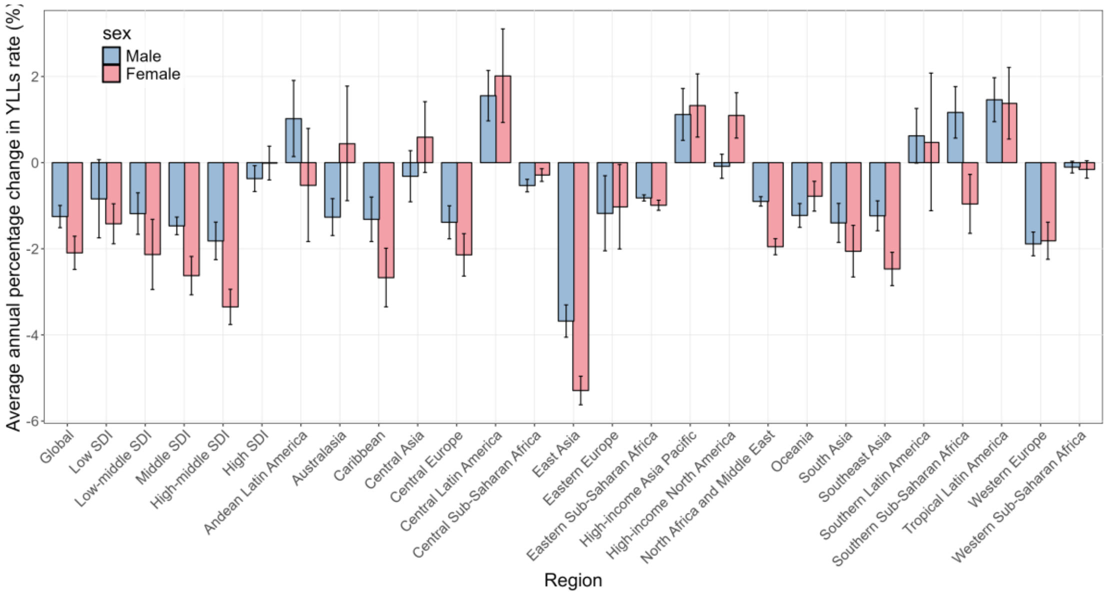

Figure S9. Joinpoint regression analysis of rate of YLLs from suicide among males and females in global and different regions, 1990-2021

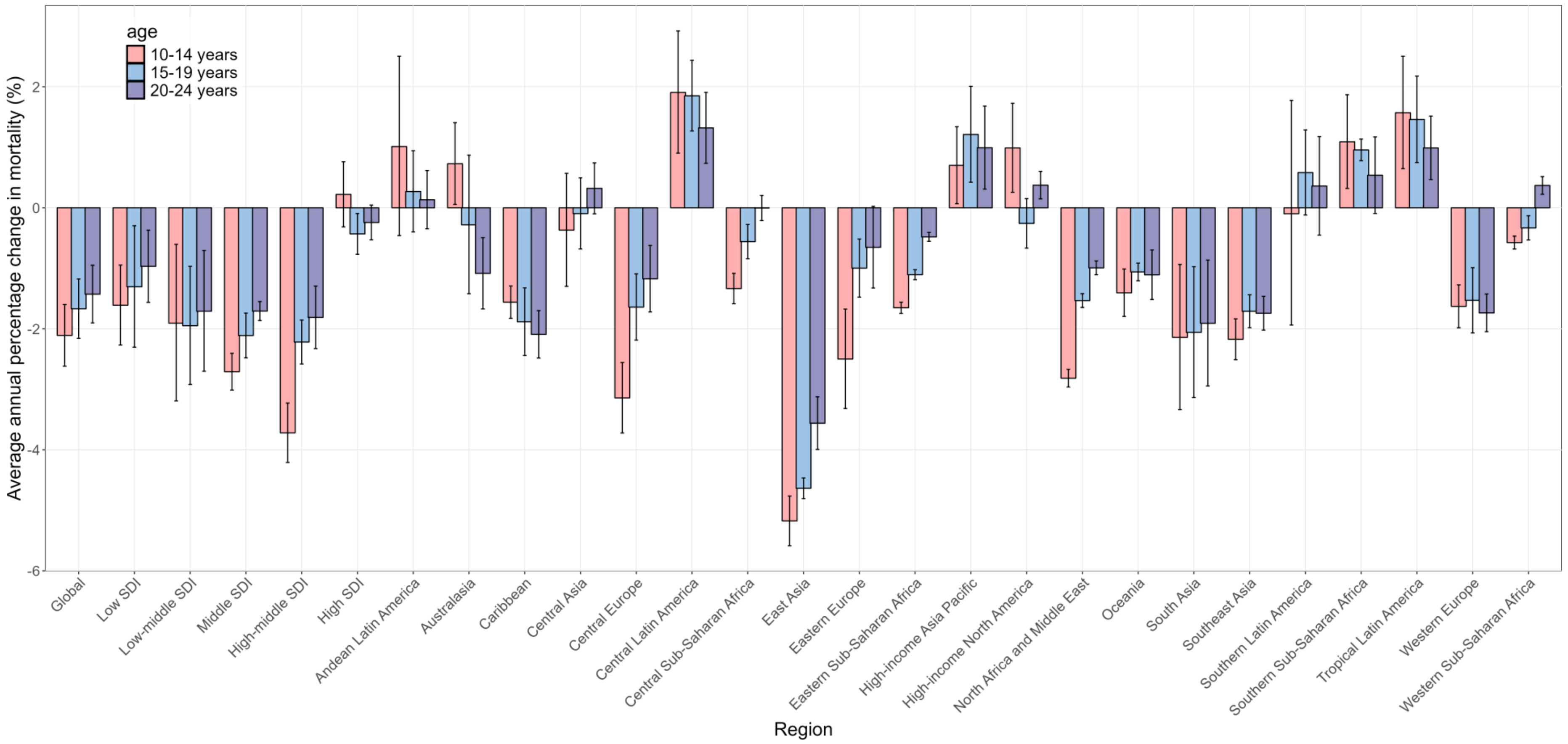

Figure S10. Joinpoint regression analysis of mortality from suicide among different age groups in global and different regions, 1990-2021

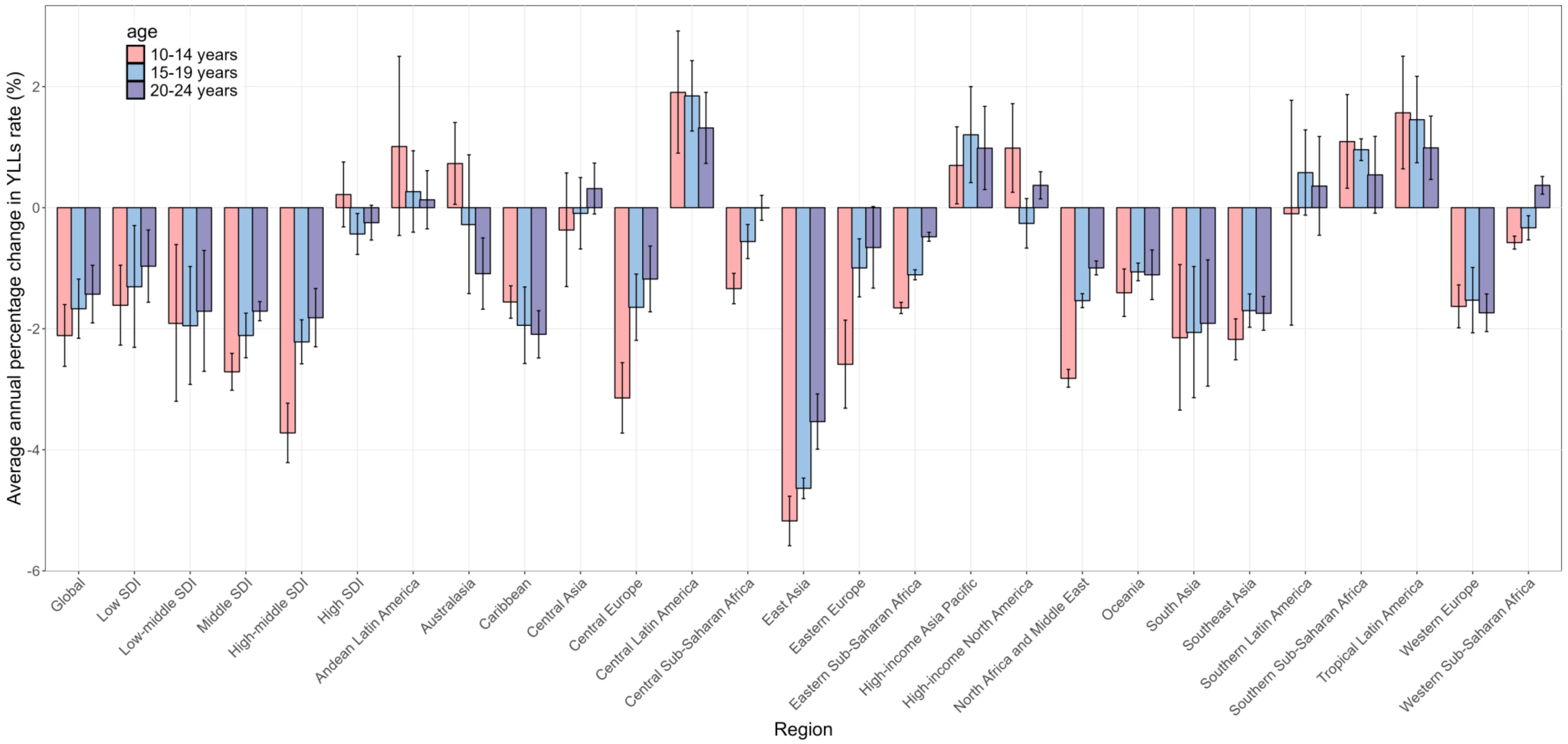

Figure S11. Joinpoint regression analysis of rate of YLLs from suicide among different age groups in global and different regions, 1990-2021

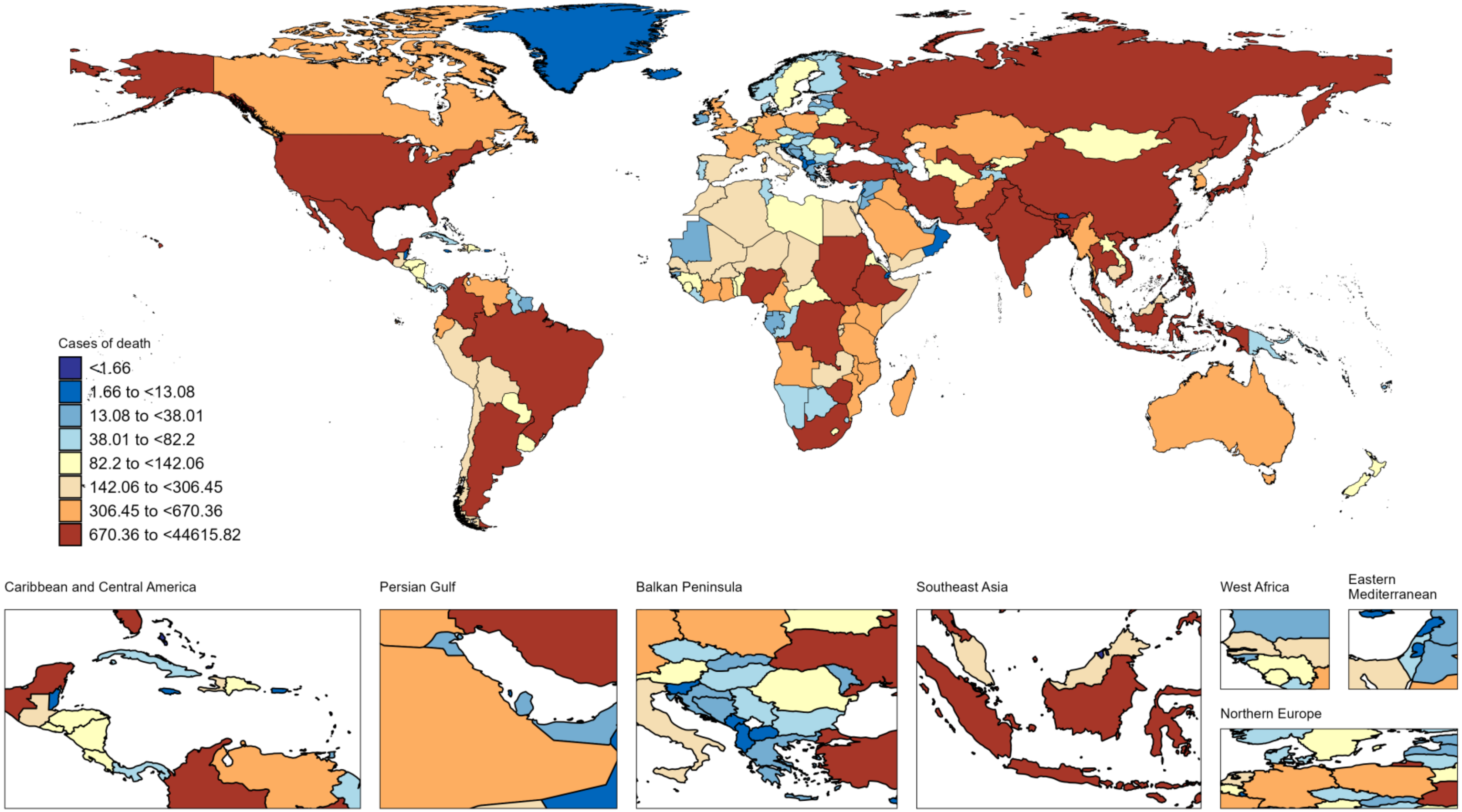

Figure S12. Global map of death cases from suicide in 2021

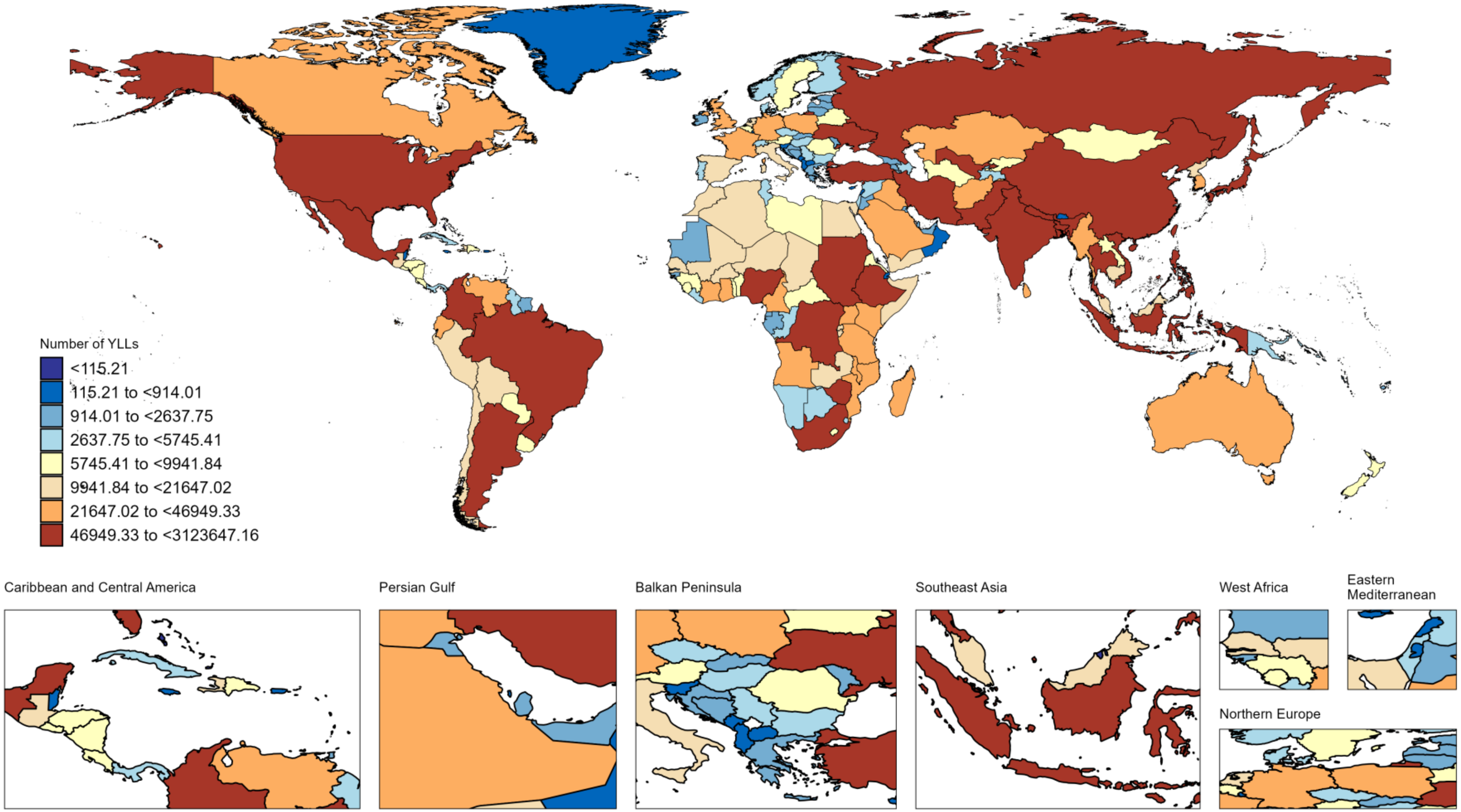

Figure S13. Global map of YLLs from suicide in 2021

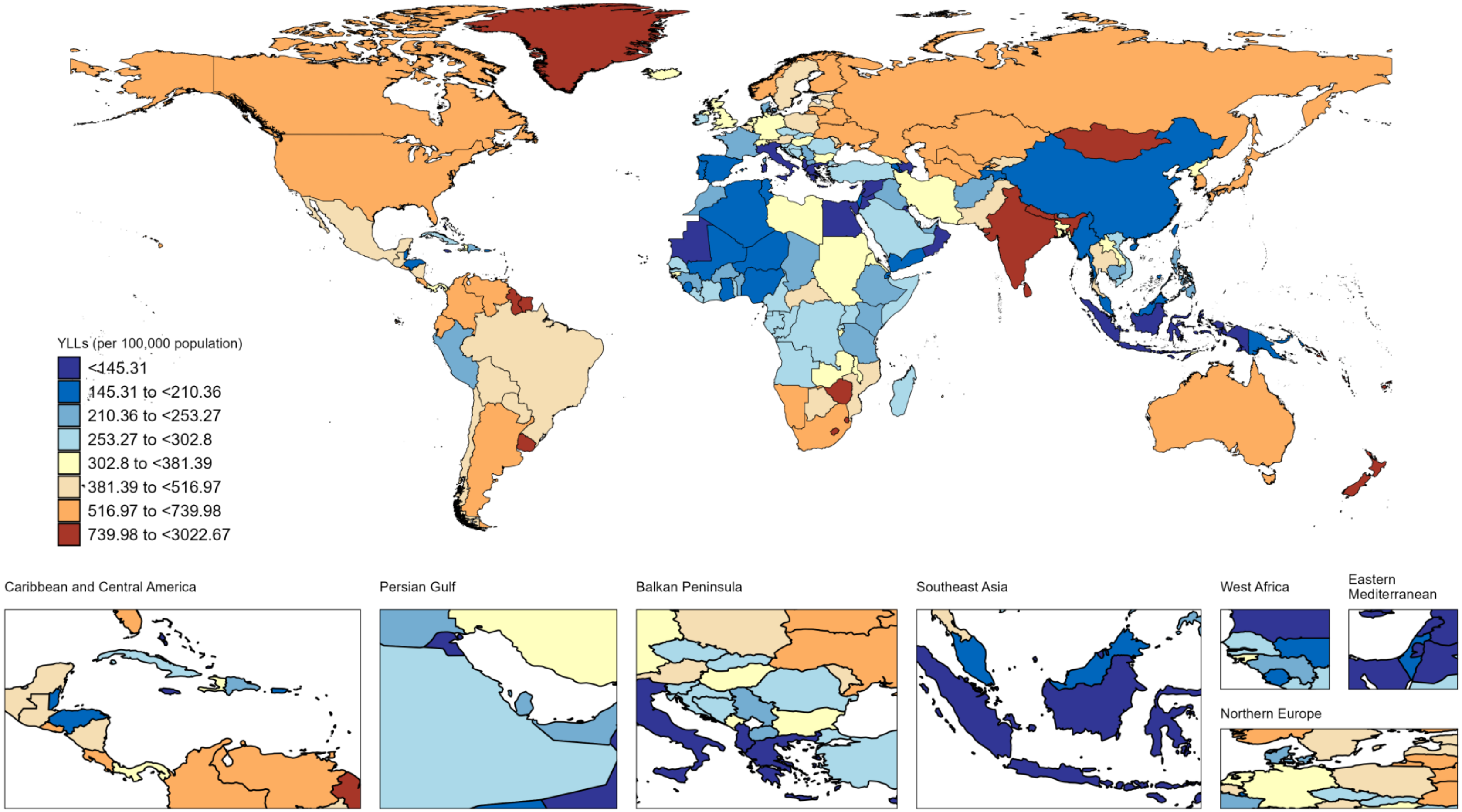

Figure S14. Global map of rate of YLLs from suicide in 2021

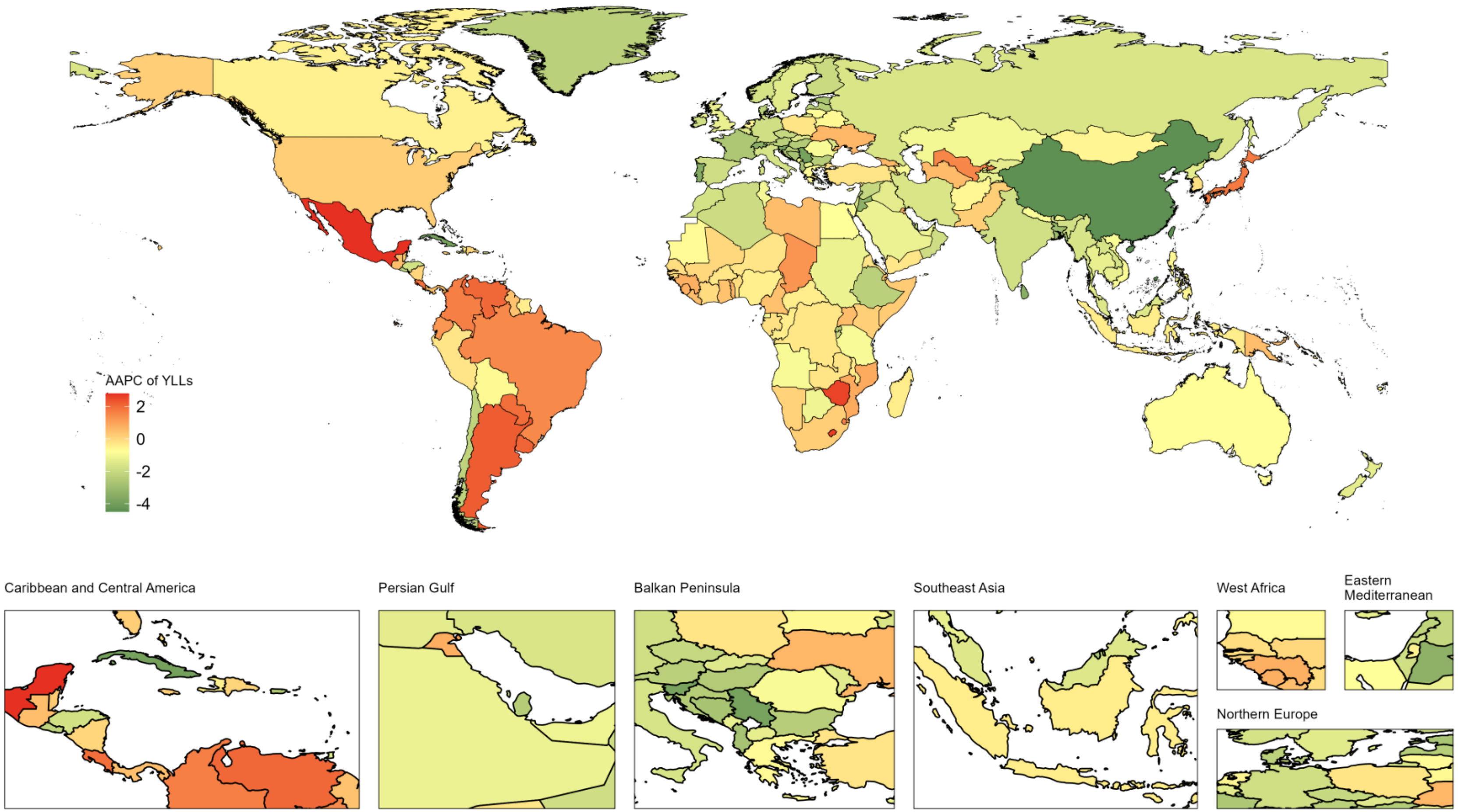

Figure S15. Global map of AAPC in rate of YLLs from suicide from 1990 to 2021

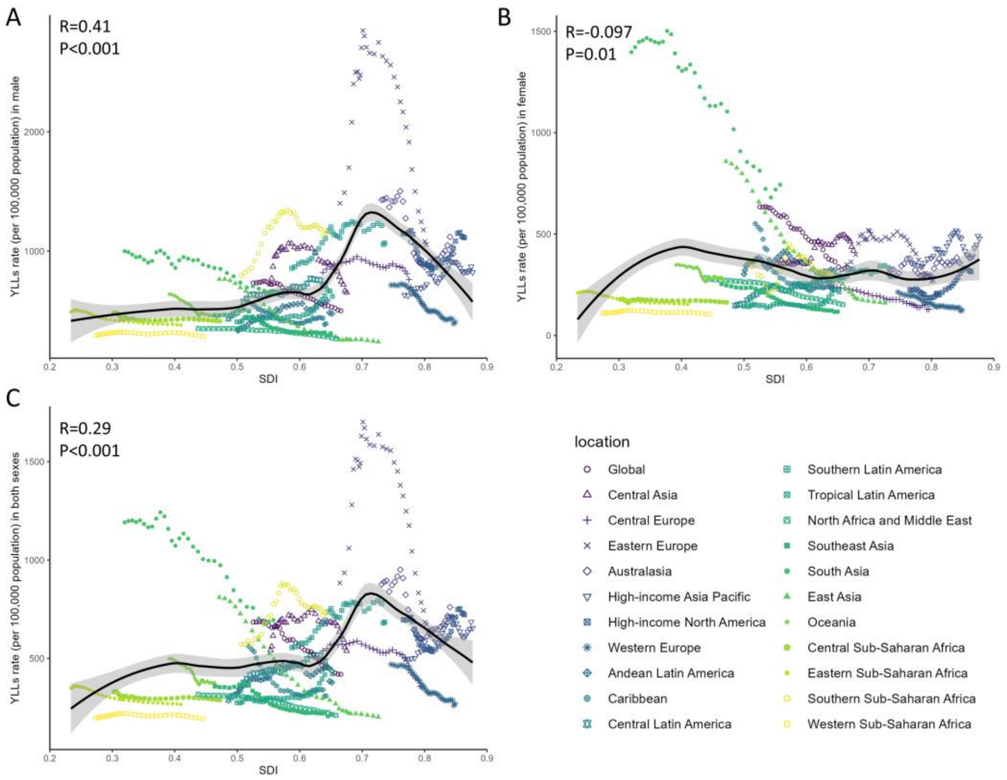

Figure S16. Trends for rate of YLLs of suicide among 21 regions by SDI for males (A), females (B), and both sexes (C) in 10-24 years population combined from 1990 to 2021

SDI: Socio-demographic Index; Black line represents the expected rate of YLLs based on SDIs in all locations.

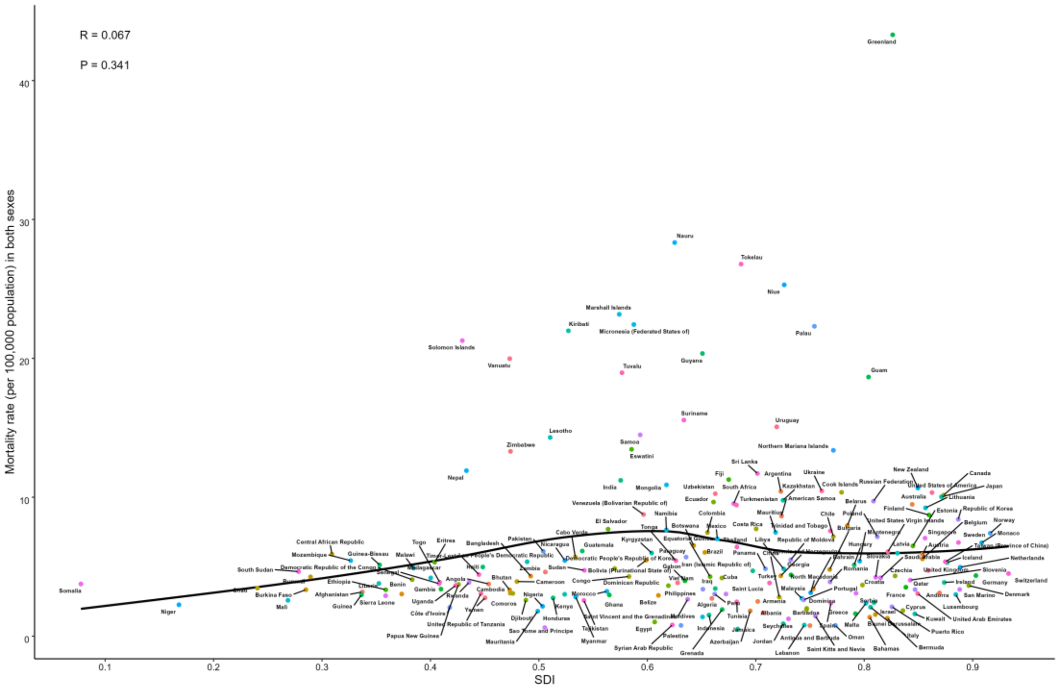

Figure S17. Trends for mortality of suicide among 204 countries by SDI for both sexes in 10-24 years population in 2021

SDI: Socio-demographic Index; Black line represents the expected mortality based on SDIs in all countries.

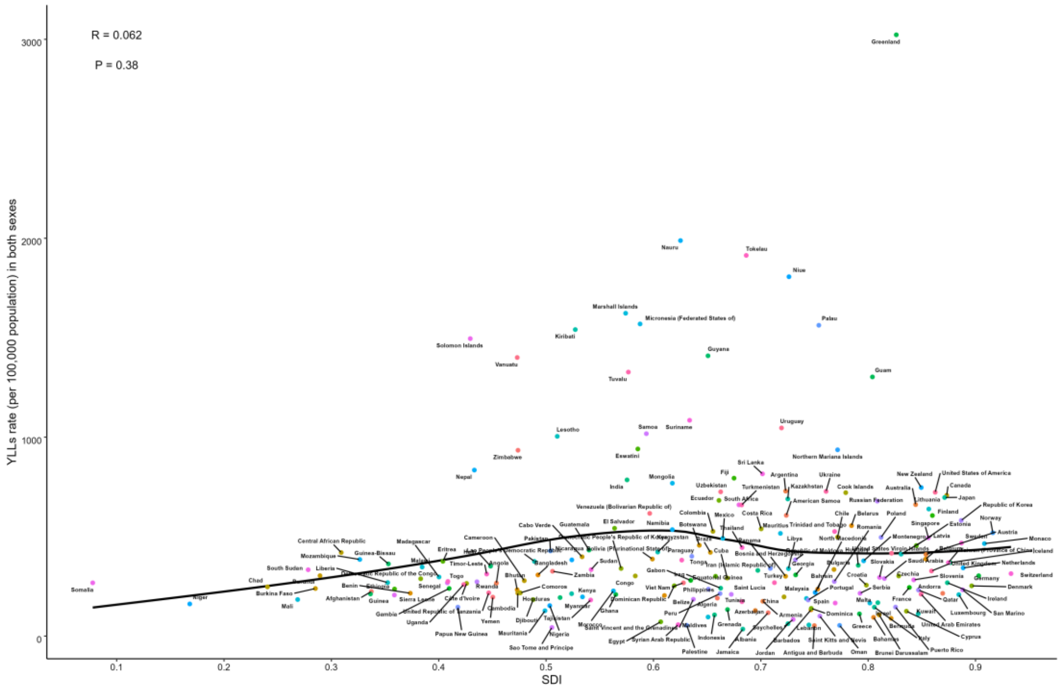

Figure S18. Trends for rate of YLLs of suicide among 204 countries by SDI for both sexes in 10-24 years population in 2021

SDI: Socio-demographic Index; Black line represents the expected rate of YLLs based on SDIs in all locations.

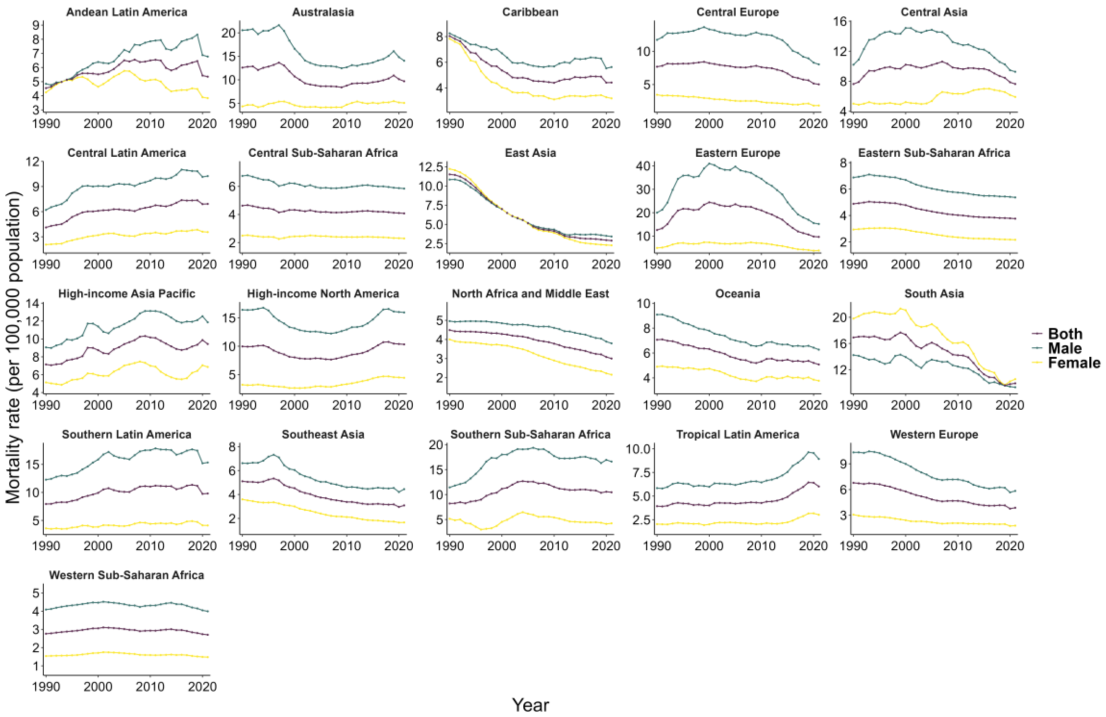

Figure S19. The trends in suicide mortality in 21 regions, 1990-2021

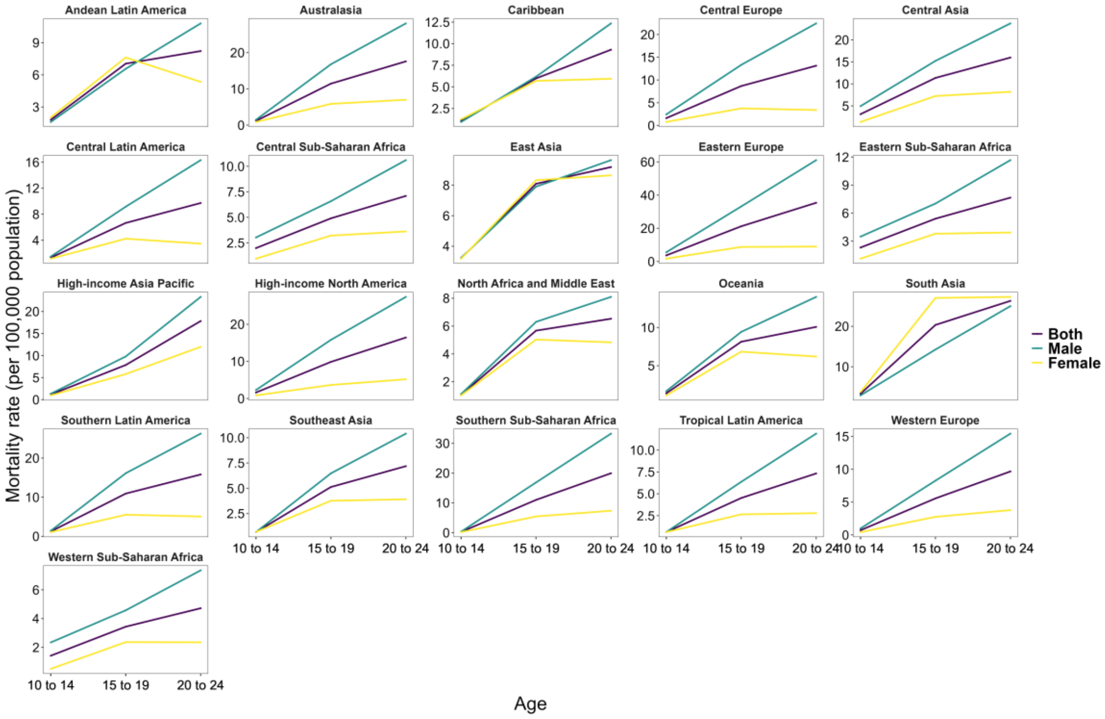

Figure S20. The age effects of suicide mortality in 21 regions, 1990-2021

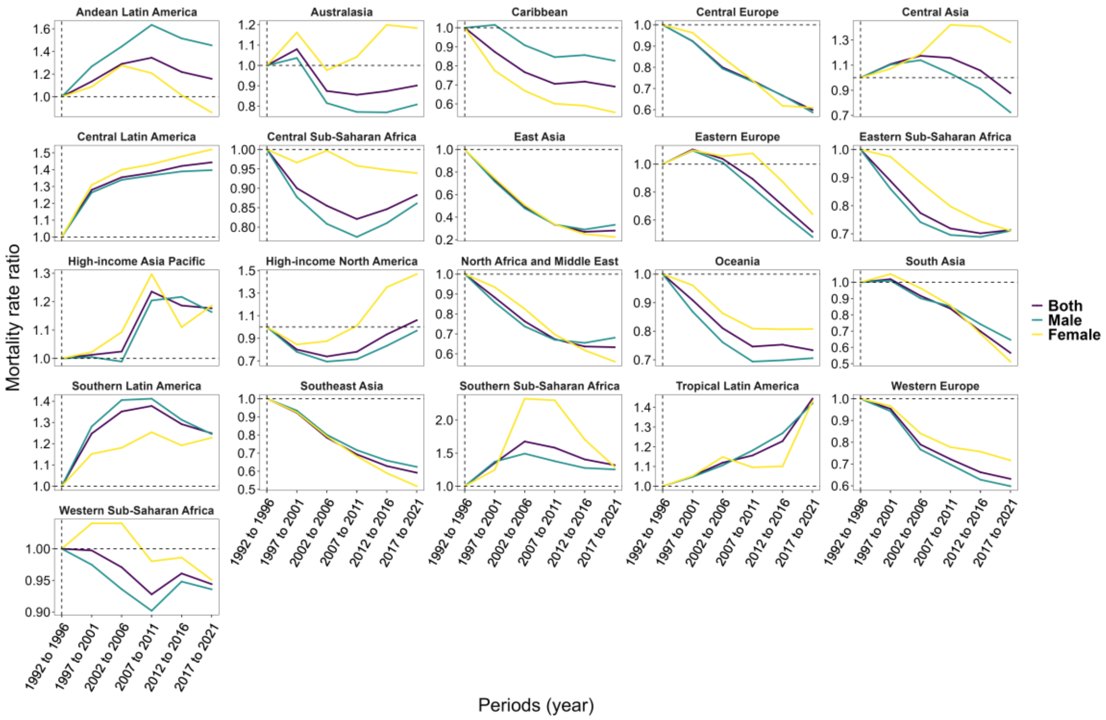

Figure S21.The period effects of suicide mortality in 21 regions, 1990-2021

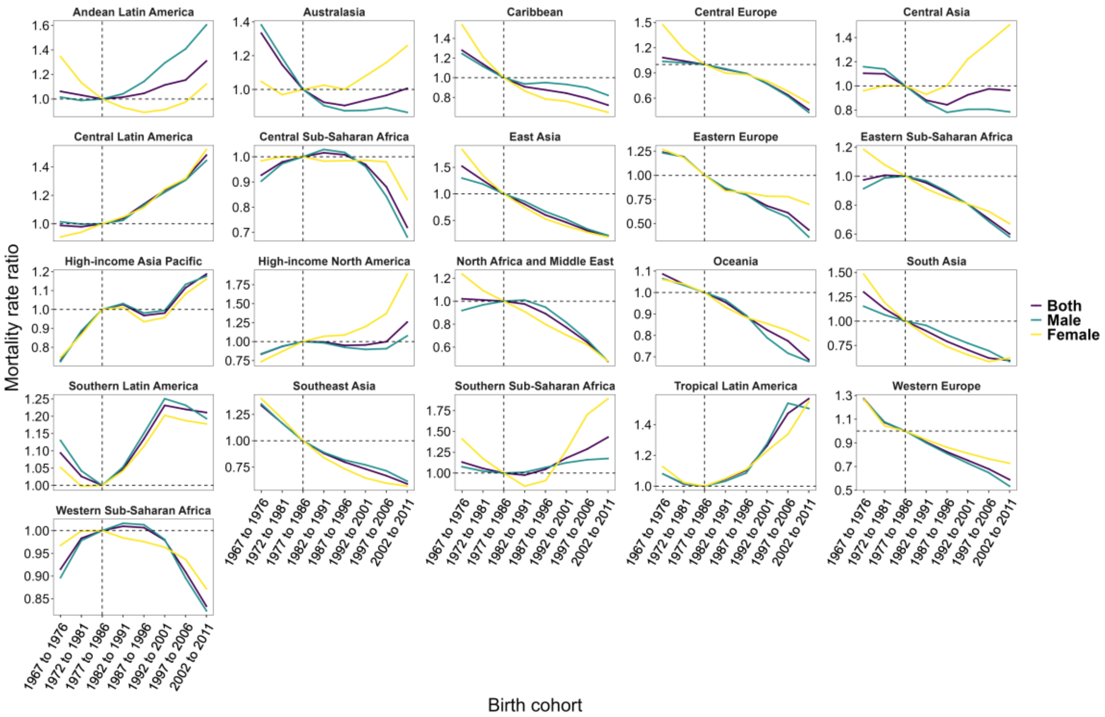

Figure S22. The cohort effects of suicide mortality in 21 regions, 1990-2021

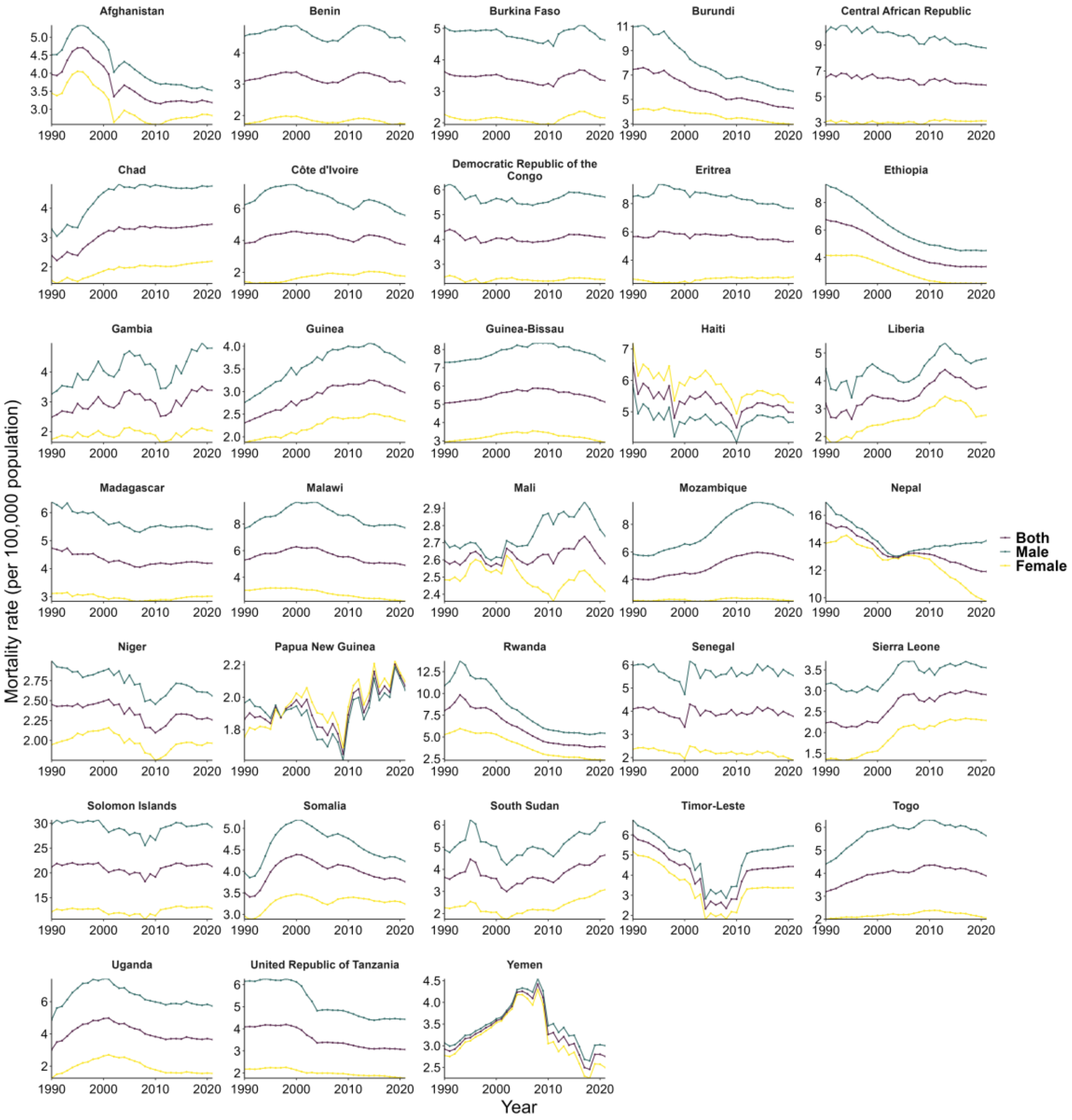

Figure S23. The trends in suicide mortality in low SDI countries, 1990-2021

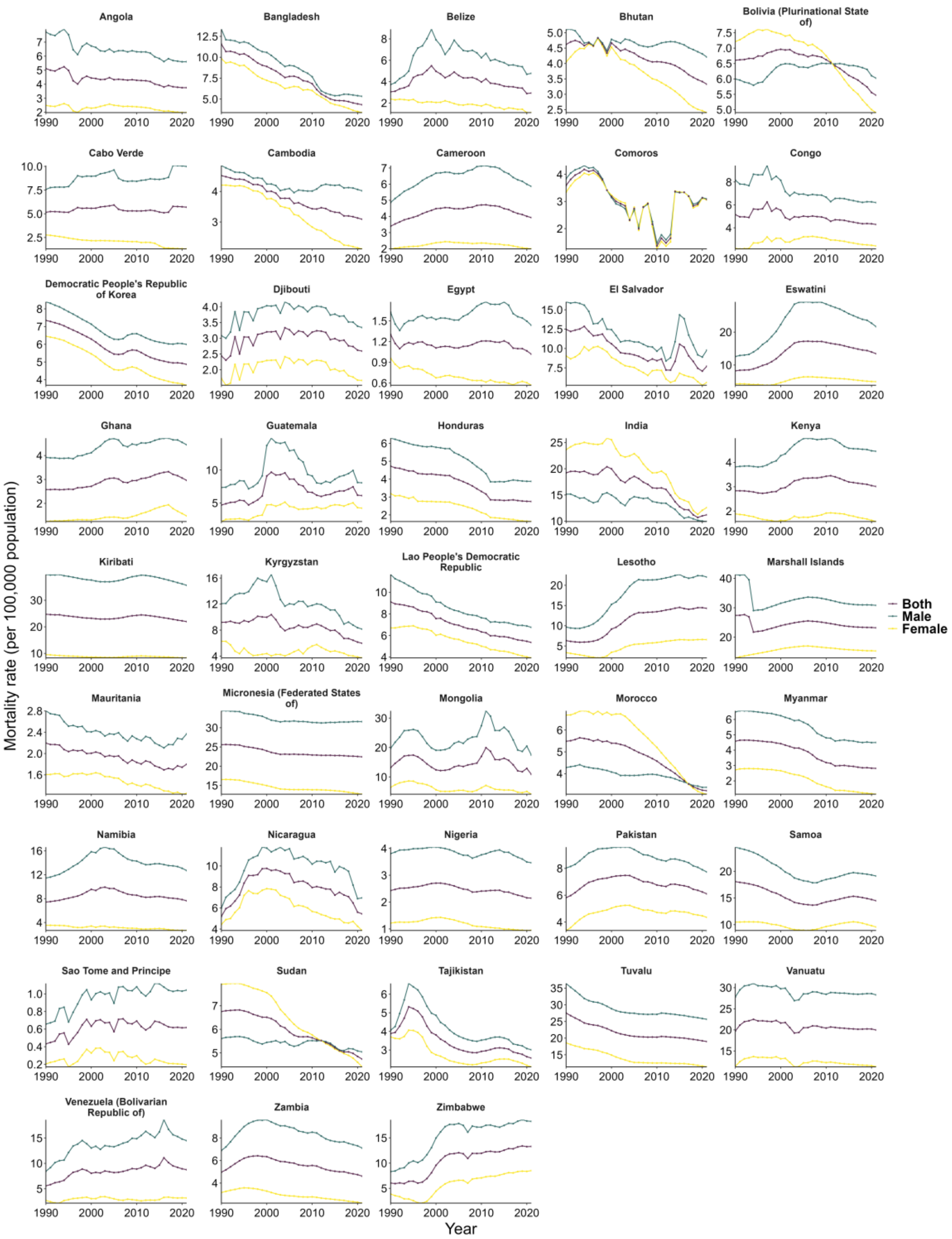

Figure S24. The trends in suicide mortality in low-middle SDI countries, 1990-2021

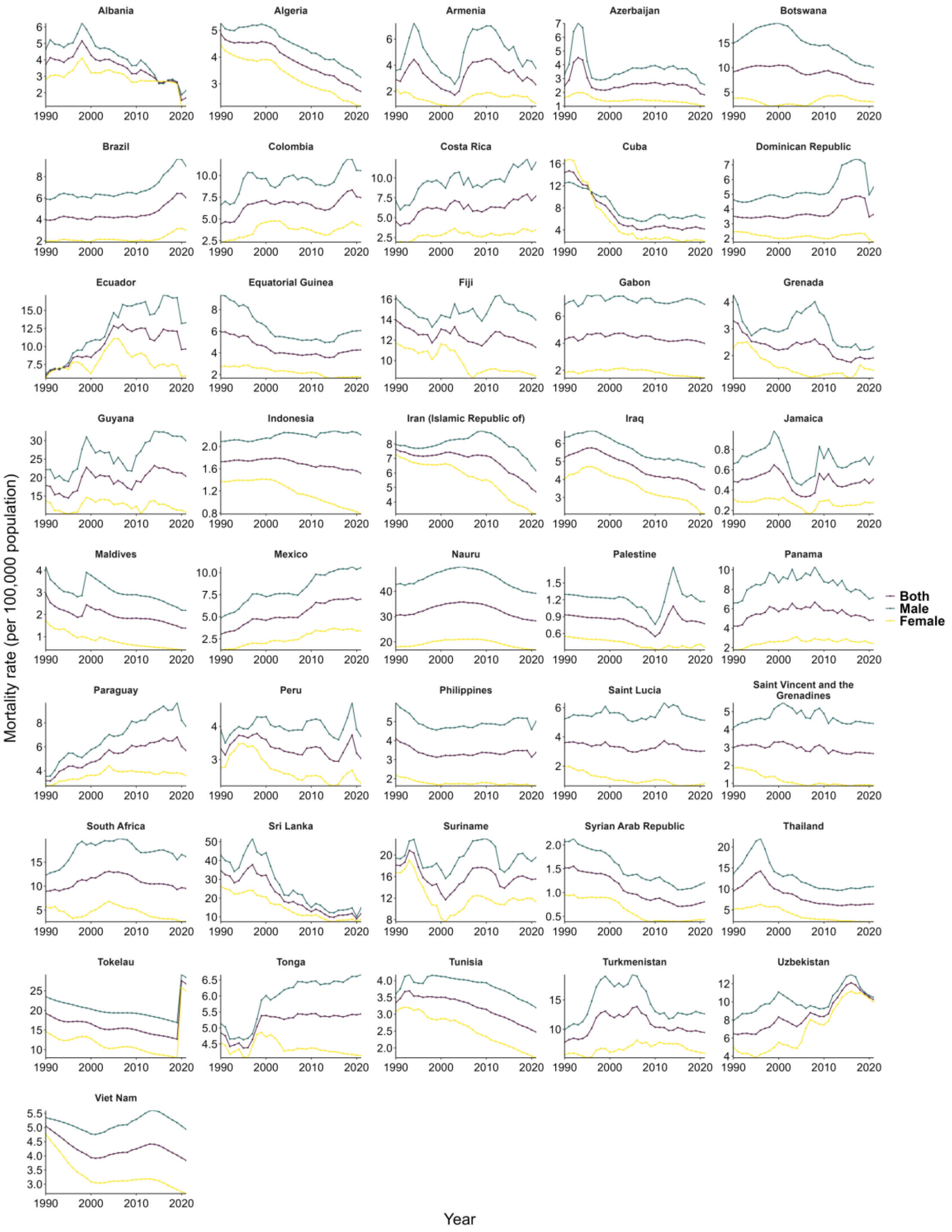

Figure S25. The trends in suicide mortality in middle SDI countries, 1990-2021

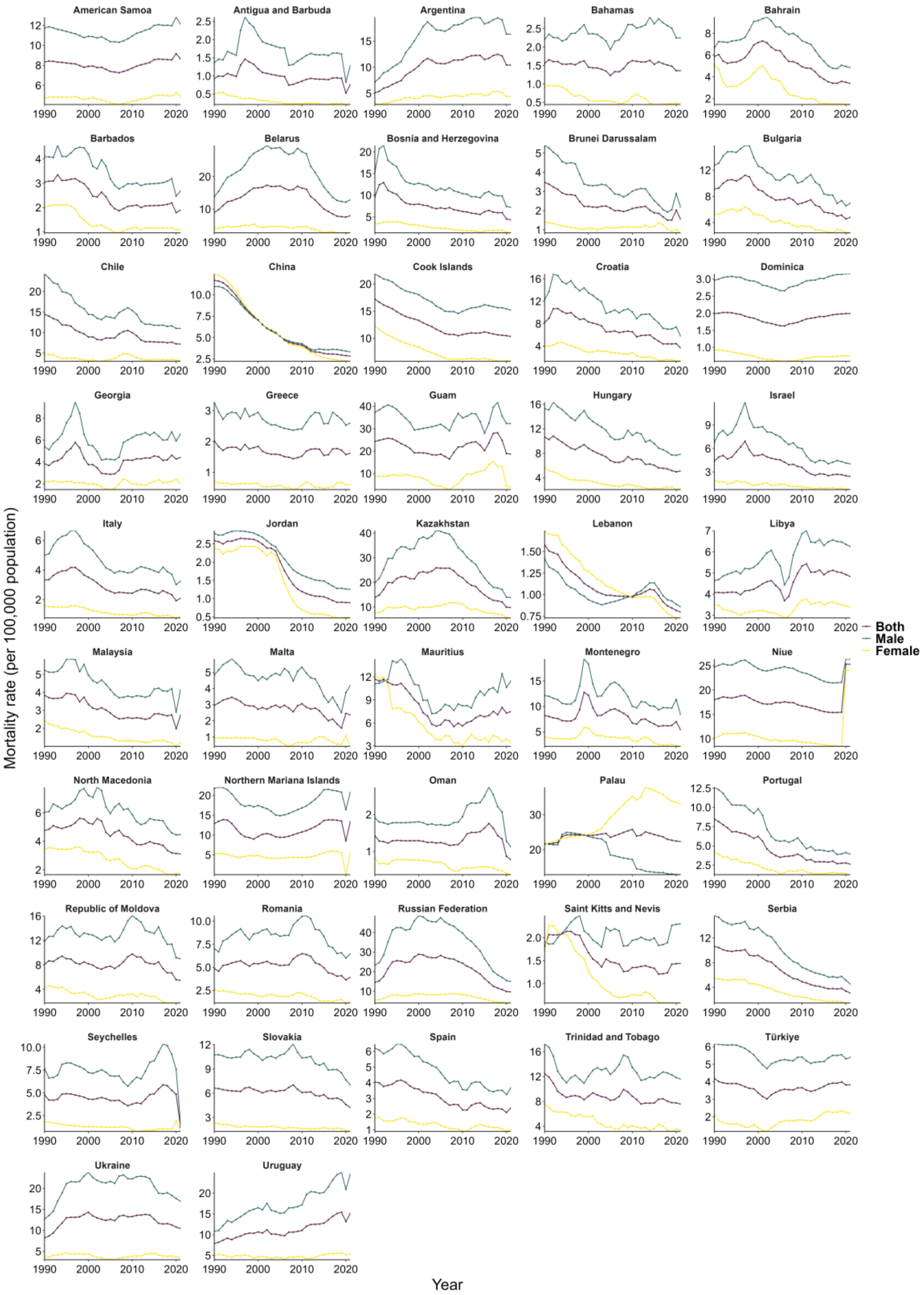

Figure S26. The trends in suicide mortality in high-middle SDI countries, 1990-2021

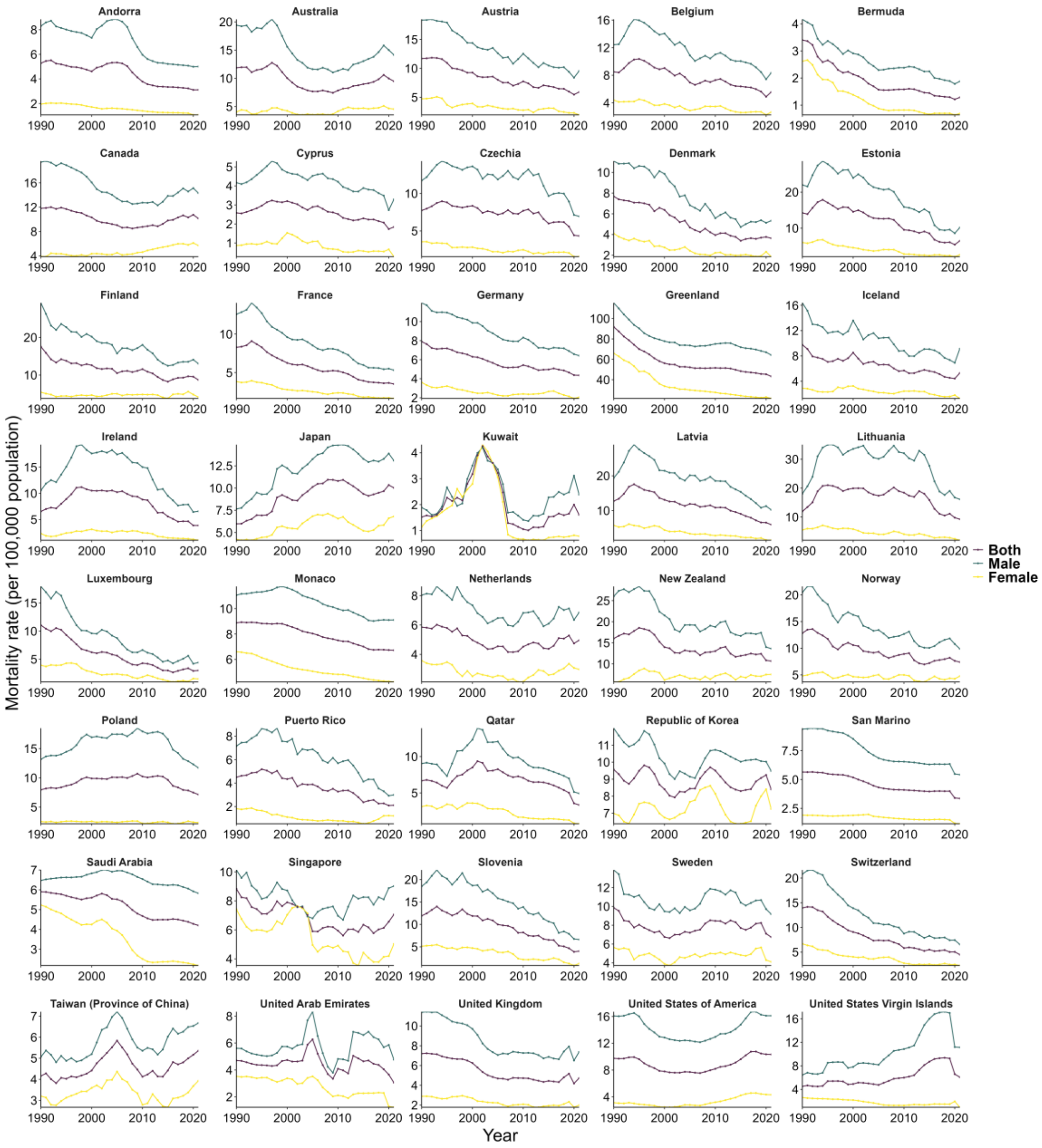

Figure S27. The trends in suicide mortality in high SDI countries, 1990-2021

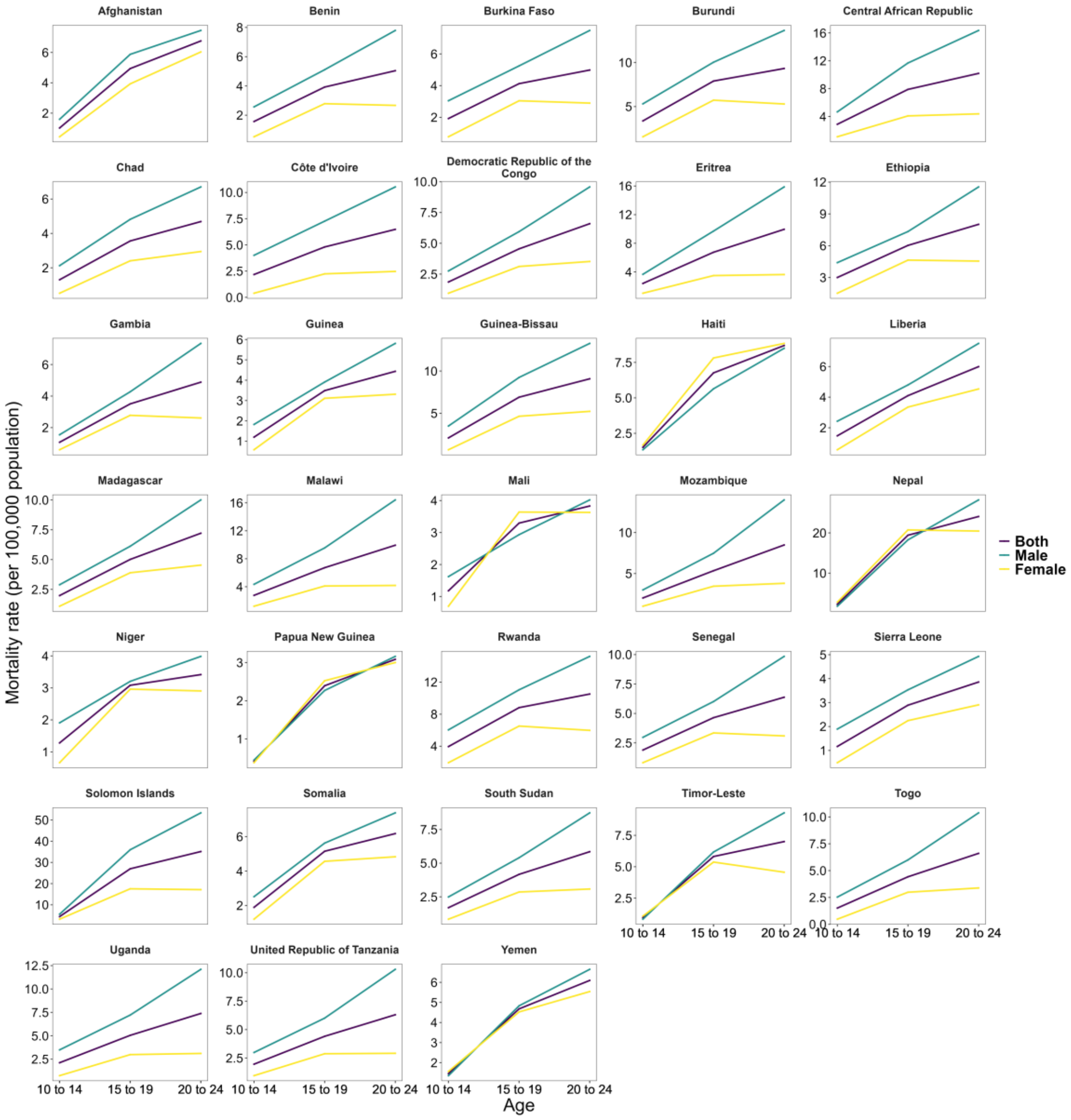

Figure S28. The age effects of suicide mortality in low SDI countries, 1990-2021

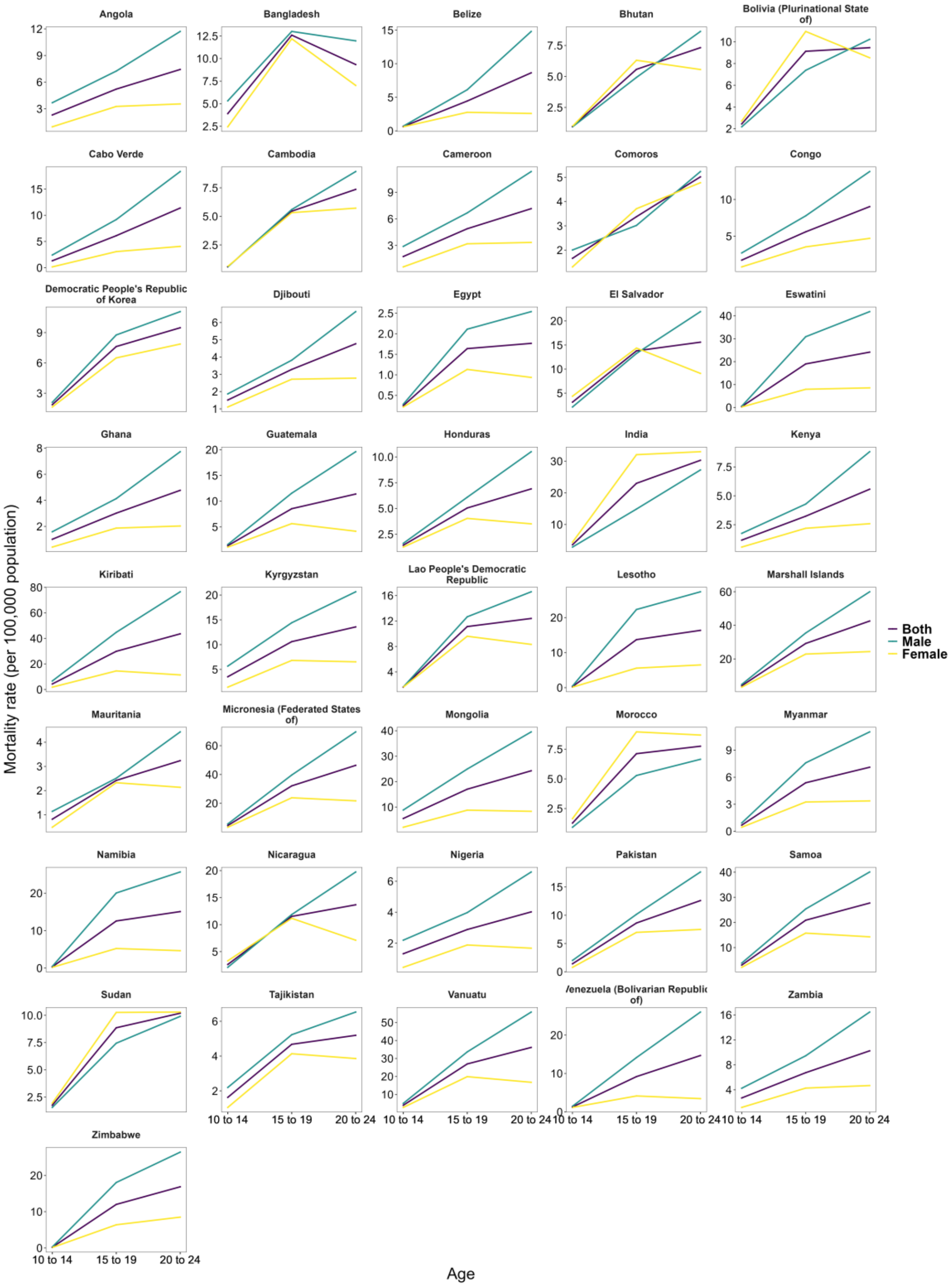

Figure S29. The age effects of suicide mortality in low-middle SDI countries, 1990-2021

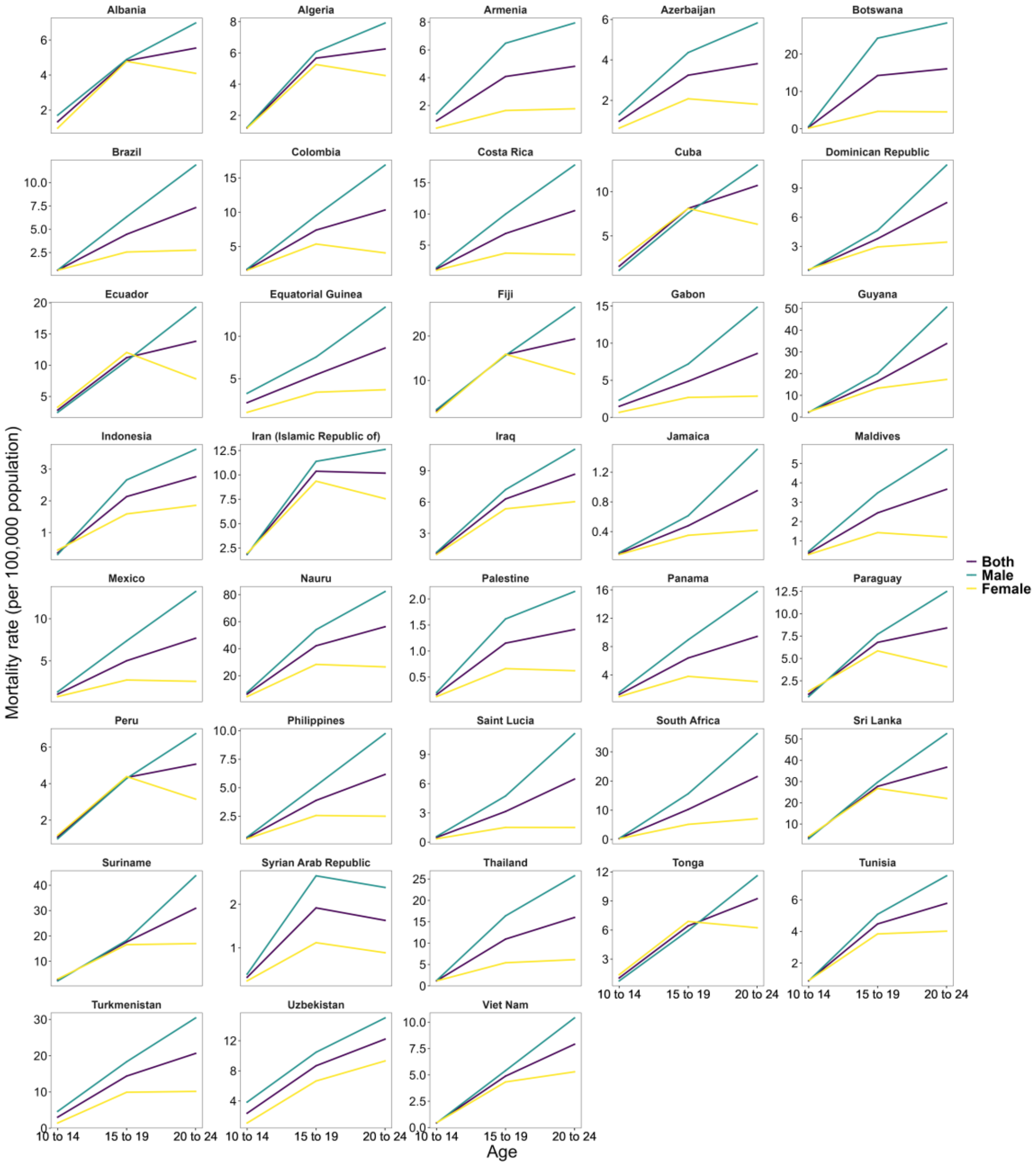

Figure S30. The age effects of suicide mortality in middle SDI countries, 1990-2021

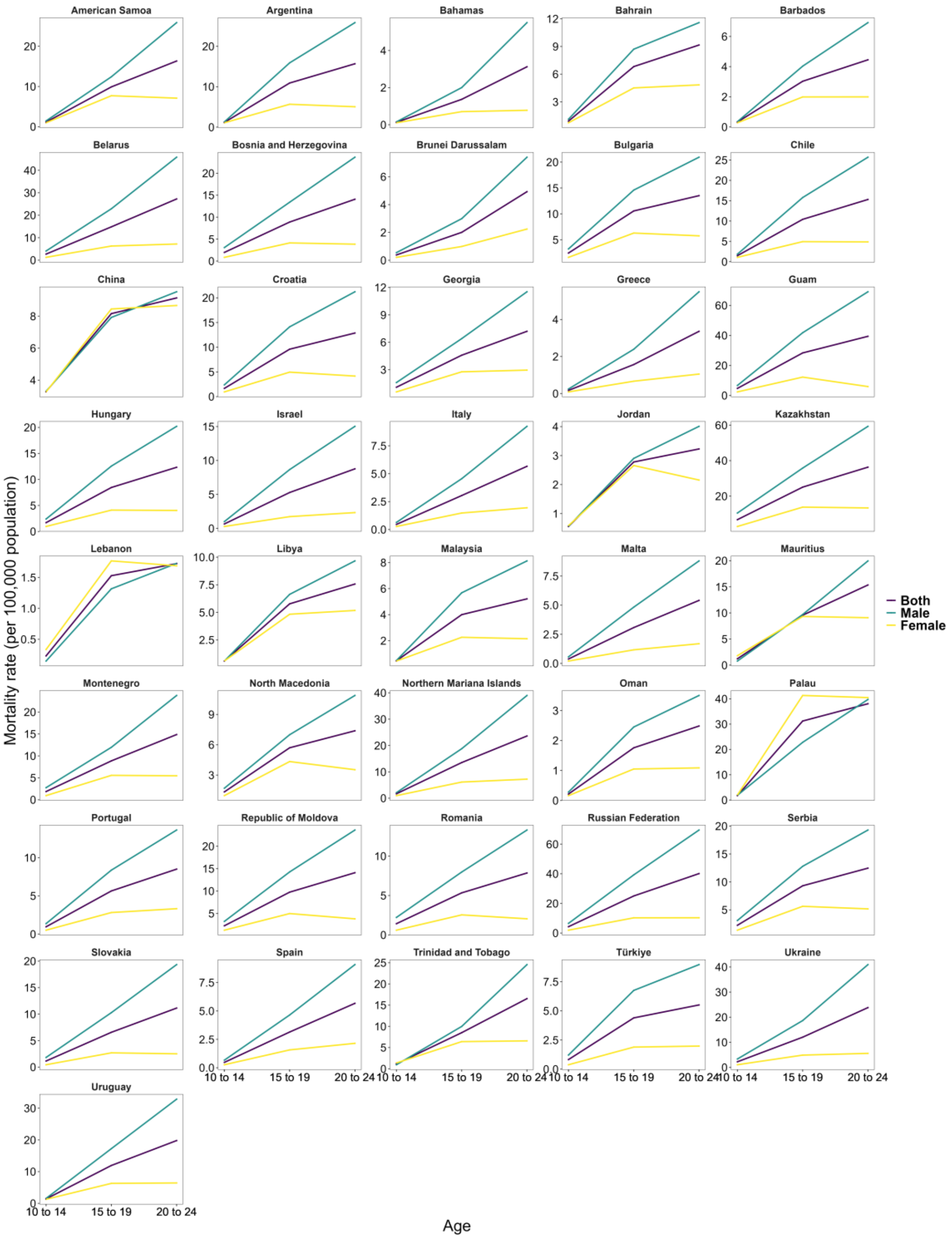

Figure S31. The age effects of suicide mortality in high-middle SDI countries, 1990-2021

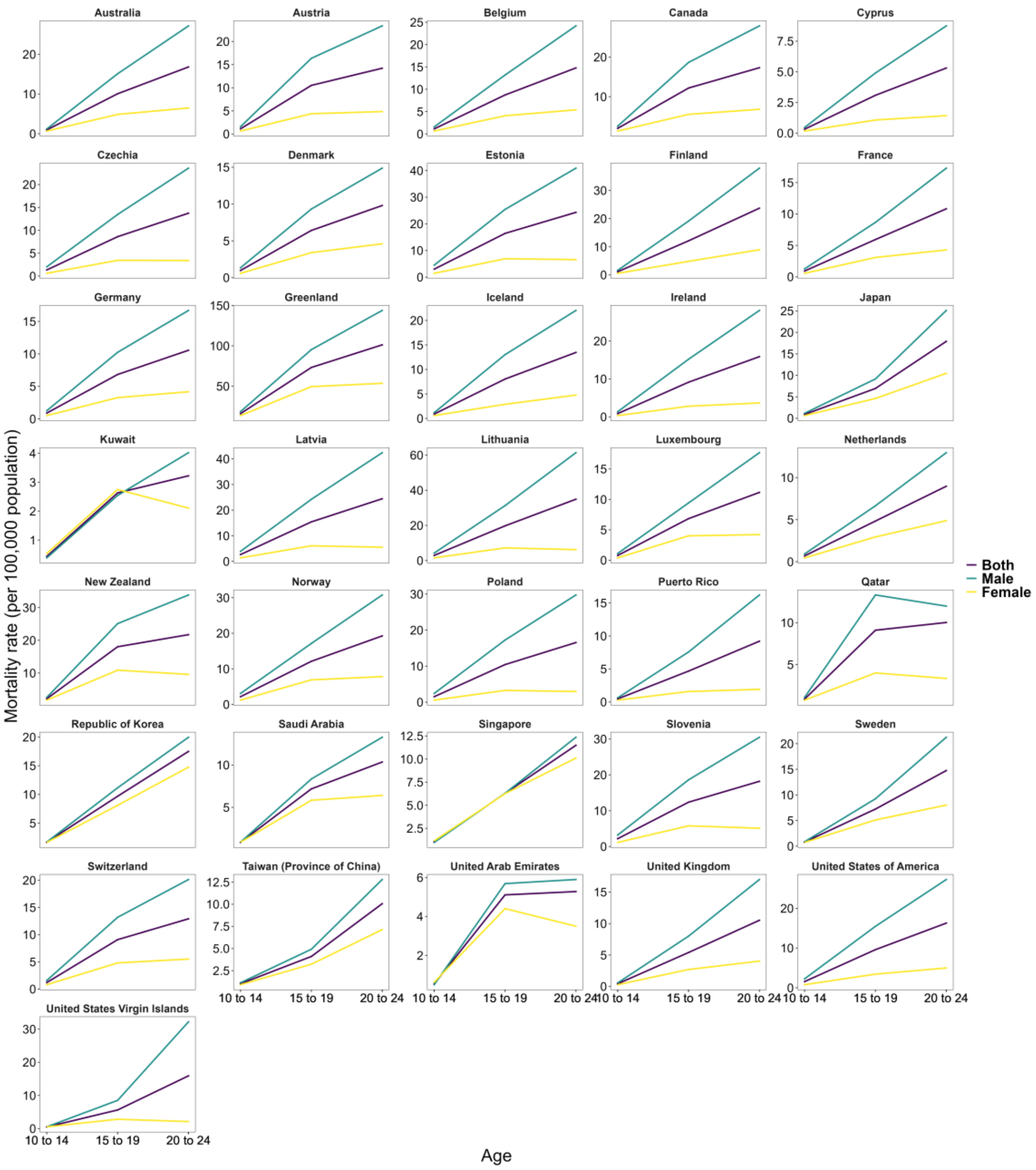

Figure S32. The age effects of suicide mortality in high SDI countries, 1990-2021

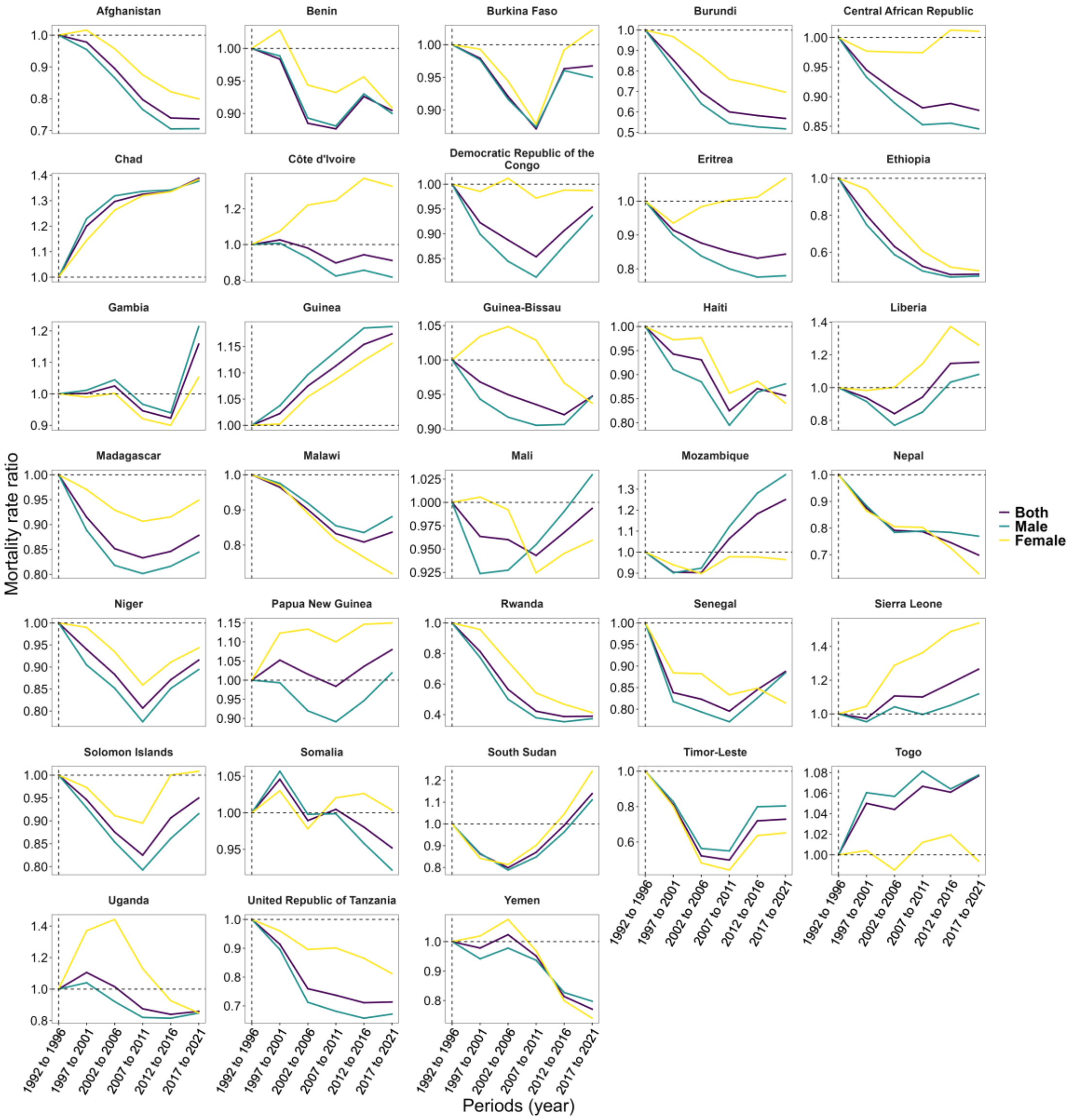

Figure S33. The period effects of suicide mortality in low SDI countries, 1990-2021

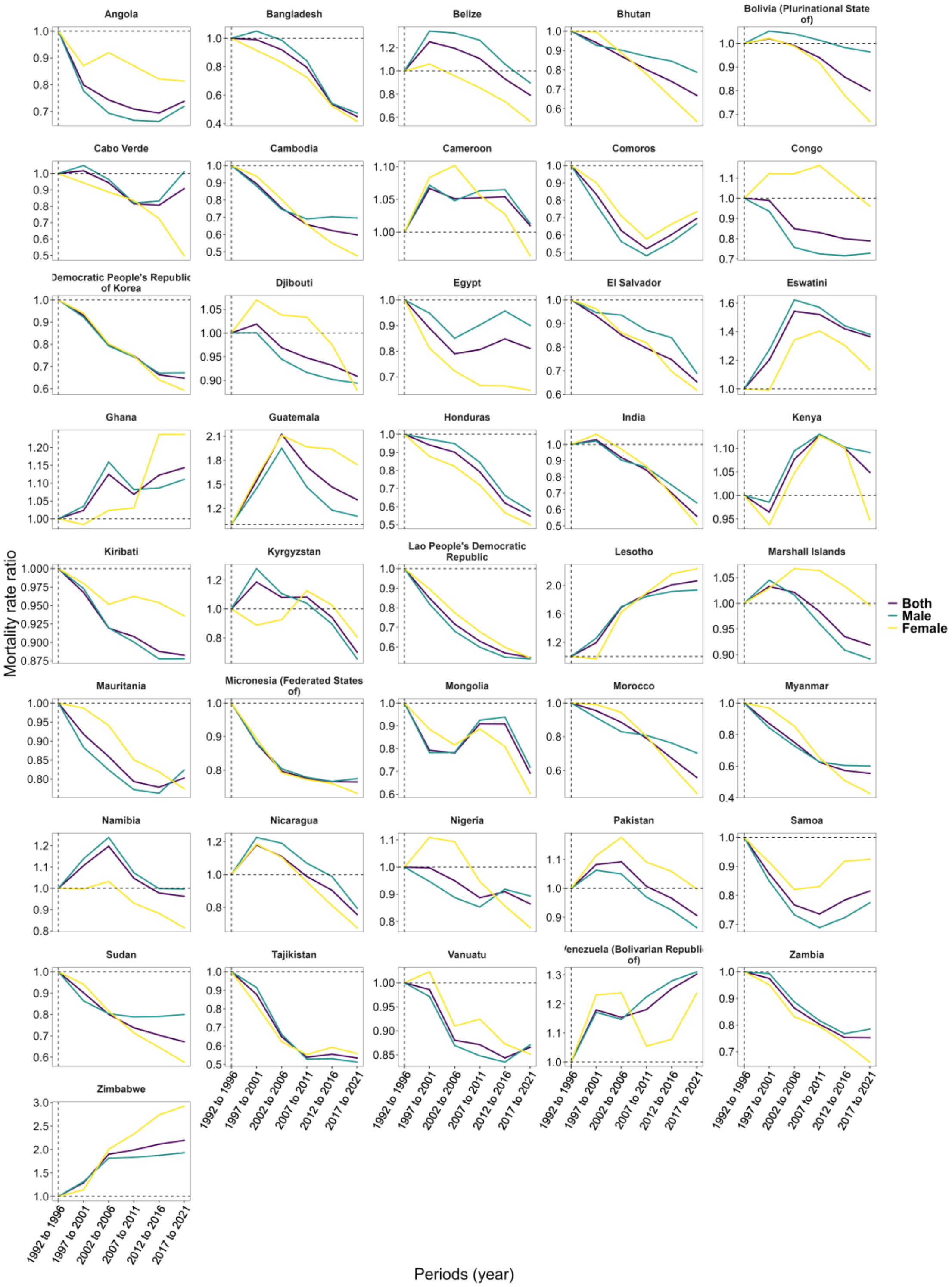

Figure S34. The period effects of suicide mortality in low-middle SDI countries, 1990-2021

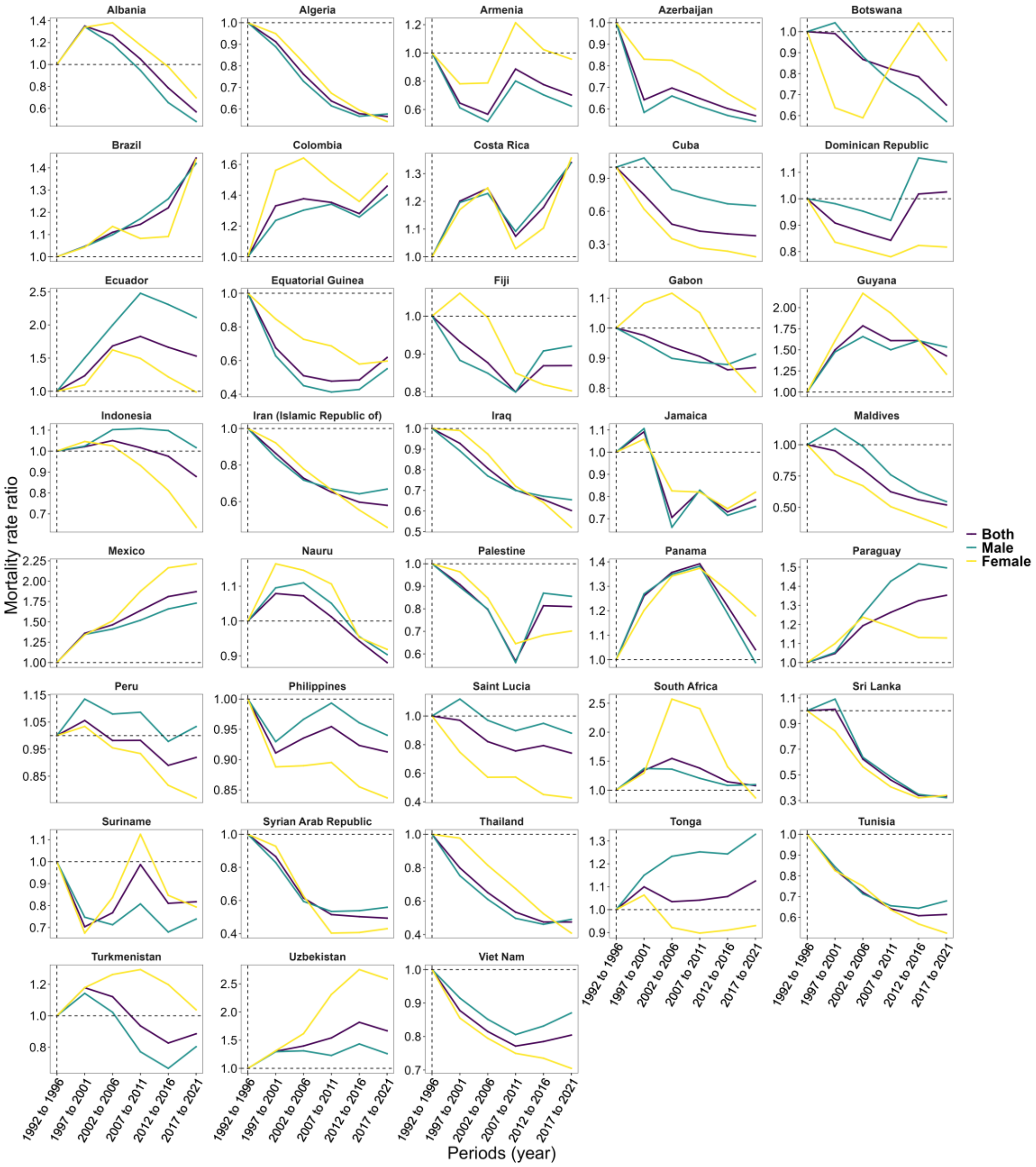

Figure S35. The period effects of suicide mortality in middle SDI countries, 1990-2021

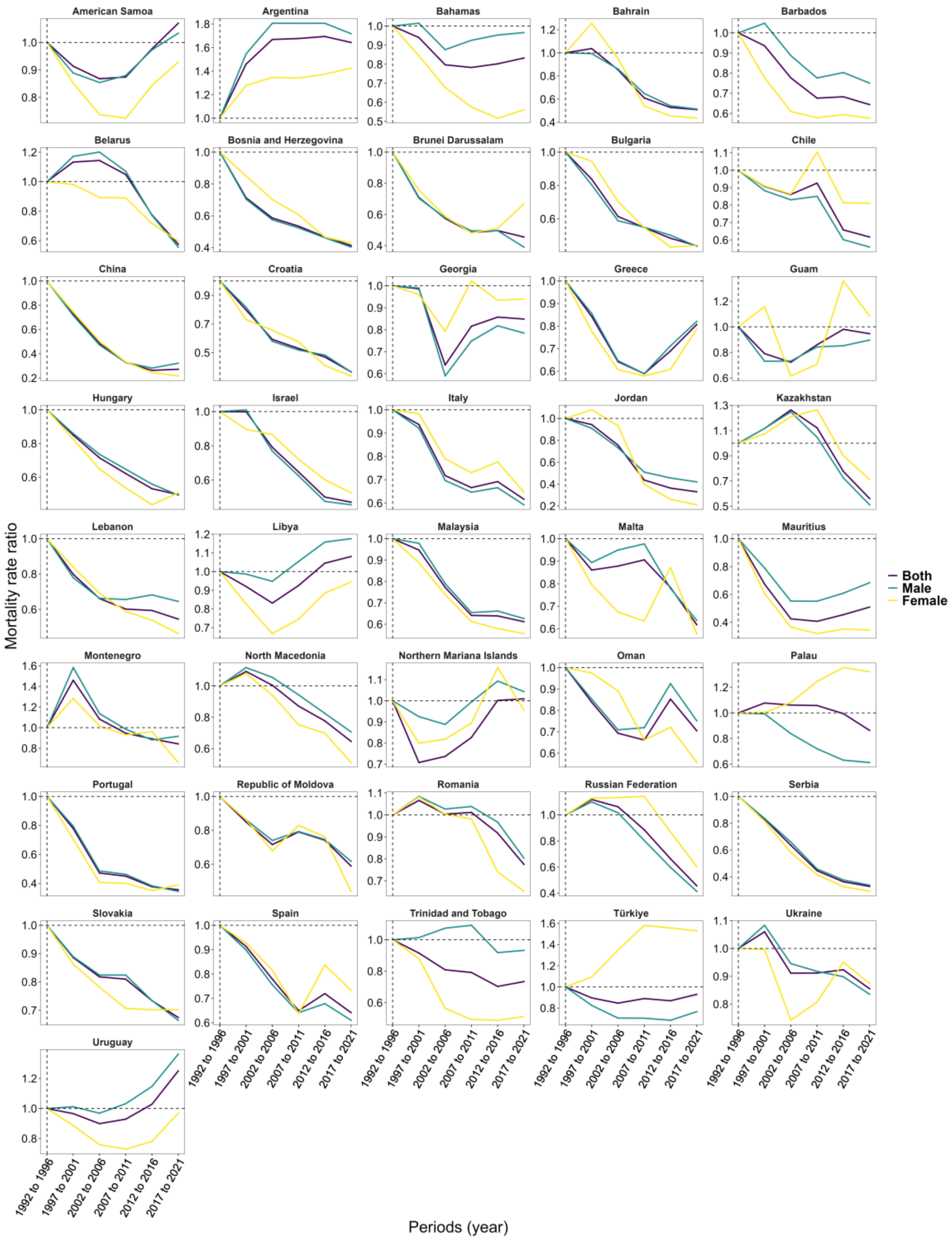

Figure S36. The period effects of suicide mortality in high-middle SDI countries, 1990-2021

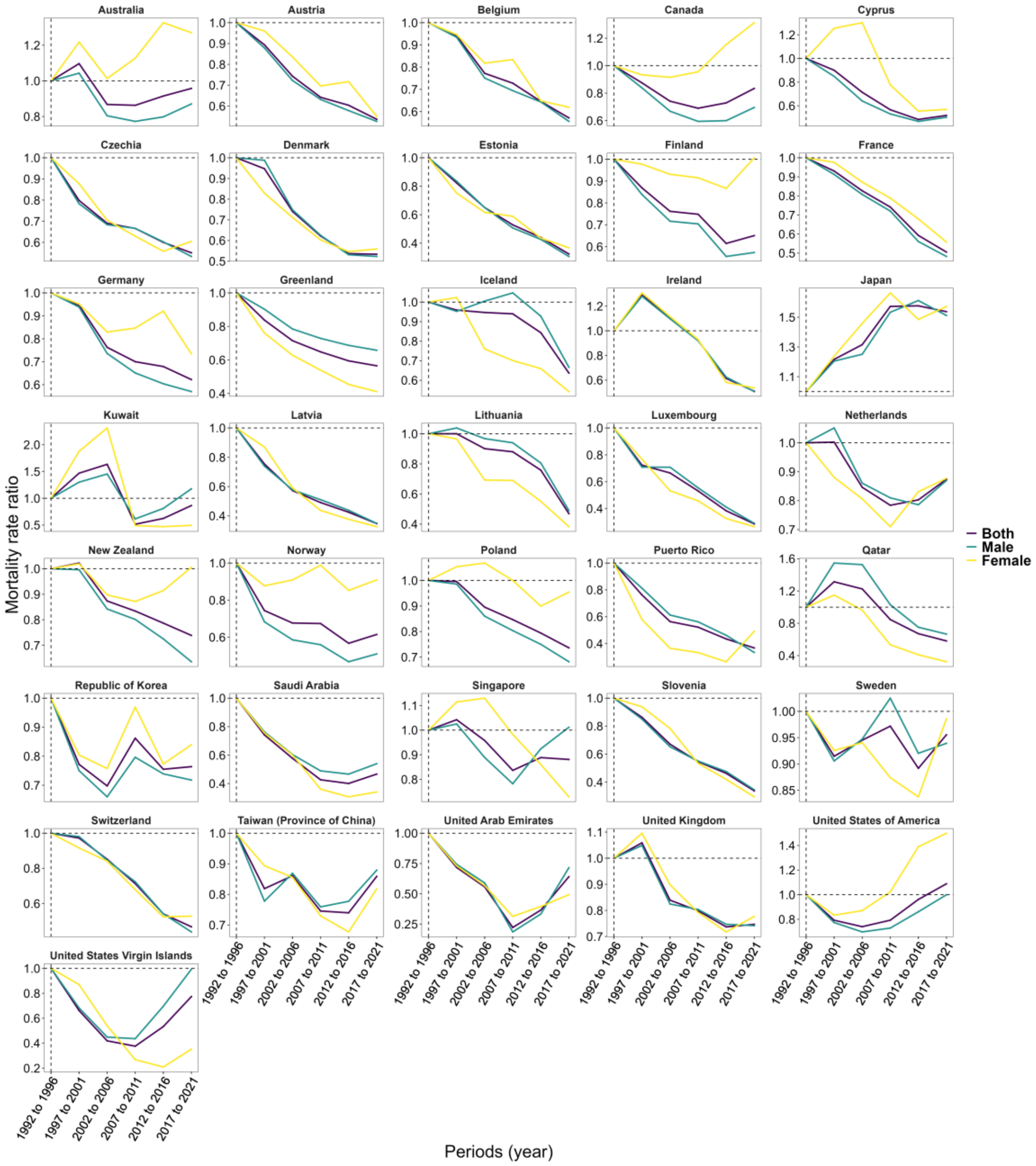

Figure S37. The period effects of suicide mortality in high SDI countries, 1990-2021

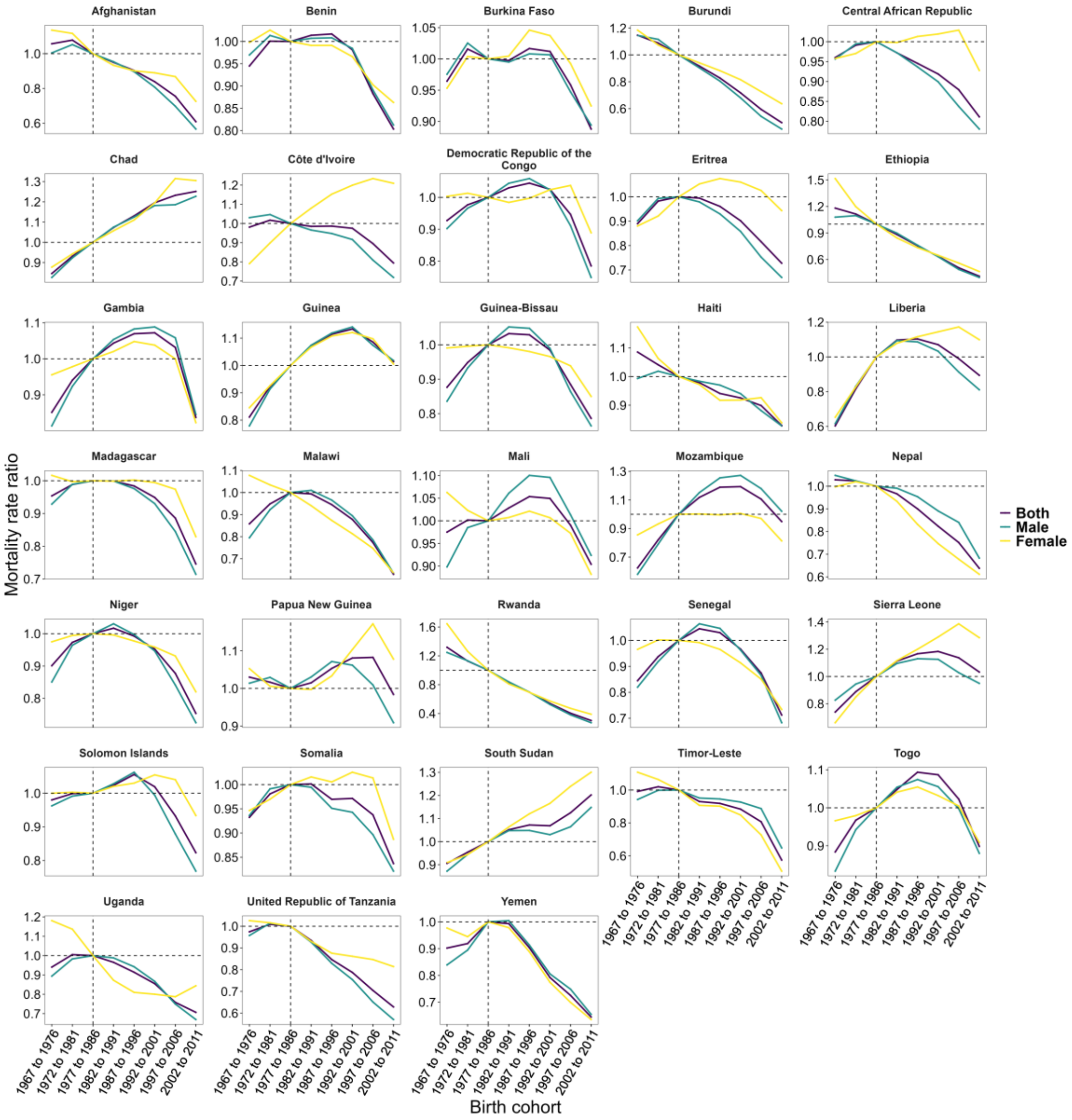

Figure S38. The cohort effects of suicide mortality in low SDI countries, 1990-2021

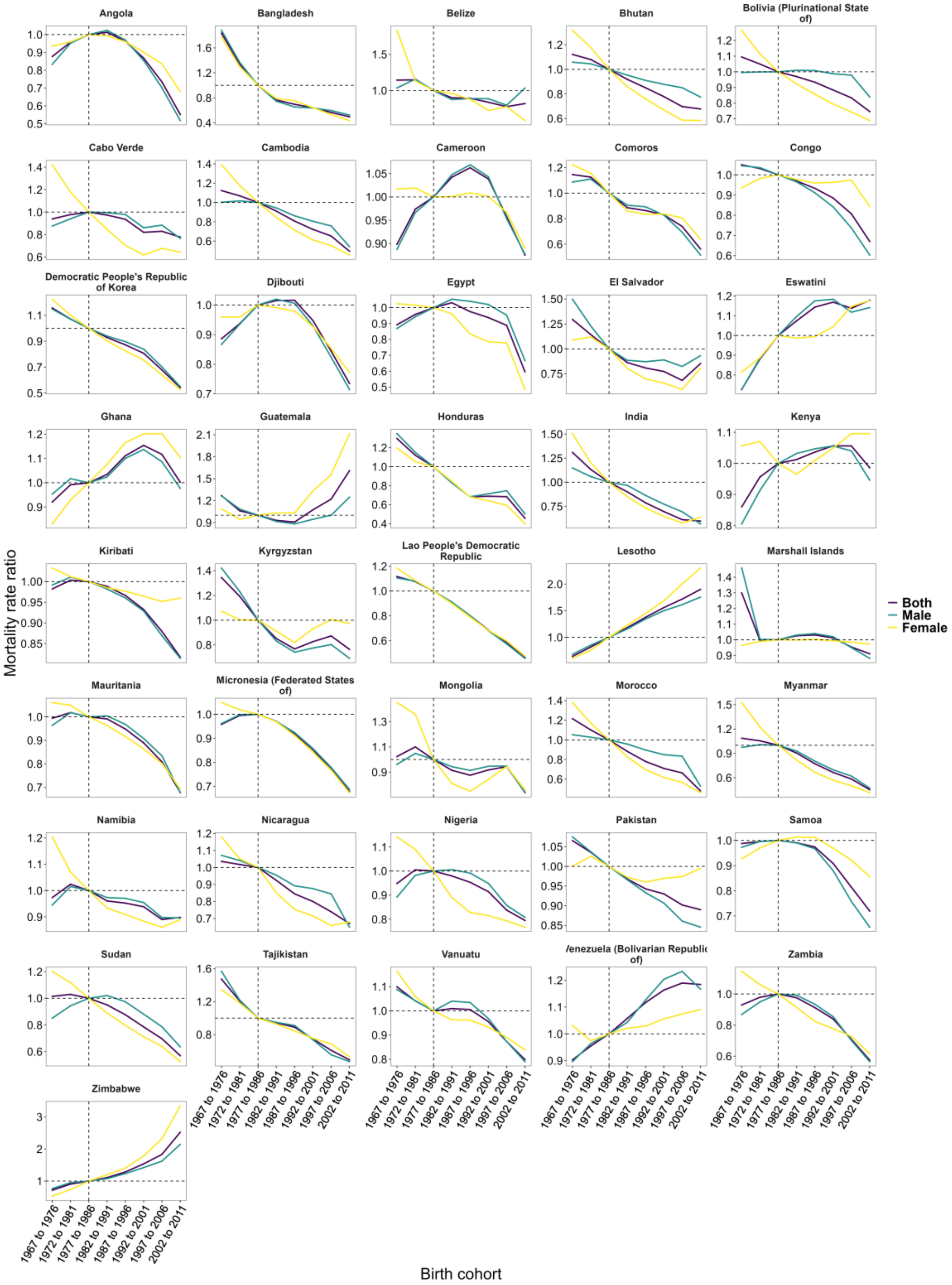

Figure S39. The cohort effects of suicide mortality in low-middle SDI countries, 1990-2021

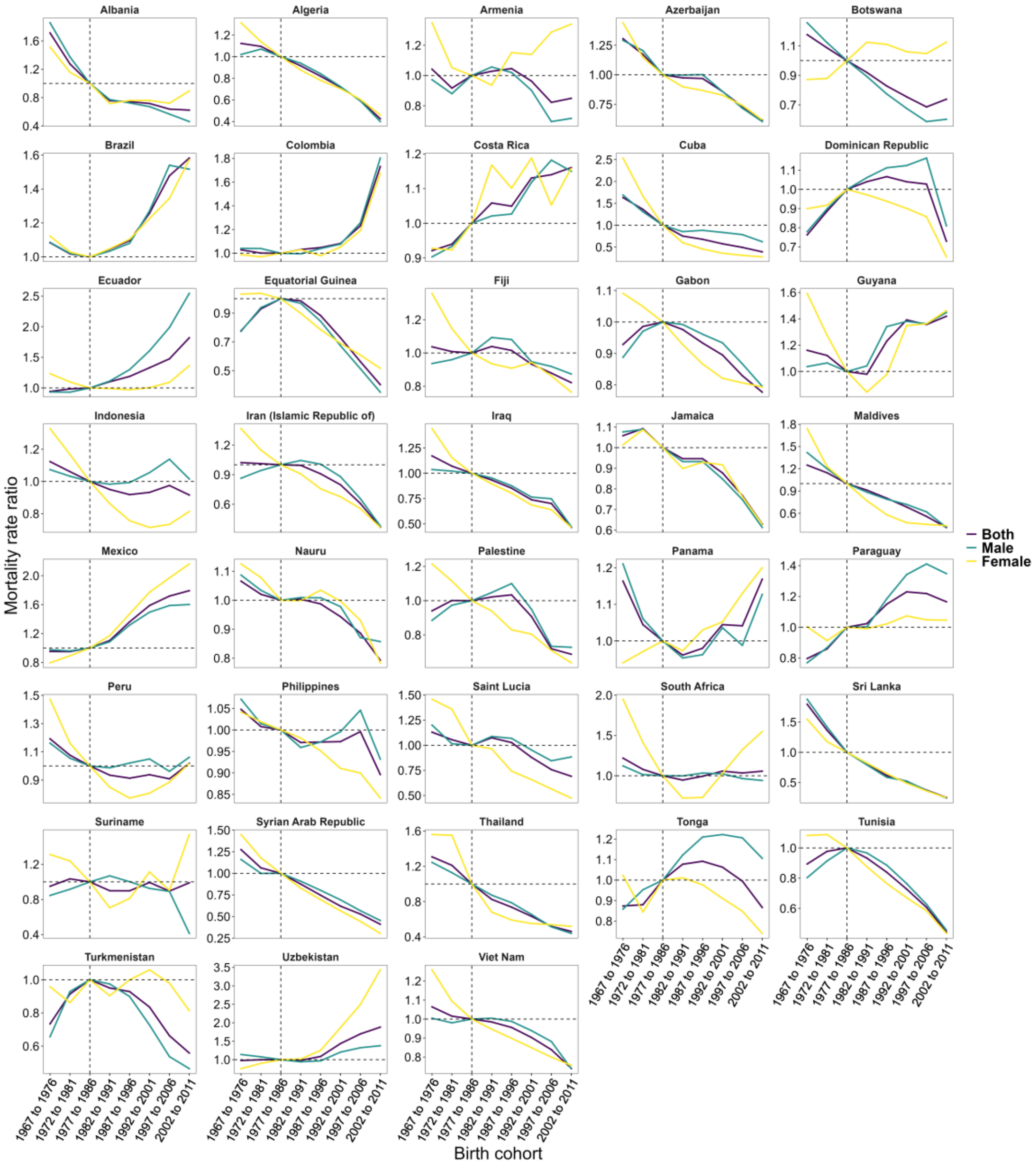

Figure S40. The cohort effects of suicide mortality in middle SDI countries, 1990-2021

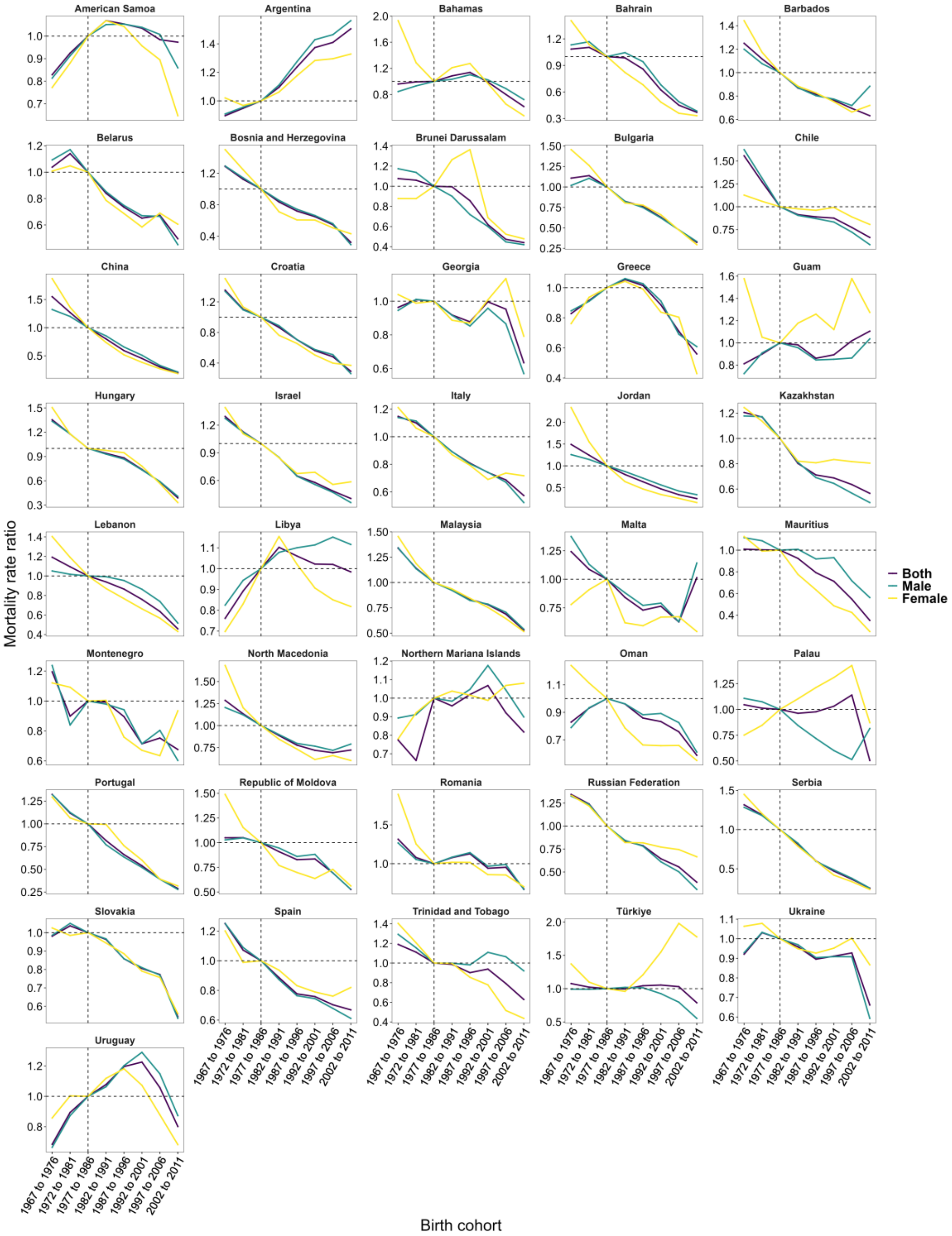

Figure S41. The cohort effects of suicide mortality in high-middle SDI countries, 1990-2021

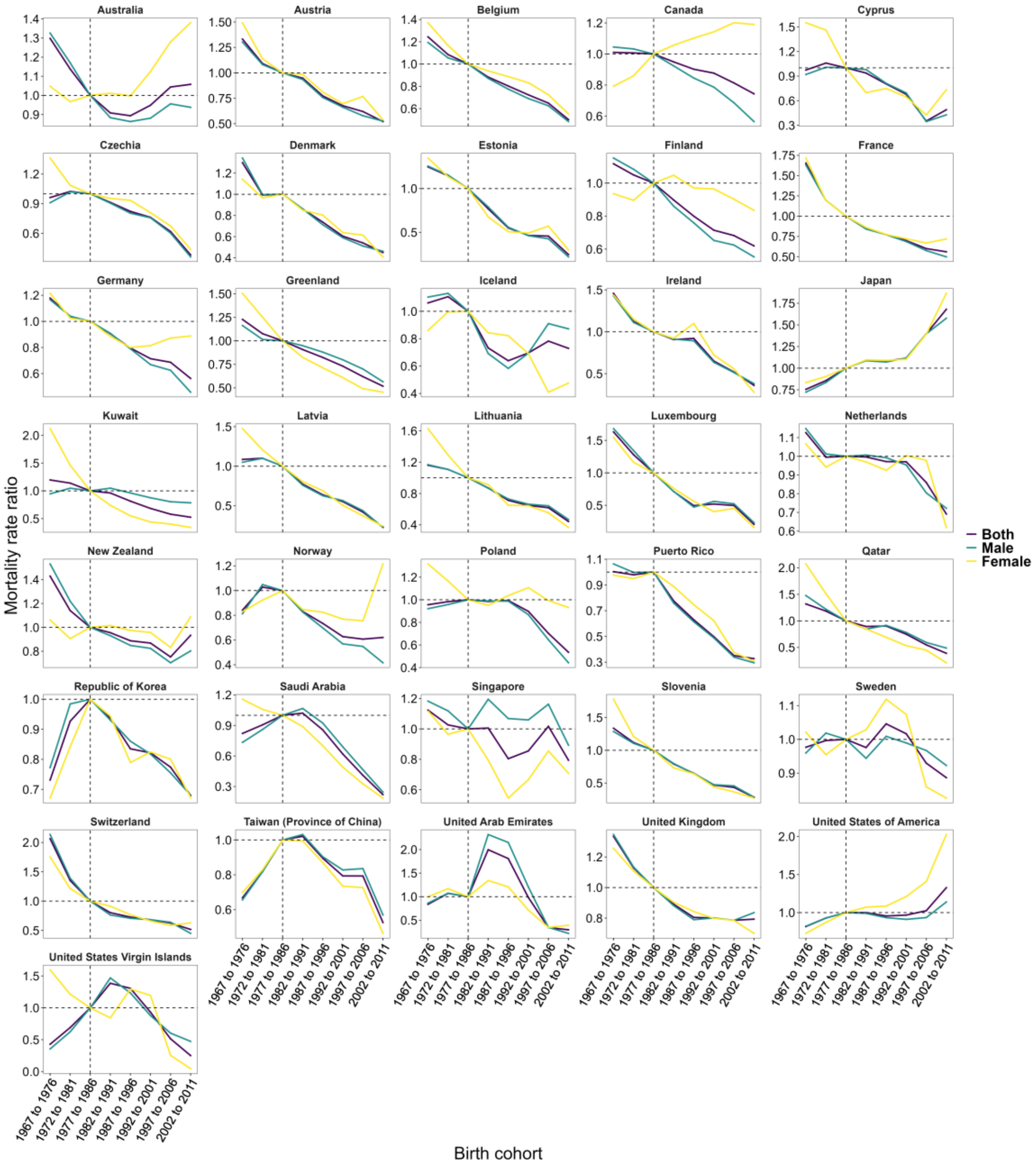

Figure S42. The cohort effects of suicide mortality in high SDI countries, 1990-2021
